# Hypervalent Iodine(III)‐Catalysed Enantioselective α‐Acetoxylation of Ketones

**DOI:** 10.1002/chem.202000927

**Published:** 2020-07-20

**Authors:** Tobias Hokamp, Thomas Wirth

**Affiliations:** ^1^ School of Chemistry Cardiff University Main Building, Park Place Cardiff CF10 3AT UK

**Keywords:** α-acetoxylation, catalysis, hypervalent iodine, ketones, stereochemistry

## Abstract

An enantioselective catalytic synthesis of α‐acetoxylated ketones through I(I)/I(III) catalysis using a resorcinol/lactamide‐based chiral iodoarene is reported. Catalyst turnover by in situ generation of the active iodine(III) derivative is achieved by oxidation with *m*CPBA in the presence of acetic acid. The prior transformation of ketones to easily accessible acetyl enol ethers is beneficial and yields up to 97 % with enantioselectivities up to 88 % *ee* are obtained using only low catalyst loadings of only 5 mol % under mild reaction conditions.

## Graphical abstract

**An efficient and easy acetoxylation** of ketones via their enolethers is described. With low catalyst loadings of a chiral aryliodine high stereoselectivities can be obtained under mild reaction conditions.

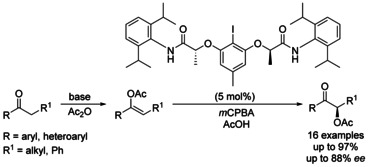

Hypervalent iodine compounds have attracted great attention in the area of modern synthetic chemistry as they are environmentally and economically benign alternatives to transition‐metal reagents.^1^ The highly electrophilic character of the iodine centre in combination with the excellent leaving group ability of the aryliodonio group is the key feature for their unique reactivity.^2^ Their synthetic applications include alkene functionalisations,^3^ oxidations of sulfides,^4^ phenolic oxidations^5^ and rearrangement reactions.^6^ Especially the synthesis of enantioenriched α‐oxygenated carbonyl compounds represents a highly relevant transformation mediated by hypervalent iodine chemistry as the resulting molecules are versatile building blocks for natural products and pharmaceuticals.^7^ Numerous chiral iodoarenes **1** have been developed to realise catalytic enantioselective α‐oxygenations (Figure 1). The first example was reported by Wirth et al., who utilised iodoarene **1 a** in the α‐oxytosylation of propiophenone (up to 28 % *ee*).^8^ Later on, Zhang and co‐workers designed spirobiindane‐based iodoarene **1 b** to increase the enantioselectivity of α‐oxytosylated ketones up to 58 % *ee*,^9^ while Legault et al. developed iodoarene **1 c** to obtain similar results (up to 54 % *ee*).^10^ More recently, Masson and co‐workers achieved enantiomeric excesses of up to 68 % *ee* by applying non‐*C*_2_‐symmetrical iodoarene **1 d**,^11^ while Nachtsheim et al. developed triazole‐substituted iodoarene **1 e**, which delivered 88 % *ee* in the direct α‐oxytosylation of propiophenone.^12^ Nevertheless, enantioselectivities remain mostly moderate. Hence, a practical method has been developed by Legault et al., who converted enol acetates into α‐oxytosyl ketones with high enantioselectivities (up to 90 % *ee*).^13^ However, this protocol requires an excess of chiral iodoarene **1 f**, which is a drawback from a practical and economic point of view. While enantioselective α‐oxytosylations of carbonyl compounds are extensively described, enantioselective α‐oxygenations including other nucleophiles remain scarce in the literature.7b Surprisingly, the α‐acetoxylation of ketones is one of the least described α‐oxygenation reactions although it is the oldest of all hypervalent iodine(III)‐mediated α‐oxygenation reactions.^14^, ^15^

**Figure 1:**
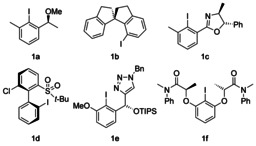
Exemplary chiral organocatalysts for the α‐oxygenation of ketones.

Herein, we report the design of the first highly enantioselective α‐acetoxylation of ketones mediated by iodine(I/III) catalysis. Generation of the active hypervalent iodine catalyst is mediated by *m*‐chloroperbenzoic acid (*m*CPBA) as terminal oxidant in combination with acetic acid.

Although the focus of this work is on the development of a catalytic use of iodoarenes, initial investigations were carried out with stoichiometric amounts of iodine(III) reagents **6** to examine the suppression of possible side reactions with different terminal oxidants. Firstly, propiophenone **2**, silyl enol ether **3** and acetyl enol ether **4 a** were oxidised in presence of lactate/resorcinol‐based chiral hypervalent iodine(III) reagent **6 a** and BF_3_⋅OEt_2_ to product (*R*)‐**5 a** (Table 1, entries 1–3) under optimised reaction conditions (see Supporting Information). It showed that the easily accessible acetyl enol ether **4 a** led to the best result (99 % yield, 66 % *ee*). Reacting **4 a** with lactamide containing iodine(III) compound **6 b** furnished the identical stereoselectivity (66 % *ee*) accompanied by a lower yield of 41 % (Table 1, entry 4).

**Table 1:** Screening of ketone derivatives and I(III) reagents for the enantioselective α‐acetoxylation of ketones.^[a]^

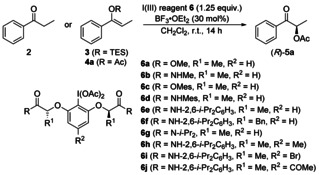				
Entry	Ketone	I(III) reagent	Yield [%]	*ee* [%]^[b]^
1	**2**	**6 a**	6	3
2^[c]^	**3**	**6 a**	91	44
3^[c]^	**4 a**	**6 a**	99	66
4	**4 a**	**6 b**	41	66
5	**4 a**	**6 c**	78	64
6	**4 a**	**6 d**	69	85
7	**4 a**	**6 e**	70	85^[d]^
8	**4 a**	**6 f**	61	77
9	**4 a**	**6 g**	26	70
10	**4 a**	**6 h**	79	85
11	**4 a**	**6 i**	76	89
12	**4 a**	**6 j**	75	87

[a] Reactions were carried out with 0.30 mmol of **2**, **3** or **4 a**, 0.38 mmol of **6** and 0.09 mmol of BF_3_⋅OEt_2_ in CH_2_Cl_2_ (1.5 mL) at room temperature for 14 h.[b] Enantiomeric excesses were determined by chiral‐phase HPLC analysis. [c] Reaction time: 3 h, mixture was gradually warmed from −78 °C to room temperature. [d] A reaction run for 24 h showed identical enantioselectivity.

An increase of the steric of the lactate‐based iodine(III) reagent (**6 c**) did not have an effect on the stereoselectivity (64 % *ee*, entry 5), while a bulkier secondary amide (**6 d** and **6 e**) gave significantly higher enantiomeric excesses (85 % *ee*) with good yields (69 % and 70 %, entries 6 and 7). Importantly, a reaction with **6 e** for 24 hours provided identical enantioselectivity, thus a racemisation of the product under the reaction conditions can be excluded. Changing the methyl to a benzyl substituent (**6 f**) and the use of hypervalent iodine(III) compound **6 g** containing a tertiary amide led to a decrease of the enantiomeric purity (77 % and 70 % *ee*), while the yields were found to be low to moderate (26 % and 61 %, entries 8 and 9). Additionally, no major effect of substituents on the central aryl group was observed (75–79 % yield, 85–89 % *ee*, entries 10–12).

With these results in hand, the suitability of the reaction under catalytic conditions was investigated. Using *m*CPBA as a widely applied stoichiometric oxidant in iodine(I/III) catalysis,7d, 16 iodobenzene was employed as catalyst (20 mol %) in the presence of acetic acid to furnish *rac*‐**5 a** in 96 % yield after 1 h.

However, a control experiment in the absence of iodobenzene formed also the product *rac*‐**5 a** within 5 h in 75 % yield, presumably via the initial generation of an epoxide by direct oxidation of **4 a** with *m*CPBA.^17^ Fortunately, kinetic studies through ^19^F NMR spectroscopic analysis using **4 b** in the presence (Method A) or absence of iodobenzene (Method B) demonstrated that the reaction without iodobenzene is comparably slow (Scheme 1). The starting material was consumed within 75 minutes in the presence of iodobenzene, while a reaction without iodobenzene did not reach full conversion within 15 hours. Hence, the transformation of **4** to **5** is facilitated by an in situ generated hypervalent iodine(III) reagent, which makes a stereoselective catalytic iodine(III)‐mediated reaction feasible.

**Scheme 1:**
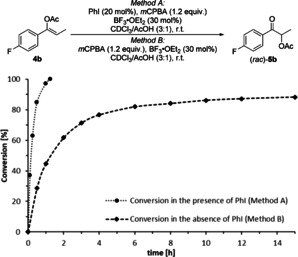
Reaction kinetics of **4 b** in the presence (Method A) and absence of iodobenzene as catalyst (Method B).

This was confirmed by the catalytic transformation of **4 a** into (*R*)‐**5 a** using iodoarene catalysts **7** (Table 2) as precursors of the most promising hypervalent iodine(III) reagents **6 e** and **6 h**–**6 j**. Employing catalyst **7 a** (20 mol %) provided (*R*)‐**5 a** in excellent 94 % yield with 86 % *ee* (Table 2, entry 1), which is similar to the result obtained by applying methyl‐substituted iodoarene **7 b** (96 % yield, 86 % *ee*, Table 2, entry 2). It was noted that bromo‐ and acetyl‐substituted iodoarenes **7 c** and **7 d** also gave high yields (93 %), while the enantioselectivities decreased (83 % and 76 % *ee*, Table 2, entries 3 and 4). The electron‐withdrawing bromo and acetyl substituents presumably raised the oxidation potential of the iodoarenes. Hence, the reaction rate of the undesired direct symmetric oxidation with *m*CPBA increased relative to the desired hypervalent iodine(III)‐mediated stereoselective oxidation, which resulted in lower enantioselectivities. Continuing further studies with iodoarene **7 b**, it was found that the catalyst loading could be reduced to 5 mol % without a loss in yield and enantiomeric purity (Table 2, entries 5–8). Alternative oxidants such as Selectfluor^®^ and sodium perborate did not promote the reaction (Table 2, entries 9–12), while Oxone^®^ provided *rac*‐**5 a** in low yield (<5 %, Table 2, entry 13) and peracetic acid furnished 38 % yield and 49 % *ee* of (*R*)‐**5 a**.

**Table 2:** Optimisation of catalytic reaction conditions.^[a]^

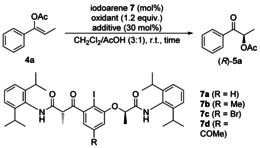						
Entry	Iodoarene (mol %)	Oxidant	Additive	*t* [h]	Yield **5 a** [%]	*ee* **5 a** [%]^[b]^
1	**7 a** (20)	*m*CPBA	BF_3_⋅OEt_2_	2	94	86
2	**7 b** (20)	*m*CPBA	BF_3_⋅OEt_2_	2	96	87
3	**7 c** (20)	*m*CPBA	BF_3_⋅OEt_2_	2	93	83
4	**7 d** (20)	*m*CPBA	BF_3_⋅OEt_2_	2	93	76
5	**7 b** (10)	*m*CPBA	BF_3_⋅OEt_2_	2	92	85
6	**7 b** (5.0)	*m*CPBA	BF_3_⋅OEt_2_	2	90	84
7	**7 b** (2.5)	*m*CPBA	BF_3_⋅OEt_2_	2	93	77
8	**7 b** (1.0)	*m*CPBA	BF_3_⋅OEt_2_	4	93	69
9	**7 b** (5.0)	Selectfluor®	BF_3_⋅OEt_2_	24	0	–
10^[c]^	**7 b** (5.0)	Selectfluor®	BF_3_⋅OEt_2_	24	0	–
11	**7 b** (5.0)	NaBO_3_⋅H_2_O	BF_3_⋅OEt_2_	24	0	–
12^[c]^	**7 b** (5.0)	NaBO_3_⋅H_2_O	BF_3_⋅OEt_2_	24	0	–
13	**7 b** (5.0)	Oxone®	BF_3_⋅OEt_2_	24	<5	0
14	**7 b** (5.0)	AcOOH	BF_3_⋅OEt_2_	24	38	49
15	**7 b** (5.0)	*m*CPBA	TfOH	2	87	79
16	**7 b** (5.0)	*m*CPBA	TMSOTf	2	55	79
17^[d]^	**7 b** (5.0)	*m*CPBA	TsOH⋅H_2_O	2	23	33

[a] Reactions were carried out with 0.3 mmol of **4 a** and 0.09 mmol of BF_3_⋅OEt_2_ in CH_2_Cl_2_ (1.12 mL) and AcOH (0.38 mL). [b] Enantiomeric excesses were determined by chiral‐phase HPLC analysis. [c] 3.0 Equivalents of the oxidant were used. [d] α‐Oxytosylated product was formed (28 % yield, 87 % *ee*).

Moreover, the use of triflic acid (TfOH) and trimethylsilyl triflate (TMSOTf) as additives gave lower yields (87 % and 55 %) and enantioselectivities (79 % *ee*, Table 2, entries 15 and 16). Interestingly, the addition of *p*‐toluenesulfonic acid monohydrate (TsOH⋅H_2_O) delivered (*R*)‐**5 a** in only 23 % yield and 33 % *ee*, whereas the corresponding α‐oxytosylate was formed in similar yield (28 %) and with high enantioselectivity (87 % *ee*, entry 17). This result indicates that the protocol bears the potential to extend the α‐functionalisation to a broader range of nucleophiles.

With **7 b** showing the highest level of stereocontrol under optimised reaction conditions (Table 2, entry 6), our focus was directed towards the investigation of the substrate scope (Scheme 2). Halogen substituents on the aromatic moiety were tolerated and enol ether **4 b** derived from 4’‐fluoropropiophenone enabled the formation of α‐acetoxylated product (*R*)‐**5 b** in good yield (75 %) and enantioselectivity (78 % *ee*), while a fluorine substituent in 3‐position (**4 c**) and a bromine substituent in 4‐position (**4 d**) gave higher yields of (*R*)‐**5 c** and (*R*)‐**5 d** (97 % and 92 %) with enantiomeric excesses of 88 % and 86 %, respectively. Furthermore, **4 e** with a trifluoromethyl group in 3‐position produced comparable results with 78 % yield and 81 % *ee* of (*R*)‐**5 e** but only after an extended reaction time of 20 hours. The yield dropped to 51 % and the enantioselectivity to 6 % *ee* when a trifluoromethyl group was located in the sterically more demanding 2‐positon in (*R*)‐**5 f**. Moreover, a nitro‐substituted reagent **4 g** provided (*R*)‐**5 g** after 20 h in moderate yield (61 %) and enantiomeric purity (59 % *ee*). Additionally, methyl‐ and *tert*‐butyl‐substituted products (*R*)‐**5 h** and (*R*)‐**5 i** were obtained in 74 % yield with good enantioselectivities (77 % and 72 % *ee*) starting from **4 h** and **4 i**, while the yield of phenyl‐substituted compound (*R*)‐**5 j** increased to 94 % with a similar enantiomeric excess (79 % *ee*). However, the presence of an electron donating methoxy substituent afforded (*R*)‐**5 k** in excellent yield (92 %) but it lowered the stereoselectivity to 48 % *ee*, presumably due to a lower oxidation potential of **4 k**, which would facilitate the undesired direct oxidation with *m*CPBA. Indeed, cyclic voltammetry studies revealed a significantly lower oxidation potential of substrate **4 k** (+1.57 V vs. Ag/AgCl) than of substrate **4 c** (+2.33 V vs. Ag/AgCl; see Supporting Information). The fact that the result could be improved to 76 % *ee* in absence of *m*CPBA by use of iodine(III) reagent **6 h** as stoichiometric oxidant further supports the hypothesis. Next, the effect of substituents in the α‐position other than methyl was explored. Acetyl enol ether **4 l** formed (*R*)‐**5 l** in moderate yield (52 %) and low enantioselectivity (2 % *ee*) after 20 h. A racemisation due to the extended reaction time can be excluded as the reaction under stoichiometric conditions afforded the identical selectivity.

**Scheme 2:**
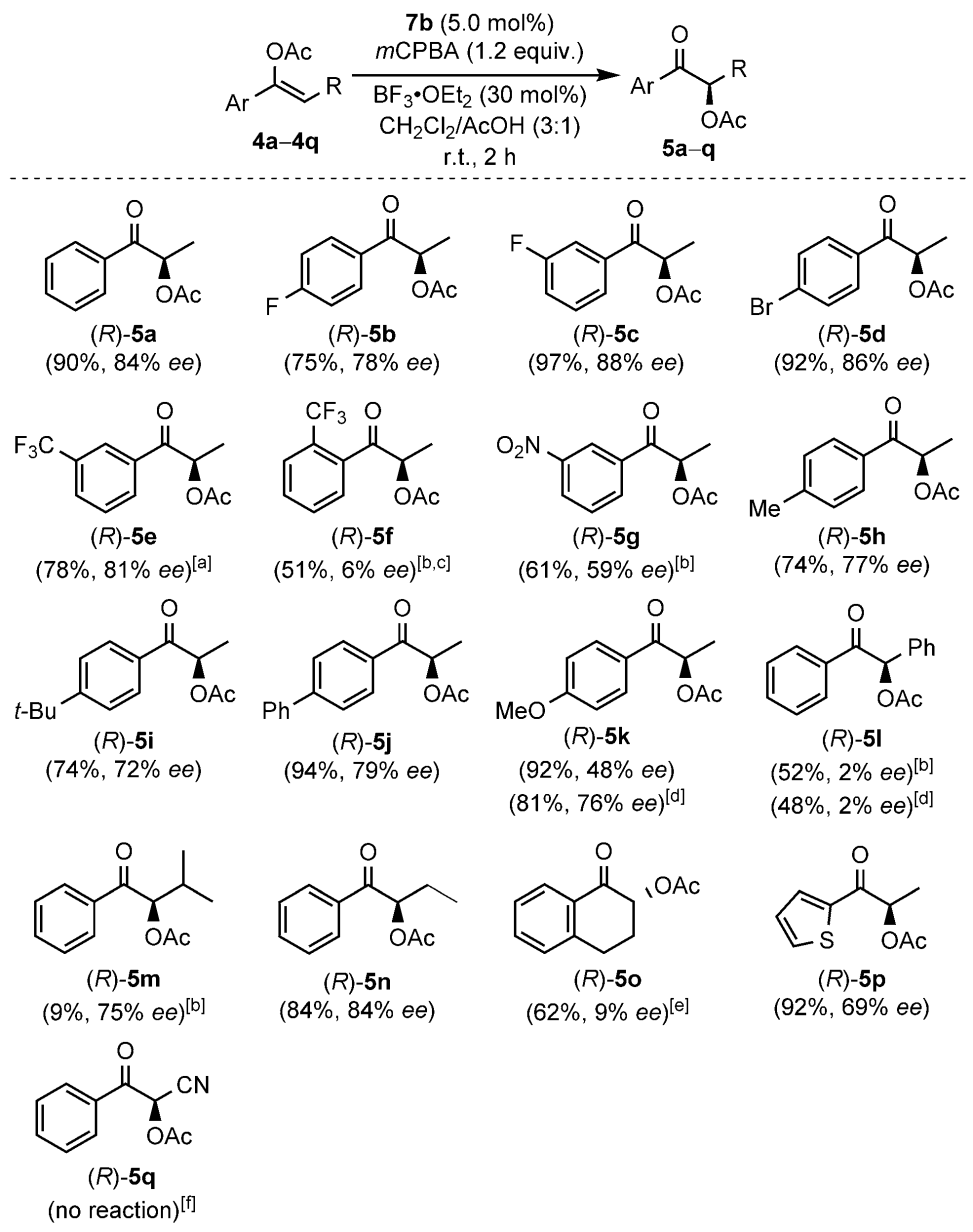
Reaction scope of the stereoselective α‐acetoxylation protocol. Reactions were carried out with 0.30 mmol of **4**, 0.015 mmol of **7 b**, 0.36 mmol of *m*CPBA and 0.09 mmol of BF_3_⋅OEt_2_ in CH_2_Cl_2_ (1.12 mL) and AcOH (0.38 mL). [a] Reaction for 5 h. [b] Reaction for 20 h. [c] **4 f** was received as a mixture of isomers (*Z*/*E=*2.7:1). [d] Yield and enantiomeric excess of the reaction under stoichiometric conditions with **6 h** (1.25 equiv) in CH_2_Cl_2_ (1.5 mL). [e] **4 o** was obtained with (*E*)‐stereochemistry. [f] No reaction even under stoichiometric conditions using **6 h** (1.25 equiv) and BF_3_⋅OEt_2_ (2.0 equiv). Product *rac*‐**5 q** could be synthesised by reaction with (diacetoxyiodo)benzene.

Additionally, α‐isopropyl‐substituted reagent **4 m** yielded (*R*)‐**5 m** in low quantity (9 % yield) but with a good enantiomeric excess of 75 %. On the other hand, α‐ethyl substituted product furnished good yields (84 %) and enantioselectivity (84 % *ee*). Cyclic substrate **4 o** afforded only modest selectivity (9 % *ee*) and moderate yield (62 %). To our delight, thiophene‐containing substrate **4 p** was well tolerated and delivered (*R*)‐**5 p** in 92 % yield with 69 % *ee*. On the contrary, α‐cyano‐substituted reagent **4 q** did not react under the optimised conditions.

Based on previous mechanistic proposals, a catalytic cycle for the iodine(III)‐mediated preparation of compounds was suggested (Scheme 3).^13^, ^15^, ^18^ Chiral iodoarene **7 b** (Ar*I) is oxidised with *m*CPBA to the active catalyst **6 h**, which is activated by boron trifluoride etherate.^19^ The activation enables a reaction with enol ether **4** to generate α‐C‐bound intermediate **8**, followed by an S_N_2 reaction to form the final product (*R*)‐**5** and to regenerate catalyst **7 b**. The high enantioselectivities compared to the corresponding ketone **2** derives from the inaccessibility of an enolate‐type oxygen‐bound iodine(III) intermediate, in which a long distance between the stereocentre of Ar* and the α‐carbon does not allow an efficient stereoinduction.^13^, ^20^

**Scheme 3:**
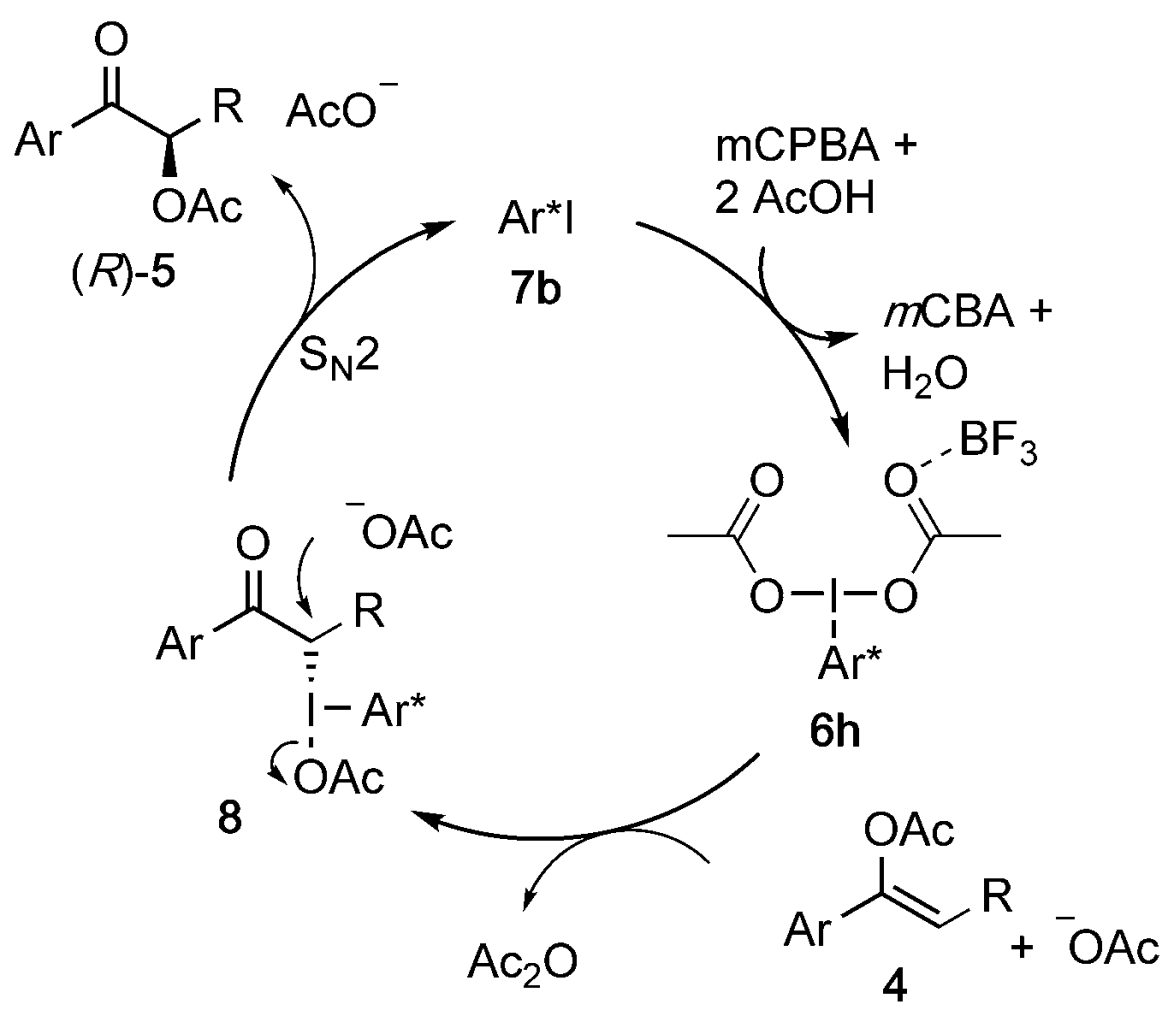
Proposed mechanism for the iodine(III)‐mediated α‐acetoxylation reaction. Active catalyst **6 h** can furthermore undergo ligand exchange with *m*‐chlorobenzoic acid (*m*CBA) and H_2_O.

In summary, we have designed the first enantioselective synthesis of α‐acetoxylated ketones mediated by hypervalent iodine(I/III) catalysis. Using easily accessible acetyl enol ethers and a low catalyst loading of only 5 mol % in combination with *m*CPBA as terminal oxidant and boron trifluoride as Lewis acid provided high yields and enantioselectivities. The extensive optimisation of the iodoarene catalyst based on a resorcinol core revealed that best results were obtained with sterically demanding flexible lactamide side chains. As the reaction rate of the direct oxidation with *m*CPBA is lower relative to the iodine(III)‐mediated oxidation, an enantioselective catalytic transformation was realised.

## Experimental Section

**General procedure for the catalytic asymmetric α‐acetoxylation**: Iodoarene **7 b** (10.7 mg, 0.0150 mmol, 5.0 mol %) and acetyl enol ether **4** (0.30 mmol) were dissolved in CH_2_Cl_2_ (1.12 mL) and AcOH (0.38 mL) under nitrogen atmosphere. After the addition of BF_3_⋅OEt_2_ (11 μL, 0.090 mmol, 30 mol %) and *m*CPBA (81 mg, 0.36 mmol, 1.2 equiv, 77 % purity), the reaction mixture was stirred for 2 h at room temperature. Subsequently, saturated aqueous Na_2_S_2_O_3_ (5 mL) was added and the resulting mixture was extracted with CH_2_Cl_2_ (3×10 mL) The combined organic layers were washed with saturated aqueous NaHCO_3_ (30 mL), dried over anhydrous MgSO_4_, concentrated under vacuum and the crude mixture was purified by flash column chromatography.

## Conflict of interest

The authors declare no conflict of interest.

## Supporting information

As a service to our authors and readers, this journal provides supporting information supplied by the authors. Such materials are peer reviewed and may be re‐organized for online delivery, but are not copy‐edited or typeset. Technical support issues arising from supporting information (other than missing files) should be addressed to the authors.

Supplementary
