# Supplementary material for: Hypervalent Iodine(III)‐Catalysed Enantioselective α‐Acetoxylation of Ketones
Source: Chemistry. 2020 Jul 20;26(46):10417–21. doi: 10.1002/chem.202000927 (PMC7496773; doi:10.1002/chem.202000927)
Supplement: Supplementary file 1 — Supplementary [file CHEM-26-10417-s001.pdf]

Chemistry–A European Journal

Supporting Information

Hypervalent Iodine(III)-Catalysed Enantioselective α-Acetoxylation of Ketones

Tobias Hokamp and Thomas Wirth*[a]

Table of Contents

1 General Methods S2

2 General Procedures S3

3 Synthesis of Enol Ethers S6

4 Synthesis of Hypervalent Iodine Reagents S15

5 Catalytic Asymmetric α-Acetoxylation of Acetyl Enol Ethers S32

6 Kinetic Studies S42

7 NMR spectra S44

8 HPLC Chromatograms S117

9 Cyclic Voltammetry S128

10 Literature S129

1 General Methods

Reactions involving air and moisture sensitive reagents were carried out in flame dried glass wares under a dry nitrogen atmosphere using *Schlenk* technique.

Solvents used in air- or moisture-sensitive reactions were either freshly distilled or dried by employing a solvent purification system *MB SPS-800* from *MBraun* and stored under a nitrogen atmosphere.

Reagents were purchased from *Acros Organics*, *Alfa Aesar*, *Fisher Scientific*, *FluoroChem*, *Merck*, *Sigma Aldrich* and *TCI*. All reagents obtained from commercial sources were used as received unless mentioned otherwise.

^{1}H, ^{13}C, ^{11}B and ^{19}F NMR spectra were recorded at 298 K on a *Bruker DPX 300*, *Bruker DPX 400*, *Bruker DPX 500* or a *Bruker DPX 600*. All resonances are reported relative to TMS. Spectra were calibrated relative to solvents' residual proton and carbon chemical shifts: $CHCl_3$ (δ = 7.26 ppm for ^{1}H NMR and δ = 77.16 for ^{13}C NMR). Further NMR spectra were referenced to $CFCl_3$ (^{19}F). Coupling constants (*J*) are reported in Hertz (Hz). The multiplicity of the signals is given as s (singlet), d (doublet), t (triplet), q (quartet), sext (sextet), m (multiplet) and br (broad). When ambiguous, proton and carbon assignments were established using APT, COSY, NOESY and HMQC experiments.

Thin layer chromatography (TLC) was performed to monitor the reactions using precoated aluminium sheets of *Merck* silica gel 60 F254 (0.20 m), and detection of compounds was performed under UV light (254 nm) or dipping into a solution of $KMnO_4$ (1.5 g in 200 mL H_2O, 5 g $NaHCO_3$).

Flash column chromatography was performed using *Merck* silica gel 60 (40-63 µm) to purify products applying nitrogen pressure of about 0.2 bar or on a *Biotage Isolera Four* using *Biotage* cartridges *SNAP Ultra 10 g*, *SNAP Ultra 25g*, *SNAP Ultra 50g* and *SNAP Ultra 100g*. The solvents were used as laboratory grade.

High resolution mass spectrometry (HRMS) analyses were performed by the EPSRC Mass Spectrometry Facility in Swansea University on a *Waters Xevo G2-S* and on a *Thermo Scientific LTQ Orbitrap XL* or at Cardiff University on a *Waters LCT Premier XE*. Ions were generated by Electrospray Ionisation (ESI), Nanospray Ionisation (NSI), Atmospheric Solids Analysis Probe (ASAP), Chemical Ionisation (CI), Atmospheric Pressure Chemical Ionisation (APCI) or Electron Ionisation (EI).

Melting points (m.p.) were determined with a *Gallenkamp* variable heater and are not corrected.

Infrared spectra (IR) were recorded on a *Shimadzu FTIR Affinity-1S* spectrometer and the position of the absorption bands is given in wave numbers ν (cm^{-1}).

High-performance liquid chromatography (HPLC) analyses were performed on an *Agilent Technologies 1260 Infinity Quaternary LC* system. 2-Propanol and *n*-hexane were obtained as HPLC grade. Separation was performed using Lux® 5 µm Cellulose-1 or Amylose-1, LC Column (250 x 4.6 mm) or YMC Chiral Amylose-C S-5µm (25 cm).

Optical rotations were measured with a *SCHMIDT + HAENSCH UniPol L* polarimeter at 20 °C in a cuvette of 50 mm length with a sodium lamp (589 nm).

Cyclovoltammetric studies were performed using an Orygalys OGF500 Potentiostat / Galvanostat with OGFPWR power supply.

2 General Procedures

<u>General Procedure **GP1** for the synthesis of acetyl enol ethers</u>

A solution of *n*-BuLi (1.32 mL, 3.30 mmol, 1.1 equiv., 2.5 M in *n*-hexane) was added slowly at −78 °C to a solution of diisopropylamine (0.47 mL, 3.3 mmol, 1.1 equiv.) in dry THF (6 mL) in a flame-dried *Schlenk* tube under nitrogen atmosphere. The solution was stirred for 30 min at −78 °C and the propiophenone derivative (3.0 mmol) was added. Afterwards, the reaction mixture was stirred for additional 45 min and acetic anhydride (0.57 mL, 6.0 mmol, 2.0 equiv.) was added. The mixture was stirred for 30 min at −78 °C, warmed up to room temperature and stirred for another hour. The resulting suspension was subsequently quenched with saturated aqueous $NaHCO_3$ solution (30 mL) and the resulting mixture was extracted with EtOAc (3 x 30 mL). The combined organic layers were washed with brine (50 mL), dried over anhydrous $MgSO_4$ and concentrated under vacuum. The crude mixture was purified by flash column chromatography.

<u>General Procedure **GP2** for the synthesis of 2-iodoresorcinol derivatives</u>

The resorcinol derivative (20.0 mmol) and iodine (5.33 g, 21.0 mmol, 1.05 equiv.) were dissolved in water (30 mL) and THF (10 mL) and the solution was cooled to 0 °C. $NaHCO_3$ (1.85 g, 22.0 mmol, 1.1 equiv.) was added portionwise and the evolution of gas was observed. The reaction mixture was stirred for 10 min at 0 °C and 30 min at room temperature.

Afterwards, saturated aqueous $Na_2S_2O_3$ (50 mL) was added and the reaction mixture was extracted with EtOAc (3 x 50 mL). The combined organic layers were washed with brine (50 mL), dried over anhydrous $MgSO_4$ and concentrated under vacuum. The crude mixture was purified by recrystallisation or flash column chromatography.

General Procedure **GP3** for the *Mitsunobu* reaction of the 2-iodoresorcinol derivatives

The 2-iodoresorcinol derivative (5.00 mmol), methyl (*S*)-(–)-lactate (1.19 mL, 12.5 mmol, 2.5 equiv.) and triphenylphosphine (3.28 g, 12.5 mmol, 2.5 equiv.) were dissolved in dry THF (25 mL) under nitrogen. The reaction mixture was cooled to 0 °C and diisopropyl azodicarboxylate (2.46 mL, 12.5 mmol, 2.5 equiv.) was added dropwise. After 1 h at 0 °C, the mixture was warmed up to room temperature and stirred for 16 h. The solvent was removed under reduced pressure and Et_2O (40 mL) was added. Triphenylphosphine oxide, which precipitated, was removed by filtration and the filtrate was concentrated under vacuum. The crude mixture was purified by flash column chromatography.

General Procedure **GP4** for the basic hydrolysis of diesters

The iodoarene (3.00 mmol) was dissolved in THF (12 mL) and MeOH (12 mL). After cooling the solution to 0 °C, a 2 M aqueous solution of NaOH (12 mL, 24 mmol, 8.0 equiv.) was added slowly and the resulting solution was stirred at room temperature for 16 h. The reaction mixture was then acidified with 3 M aqueous HCl at 0 °C and extracted with EtOAc (3 x 15 mL). The combined organic layers were washed with brine (30 mL), dried over anhydrous $MgSO_4$ and concentrated under vacuum to afford the pure product.

General Procedure **GP5** for the synthesis of lactamides

The dicarboxylic acid (1.5 mmol) was suspended in dry CH_2Cl_2 (15 mL) in a flame-dried *Schlenk* tube under nitrogen atmosphere. After the addition of a catalytic amount of DMF (1 drop) and oxalyl chloride (450 µL, 5.25 mmol, 3.5 equiv.) at 0 °C, the reaction mixture was warmed up to room temperature, stirred for 3 h and then concentrated under reduced pressure. Afterwards, the crude product was redissolved in dry CH_2Cl_2 (7 mL) under nitrogen atmosphere and the solution was cooled to 0 °C. Subsequently, the desired amine (6.0 mmol, 4.0 equiv.) and dry pyridine (484 µL, 6.0 mmol, 4.0 equiv.) were added, the reaction solution was warmed to room temperature and stirred for 16 h. The reaction was then quenched with 3 M aqueous HCl and the resulting mixture extracted with CH_2Cl_2 (3 x 10 mL). The combined organic layers

were dried over anhydrous $MgSO_4$, concentrated under vacuum and the crude mixture was purified by flash column chromatography.

General Procedure **GP6** for the oxidation of iodoarenes with Selectfluor®

The iodoarene (0.400 mmol) was dissolved in CH_3CN (12 mL) and glacial acetic acid (4 mL) under nitrogen atmosphere. Selectfluor® (709 mg, 2.00 mmol, 5.0 equiv.) was added subsequently and the resulting suspension was stirred at room temperature for 5 h. After completion of the reaction, the solvents were removed under vacuum and the product was dissolved in $CHCl_3$. After filtration under nitrogen atmosphere, the filtrate was concentrated under reduced pressure and the residue was washed with Et_2O/*n*-hexane (3:1) to afford the pure product.

General Procedure **GP7** for the catalytic asymmetric *α*-acetoxylation

Iodoarene **7b** (10.7 mg, 0.0150 mmol, 5.0 mol%) and acetyl enol ether **4** (0.30 mmol) were dissolved in CH_2Cl_2 (1.12 mL) and AcOH (0.38 mL) under nitrogen atmosphere. After the addition of $BF_3 \cdot OEt_2$ (11 µL, 0.090 mmol, 30 mol%) and *m*CPBA (81 mg, 0.36 mmol, 1.2 equiv., 77% purity), the reaction mixture was stirred for 2 h at room temperature. Subsequently, saturated aqueous $Na_2S_2O_3$ (5 mL) was added and the resulting mixture was extracted with CH_2Cl_2 (3 x 10 mL) The combined organic layers were washed with saturated aqueous $NaHCO_3$ (30 mL), dried over anhydrous $MgSO_4$, concentrated under vacuum and the crude mixture was purified by flash column chromatography.

3 Optimisation of the Reaction Conditions

Table S1: Synthesis of *α*-acetoxy ketone (*R*)-**5a** using hypervalent iodine reagent **6d**.[a]

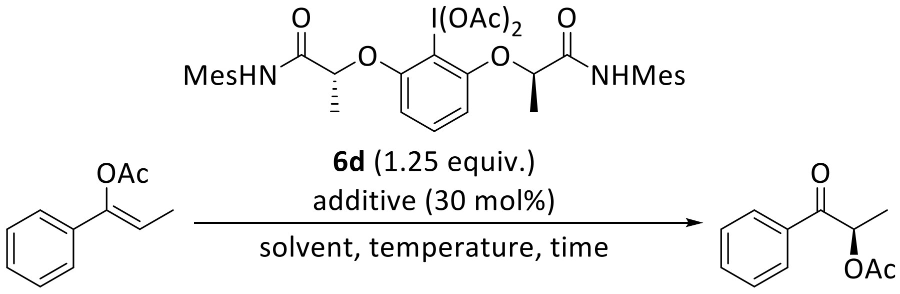

4a (*R*)-**5a**

Entry	Solvent	Additive	T [°C]	t [h]	Yield **5a** [%][b]	*ee* **5a** [%][c]
1	CH_2Cl_2	$BF_3{\cdot}OEt_2$	r.t.	14	69 (21)	85
2	MeCN	$BF_3{\cdot}OEt_2$	r.t.	14	33 (55)	61
3	CH_2Cl_2/TFE (9:1)	$BF_3{\cdot}OEt_2$	r.t.	14	41 (12)	83
4	HOAc	$BF_3{\cdot}OEt_2$	r.t.	14	48	43
5	CH_2Cl_2	$BF_3{\cdot}OEt_2$	−40	22	0	–
6	CH_2Cl_2	$BF_3{\cdot}OEt_2$	−78 to r.t.	14	10 (75)	80
7	CH_2Cl_2	$BF_3{\cdot}OEt_2$	−15	14	31 (41)	83
8	CH_2Cl_2	HOTf	−78	22	33 (36)	88

[a] All reactions were carried out with 0.30 mmol of **4a** and 0.38 mmol of **6d** in various solvents (1.5 mL). [b] Yield of recovered starting material in parentheses. [c] Enantiomeric excess was determined *via* chiral HPLC analysis.

4 Synthesis of Enol Ethers

(*Z*)-1-Phenyl-1-triethylsiloxy-1-propene (3)

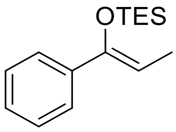

Propiophenone (0.40 mL, 3.0 mmol) was reacted with triethylsilyl chloride (0.55 mL, 3.3 mmol, 1.1 equiv.) instead of acetic anhydride according to **GP1** to give **3** as a colourless liquid in 96% yield (0.72 g, 2.9 mmol) after flash column chromatography (*n*-hexane).

^{1}**H NMR** (500 MHz, $CDCl_3$): *δ* = 7.47 – 7.41 (m, 2H, CH_{arom}), 7.32 – 7.20 (m, 3H, CH_{arom}), 5.21 (q, *J* = 6.3 Hz, 1H, C*H*), 1.75 (d, *J* = 6.5 Hz, 3H, CH_3), 0.93 (t, *J* = 7.8 Hz, 9H, CH_3), 0.61 (q, *J* = 7.6 Hz, 6H, CH_2) ppm. The spectroscopic data are in agreement with the literature.[1]

(*Z*)-1-Phenylprop-1-en-1-yl acetate (4a)

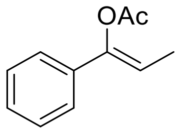

Propiophenone (1.33 mL, 10.0 mmol) was reacted according to **GP1** with *n*-BuLi (4.40 mL, 11.0 mmol, 1.1 equiv., 2.5 M in *n*-hexane), diisopropylamine (1.55 mL, 11.0 mmol, 1.1 equiv.) and acetic anhydride (1.89 mL, 20.0 mmol, 2.0 equiv.) to give **4a** as a colourless liquid in 82% yield (1.45 g, 8.20 mmol) after flash column chromatography (*n*-hexane:EtOAc = 95:5).

^{1}**H NMR** (500 MHz, $CDCl_3$): *δ* = 7.43 – 7.37 (m, 2H, CH_{arom}), 7.35 – 7.30 (m, 2H, CH_{arom}), 7.30 – 7.27 (m, 1H, CH_{arom}), 5.91 (q, *J* = 7.0 Hz, 1H, C*H*), 2.31 (s, 3H, CH_3), 1.72 (d, *J* = 7.0 Hz, 3H, CH_3) ppm. ^{13}**C NMR** (126 MHz, $CDCl_3$): *δ* = 168.7 (C), 147.05 (C), 135.1 (C), 128.6 (2 x CH), 128.1 (CH), 124.4 (2 x CH), 112.7 (CH), 20.7 (CH_3), 11.7 (CH_3) ppm. The spectroscopic data are in agreement with the literature.[2]

(*Z*)-1-(4-Fluorophenyl)prop-1-en-1-yl acetate (4b)

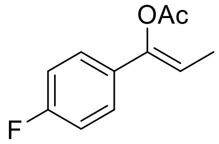

4'-Fluoropropiophenone (0.42 mL, 3.0 mmol) was reacted according to **GP1** to give **4b** as a colourless oil in 76% yield (441 mg, 2.27 mmol) after flash column chromatography (*n*-hexane:EtOAc = 95:5).

^{1}**H NMR** (500 MHz, $CDCl_3$): *δ* = 7.39 – 7.33 (m, 2H, CH_{arom}), 7.03 – 6.97 (m, 2H, CH_{arom}), 5.81 (q, *J* = 7.0 Hz, 1H, C*H*), 2.29 (s, 3H, CH_3), 1.70 (d, *J* = 7.0 Hz, 3H, CH_3) ppm. ^{13}**C NMR** (126 MHz, $CDCl_3$): *δ* = 168.7 (C), 162.7 (d, *J* = 247.5 Hz, C), 146.3 (C), 131.5 (d, *J* = 3.3 Hz, C), 126.3 (d, *J* = 8.1 Hz, 2 x CH), 115.6 (d, *J* = 21.8 Hz, 2 x CH), 112.7 (d, *J* = 1.6 Hz, CH), 20.7 (CH_3), 11.7 (CH_3) ppm. ^{19}**F NMR** (471 MHz, $CDCl_3$): *δ* = −113.83 – −113.94 (m, 1F, C*F*) ppm. **HRMS** (EI): *m/z* = 194.0743 calcd. for $C_{11}H_{11}O_2F^+$ $[M]^+$, found: 194.0743. **IR** (neat): *ν* = 1755*s*, 1607*w*, 1508*s*, 1369*w*, 1202*s*, 1159*m*, 1030*m*, 843*w*, 812*w*, 606*w* cm^{-1}.

(*Z*)-1-(3-Fluorophenyl)prop-1-en-1-yl acetate (4c)

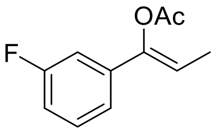

3'-Fluoropropiophenone (457 mg, 3.00 mmol) was reacted according to **GP1** to give **4c** as a colourless oil in 53% yield (309 mg, 1.59 mmol) after flash column chromatography (*n*-hexane:EtOAc = 95:5).

^{1}H NMR (500 MHz, $CDCl_3$): δ = 7.28 (m, 1H, CH_{arom}), 7.18 (ddd, J = 8.0, 1.7, 1.0 Hz, 1H, CH_{arom}), 7.10 – 7.04 (dd, J = 10.3, 1.8 Hz, 1H, CH_{arom}), 6.96 (tdd, J = 8.3, 2.6, 1.0 Hz, 1H, CH_{arom}), 5.93 (q, J = 7.0 Hz, 1H, CH), 2.31 (s, 3H, CH_3), 1.72 (d, J = 7.0 Hz, 3H, CH_3) ppm. **^{13}C NMR** (126 MHz, $CDCl_3$): δ = 168.6 (C), 163.1 (d, J = 245.2 Hz, CF), 146.1 (d, J = 2.9 Hz, C), 137.5 (d, J = 7.8 Hz, C), 130.2 (d, J = 8.4 Hz, CH), 120.0 (d, J = 2.8 Hz, CH), 115.0 (d, J = 21.3 Hz, CH), 114.2 (CH), 111.4 (d, J = 23.3 Hz, CH), 20.7 (CH_3), 11.8 (CH_3) ppm. **^{19}F NMR** (471 MHz, $CDCl_3$): δ = –113.0 – –113.1 (m, 1F, CF) ppm. **HRMS** (EI): m/z = 194.0743 calcd. for $C_{11}H_{11}O_2F^+$ $[M]^+$, found: 194.0741. **IR** (neat): ν = 1755*s*, 1614*w*, 1584*m*, 1489*w*, 1435*w*, 1369*m*, 1202*m*, 1179*m*, 1157*m*, 1030*m*, 910*w*, 864*m*, 785*m*, 764*w*, 687*w*, 665*w*, 642*w* cm^{-1}.

(*Z*)-1-(4-Bromophenyl)prop-1-en-1-yl acetate (4d)

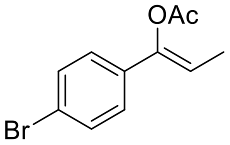

4'-Bromopropiophenone (639 mg, 3.00 mmol) was dissolved in dry DMSO (12 mL) in a flame-dried *Schlenk* tube under nitrogen atmosphere. After the solution was cooled to *ca*. 10 °C, NaH (0.18 g, 4.5 mmol, 1.5 equiv., 60% dispersion in mineral oil) was added portionwise over 30 min. Afterwards, the resulting suspension was warmed to room temperature and stirred for another 30 min. Acetic anhydride (0.78 mL, 7.2 mmol, 2.4 equiv.) was then added and the reaction mixture was stirred for 30 min.

The suspension was then quenched with water (30 mL) and the resulting mixture was extracted with *n*-hexane (3 x 30 mL). The combined organic layers were washed with brine (50 mL), dried over anhydrous $MgSO_4$ and concentrated under vacuum. The crude mixture was purified by flash column chromatography (*n*-hexane:EtOAc = 95:5) to give **4d** as a colourless solid in 60% (458 mg, 1.80 mmol) yield.

M.p.: 53 – 56 °C. **^{1}H NMR** (500 MHz, $CDCl_3$): δ = 7.46 – 7.42 (m, 2H, CH_{arom}), 7.27 – 7.23 (m, 2H, CH_{arom}), 5.89 (q, J = 7.0 Hz, 1H, C*H*), 2.29 (s, 3H, CH_3), 1.70 (d, J = 7.0 Hz, 3H, CH_3) ppm. **^{13}C NMR** (126 MHz, $CDCl_3$): δ = 168.6 (C), 146.2 (C), 134.2 (C), 131.7 (2 x CH), 126.0 (2 x CH), 122.1 (C), 113.6 (CH), 20.7 (CH_3), 11.8 (CH_3) ppm. **HRMS** (EI): m/z = 253.9942 calcd. for $C_{11}H_{11}O_2Br^+$ $[M]^+$, found: 253.9938. **IR** (neat): ν = 1753*s*, 1665*w*, 1584*w*, 1485*m*, 1396*w*, 1366*m*, 1202*s*, 1179*m*, 1113*w*, 1072*m*, 1030*m*, 1001*m*, 968*w*, 893*w*, 849*m*, 829*m*, 812*m*, 787*m*, 691*w*, 644*w* cm^{-1}.

(*Z*)-1-(3-(Trifluoromethyl)phenyl)prop-1-en-1-yl acetate (4e)

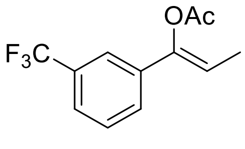

3′-(Trifluoromethy)propiophenone (505 µL, 3.00 mmol) was reacted according to **GP1** to give **4e** as a colourless oil in 56% yield (407 mg, 1.67 mmol) after flash column chromatography (*n*-hexane:EtOAc = 98:2).

^{1}H NMR (500 MHz, $CDCl_3$): δ = 7.63 (s, 1H, CH_{arom}), 7.56 (d, J = 7.8 Hz, 1H, CH_{arom}), 7.52 (d, J = 7.8 Hz, 1H, CH_{arom}), 7.44 (t, J = 7.8 Hz, 1H, CH_{arom}), 5.98 (q, J = 7.0 Hz, 1H, C*H*), 2.32 (s, 3H, CH_3), 1.74 (d, J = 7.0 Hz, 3H, CH_3) ppm. **^{13}C NMR** (126 MHz, $CDCl_3$): δ = 168.6 (C), 145.9 (C), 136.1 (C), 131.1 (q, J = 32.3 Hz, C), 129.2 (CH), 127.6 (d, J = 1.1 Hz, CH), 124.8 (q, J = 3.7 Hz, CH), 124.1 (q, J = 272.4 Hz, CF_3), 121.2 (q, J = 3.9 Hz, CH), 114.8 (CH), 20.7 (CH_3), 11.8 (CH_3) ppm. **^{19}F NMR** (471 MHz, $CDCl_3$): δ = −62.8 (s, 3F, CF_3) ppm. The spectroscopic data are in agreement with the literature.[3]

1-(2-(Trifluoromethyl)phenyl)prop-1-en-1-yl acetate (4f)

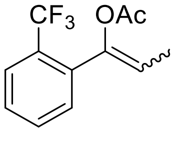

2′-(Trifluoromethy)propiophenone (500 µL, 3.00 mmol) was reacted according to **GP1** to give **4f** as a colourless oil in 61% yield (448 mg, 1.84 mmol) after flash column chromatography (*n*-hexane:EtOAc = 98:2) as a mixture of (*Z*)- and (*E*)-isomers (*Z*/*E* = 2.7:1, determined *via* ^{1}H NMR analysis).

^{1}H NMR (500 MHz, $CDCl_3$): δ = 7.72 – 7.68 (m, 1H, *Z*-CH_{arom}), 7.66 – 7.53 (m, 4H, *Z*-CH_{arom} and *E*-CH_{arom}), 7.51 (m, 1H, *E*-CH_{arom}), 7.49 – 7.44 (m, 1H, *Z*-CH_{arom}), 7.42 (m, 1H, *E*-CH_{arom}), 5.66 (q,

J = 7.2 Hz, 1H, *Z*-C*H*), 5.49 (q, J = 6.9 Hz, 1H, *E*-C*H*), 2.14 (s, 3H, *E*-CH_3), 2.07 (s, 3H, *Z*-CH_3), 1.73 (d, J = 6.9 Hz, 3H, CH_3), 1.55 (d, J = 7.2 Hz, 3H, CH_3) ppm. **^{13}C NMR** (126 MHz, $CDCl_3$): δ = 169.8 (C), 168.9 (C), 145.7 (C), 144.4 (C), 135.3 (q, J = 2.1 Hz, C), 133.6 (CH), 132.4 (q, J = 2.1 Hz, C), 132.1 (CH), 131.8 (d, J = 0.9 Hz, CH), 131.6 (d, J = 0.9 Hz, CH), 129.3 (d, J = 30.8 Hz, C), 129.0 (CH), 128.6 (CH), 127.9 (q, J = 30.9 Hz, C), 126.4 (q, J = 5.3 Hz, CH), 126.3 (q, J = 5.6 Hz, CH), 124.1 (q, J = 273.6 Hz, CF_3), 123.9 (q, J = 273.6 Hz, CF_3), 117.5 (CH), 117.2 (CH), 21.1 (CH_3), 20.8 (CH_3), 12.7 (CH_3), 11.6 (CH_3) ppm. **^{19}F NMR** (471 MHz, $CDCl_3$): δ = −59.8 (s, 3F, *E*-CF_3), −60.6 (s, 3F, *Z*-CF_3) ppm. The spectroscopic data are in agreement with the literature.[3]

(*Z*)-1-(3-Nitrophenyl)prop-1-en-1-yl acetate (4g)

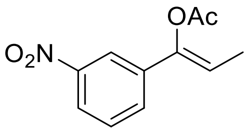

3′-Nitropropiophenone (538 µL, 3.00 mmol) was reacted according to **GP1** to give **4g** as a yellow oil in 43% yield (285 mg, 1.29 mmol) after flash column chromatography (*n*-hexane:EtOAc = 100:0 → 95:5).

^{1}H NMR (500 MHz, $CDCl_3$): δ = 8.23 (t, J = 2.0 Hz, 1H, CH_{arom}), 8.12 (ddd, J = 8.1, 2.1, 0.9 Hz, 1H, CH_{arom}), 7.70 (ddd, J = 8.0, 1.8, 1.0 Hz, 1H, CH_{arom}), 7.50 (t, J = 8.0 Hz, 1H, CH_{arom}), 6.05 (q, J = 7.0 Hz, 1H, C*H*), 2.34 (s, 3H, CH_3), 1.77 (d, J = 7.0 Hz, 3H, CH_3) ppm. **^{13}C NMR** (126 MHz, $CDCl_3$): δ = 168.6 (C), 148.7 (C), 145.1 (C), 137.1 (C), 130.1 (CH), 129.7 (CH), 122.8 (CH), 119.4 (CH), 116.0 (CH), 20.7 (CH_3), 11.9 (CH_3) ppm. **HRMS** (EI): *m/z* = 221.0688 calcd. for $C_{11}H_{11}NO_2^+$ [M]$^+$, found: 221.0692. **IR** (neat): ν = 1755*s*, 1526*s*, 1433*w*, 1346*m*, 1192*s*, 1123*w*, 1032*m*, 899*w*, 791*w*, 737*s*, 704*w*, 637*w* cm^{-1}.

(*Z*)-1-(*p*-Tolyl)prop-1-en-1-yl acetate (4h)

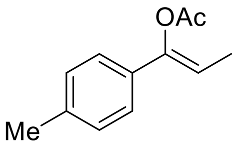

4′-Methylpropiophenone (448 µL, 3.00 mmol) was reacted according to **GP1** to give **4h** as a colourless oil in 88% yield (502 mg, 2.64 mmol) after flash column chromatography (*n*-hexane:EtOAc = 95:5).

^{1}H NMR (500 MHz, $CDCl_3$): δ = 7.30 – 7.26 (m, 2H, CH_{arom}), 7.15 – 7.10 (m, 2H, CH_{arom}), 5.84 (q, J = 7.0 Hz, 1H, C*H*), 2.33 (s, 3H, CH_3), 2.30 (s, 3H, CH_3), 1.71 (d, J = 7.0 Hz, 3H, CH_3) ppm. **^{13}C NMR** (126 MHz, $CDCl_3$): δ = 168.8 (C), 147.1 (C), 138.0 (C), 132.4 (C), 129.3 (2 x CH), 124.3 (2 x CH), 111.8 (CH), 21.3 (CH_3), 20.8 (CH_3), 11.6 (CH_3) ppm. The spectroscopic data are in agreement with the literature.[3]

(*Z*)-1-(4-(*tert*-Butyl)phenyl)prop-1-en-1-yl acetate (4i)

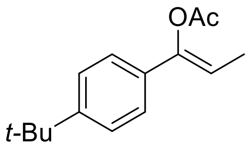

4'-*tert*-Butylpropiophenone (595 µL, 3.00 mmol) was reacted according to **GP1** to give **4i** as a colourless oil in 50% yield (350 mg, 1.51 mmol) after flash column chromatography (*n*-hexane:EtOAc = 95:5).

^{1}H NMR (500 MHz, $CDCl_3$): δ = 7.35 – 7.31 (m, 4H, CH_{arom}), 5.86 (q, J = 7.0 Hz, 1H, C*H*), 2.30 (s, 3H, CH_3), 1.71 (d, J = 7.0 Hz, 3H, CH_3), 1.30 (s, 9H, CH_3) ppm. **^{13}C NMR** (126 MHz, $CDCl_3$): δ = 168.8 (C), 151.2 (C), 147.1 (C), 132.3 (C), 125.6 (2 x CH), 124.1 (2 x CH), 111.9 (CH), 34.7 (C), 31.4 (3 x CH_3), 20.8 (CH_3), 11.7 (CH_3) ppm. **HRMS** (ESI): *m/z* = 255.1361 calcd. for $C_{15}H_{20}O_2Na^+$ [M+Na]$^+$, found: 255.1366. **IR** (neat): ν = 2961*w*, 1757*s*, 1510*w*, 1366*m*, 1275*w*, 1209*m*, 1188*m*, 1030*w*, 1015*w*, 791*w*, 731*w* cm^{-1}.

(*Z*)-1-([1,1'-Biphenyl]-4-yl)prop-1-en-1-yl acetate (4j)

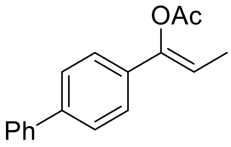

4'-Phenylpropiophenone (631 mg, 3.00 mmol) was reacted according to **GP1** to give **4j** as a colourless solid in 83% yield (632 mg, 2.50 mmol) after recrystallisation from EtOAc/*n*-hexane. **M.p.**: 144 – 146 °C. **^{1}H NMR** (500 MHz, $CDCl_3$): δ = 7.61 – 7.54 (m, 4H, CH_{arom}), 7.49 – 7.41 (m, 4H, CH_{arom}), 7.38 – 7.32 (m, 1H, CH_{arom}), 5.96 (q, J = 7.0 Hz, 1H, C*H*), 2.34 (s, 3H, CH_3), 1.75 (d, J = 7.0 Hz, 3H, CH_3) ppm. **^{13}C NMR** (126 MHz, $CDCl_3$): δ = 168.8 (C), 146.8 (C), 140.9 (C), 140.7 (C), 134.1 (C), 128.9 (2 x CH), 127.5 (CH), 127.3 (2 x CH), 127.1 (2 x CH), 124.8 (2 x CH), 112.9 (CH), 20.8 (CH_3), 11.8 (CH_3) ppm. **HRMS** (ESI): *m/z* = 275.1048 calcd. for $C_{17}H_{16}O_2Na^+$ [M+Na]$^+$,

found: 275.1046. **IR** (neat): ν = 1749*s*, 1487*w*, 1375*m*, 1219*m*, 1196*w*, 1031*w*, 901*w*, 841*w*, 762*s*, 737*w*, 692*m*, 660*w* cm^{-1}.

(*Z*)-1-(4-Methoxyphenyl)prop-1-en-1-yl acetate (4k)

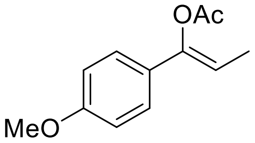

4'-Methoxypropiophenone (526 µL, 3.00 mmol) was reacted according to **GP1** to give **4k** as a colourless solid in 37% yield (228 mg, 1.10 mmol) after flash column chromatography (*n*-hexane:EtOAc = 97:3).

^{1}H NMR (500 MHz, $CDCl_3$): δ = 7.35 – 7.29 (m, 2H, CH_{arom}), 6.88 – 6.82 (m, 2H, CH_{arom}), 5.76 (q, *J* = 7.0 Hz, 1H, C*H*), 3.80 (s, 3H, CH_3), 2.29 (s, 3H CH_3), 1.69 (d, *J* = 7.0 Hz, 3H, CH_3) ppm. **^{13}C NMR** (126 MHz, $CDCl_3$): δ = 168.8 (C), 159.6 (C), 146.9 (C), 127.9 (C), 125.8 (2 x CH), 114.0 (2 x CH), 110.8 (CH), 55.4 (CH_3), 20.8 (CH_3), 11.6 (CH_3) ppm. The spectroscopic data are in agreement with the literature.[3]

(*Z*)-1,2-Diphenylvinyl acetate (4l)

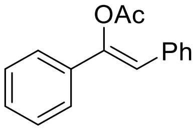

2-Phenylacetophenone (589 mg, 3.00 mmol) was reacted according to **GP1** to give **4l** as a colourless solid in 48% yield (345 mg, 1.45 mmol) after flash column chromatography (*n*-hexane:EtOAc = 95:5).

^{1}H NMR (500 MHz, $CDCl_3$): δ = 7.56 – 7.50 (m, 4H, CH_{arom}), 7.42 – 7.31 (m, 5H, CH_{arom}), 7.30 – 7.25 (m, 1H, CH_{arom}), 6.71 (s, 1H, C*H*), 2.32 (s, 3H, CH_3) ppm. **^{13}C NMR** (126 MHz, $CDCl_3$): δ = 168.7 (C), 146.7 (C), 135.7 (C), 134.5 (C), 128.8 (5 x CH), 128.7 (2 x CH), 127.8 (CH), 124.9 (2 x CH), 116.9 (CH), 21.2 (CH_3) ppm. The spectroscopic data are in agreement with the literature.[4]

(*Z*)-3-Methyl-1-phenylbut-1-en-1-yl acetate (4m)

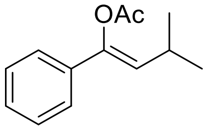

Isobutyl phenyl ketone (504 µL, 3.00 mmol) was reacted according to **GP1** to give **4m** as a colourless oil in 75% yield (458 mg, 2.24 mmol) after flash column chromatography (*n*-hexane:EtOAc = 97:3).

^{1}H NMR (500 MHz, $CDCl_3$): δ = 7.41 – 7.38 (m, 2H, CH_{arom}), 7.34 – 7.30 (m, 2H, CH_{arom}), 7.29 – 7.24 (m, 1H, CH_{arom}), 5.65 (d, *J* = 9.6 Hz, 1H, C*H*), 2.61 (dhept, *J* = 9.6, 6.7 Hz, 1H, C*H*), 2.29 (s, 3H, CH_3), 1.07 (d, *J* = 6.7 Hz, 6H, CH_3) ppm. **^{13}C NMR** (126 MHz, $CDCl_3$): δ = 169.1 (C), 144.5 (C), 135.2 (C), 128.6 (2 x CH), 128.2 (CH), 125.3 (CH), 124.5 (2 x CH), 26.4 (CH), 22.7 (2 x CH_3), 20.8 (CH_3) ppm. The spectroscopic data are in agreement with the literature.[5]

(*Z*)-1-Phenylbut-1-en-1-yl acetate (4n)

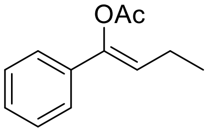

Butyrophenone (435 µL, 3.00 mmol) was reacted according to **GP1** to give **4n** as a colourless oil in 88% yield (500 mg, 2.62 mmol) after flash column chromatography (*n*-hexane:EtOAc = 95:5).

^{1}H NMR (500 MHz, $CDCl_3$): δ = 7.42 – 7.38 (m, 2H, CH_{arom}), 7.35 – 7.30 (m, 2H, CH_{arom}), 7.29 – 7.25 (m, 1H, CH_{arom}), 5.82 (t, *J* = 7.5 Hz, 1H, C*H*), 2.30 (s, 3H, CH_3), 2.15 (pent, *J* = 7.5 Hz, 2H, CH_2), 1.08 (t, *J* = 7.5 Hz, 3H, CH_3) ppm. **^{13}C NMR** (126 MHz, $CDCl_3$): δ = 168.9 (C), 145.7 (C), 135.2 (C), 128.6 (2 x CH), 128.2 (CH), 124.5 (2 x CH), 119.9 (CH), 20.8 (CH_3), 19.8 (CH_2), 13.6 (CH_3) ppm. The spectroscopic data are in agreement with the literature.[3]

3,4-Dihydronaphthalen-1-yl acetate (4o)

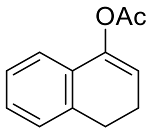

1-Tetralone (400 µL, 3.00 mmol) was reacted according to **GP1** to give **4o** as a colourless solid in 97% yield (550 mg, 2.92 mmol) after flash column chromatography (*n*-hexane:EtOAc = 100:0 → 90:10).

^{1}H NMR (500 MHz, $CDCl_3$): *δ* = 7.22 – 7.16 (m, 3H, CH_{arom}), 7.14 – 7.10 (m, 1H, CH_{arom}), 5.74 (t, *J* = 4.7 Hz, 1H, C*H*), 2.90 (t, *J* = 8.1 Hz, 2H, CH_2), 2.48 (td, *J* = 8.1, 4.7 Hz, 2H, CH_2), 2.33 (s, 3H, CH_3) ppm. **^{13}C NMR** (126 MHz, $CDCl_3$): *δ* = 169.4 (C), 145.7 (C), 136.5 (C), 130.5 (C), 128.0 (CH), 127.7 (CH), 126.5 (CH), 120.8 (CH), 115.6 (CH), 27.6 (CH_2), 22.1 (CH_2), 21.0 (CH_3) ppm. The spectroscopic data are in agreement with the literature.[6]

(*Z*)-1-(Thiophen-2-yl)prop-1-en-1-yl acetate (4p)

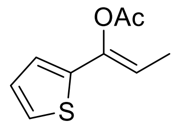

2-Propionylthiophene (374 µL, 3.00 mmol) was reacted according to **GP1**to give **4p** as a colourless oil in 58% yield (318 mg, 1.74 mmol) after flash column chromatography (*n*-hexane:EtOAc = 100:0 → 96:4).

^{1}H NMR (500 MHz, $CDCl_3$): *δ* = 7.16 (dd, *J* = 5.0, 1.2 Hz, 1H, CH_{arom}), 6.97 (dd, *J* = 3.6, 1.2 Hz, 1H, CH_{arom}), 6.94 (dd, *J* = 5.0, 3.6 Hz, 1H, CH_{arom}), 5.81 (q, *J* = 7.1 Hz, 1H, C*H*), 2.29 (s, 3H, CH_3), 1.68 (d, *J* = 7.1 Hz, 3H, CH_3) ppm. **^{13}C NMR** (126 MHz, $CDCl_3$): *δ* = 168.4 (C), 142.3 (C), 139.2 (C), 127.5 (CH), 124.6 (CH), 123.1 (CH), 112.5 (CH), 20.7 (CH_3), 11.6 (CH_3) ppm. The spectroscopic data are in agreement with the literature.[3]

2-Cyano-1-phenylvinyl acetate (4q)

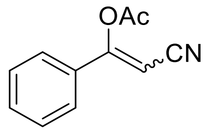

Benzoylacetonitrile (435 mg, 3.00 mmol) was reacted according to **GP1** to give **4q** as a colourless oil in 80% yield (449 mg, 2.40 mmol) after flash column chromatography (*n*-hexane:EtOAc = 90:10) as a mixture of (*Z*)- and (*E*)-isomers (*Z*/*E* = 1.4:1, determined *via* ^{1}H and NOESY NMR analysis).

^{1}H NMR (500 MHz, $CDCl_3$): *δ* = 7.78 – 7.70 (m, 2H, *E*-CH_{arom}), 7.55 – 7.38 (m, 8H, *Z*-CH_{arom} and *E*-CH_{arom}), 5.70 (s, 1H, Z-C*H*), 5.51 (s, 1H, *E*-C*H*), 2.41 (s, 3H, *Z*-CH_3), 2.29 (s, 3H, *E*-CH_3) ppm. **^{13}C NMR** (101 MHz, $CDCl_3$): *δ* = 167.7 (C), 167.2 (C), 164.7 (C), 164.3 (C), 132.0 (CH), 131.79 (CH), 131.75 (C), 131.7 (C), 129.2 (2 x CH), 129.0 (2 x CH), 127.5 (2 x CH), 125.7 (2 x CH), 116.1 (C), 114.7 (C), 88.4 (CH, *E*), 87.4 (CH, *Z*), 21.2 (CH_3, *E*), 20.8 (CH_3, *Z*) ppm. **HRMS** (EI):

m/z = 187.0633 calcd. for $C_{11}H_9INO_2^+$ $[M]^+$, found: 187.0637. **IR** (neat): ν = 2220*m*, 1771*s*, 1626*m*, 1371*w*, 1167*s*, 1067*m*, 1009*w*, 887*w*, 756*m*, 689*m* cm^{-1}.

5 Synthesis of Hypervalent Iodine Reagents

2-Iodoresorcinol (S1a)

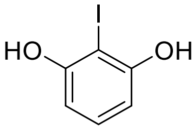

Resorcinol (5.00 g, 45.4 mmol), iodine (11.9 g, 47.7 mmol, 1.05 equiv.) and $NaHCO_3$ (4.19 g, 49.9 mmol, 1.1 equiv.) were reacted according to **GP2**. The crude product was crystallised from $CHCl_3$ to afford **S1a** as colourless crystals in 64% yield (6.86 g, 29.1 mmol).

^{1}H NMR (500 MHz, Acetone-d$_6$): δ = 8.78 (s, 2H, O*H*), 7.00 (t, J = 8.0 Hz, 1H, CH_{arom}), 6.46 (d, J = 8.0 Hz, 2H, CH_{arom}) ppm. **^{13}C NMR** (126 MHz, Acetone-d$_6$): δ = 158.9 (2 x C), 130.4 (CH), 107.1 (2 x CH), 75.3 (C) ppm. The spectroscopic data are in agreement with the literature.[7]

2-Iodo-5-methylbenzene-1,3-diol (S1b)

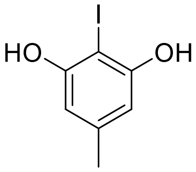

5-Methylresorcinol (2.48 g, 20.0 mmol) was reacted according to **GP2** to give **S1b** as a colourless solid in 73% yield (3.65 g, 14.6 mmol) after flash column chromatography (*n*-hexane:EtOAc = 95:5).

^{1}H NMR (400 MHz, $CDCl_3$): δ = 6.40 (q, J = 0.6 Hz, 2H, CH_{arom}), 5.27 (s, 2H, O*H*), 2.25 (t, J = 0.6 Hz, 3H, CH_3) ppm. **^{13}C NMR** (126 MHz, $CDCl_3$): δ = 155.4 (2 x C), 141.3 (C), 108.4 (2 x CH), 73.7 (C), 21.3 (CH_3) ppm. The spectroscopic data are in agreement with the literature.[8]

5-Bromo-2-iodobenzene-1,3-diol (S1c)

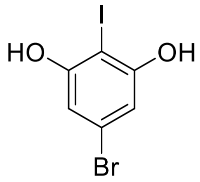

5-Bromoresorcinol (3.78 g, 20.0 mmol) was reacted according to **GP2** to give **S1c** as a colourless solid in 42% yield (2.65 g, 8.40 mmol) after flash column chromatography (*n*-hexane:EtOAc = 90:10).

^{1}H NMR (500 MHz, $CDCl_3$): δ = 6.74 (s, 2H, CH_{arom}), 5.38 (s, 2H, O*H*) ppm. **^{13}C NMR** (126 MHz, $CDCl_3$): δ = 156.2 (2 x C), 123.8 (C), 111.0 (2 x CH), 76.4 (C) ppm. The spectroscopic data are in agreement with the literature.[9]

1-(3,5-Dihydroxy-4-iodophenyl)ethan-1-one (S1d)

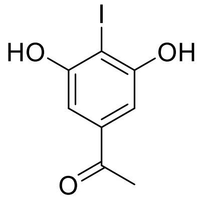

3',5'-Dihydroxyacetophenone (3.04 g, 20.0 mmol) was reacted according to **GP2** to give **S1d** as a pale yellow solid in 48% yield (2.66 g, 9.57 mmol) after flash column chromatography (*n*-hexane:EtOAc = 80:20).

^{1}H NMR (400 MHz, $CDCl_3$, spiked with MeOD): δ = 6.89 (s, 2H, CH_{arom}), 2.92 (br, 2H, O*H*), 2.49 (s, 3H, CH_3) ppm. **^{13}C NMR** (126 MHz, $CDCl_3$, spiked with MeOD): δ = 199.1 (C), 157.6 (2 x C), 138.3 (C), 105.6 (2 x CH), 82.9 (C), 26.6 (CH_3) ppm. The spectroscopic data are in agreement with the literature.[10]

Dimethyl 2,2'-((2-iodo-1,3-phenylene)bis(oxy))(2*R*,2'*R*)-dipropionate (S2a)

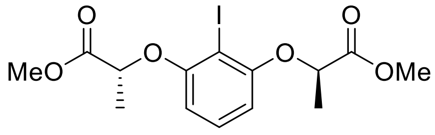

2-Iodoresorcinol (2.36 g, 10.0 mmol) was reacted with methyl (*S*)-(−)-lactate (2.4 mL, 25 mmol, 2.5 equiv.), triphenylphosphine (6.6 g, 25 mmol, 2.5 equiv.) and diisopropyl azodicarboxylate (2.4 mL, 25 mmol, 2.5 equiv.) in THF (50 mL) according to **GP3** to give **S2a** as

a colourless solid in 88% yield (3.58 g, 8.76 mmol) after flash column chromatography (*n*-hexane:EtOAc = 95:5 → 85:15).

^{1}H NMR (500 MHz, $CDCl_3$): δ = 7.13 (t, J = 8.3 Hz, 1H, CH_{arom}), 6.36 (d, J = 8.3 Hz, 2H, CH_{arom}), 4.76 (q, J = 6.8 Hz, 2H, C*H*), 3.74 (s, 6H, CH_3), 1.70 (d, J = 6.8 Hz, 6H, CH_3) ppm. **^{13}C NMR** (126 MHz, $CDCl_3$): δ = 172.3 (2 x C), 158.4 (2 x C), 129.7 (CH), 107.1 (2 x CH), 80.8 (C), 74.4 (2 x CH), 52.5 (2 x CH_3), 18.8 (2 x CH_3) ppm. The spectroscopic data are in agreement with the literature.[11]

Dimethyl 2,2'-((2-iodo-1,3-phenylene)bis(oxy))(2*R*,2'*R*)-bis(3-phenylpropanoate) (S2b)

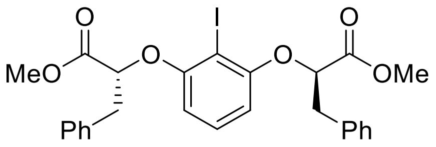

2-Iodoresorcinol (708 mg, 3.00 mmol) was reacted with methyl L-3-phenyllactate (1.35 g, 7.50 mmol, 2.5 equiv.), triphenylphosphine (1.98 g, 7.50 mmol, 2.5 equiv.) and diisopropyl azodicarboxylate (1.57 mL, 7.5 mmol, 2.5 equiv.) in THF (15 mL) according to **GP3** to give **S2b** as a colourless solid in 48% yield (807 mg, 1.44 mmol) after flash column chromatography (*n*-hexane:EtOAc = 90:10).

M.p.: 82 – 86 °C. **^{1}H NMR** (500 MHz, $CDCl_3$): δ = 7.45 – 7.41 (m, 4H, CH_{arom}), 7.33 – 7.29 (m, 4H, CH_{arom}), 7.27 – 7.23 (m, 2H, CH_{arom}), 7.05 (t, J = 8.3 Hz, 1H, CH_{arom}), 6.22 (d, J = 8.3 Hz, 2H, CH_{arom}), 4.82 (dd, J = 8.0, 4.4 Hz, 2H, C*H*), 3.67 (s, 6H, CH_3), 3.36 (dd, J = 14.0, 8.0 Hz, 2H, CH_2), 3.29 (dd, J = 14.0, 4.4 Hz, 2H, CH_2) ppm. **^{13}C NMR** (126 MHz, $CDCl_3$): δ = 171.1 (2 x C), 158.2 (2 x C), 136.1 (2 x C), 130.0 (4 x CH), 129.6 (CH), 128.5 (4 x CH), 127.2 (2 x CH), 106.0 (2 x CH), 79.5 (C), 78.9 (2 x CH), 52.4 (2 x CH_3), 39.2 (2 x CH_2) ppm. **HRMS** (ESI): *m/z* = 561.0774 calcd. for $C_{26}H_{26}O_6I^+$ [M+H]$^+$, found: 561.0784. **IR** (neat): ν = 1736*m*, 1566*m*, 1587*m*, 1458*s*,1437*w*, 1292*w*, 1246*w*, 1198*w*, 1175*w*, 1101*s*, 1020*w*, 907*s*, 725*s*, 698*s*, 646*w* cm^{-1}. **$[\alpha]_D^{20}$** = +65.0 (c = 0.40, $CHCl_3$).

Dimethyl 2,2'-((2-iodo-5-methyl-1,3-phenylene)bis(oxy))(2*R*,2'*R*)-dipropionate (S2c)

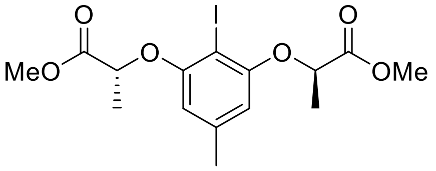

2-Iodo-5-methylbenzene-1,3-diol (**S1b**, 1.25 g, 5.00 mmol) was reacted according to **GP3** to give **S2c** as a colourless solid in 77% yield (1.63 g, 3.85 mmol) after flash column chromatography (*n*-hexane:EtOAc = 95:5 → 85:15).

^{1}H NMR (400 MHz, $CDCl_3$): δ = 6.19 (s, 2H, CH_{arom}), 4.74 (q, J = 6.8 Hz, 2H, C*H*), 3.75 (s, 6H, CH_3), 2.25 (s, 3H, CH_3), 1.68 (d, J = 6.8 Hz, 6H, CH_3) ppm. **^{13}C NMR** (126 MHz, $CDCl_3$): δ = 172.4 (2 x C), 158.1 (2 x C), 140.3 (C), 108.3 (2 x CH), 76.9 (C), 74.4 (2 x CH), 52.5 (2 x CH_3), 22.0 (CH_3), 18.8 (2 x CH_3) ppm. The spectroscopic data are in agreement with the literature.[8]

Dimethyl 2,2'-((5-bromo-2-iodo-1,3-phenylene)bis(oxy))(2*R*,2'*R*)-dipropionate (S2d)

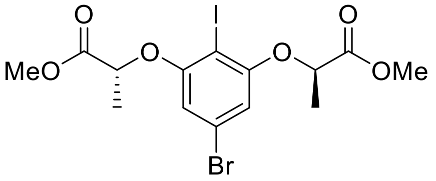

5-Bromo-2-iodobenzene-1,3-diol (**S1c**, 1.57 g, 5.00 mmol) was reacted according to **GP3** to give **S2d** as a colourless solid in 68% yield (1.66 g, 3.41 mmol) after flash column chromatography (*n*-hexane:EtOAc = 90:10).

M.p.: 96 – 100 °C. **^{1}H NMR** (400 MHz, $CDCl_3$): δ = 6.50 (s, 2H, CH_{arom}), 4.74 (q, J = 6.8 Hz, 2H, C*H*), 3.77 (s, 6H, CH_3), 1.69 (d, J = 6.8 Hz, 6H, CH_3) ppm. **^{13}C NMR** (126 MHz, $CDCl_3$): δ = 171.7 (2 x C), 158.7 (2 x C), 123.0 (C), 110.7 (2 x CH), 79.5 (C), 74.5 (2 x CH), 52.6 (2 x CH_3), 18.7 (2 x CH_3) ppm. **HRMS** (ESI): m/z = 508.9073 calcd. for $C_{14}H_{16}IO_6BrNa^+$ $[M+Na]^+$, found: 508.9081. **IR** (neat): ν = 1736*s*, 1574*m*, 1557*m*, 1447*w*, 1408*m*, 1375*w*, 1277*w*, 1206*s*, 1130*s*, 1103*m*, 1018*m*, 972*m*, 831*m*, 810*m*, 640*m* cm^{-1}. **$[\alpha]_D^{20}$** = +15.0 (c = 0.4, $CHCl_3$).

Dimethyl 2,2'-((5-acetyl-2-iodo-1,3-phenylene)bis(oxy))(2*R*,2'*R*)-dipropionate (S2e)

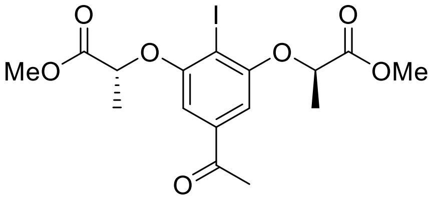

1-(3,5-Dihydroxy-4-iodophenyl)ethan-1-one (**S1d**, 1.39 g, 5.00 mmol) was reacted according to **GP3** to give **S2e** as a colourless solid in 56% yield (1.27 g, 2.81 mmol) after flash column chromatography (*n*-hexane:EtOAc = 80:20).

M.p.: 68 – 70 °C. **^{1}H NMR** (500 MHz, $CDCl_3$): δ = 6.95 (s, 2H, C*H*$_{arom}$), 4.87 (q, *J* = 6.8 Hz, 2H, C*H*), 3.76 (s, 6H, CH_3), 2.51 (s, 3H, CH_3), 1.72 (d, *J* = 6.8 Hz, 6H, CH_3) ppm. **^{13}C NMR** (126 MHz, $CDCl_3$): δ = 196.5 (C), 171.9 (2 x C), 158.5 (2 x C), 138.7 (C), 106.5 (2 x CH), 88.1 (C), 74.4 (2 x CH), 52.6 (2 x CH_3), 26.6 (CH_3), 18.6 (2 x CH_3) ppm. **HRMS** (ESI): *m/z* = 473.0073 calcd. for $C_{16}H_{19}IO_7Na^+$ $[M+Na]^+$, found: 473.0073. **IR** (neat): ν = 1751*s*, 1684*m*, 1570*m*, 1447*w*, 1414*m*, 1358*w*, 1314*w*, 1279*w*, 1211*s*, 1134*s*, 1109*m*, 1020*m*, 974*w*, 847*w* cm^{-1}. **$[\alpha]_D^{20}$** = +20.3 (c = 0.4, $CHCl_3$).

(2*R*,2'*R*)-2,2'-((2-Iodo-1,3-phenylene)bis(oxy))dipropionic acid (S3a)

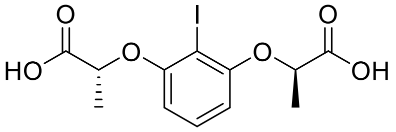

Iodoarene **S2a** (1.22 g, 3.00 mmol) was reacted according to **GP4** to give **S3a** as a colourless solid in 99% yield (1.13 g, 2.98 mmol).

^{1}H NMR (500 MHz, MeOD): δ = 7.17 (t, *J* = 8.2 Hz, 1H, C*H*$_{arom}$), 6.44 (d, *J* = 8.2 Hz, 2H, C*H*$_{arom}$), 4.80 (q, *J* = 6.8 Hz, 2H, C*H*), 1.65 (d, *J* = 6.8 Hz, 6H, CH_3) ppm. **^{13}C NMR** (126 MHz, MeOD): δ = 175.2 (2 x C), 159.7 (2 x C), 130.6 (CH), 107.7 (2 x CH), 80.6 (C), 75.0 (2 x CH), 19.0 (2 x CH_3) ppm. The spectroscopic data are in agreement with the literature.[12]

(2*R*,2'*R*)-2,2'-((2-Iodo-1,3-phenylene)bis(oxy))bis(3-phenylpropanoic acid) (S3b)

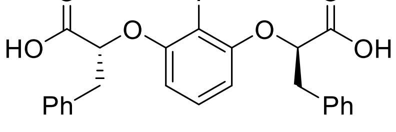

Iodoarene **S2b** (560 mg, 1.00 mmol) was reacted according to **GP4** with a 2 M aqueous solution of NaOH (4 mL) in THF (4 mL) and MeOH (4 mL) to give **S3b** as a colourless solid in 98% (523 mg, 0.98 mmol) yield.

M.p.: 154 – 157 °C. **^{1}H NMR** (500 MHz, Acetone-d$_6$): δ = 11.45 (s, 2H, O*H*), 7.53 (d, *J* = 7.5 Hz, 4H, C*H*$_{arom}$), 7.29 (t, *J* = 7.5 Hz, 4H, C*H*$_{arom}$), 7.22 (t, *J* = 7.5 Hz, 2H, C*H*$_{arom}$), 7.16 (t, *J* = 8.3 Hz, 1H, C*H*$_{arom}$), 6.43 (d, *J* = 8.3 Hz, 2H, C*H*$_{arom}$), 4.99 (dd, *J* = 7.1, 4.9 Hz, 2H, C*H*), 3.42 – 3.28 (m, 4H, C*H*$_2$) ppm. **^{13}C NMR** (126 MHz, Acetone-d$_6$): δ = 171.5 (2 x C), 159.1 (2 x C), 137.6 (2 x C), 130.9 (4 x CH), 130.4 (CH), 129.0 (4 x CH), 127.6 (2 x CH), 106.5 (2 x CH), 79.2 (C), 78.9 (2 x CH), 39.5 (2 x CH$_2$) ppm. **HRMS** (ESI): *m/z* = 531.0305 calcd. for $C_{24}H_{20}IO_6^-$ [M−H]$^-$, found: 531.0314. **IR** (neat): ν = 2980*br*, 1701*s*, 1583*m*, 1497*w*, 1458*s*, 1246*m*, 1192*m*, 1103*s*, 1022*w*, 750*w*, 698*s* cm^{-1}. **[α]$_D^{20}$** = +70.0 (c = 0.4, MeOH).

(2*R*,2'*R*)-2,2'-((2-Iodo-5-methyl-1,3-phenylene)bis(oxy))dipropionic acid (S3c)

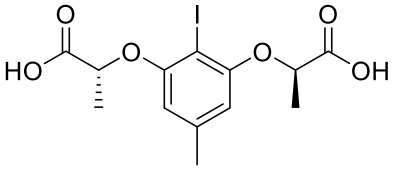

Iodoarene **S2c** (1.27 g, 3.00 mmol) was reacted according to **GP4** to give **S3c** as a colourless solid in > 99% (1.18 g, 3.00 mmol) yield.

^{1}H NMR (400 MHz, CDCl$_3$): δ = 11.27 (br, 2H, O*H*), 6.27 (s, 2H, C*H*$_{arom}$), 4.81 (q, *J* = 6.9 Hz, 2H, C*H*), 2.29 (s, 3H, C*H*$_3$), 1.73 (d, *J* = 6.9 Hz, 6H, C*H*$_3$) ppm. **^{13}C NMR** (126 MHz, CDCl$_3$): δ = 176.3 (2 x C), 157.5 (2 x C), 140.8 (C), 108.6 (2 x CH), 76.9 (C), 73.8 (2 x CH), 22.0 (CH$_3$), 18.5 (2 x CH$_3$) ppm. The spectroscopic data are in agreement with the literature.[8]

(2*R*,2'*R*)-2,2'-((5-Bromo-2-iodo-1,3-phenylene)bis(oxy))dipropionic acid (S3d)

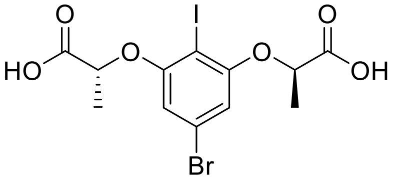

Iodoarene **S2d** (1.46 g, 3.00 mmol) was reacted according to **GP4** to give **S3d** as a colourless solid in > 99% (1.37 g, 3.00 mmol) yield.

M.p.: 180 – 183 °C. **^{1}H NMR** (400 MHz, Acetone-d$_6$): δ = 11.50 (br, 2H, O*H*), 6.70 (s, 2H, C*H*$_{arom}$), 5.01 (q, *J* = 6.8 Hz, 2H, C*H*), 1.68 (d, *J* = 6.8 Hz, 6H, C*H*$_3$) ppm. **^{13}C NMR** (126 MHz, Acetone-d$_6$): δ = 172.4 (2 x C), 159.8 (2 x C), 123.3 (C), 110.5 (2 x CH), 79.2 (C), 74.4 (2 x CH), 18.7 (2 x CH$_3$) ppm. **HRMS** (ESI): *m/z* = 480.8760 calcd. for $C_{12}H_{12}IO_6BrNa^+$ [M+Na]$^+$, found: 480.8761. **IR**

(neat): ν = 3512*br*, 1732*m*, 1692*s*, 1576*m*, 1557*m*, 1449*w*, 1410*m*, 1248*m*, 1229*m*, 1134*s*, 1107*s*, 1018*m*, 810*w* cm^{-1}. **[α]$_D$^{20}** = +8.6 (c = 0.4, MeOH).

(2*R*,2'*R*)-2,2'-((5-Acetyl-2-iodo-1,3-phenylene)bis(oxy))dipropionic acid (S3e)

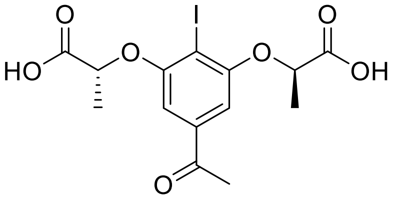

Iodoarene **S2e** (1.35 g, 3.00 mmol) was reacted according to **GP4** to give **S3e** as a pale yellow solid in > 99% (1.26 g, 3.00 mmol) yield.

M.p.: 150 – 152 °C. **^{1}H NMR** (400 MHz, Acetone-d$_6$): δ = 11.41 (s, 2H, O*H*), 7.10 (s, 2H, C*H*$_{arom}$), 5.08 (q, *J* = 6.8 Hz, 2H, C*H*), 2.53 (s, 3H, C*H*$_3$), 1.70 (d, *J* = 6.8 Hz, 6H, C*H*$_3$) ppm. **^{13}C NMR** (126 MHz, Acetone-d$_6$): δ = 196.8 (C), 172.6 (2 x C), 159.4 (2 x C), 139.5 (C), 106.3 (2 x CH), 86.8 (C), 74.3 (2 x CH), 26.64 (CH$_3$), 18.7 (2 x CH$_3$) ppm. **HRMS** (ESI): *m/z* = 422.9941 calcd. for $C_{14}H_{16}IO_7^+$ [M+H]$^+$, found: 422.9947. **IR** (neat): ν = 3522*br*, 1744*m*, 1676*m*, 1568*s*, 1540*w*, 1414*s*, 1362*w*, 1314*m*, 1223*s*, 1134*s*, 1105*m*, 1020*s*, 826*w* cm^{-1}. **[α]$_D$^{20}** = +6.9 (c = 0.4, MeOH).

(2*R*,2'*R*)-2,2'-((2-Iodo-1,3-phenylene)bis(oxy))bis(*N*-methylpropanamide) (S4a)

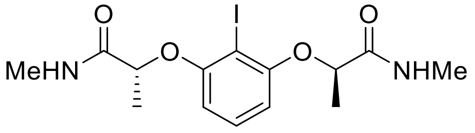

Iodoarene **S2a** (245 mg, 0.600 mmol) was dissolved in a solution of methylamine in ethanol (3.8 mL, 30 mmol, 50 equiv., 8 M) in a flame-dried *Schlenk* tube under nitrogen atmosphere. The solution was stirred at room temperature for 20 h and a colourless solid was formed. Afterwards, all volatiles were removed under vacuum and the solid was washed with Et_2O to afford **S4a** as a colourless solid in 96% yield (234 mg, 0.576 mmol).

M.p.: 162 – 166 °C. **^{1}H NMR** (500 MHz, CDCl$_3$): δ = 7.25 (t, 1H, *J* = 8.3 Hz, C*H*$_{arom}$), 6.82 (br, 2H, N*H*), 6.48 (d, *J* = 8.3 Hz, 2H, C*H*$_{arom}$), 4.77 (q, *J* = 6.7 Hz, 2H, C*H*), 2.88 (d, *J* = 4.9 Hz, 6H, C*H*$_3$), 1.63 (d, *J* = 6.7 Hz, 6H, C*H*$_3$) ppm. **^{13}C NMR** (126 MHz, CDCl$_3$): δ = 171.9 (2 x C), 157.2 (2 x C), 130.7 (CH), 107.1 (2 x CH), 80.6 (C), 76.3 (2 x CH), 26.1 (2 x CH$_3$), 18.6 (2 x CH$_3$) ppm. **HRMS** (ESI): *m/z* = 407.0468 calcd. for $C_{14}H_{20}N_2O_4I^+$ [M+H]$^+$, found: 407.0449. **IR** (neat): ν = 3414*w*,

3306*br*, 1655*s*, 1584*w*, 1535*m*, 1456*s*, 1410*w*, 1371*w*, 1244*s*, 1092*s*, 1020*w*, 920*w*, 766*m*, 729*m* cm^{-1}. **[α]$_D^{20}$** = –154.2 (c = 0.4, $CHCl_3$).

Dimesityl 2,2'-((2-iodo-1,3-phenylene)bis(oxy))(2*R*,2'*R*)-dipropionate (S4b)

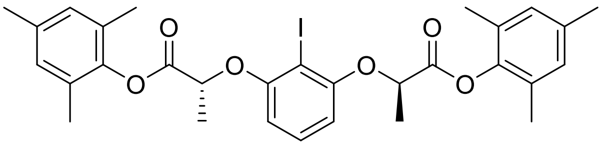

Dicarboxylic acid **S3a** (456 mg, 1.2 mmol), mesitol (409 mg, 3.00 mmol, 2.5 equiv.), dry triethylamine (333 µL, 2.4 mmol, 2.0 equiv.) and 4-DMAP (36 mg, 0.30 mmol, 0.25 equiv.) were dissolved in dry CH_2Cl_2 (15 mL) in a flame-dried *Schlenk* tube under nitrogen atmosphere and EDC·HCl (576 mg, 3.00 mmol, 2.5 equiv.) was added. After the solution was stirred at room temperature for 22 h, 3 M aqueous HCl was added and the resulting mixture was extracted with CH_2Cl_2 (3 x 10 mL), dried over anhydrous $MgSO_4$ and concentrated under vacuum. The crude mixture was purified by flash column chromatography (*n*-hexane:EtOAc = 90:10) to afford **S4b** as a colourless solid in 55% yield (407 mg, 0.660 mmol).

^{1}H NMR (500 MHz, $CDCl_3$): δ = 7.22 (t, *J* = 8.2 Hz, 1H, CH_{arom}), 6.85 (s, 4H, CH_{arom}), 6.63 (d, *J* = 8.2 Hz, 2H, CH_{arom}), 5.10 (q, *J* = 6.7 Hz, 2H, C*H*), 2.26 (s, 6H, CH_3), 2.03 (s, 12H, CH_3), 1.93 (d, *J* = 6.7 Hz, 6H, CH_3) ppm. **^{13}C NMR** (126 MHz, $CDCl_3$): δ = 169.7 (2 x C), 158.4 (2 x C), 145.5 (2 x C), 135.7 (2 x C), 129.7 (C), 129.6 (4 x C), 129.5 (4 x CH), 107.5 (2 x CH), 81.1 (C), 74.3 (2 x CH), 20.8 (2 x CH_3), 19.0 (2 x CH_3), 16.4 (4 x CH_3) ppm. The spectroscopic data are in agreement with the literature.[13]

(2*R*,2'*R*)-2,2'-((2-Iodo-1,3-phenylene)bis(oxy))bis(*N*-mesitylpropanamide) (S4c)

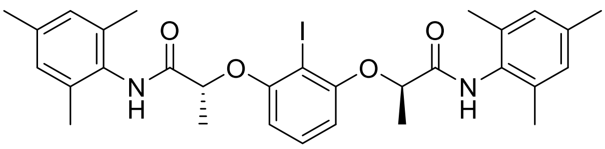

Dicarboxylic acid **S3a** (570 mg, 1.50 mmol) was reacted with 2,4,6-trimethylaniline (842 µL, 6.00 mmol, 4.0 equiv.) according to **GP5**to give **S4c** as a colourless solid in 72% yield (664 mg, 1.08 mmol) after flash column chromatography (*n*-hexane:EtOAc = 70:30 → 50:50), followed by trituration of the brown solid with Et_2O.

^{1}H NMR (500 MHz, $CDCl_3$): δ = 8.02 (s, 2H, N*H*), 7.35 (t, *J* = 8.3 Hz, 1H, CH_{arom}), 6.91 (s, 4H, CH_{arom}), 6.66 (d, *J* = 8.3 Hz, 2H, CH_{arom}), 5.02 (q, *J* = 6.7 Hz, 2H, C*H*), 2.28 (s, 6H, CH_3), 2.16 (s,

12H, CH_3), 1.79 (d, J = 6.7 Hz, 6H, CH_3) ppm. **^{13}C NMR** (126 MHz, $CDCl_3$): δ = 169.7 (2 x C), 157.1 (2 x C), 137.4 (2 x C), 135.2 (4 x C), 130.8 (CH), 130.2 (2 x C), 129.1 (4 x CH), 107.2 (2 x CH), 80.7 (C), 76.2 (2 x CH), 21.0 (2 x CH_3), 18.9 (2 x CH_3), 18.4 (4 x CH_3) ppm. The spectroscopic data are in agreement with the literature.[12]

(2*R*,2'*R*)-2,2'-((2-Iodo-1,3-phenylene)bis(oxy))bis(*N*-(2,6-diisopropylphenyl)propanamide) (7a)

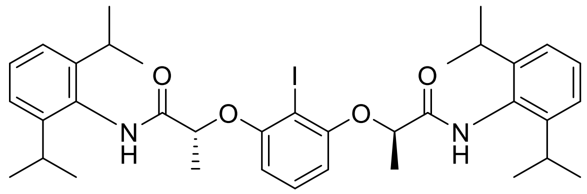

Dicarboxylic acid **S3a** (570 mg, 1.50 mmol) was reacted with 2,6-diisopropylaniline (1.14 mL, 6.00 mmol, 4.0 equiv.) according to **GP5** to give **7a** as a colourless solid in 79% yield (829 mg, 1.19 mmol) after flash column chromatography (*n*-hexane:EtOAc:CH_2Cl_2 = 80:10:10 → 60:20:20).

M.p.: 202 – 204 °C. **^{1}H NMR** (500 MHz, $CDCl_3$): δ = 7.97 (s, 2H, NH), 7.40 (t, J = 8.3 Hz, 1H, CH_{arom}), 7.31 (t, J = 7.7 Hz, 2H, CH_{arom}), 7.19 (d, J = 7.7 Hz, 4H, CH_{arom}), 6.71 (d, J = 8.3 Hz, 2H, CH_{arom}), 5.07 (q, J = 6.6 Hz, 2H, CH), 2.97 (br, 4H, CH), 1.80 (d, J = 6.6 Hz, 6H, CH_3), 1.20 (d, J = 6.8 Hz, 12H, CH_3), 1.13 (br, 12H, CH_3) ppm. **^{13}C NMR** (126 MHz, $CDCl_3$): δ = 170.7 (2 x C), 157.1 (2 x C), 146.3 (4 x C), 130.8 (CH), 130.2 (2 x C), 128.8 (2 x CH), 123.7 (4 x CH), 107.2 (2 x CH), 80.7 (C), 76.2 (2 x CH), 28.8 (4 x CH), 23.7 (8 x CH_3), 18.8 (2 x CH_3) ppm. **HRMS** (ESI): m/z = 699.2659 calcd. for $C_{36}H_{48}N_2O_4I^+$ [M+H]$^+$, found: 699.2660. **IR** (neat): ν = 3381*w*, 2962*w*, 1686*m*, 1585*w*, 1501*m*, 1458*m*, 1383*w*, 1331*w*, 1248*m*, 1132*w*, 1092*m*, 1059*m*, 907*s*, 797*m*, 727*s*, 646*m* cm^{-1}. **[α]$_D^{20}$** = −86.5 (c = 0.42, $CHCl_3$).

(2*R*,2'*R*)-2,2'-((2-Iodo-1,3-phenylene)bis(oxy))bis(*N*-(2,6-diisopropylphenyl)-3-phenyl-propanamide) (S4d)

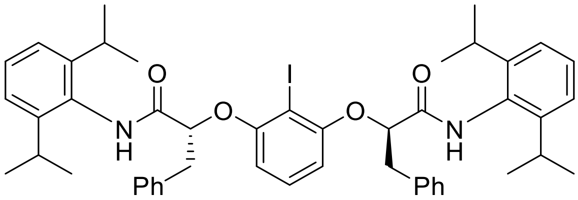

Dicarboxylic acid **S3b** (490 mg, 0.92 mmol) was reacted with oxalyl chloride (277 µL, 3.22 mmol, 3.5 equiv.), pyridine (298 µL, 3.68 mmol, 4.0 equiv.) and 2,6-diisopropylaniline (694 µL, 3.68 mmol, 4.0 equiv.) according to **GP5** to give **S4d** as a colourless foam in 75% yield (584 mg, 0.686 mmol) after flash column chromatography (*n*-hexane:EtOAc = 75:25 → 0:100). **^{1}H NMR** (400 MHz, $CDCl_3$): δ = 7.80 (s, 2H, N*H*), 7.40 (dd, *J* = 7.6, 1.5 Hz, 4H, CH_{arom}), 7.35 – 7.21 (m, 9H, CH_{arom}), 7.14 (d, *J* = 7.8 Hz, 4H, CH_{arom}), 6.62 (d, *J* = 8.4 Hz, 2H, CH_{arom}), 5.22 (dd, *J* = 6.8, 3.5 Hz, 2H, C*H*), 3.52 (dd, *J* = 14.3, 3.5 Hz, 2H, CH_2), 3.44 (dd, *J* = 14.3, 6.8 Hz, 2H, CH_2), 2.62 (hept, *J* = 6.8 Hz, 4H, C*H*) 1.07 (d, *J* = 6.8 Hz, 12H, CH_3), 1.05 – 0.96 (m, 12H, CH_3) ppm. **^{13}C NMR** (126 MHz, $CDCl_3$): δ = 169.1 (2 x C), 157.3 (2 x C), 146.2 (4 x C), 135.9 (2 x C), 130.7 (CH), 130.3 (4 x CH), 129.9 (2 x C), 128.7 (2 x CH), 128.5 (4 x CH), 127.2 (2 x CH), 123.6 (4 x CH), 106.8 (2 x CH), 80.5 (2 x CH), 79.4 (C), 38.2 (2 x CH_2), 28.6 (4 x CH), 23.9 (4 x CH_3), 23.7 (4 x CH_3) ppm. **HRMS** (ESI): *m/z* = 851.3285 calcd. for $C_{48}H_{56}IN_2O_4^+$ $[M+H]^+$, found: 851.3290. **IR** (neat): ν = 3387*w*, 1686*s*, 1585*m*, 1497*m*, 1454*s*, 1383*w*, 1240*m*, 1088*s*, 907*s*, 797*m*, 727*s*, 698*s*, 646*m* cm^{-1}. **$[\alpha]_D^{20}$** = +20.8 (c = 0.4, $CHCl_3$).

(2*R*,2'*R*)-2,2'-((2-Iodo-1,3-phenylene)bis(oxy))bis(*N*,*N*-diisopropylpropanamide) (S4e)

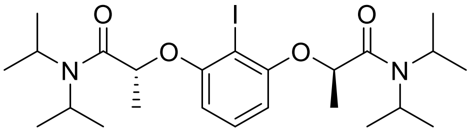

Dicarboxylic acid **S3a** (570 mg, 1.50 mmol) was reacted with diisopropylamine (841 µL, 6.00 mmol, 4.0 equiv.) according to **GP5** to give **S4e** as a colourless solid in 83% yield (682 mg, 1.25 mmol) after flash column chromatography (*n*-hexane:EtOAc = 80:20 → 60:40).

^{1}H NMR (400 MHz, $CDCl_3$): δ = 7.12 (t, *J* = 8.4 Hz, 1H, CH_{arom}), 6.52 (d, *J* = 8.4 Hz, 2H, CH_{arom}), 4.82 (q, *J* = 6.9 Hz, 2H, C*H*), 4.53 (hept, *J* = 6.7 Hz, 2H, C*H*), 3.29 (hept, *J* = 6.7 Hz, 2H, C*H*), 1.66 (d, *J* = 6.9 Hz, 6H, CH_3), 1.40 (d, *J* = 6.7 Hz, 6H, CH_3), 1.28 (d, *J* = 6.7 Hz, 6H, CH_3), 1.18 (d, *J* = 6.7 Hz, 6H, CH_3), 0.90 (d, *J* = 6.7 Hz, 6H, CH_3) ppm. **^{13}C NMR** (126 MHz, $CDCl_3$): δ = 169.6 (2 x

C), 157.9 (2 x C), 130.0 (CH), 106.1 (2 x CH), 78.7 (C), 78.0 (2 x CH), 47.7 (2 x CH), 46.5 (2 x CH), 21.0 (2 x CH_3), 20.7 (2 x CH_3), 20.7 (2 x CH_3), 20.0 (2 x CH_3), 18.1 (2 x CH_3) ppm. The spectroscopic data are in agreement with the literature.[14]

(2*R*,2'*R*)-2,2'-((2-Iodo-5-methyl-1,3-phenylene)bis(oxy))bis(*N*-(2,6-diisopropylphenyl)-propanamide) (7b)

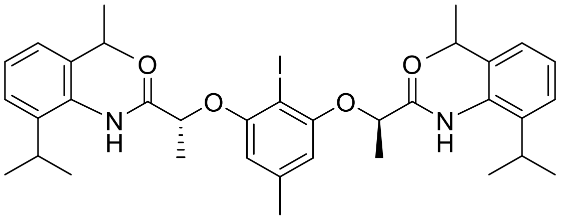

Dicarboxylic acid **S3c** (591 mg, 1.50 mmol) was reacted with 2,6-diisopropylaniline (1.14 mL, 6.00 mmol, 4.0 equiv.) according to **GP5** to give **7b** as a colourless solid in 82% yield (878 mg, 1.23 mmol) after flash column chromatography (*n*-hexane:EtOAc:CH_2Cl_2 = 90:5:5 → 30:35:35) followed by trituration of the yellow solid with Et_2O.

^{1}H NMR (500 MHz, $CDCl_3$): δ = 7.90 (s, 2H, N*H*), 7.31 (t, *J* = 7.7 Hz, 2H, CH_{arom}), 7.18 (d, *J* = 7.7 Hz, 4H, CH_{arom}), 6.54 (s, 2H, CH_{arom}), 5.03 (q, *J* = 6.7 Hz, 2H, C*H*), 2.96 (br, 4H, C*H*), 2.40 (s, 3H, CH_3), 1.80 (d, *J* = 6.7 Hz, 6H, CH_3), 1.20 (d, *J* = 6.9 Hz, 12H, CH_3), 1.12 (br, 12H, CH_3) ppm.
^{13}C NMR (126 MHz, $CDCl_3$): δ = 170.8 (2 x C), 156.9 (2 x C), 146.3 (4 x C), 141.7 (C), 130.1 (2 x C), 128.8 (2 x CH), 123.7 (4 x CH), 108.2 (2 x CH), 76.4 (C), 76.2 (2 x CH), 28.8 (4 x CH), 23.7 (8 x CH_3), 22.1 (CH_3), 18.9 (2 x CH_3) ppm. The spectroscopic data are in agreement with the literature.[8]

(2*R*,2'*R*)-2,2'-((5-Bromo-2-iodo-1,3-phenylene)bis(oxy))bis(*N*-(2,6-diisopropylphenyl)-propanamide) (7c)

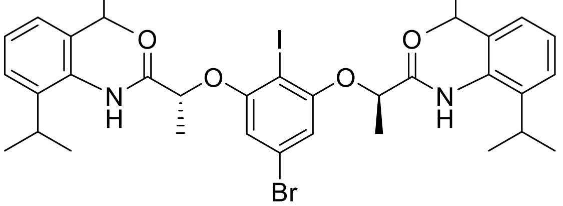

Dicarboxylic acid **S3d** (689 mg, 1.5 mmol) was reacted with 2,6-diisopropylaniline (1.14 mL, 6.00 mmol, 4.0 equiv.) according to **GP5** to give **7c** as a colourless solid in 99% yield (1.15 g, 1.48 mmol) after flash column chromatography (*n*-hexane:EtOAc:CH_2Cl_2 = 90:5:5 → 20:40:40).

M.p.: 208 – 210 °C. **^{1}H NMR** (500 MHz, $CDCl_3$): δ = 7.80 (s, 2H N*H*), 7.32 (t, *J* = 7.7 Hz, 2H, CH_{arom}), 7.19 (d, *J* = 7.7 Hz, 4H, CH_{arom}), 6.87 (s, 2H, CH_{arom}), 5.01 (q, *J* = 6.7 Hz, 2H, C*H*), 2.96 (br, 4H, C*H*), 1.81 (d, *J* = 6.7 Hz, 6H, CH_3), 1.22 (d, *J* = 6.9 Hz, 12H, CH_3), 1.13 (br, 12H, CH_3) ppm. **^{13}C NMR** (126 MHz, $CHCl_3$): δ = 170.1 (2 x C), 157.6 (2 x C), 146.3 (4 x C), 130.0 (2 x C), 128.9 (2 x CH), 124.4 (C), 123.7 (4 x CH), 111.0 (2 x CH), 79.0 (C), 76.7 (2 x CH), 28.9 (4 x CH), 23.7 (br, 8 x CH_3) 18.8 (2 x CH_3) ppm. **HRMS** (ESI): *m/z* = 777.1764 calcd. for $C_{36}H_{47}IN_2O_4Br^+$ [M+H]$^+$, found: 777.1766. **IR** (neat): ν = 3200*br*, 2961*m*, 1661*s*, 1574*w*, 1522*w*, 1458*w*, 1410*m*, 1379*w*, 1331*w*, 1221*m*, 1132*m*, 1099*m*, 1020*m*, 908*m*, 797*w*, 731*s* cm^{-1}. **[α]$_D^{20}$** = +8.7 (c = 0.4, $CHCl_3$).

(2*R*,2'*R*)-2,2'-((5-Acetyl-2-iodo-1,3-phenylene)bis(oxy))bis(*N*-(2,6-diisopropylphenyl)-propanamide) (7d)

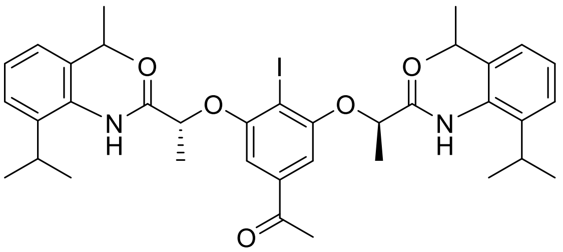

Dicarboxylic acid **S3e** (634 mg, 1.50 mmol) was reacted with 2,6-diisopropylaniline (1.14 mL, 6.00 mmol, 4.0 equiv.) according to **GP5** to give **7d** as a colourless solid in 78% yield (871 mg, 1.18 mmol) after flash column chromatography (*n*-hexane:EtOAc:CH_2Cl_2 = 90:5:5 → 50:25:25).

M.p.: 94 – 97 °C. **^{1}H NMR** (500 MHz, $CDCl_3$): δ = 7.91 (s, 2H, N*H*), 7.32 (t, *J* = 7.7 Hz, 2H, CH_{arom}), 7.19 (d, *J* = 7.7 Hz, 4H, CH_{arom}), 5.17 (q, *J* = 6.7 Hz, 2H, C*H*), 2.97 (br, 4H, C*H*), 2.62 (s, 3H, CH_3), 1.83 (d, *J* = 6.7 Hz, 6H, CH_3), 1.20 (d, *J* = 6.8 Hz, 12H, CH_3), 1.13 (br, 12H, CH_3) ppm. **^{13}C NMR** (126 MHz, $CDCl_3$): δ = 195.9 (C), 170.2 (2 x C), 157.4 (2 x C), 146.2 (4 x C), 139.8 (C), 130.0 (2 x C), 128.9 (2 x CH), 123.7 (4 x CH), 106.4 (2 x CH), 87.1 (C), 76.5 (2 x CH), 28.9 (4 x CH), 26.7 (CH_3), 23.8 (4 x CH_3), 23.7 (4 x CH_3), 18.7 (2 x CH_3) ppm. **HRMS** (ESI): *m/z* = 741.2764 calcd. for $C_{38}H_{50}IN_2O_6^+$ [M+H]$^+$, found: 741.2762. **IR** (neat): ν = 3379*w*, 2963*w*, 1684*s*, 1572*m*, 1501*m*, 1412*m*, 1362*w*, 1221*w*, 1107*m*, 1018*w*, 908*m*, 798*m*, 731*s*, 646*w* cm^{-1}. **[α]$_D^{20}$** = –25.0 (c = 0.4, $CHCl_3$).

Dimethyl 2,2'-((2-(diacetoxy-λ^3-iodaneyl)-1,3-phenylene)bis(oxy))(2*R*,2'*R*)-dipropionate (6a)

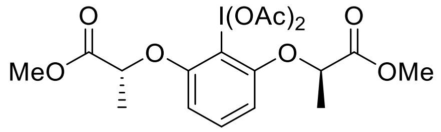

Iodoarene **S2a** (163 mg, 0.400 mmol) was reacted according to **GP6** to give **6a** as a pale yellow oil in 95% yield (200 mg, 0.380 mmol).

^{1}H NMR (500 MHz, $CDCl_3$): δ = 7.38 (t, J = 8.4 Hz, 1H, CH_{arom}), 6.56 (d, J = 8.4 Hz, 2H, CH_{arom}), 4.85 (q, J = 6.8 Hz, 2H, C*H*), 3.73 (s, 6H, CH_3), 1.94 (s, 6H, CH_3), 1.65 (d, J = 6.8 Hz, 6H, CH_3) ppm. **^{13}C NMR** (126 MHz, $CDCl_3$): δ = 177.0 (2 x C), 171.4 (2 x C), 156.7 (2 x C), 135.3 (CH), 106.9 (C), 106.3 (2 x CH), 74.5 (2 x CH), 52.6 (2 x CH), 20.5 (2 x CH_3), 18.4 (2 x CH_3) ppm. The spectroscopic data are in agreement with the literature.[11]

(2,6-Bis(((*R*)-1-(methylamino)-1-oxopropan-2-yl)oxy)phenyl)-λ^3-iodanediyl diacetate (6b)

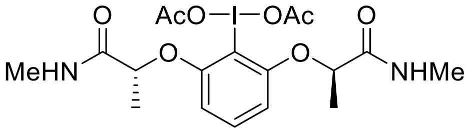

Iodoarene **S4a** (162 mg, 0.400 mmol) was reacted according to **GP6** to give **6b** as a colourless solid in 67% yield (141 mg, 0.268 mmol).

M.p.: 150 – 154 °C. **^{1}H NMR** (400 MHz, $CDCl_3$): δ = 7.46 (t, J = 8.4 Hz, 1H, CH_{arom}), 7.10 – 6.95 (m, 2H, N*H*), 6.65 (d, J = 8.4 Hz, 2H, CH_{arom}), 4.91 (q, J = 6.7 Hz, 2H, C*H*), 2.74 (d, J = 4.8 Hz, 6H, CH_3), 2.00 (s, 6H, CH_3), 1.67 (d, J = 6.7 Hz, 6H, CH_3) ppm. **^{13}C NMR** (126 MHz, $CDCl_3$): δ = 176.5 (2 x C), 171.5 (2 x C), 155.8 (2 x C), 136.4 (CH), 105.8 (2 x CH), 105.3 (C), 76.0 (2 x CH), 26.1 (2 x CH_3), 20.5 (2 x CH_3), 19.0 (2 x CH_3) ppm. **HRMS** (CI): *m/z* = 465.0517 calcd. for $C_{16}H_{22}IN_2O_6^+$ [M−OAc]$^+$, found: 465.0521. **IR** (neat): ν = 3356*br*, 1663*s*, 1545*m*, 1464*s*, 1412*w*, 1364*w*, 1248*s*, 1090*s*, 1005*w*, 914*m*, 772*m*, 727*s*, 665*m* cm^{-1}. **$[\alpha]_D^{20}$** = −50.0 (c = 0.4, $CHCl_3$).

Dimesityl 2,2'-((2-(diacetoxy-λ^3-iodaneyl)-1,3-phenylene)bis(oxy))(2*R*,2'*R*)-dipropionate (6c)

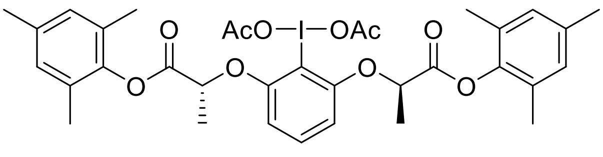

Iodoarene **S4b** (247 mg, 0.400 mmol) was reacted according to **GP6** to give **6c** as a colourless solid in 96% yield (283 mg, 0.385 mmol).

M.p.: 152 – 156 °C. **^{1}H NMR** (400 MHz, $CDCl_3$): *δ* = 7.48 (t, *J* = 8.4 Hz, 1H, CH_{arom}), 6.89 – 6.79 (m, 6H, CH_{arom}), 5.20 (q, *J* = 6.8 Hz, 2H, C*H*), 2.24 (s, 6H, CH_3), 1.96 (br, 12H, CH_3), 1.90 (d, *J* = 6.8 Hz, 6H, CH_3), 1.69 (s, 6H, CH_3) ppm. **^{13}C NMR** (126 MHz, $CDCl_3$): *δ* = 176.9 (2 x C), 168.9 (2 x C), 156.7 (2 x C), 145.2 (2 x C), 135.7 (2 x C), 135.1 (CH), 129.34 (4 x C), 129.31 (4 x CH), 107.8 (C), 106.5 (2 x CH), 74.5 (2 x CH), 20.6 (2 x CH_3), 19.9 (2 x CH_3), 18.6 (2 x CH_3), 16.1 (4 x CH_3) ppm. **HRMS** (CI): *m/z* = 692.1715 calcd. for $C_{32}H_{39}INO_8^+$ [M+NH_4–HOAc]$^+$, found: 692.1719. **IR** (neat): *ν* = 1755*m*, 1643*m*, 1589*w*, 1464*m*, 1364*m*, 1254*s*, 1190*m*, 1132*s*, 1098*s*, 1005*w*, 908*m*, 851*w*, 727*s*, 665*s* cm^{-1}. **[*α*]$_D^{20}$** = −101.3 (c = 0.4, $CHCl_3$).

(2,6-Bis(((*R*)-1-(mesitylamino)-1-oxopropan-2-yl)oxy)phenyl)-*λ*^{3}-iodanediyl diacetate (6d)

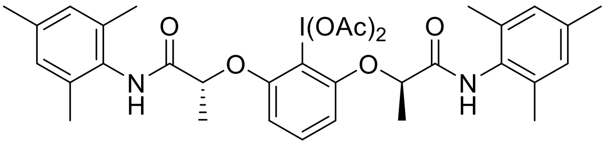

Iodoarene **S4c** (246 mg, 0.400 mmol) was reacted according to **GP6** to give **6d** as a yellow solid in 90% yield (265 mg, 0.361 mmol).

^{1}H NMR (500 MHz, $CDCl_3$): *δ* = 8.34 (s, 2H, N*H*), 7.54 (t, *J* = 8.4 Hz, 1H, CH_{arom}), 6.92 (d, *J* = 8.5 Hz, 2H, CH_{arom}), 6.78 (s, 4H, CH_{arom}), 5.15 (q, *J* = 6.7 Hz, 2H, C*H*), 2.20 (s, 6H, CH_3), 1.87 (d, *J* = 6.7 Hz, 6H, CH_3), 1.86 (br, 12H, CH_3), 1.48 (s, 6H, CH_3) ppm. **^{13}C NMR** (126 MHz, $CDCl_3$): *δ* = 176.8 (2 x C), 169.7 (2 x C), 156.0 (2 x C), 137.4 (2 x C), 136.6 (CH), 135.0 (2 x C), 129.7 (4 x C), 129.1 (4 x CH), 106.4 (2 x CH), 104.2 (C), 76.3 (2 x CH), 20.9 (2 x CH_3), 19.5 (2 x CH_3), 19.5 (2 x CH_3), 17.7 (4 x CH_3) ppm. The spectroscopic data are in agreement with the literature and ^{13}C spectral assignment was conducted based on reported HMQC and HMBC studies.[12]

(2,6-Bis(((*R*)-1-((2,6-diisopropylphenyl)amino)-1-oxopropan-2-yl)oxy)phenyl)-*λ*^{3}-iodanediyl diacetate (6e)

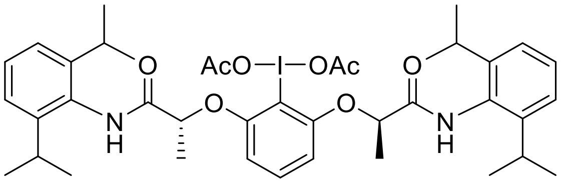

Iodoarene **7a** (280 mg, 0.400 mmol) was reacted according to **GP6** to give **6e** as a yellow solid in 78% yield (254 mg, 0.311 mmol).

M.p.: 172 – 174 °C. **^{1}H NMR** (400 MHz, $CDCl_3$): *δ* = 8.24 (s, 2H, N*H*), 7.66 (t, *J* = 8.4 Hz, 1H, CH_{arom}), 7.25 (t, *J* = 7.7 Hz, 2H, CH_{arom}), 7.10 (br, 4H, CH_{arom}), 6.98 (d, *J* = 8.5 Hz, 2H, CH_{arom}),

5.16 (q, J = 6.7 Hz, 2H, CH), 3.00 (br, 2H, CH), 2.19 (br, 2H, CH), 1.89 (d, J = 6.7 Hz, 6H, CH_3), 1.42 (s, 6H, CH_3), 1.14 (br, 12H, CH_3), 0.97 (br, 6H, CH_3), 0.69 (br, 6H, CH_3) ppm. **^{13}C NMR** (126 MHz, $CDCl_3$): δ = 176.8 (2 x C), 170.6 (2 x C), 156.2 (2 x C), 146.3 (4 x C), 136.4 (CH), 129.9 (2 x C), 128.9 (2 x CH), 123.6 (4 x CH), 106.3 (2 x CH), 105.4 (C), 76.5 (2 x CH), 28.6 (br, 4 x CH), 23.9 (4 x CH_3), 23.4 (4 x CH_3), 19.7 (2 x CH_3), 19.4 (2 x CH_3) ppm. **HRMS** (CI): m/z = 774.2974 calcd. for $C_{38}H_{53}IN_3O_6^+$ [M+NH_4–OAc]$^+$, found: 774.2970. **IR** (neat): ν = 3321*br*, 2963*w*, 1667*m*, 1589*w*, 1506*m*, 1460*s*, 1364*w*, 1248*s*, 1128*w*, 1092*m*, 910*m*, 798*w*, 729*s*, 667*s* cm^{-1}. **[α]$_D^{20}$** = +37.5 (c = 0.4, $CHCl_3$).

(2,6-Bis(((*R*)-1-((2,6-diisopropylphenyl)amino)-1-oxo-3-phenylpropan-2-yl)oxy)phenyl)-λ^3-iodanediyl diacetate (6f)

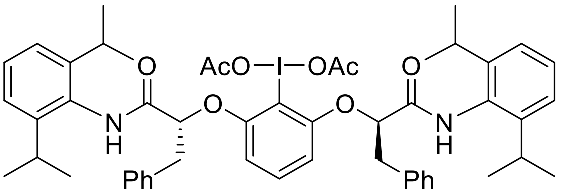

Iodoarene **S4d** (340 mg, 0.400 mmol) was reacted according to **GP6** to give **6f** as a pale yellow solid in 69% yield (267 mg, 0.276 mmol).

M.p.: 168 – 170 °C. **^{1}H NMR** (400 MHz, $CDCl_3$): δ = 8.19 (s, 2H, NH), 7.56 (t, J = 8.4 Hz, 1H, CH_{arom}), 7.46 – 7.40 (m, 4H, CH_{arom}), 7.28 – 6.96 (m, 12H, CH_{arom}), 6.87 (d, J = 8.4 Hz, 2H, CH_{arom}), 5.20 (dd, J = 9.0, 2.9 Hz, 2H, CH), 3.57 (dd, J = 14.2, 2.9 Hz, 2H, CH_2), 3.38 (dd, J = 14.2, 9.0 Hz, 2H, CH_2), 2.84 (br, 2H, CH), 2.08 (br, 2H, CH), 1.33 (s, 6H, CH_3), 1.23 (br, 6H, CH_3), 1.10 (br, 6H, CH_3), 0.89 (br, 6H, CH_3), 0.41 (br, 6H, CH_3) ppm. **^{13}C NMR** (126 MHz, $CDCl_3$): δ = 176.6 (2 x C), 169.1 (2 x C), 155.9 (2 x C), 146.2 (br, 4 x C), 136.2 (CH), 135.9 (2 x C), 129.9 (4 x CH), 129.8 (2 x C), 128.9 (2 x CH), 128.6 (4 x CH), 127.3 (2 x CH), 123.6 (br, 4 x CH), 105.8 (2 x CH), 105.5 (C), 80.7 (2 x CH), 39.3 (2 x CH_2), 28.6 (br, 4 x CH), 24.0 (br, 8 x CH_3) 19.6 (2 x CH_3) ppm. **HRMS** (ASAP): m/z = 909.3340 calcd. for $C_{50}H_{58}IN_2O_6^+$ [M–OAc]$^+$, found: 909.3364. **IR** (neat): ν = 3343*w*, 1678*w*, 1504*w*, 1462*w*, 1263*m*, 1098*m*, 907*s*, 723*s*, 667*m* cm^{-1}. **[α]$_D^{20}$** = +90.0 (c = 0.4, $CHCl_3$).

(2,6-Bis(((*R*)-1-(diisopropylamino)-1-oxopropan-2-yl)oxy)phenyl)-λ^3-iodanediyl diacetate (6g)

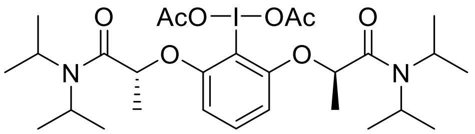

Iodoarene **S4e** (219 mg, 0.400 mmol) was dissolved in dry AcOH (5 mL) under nitrogen atmosphere. Peracetic acid (348 mg, 1.60 mmol, 4.0 equiv., 35 wt.% in AcOH) was added and the solution was stirred for 18 h at room temperature. Afterwards, all volatiles were removed under vacuum and the resulting oil was washed with Et_2O/*n*-hexane (3:1) to afford **6g** as a pale yellow solid in 93% yield (246 mg, 0.370 mmol).

^{1}H NMR (500 MHz, $CDCl_3$): δ = 7.35 (t, *J* = 8.4 Hz, 1H, CH_{arom}), 6.71 (d, *J* = 8.4 Hz, 2H, CH_{arom}), 4.82 (q, *J* = 6.9 Hz, 2H, C*H*), 4.38 (hept, *J* = 6.7 Hz 2H, C*H*), 3.31 (hept, *J* = 6.7 Hz, 2H, C*H*), 1.87 (s, 6H, CH_3), 1.57 (d, *J* = 6.9 Hz, 6H, CH_3), 1.37 (d, *J* = 6.8 Hz, 6H, CH_3), 1.29 (d, *J* = 6.7 Hz, 6H, CH_3), 1.15 (d, *J* = 6.7 Hz, 6H, CH_3), 0.93 (d, *J* = 6.7 Hz, 6H, CH_3) ppm. **^{13}C NMR** (126 MHz, $CDCl_3$): δ = 177.1 (2 x C), 169.1 (2 x C), 157.0 (2 x C), 135.8 (CH), 106.5 (2 x CH), 105.2 (C), 79.4 (2 x CH), 48.2 (2 x CH), 46.7 (2 x CH), 21.0 (2 x CH_3), 20.8 (2 x CH_3), 20.7 (2 x CH_3), 20.5 (2 x CH_3), 20.1 (2 x CH_3), 18.0 (2 x CH_3) ppm. The spectroscopic data are in agreement with the literature.[14]

(2,6-Bis(((*R*)-1-((2,6-diisopropylphenyl)amino)-1-oxopropan-2-yl)oxy)-4-methylphenyl)-λ^3-iodanediyl diacetate (6h)

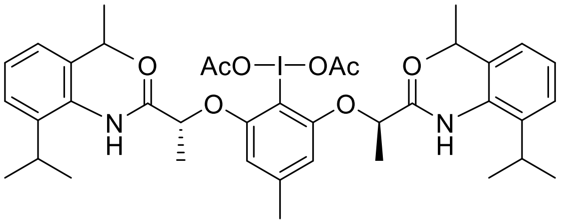

Iodoarene **7b** (285 mg, 0.400 mmol) was reacted according to **GP6** to give **6h** as a yellow solid in 87% yield (288 mg, 0.347 mmol).

^{1}H NMR (500 MHz, $CDCl_3$): δ = 8.25 (s, 2H, N*H*), 7.28 – 7.21 (m, 2H, CH_{arom}), 7.13 (br, 2H, CH_{arom}), 7.06 (br, 2H, CH_{arom}), 5.14 (q, *J* = 6.7 Hz, 2H, C*H*), 2.99 (br, 2H, C*H*), 2.48 (s, 3H, CH_3), 2.20 (br, 2H, C*H*), 1.87 (d, *J* = 6.7 Hz, 6H, CH_3), 1.40 (s, 6H, CH_3), 1.23 (br, 6H, CH_3), 1.15 (br, 6H, CH_3), 0.96 (br, 6H, CH_3), 0.68 (br, 6H, CH_3) ppm. **^{13}C NMR** (126 MHz, $CDCl_3$): δ = 176.7 (2 x C), 170.7 (2 x C), 156.0 (2 x C), 148.8 (C), 146.2 (br, 4 x C), 129.8 (2 x C), 128.9 (2 x CH), 123.6 (br, 4 x CH), 107.0 (2 x CH), 101.8 (C), 76.3 (2 x CH) 28.5 (br, 4 x CH), 23.8 (4 x CH_3), 23.4 (4 x CH_3),

22.7 (CH_3), 19.7 (2 x CH_3), 19.4 (2 x CH_3) ppm. The spectroscopic data are in agreement with the literature.[8]

(4-Bromo-2,6-bis(((*R*)-1-((2,6-diisopropylphenyl)amino)-1-oxopropan-2-yl)oxy)phenyl)-*λ*³-iodanediyl diacetate (6i)

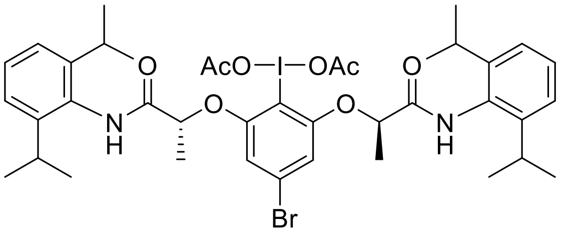

Iodoarene **7c** (311 mg, 0.400 mmol) was reacted according to **GP6**. After 5 h, another portion of Selectfluor® (709 mg, 2.00 mmol, 5.0 equiv.) was added and the reaction mixture was stirred for additional 5 h to reach full conversion. Product **6i** was obtained as a pale yellow solid in 84% yield (299 mg, 0.334 mmol).

M.p.: 188 – 192 °C. **^{1}H NMR** (500 MHz, $CDCl_3$): δ = 8.16 (s, 2H, N*H*), 7.27 (t, *J* = 7.7 Hz, 2H, CH_{arom}), 7.15 (s, 2H, CH_{arom}), 7.12 (br, 4H, CH_{arom}), 5.11 (q, *J* = 6.7 Hz, 2H, C*H*), 3.00 (br, 2H, C*H*), 2.32 (br, 2H, C*H*), 1.89 (d, *J* = 6.7 Hz, 6H, CH_3), 1.45 (s, 6H, CH_3), 1.32 – 1.05 (m, 18H, CH_3), 0.74 (br, 6H, CH_3) ppm. **^{13}C NMR** (126 MHz, $CDCl_3$): δ = 176.8 (2 x C), 170.1 (2 x C), 156.4 (2 x C), 146.3 (4 x C), 131.6 (C), 129.8 (2 x C), 129.1 (2 x CH), 123.7 (br, 4 x CH), 110.1 (2 x CH), 104.7 (C), 77.0 (2 x CH), 28.8 (4 x CH), 23.8 (4 x CH_3), 23.6 (4 x CH_3), 19.7 (2 x CH_3), 19.4 (2 x CH_3) ppm. **HRMS** (ASAP): *m/z* = 835.1819 calcd. for $C_{38}H_{49}IN_2O_6Br^+$ [M−OAc]$^+$, found: 835.1834. **IR** (neat): ν = 3343*m*, 1686*s*, 1645*m*, 1585*w*, 1560*w*, 1506*m*, 1416*w*, 1364*w*, 1267*s*, 1233*m*, 1128*w*, 1101*m*, 1018*w*, 924*w*, 833*w*, 804*w*, 731*w*, 671*s* cm^{-1}. **[*α*]$_D^{20}$** = +183.8 (c = 0.4, $CHCl_3$).

(4-Acetyl-2,6-bis(((*R*)-1-((2,6-diisopropylphenyl)amino)-1-oxopropan-2-yl)oxy)phenyl)-*λ*³-iodanediyl diacetate (6j)

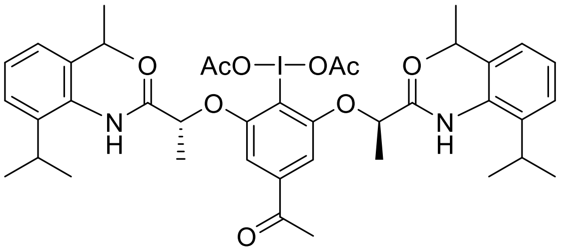

Iodoarene **7d** (297 mg, 0.400 mmol) was reacted according to **GP6**. After 5 h, another portion of Selectfluor® (709 mg, 2.00 mmol, 5.0 equiv.) was added and the reaction mixture was

stirred for additional 5 h to reach full conversion. Product **6j** was obtained as a pale yellow solid in 68% yield (235 mg, 0.274 mmol).

M.p.: 162 – 165 °C. **^{1}H NMR** (500 MHz, $CDCl_3$): δ = 8.20 (s, 2H, N*H*), 7.50 (s, 2H, CH_{arom}), 7.26 (t, *J* = 7.8 Hz, 2H, CH_{arom}), 7.10 (br, 4H, CH_{arom}), 5.29 (q, *J* = 6.7 Hz, 2H, C*H*), 3.00 (br, 2H, C*H*), 2.60 (s, 3H, CH_3), 2.19 (br, 2H, C*H*), 1.91 (d, *J* = 6.7 Hz, 6H, CH_3), 1.45 (s, 6H, CH_3), 1.19 (br, 12H, CH_3), 0.91 (br, 6H, CH_3), 0.67 (br, 6H, CH_3) ppm. **^{13}C NMR** (126 MHz, $CDCl_3$): δ = 195.0 (C), 176.8 (2 x C), 170.1 (2 x C), 156.4 (2 x C), 146.2 (br, 4 x C), 144.0 (C), 129.8 (2 x C), 129.0 (2 x CH), 123.7 (br, 4 x CH), 109.9 (C), 105.5 (2 x CH), 76.8 (2 x CH), 28.7 (br, 4 x CH), 26.8 (CH_3), 23.8 (4 x CH_3), 23.5 (4 x CH_3), 19.7 (2 x CH_3), 19.3 (2 x CH_3) ppm. **HRMS** (ASAP): *m/z* = 799.2819 calcd. for $C_{40}H_{52}IN_2O_7^+$ $[M{-}OAc]^+$, found: 799.2840. **IR** (neat): ν = 3312*w*, 2967*w*, 1682*m*, 1647*w*, 1576*w*, 1510*w*, 1416*w*, 1530*w*, 1217*w*, 1099*w*, 907*m*, 799*w*, 727*s*, 673*w*, 646*w* cm^{-1}. **$[\alpha]_D^{20}$** = +210.0 (c = 0.4, $CHCl_3$).

6 Catalytic Asymmetric *α*-Acetoxylation of Acetyl Enol Ethers

(*R*)-1-Oxo-1-phenylpropan-2-yl acetate ((*R*)-5a)

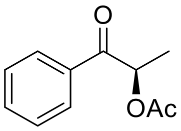

Acetyl enol ether **4a** (53 mg, 0.30 mmol) was reacted according to **GP7**to give (*R*)-**5a** as a colourless oil in 90% yield (52 mg, 0.27 mmol) after flash column chromatography (*n*-hexane:EtOAc = 95:5).

^{1}H NMR (500 MHz, $CDCl_3$): δ = 7.97 – 7.91 (m, 2H, CH_{arom}), 7.62 – 7.56 (m, 1H, CH_{arom}), 7.51 – 7.44 (m, 2H, CH_{arom}), 5.97 (q, *J* = 7.0 Hz, 1H, C*H*), 2.15 (s, 3H, CH_3), 1.53 (d, *J* = 7.0 Hz, 3H, CH_3) ppm. **^{13}C NMR** (126 MHz, $CDCl_3$): δ = 197.0 (C), 170.5 (C), 134.5 (C), 133.7 (CH), 128.9 (2 x CH), 128.6 (2 x CH), 71.5 (CH), 20.9 (CH_3), 17.3 (CH_3) ppm. The spectroscopic data are in agreement with the literature.[15]

(*R*)-1-(4-Fluorophenyl)-1-oxopropan-2-yl acetate ((*R*)-5b)

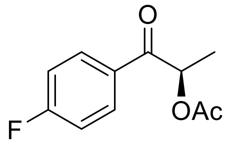

Acetyl enol ether **4b** (58 mg, 0.30 mmol) was reacted according to **GP7** to give (*R*)-**5b** as a colourless oil in 75% yield (47 mg, 0.22 mmol) after flash column chromatography (*n*-hexane:EtOAc = 95:5).

^{1}H NMR (500 MHz, $CDCl_3$): δ = 8.01 – 7.94 (m, 2H, CH_{arom}), 7.18 – 7.12 (m, 2H, CH_{arom}), 5.91 (q, J = 7.0 Hz, 1H, C*H*), 2.16 – 2.12 (m, 3H, CH_3), 1.52 (d, J = 7.0 Hz, 3H, CH_3) ppm. **^{13}C NMR** (126 MHz, $CDCl_3$): δ = 195.5 (C), 170.6 (C), 166.1 (d, J = 255.8 Hz, C), 131.3 (d, J = 9.4 Hz, 2 x CH), 130.9 (d, J = 3.1 Hz, C), 116.1 (d, J = 22.0 Hz, 2 x CH), 71.4 (CH), 20.8 (CH_3), 17.2 (CH_3) ppm. **^{19}F NMR** (376 MHz, $CDCl_3$): δ = −104.0 (s, 1F, C*F*) ppm. **[α]$_D^{20}$** = −31.7 (c = 1.0, $CHCl_3$). The spectroscopic data are in agreement with the literature.[15]

(*R*)-1-(3-Fluorophenyl)-1-oxopropan-2-yl acetate ((*R*)-5c)

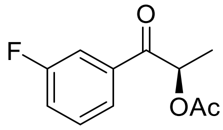

Acetyl enol ether **4c** (58 mg, 0.30 mmol) was reacted according to **GP7** to give (*R*)-**5c** as a colourless oil in 97% yield (61 mg, 0.29 mmol) after flash column chromatography (*n*-hexane:EtOAc = 90:10).

^{1}H NMR (500 MHz, $CDCl_3$): δ = 7.72 (ddd, J = 7.8, 1.6, 1.0 Hz, 1H, CH_{arom}), 7.63 (ddd, J = 2.6, 1.6, 0.3 Hz 1H, CH_{arom}), 7.50 – 7.43 (m, 1H, CH_{arom}), 7.30 (tdd, J = 8.3, 2.6, 1.0 Hz, 1H, CH_{arom}), 5.88 (q, J = 7.0 Hz, 1H, C*H*), 2.15 (s, 3H, CH_3), 1.53 (d, J = 7.1 Hz, 3H, CH_3) ppm. **^{13}C NMR** (126 MHz, $CDCl_3$): δ = 195.9 (d, J = 2.1 Hz, C), 170.5 (C), 163.0 (d, J = 248.5 Hz, CF), 136.5 (d, J = 6.3 Hz, C), 130.6 (d, J = 7.7 Hz, CH), 124.2 (d, J = 3.1 Hz, CH), 120.8 (d, J = 21.4 Hz, CH), 115.4 (d, J = 22.6 Hz, CH), 71.6 (CH), 20.8 (CH_3), 17.1 (CH_3) ppm. **^{19}F NMR** (471 MHz, $CDCl_3$): δ = −111.3 (td, J = 8.7, 5.6 Hz, 1F, C*F*) ppm. **HRMS** (ESI): *m/z* = 211.0770 calcd. for $C_{11}H_{12}O_3F^+$ $[M+H]^+$, found: 211.0768. **IR** (neat): ν = 1740*m*, 1697*m*, 1587*m*, 1485*w*, 1443*w*, 1371*m*, 1231*s*, 1125*w*, 1088*m*, 1040*m*, 989*w*, 839*m*, 799*w*, 764*w*, 737*w*, 673*m*, 602*w* cm^{-1}. **[α]$_D^{20}$** = −36.3 (c = 0.4, $CHCl_3$).

(*R*)-1-(4-Bromophenyl)-1-oxopropan-2-yl acetate ((*R*)-5d)

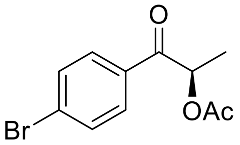

Acetyl enol ether **4d** (77 mg, 0.30 mmol) was reacted according to **GP7** to give (*R*)-**5d** as a pale yellow oil in 92% yield (75 mg, 0.28 mmol) after flash column chromatography (*n*-hexane:EtOAc = 90:10).

^{1}H NMR (500 MHz, $CDCl_3$): δ = 7.83 – 7.75 (m, 2H, CH_{arom}), 7.66 – 7.58 (m, 2H, CH_{arom}), 5.88 (q, J = 7.0 Hz, 1H, C*H*), 2.13 (s, 3H, CH_3), 1.51 (d, J = 7.0 Hz, 3H, CH_3) ppm. **^{13}C NMR** (126 MHz, $CDCl_3$): δ = 196.1 (C), 170.5 (C), 133.2 (C), 132.2 (2 x CH), 130.0 (2 x CH), 128.9 (C), 71.4 (CH), 20.8 (CH_3), 17.1 (CH_3) ppm. **$[\alpha]_D^{20}$** = –26.3 (c = 0.40, $CHCl_3$). The spectroscopic data are in agreement with the literature.[16]

(*R*)-1-Oxo-1-(3-(trifluoromethyl)phenyl)propan-2-yl acetate ((*R*)-5e)

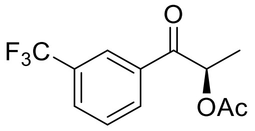

Acetyl enol ether **4e** (73 mg, 0.30 mmol) was for reacted for 5 h according to **GP7** to give (*R*)-**5e** as a colourless oil in 78% yield (61 mg, 0.23 mmol) after flash column chromatography (*n*-hexane:EtOAc = 90:10).

^{1}H NMR (500 MHz, $CDCl_3$): δ = 8.19 (s, 1H, CH_{arom}), 8.12 (d, J = 7.8 Hz, 1H, CH_{arom}), 7.85 (d, J = 7.8 Hz, 1H, CH_{arom}), 7.63 (t, J = 7.8 Hz, 1H, CH_{arom}), 5.91 (q, J = 7.1 Hz, 1H, C*H*), 2.15 (s, 3H, $OCOCH_3$), 1.55 (d, J = 7.1 Hz, 3H, CH_3) ppm. **^{13}C NMR** (126 MHz, $CDCl_3$): δ = 196.0 (C), 170.6 (C), 135.1 (C), 131.7 (d, J = 0.9 Hz, CH), 131.6 (q, J = 33.1 Hz, C), 130.1 (q, J = 3.6 Hz, CH), 129.6 (CH), 125.5 (q, J = 3.8 Hz, CH), 123.7 (q, J = 272.6 Hz, CF_3), 71.6 (CH), 20.8 (CH_3), 17.1 (CH_3) ppm. **^{19}F NMR** (376 MHz, $CDCl_3$): δ = –62.9 (s, 3F, CF_3) ppm. **HRMS** (ESI): *m/z* = 283.0558 calcd. for $C_{12}H_{11}O_3F_3Na^+$ $[M+Na]^+$, found: 283.0556. **IR** (neat): ν = 1742m, 1703*s*, 1612*w*, 1373*w*, 1329*s*, 1238*w*, 1209*w*, 1169*w*, 1123*m*, 1072*w*, 1039*w*, 982*w*, 814*w*, 692*s*, 652*w* cm^{-1}. **$[\alpha]_D^{20}$** = –29.8 (c = 0.4, $CHCl_3$).

(*R*)-1-Oxo-1-(2-(trifluoromethyl)phenyl)propan-2-yl acetate ((*R*)-5f)

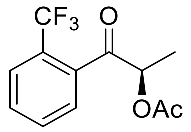

Acetyl enol ether **4f** (73 mg, 0.30 mmol) was for reacted for 20 h according to **GP7** to give (*R*)-**5f** as a colourless oil in 51% yield (40 mg, 0.15 mmol) after flash column chromatography (*n*-hexane:EtOAc = 90:10).

^{1}H NMR (500 MHz, $CDCl_3$): δ = 7.78 – 7.73 (m, 1H, CH_{arom}), 7.73 – 7.68 (m, 1H, CH_{arom}), 7.66 – 7.58 (m, 2H, CH_{arom}), 5.76 (q, J = 7.1 Hz, 1H, C*H*), 2.09 (s, 3H, CH_3), 1.45 (d, J = 7.1 Hz, 3H, CH_3) ppm. **^{13}C NMR** (126 MHz, $CDCl_3$): δ = 200.4 (C), 170.5 (C), 136.9 (d, J = 1.9 Hz, C), 131.9 (CH), 131.0 (CH), 128.3 (q, J = 32.5 Hz, C), 127.9 (CH), 127.2 (q, J = 4.9 Hz, CH), 123.4 (q, J = 273.7 Hz, CF_3), 73.9 (CH), 20.7 (CH_3), 16.0 (CH_3) ppm. **^{19}F NMR** (471 MHz, $CDCl_3$): δ = −58.3 (s, 3F, CF_3) ppm. **HRMS** (ESI): m/z = 261.0739 calcd. for $C_{12}H_{12}O_3F^+$ [M+H]$^+$, found: 261.0735. **IR** (neat): ν = 1748*m*, 1719*m*, 1447*w*, 1371*w*, 1312*s*, 1223*m*, 1171*w*, 1130*m*, 1063*w*, 1032*m*, 934*w*, 864*w*, 770*s*, 685*w* cm^{-1}.

(*R*)-1-(3-Nitrophenyl)-1-oxopropan-2-yl acetate ((*R*)-5g)

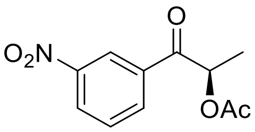

Acetyl enol ether **4g** (66 mg, 0.30 mmol) was for reacted for 20 h according to **GP7** to give (*R*)-**5g** as a colourless solid in 61% yield (43 mg, 0.18 mmol) after flash column chromatography (*n*-hexane:EtOAc = 85:15).

M.p.: 49 – 52 °C. **^{1}H NMR** (500 MHz, $CDCl_3$): δ = 8.78 – 8.73 (m, 1H, CH_{arom}), 8.47 – 8.40 (m, 1H, CH_{arom}), 8.29 – 8.23 (m, 1H, CH_{arom}), 7.70 (t, J = 7.8 Hz, 1H, CH_{arom}), 5.89 (q, J = 7.0 Hz, 1H, C*H*), 2.14 (s, 3H, CH_3), 1.56 (d, J = 7.0 Hz, 3H, CH_3) ppm. **^{13}C NMR** (126 MHz, $CDCl_3$): δ = 195.3 (C), 170.6 (C), 148.6 (C), 135.8 (C), 134.1 (CH), 130.3 (CH), 127.9 (CH), 123.4 (CH), 71.6 (CH), 20.7 (CH_3), 17.0 (CH_3) ppm. **HRMS** (ESI): m/z = 238.0715 calcd. for $C_{11}H_{12}NO_5^+$ [M+H]$^+$, found: 238.0723. **IR** (neat): ν = 1722*m*, 1701*m*, 1610*m*, 1526*s*, 1445*w*, 1350*m*, 1240*m*, 1221*m*, 1140*w*, 1099*w*, 1076*m*, 1040*m*, 982*m*, 941*m*, 910*w*, 860*w*, 827*m*, 787*w*, 710*s*, 662*w* cm^{-1}. **$[\alpha]_D^{20}$** = –14.2 (c = 0.9, $CHCl_3$).

(*R*)-1-Oxo-1-(*p*-tolyl)propan-2-yl acetate ((*R*)-5h)

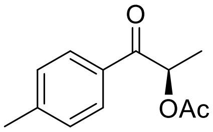

Acetyl enol ether **4h** (57 mg, 0.30 mmol) was reacted according to **GP7** to give (*R*)-**5h** as a colourless solid in 74% yield (46 mg, 0.22 mmol) after flash column chromatography (*n*-hexane:EtOAc = 90:10).

^{1}H NMR (500 MHz, $CDCl_3$): δ = 7.86 – 7.81 (m, 2H, CH_{arom}), 7.29 – 7.26 (m, 2H, CH_{arom}), 5.95 (q, J = 7.0 Hz, 1H, C*H*), 2.42 (s, 3H, CH_3), 2.14 (s, 3H, CH_3), 1.52 (d, J = 7.0 Hz, 3H, CH_3) ppm. **^{13}C NMR** (126 MHz, $CDCl_3$): δ = 196.5 (C), 170.6 (C), 144.7 (C), 131.9 (C), 129.6 (2 x CH), 128.7 (2 x CH), 71.5 (CH), 21.8 (CH_3), 20.9 (CH_3), 17.4 (CH_3) ppm. **$[\alpha]_D^{20}$** = −33.7 (c = 0.40, $CHCl_3$). The spectroscopic data are in agreement with the literature.[15]

(*R*)-1-(4-(*tert*-Butyl)phenyl)-1-oxopropan-2-yl acetate ((*R*)-5i)

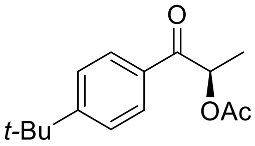

Acetyl enol ether **4i** (70 mg, 0.30 mmol) was reacted according to **GP7** to give (*R*)-**5i** as a colourless oil in 74% yield (55 mg, 0.22 mmol) after flash column chromatography (*n*-hexane:EtOAc = 90:10).

^{1}H NMR (500 MHz, $CDCl_3$): δ = 7.91 – 7.86 (m, 2H, CH_{arom}), 7.52 – 7.46 (m, 2H, CH_{arom}), 5.97 (q, J = 7.0 Hz, 1H, C*H*), 2.15 (s, 3H, CH_3), 1.53 (d, J = 7.0 Hz, 3H, CH_3), 1.34 (s, 9H, CH_3) ppm. **^{13}C NMR** (126 MHz, $CDCl_3$): δ = 196.5 (C), 170.6 (C), 157.6 (C), 131.8 (C), 128.6 (2 x CH), 125.9 (2 x CH), 71.5 (CH), 35.3 (C), 31.2 (3 x CH_3), 20.9 (CH_3), 17.4 (CH_3) ppm. **HRMS** (ESI): *m/z* = 271.1310 calcd. for $C_{15}H_{20}O_3Na^+$ $[M+Na]^+$, found: 271.1311. **IR** (neat): ν = 2965*w*, 1742*m*, 1964*s*, 1605*m*, 1410*w*, 1369*m*, 1231*s*, 1196*w*, 1134*w*, 1109*w*, 1088*m*, 1038*m*, 974*s*, 935*m*, 847*m*, 719*w*, 604*w* cm^{-1}. **$[\alpha]_D^{20}$** = –22.1 (c = 0.43, $CHCl_3$).

(*R*)-1-([1,1'-Biphenyl]-4-yl)-1-oxopropan-2-yl acetate ((*R*)-5j)

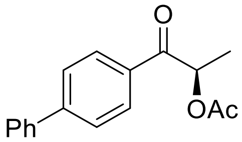

Acetyl enol ether **4j** (76 mg, 0.30 mmol) was reacted according to **GP7** to give (*R*)-**5j** as a colourless solid in 94% yield (76 mg, 0.28 mmol) after flash column chromatography (*n*-hexane:EtOAc = 90:10).

M.p.: 71 – 74 °C. ^{1}**H NMR** (500 MHz, $CDCl_3$): δ = 8.06 – 7.99 (m, 2H, CH_{arom}), 7.73 – 7.67 (m, 2H, CH_{arom}), 7.65 – 7.59 (m, 2H, CH_{arom}), 7.51 – 7.44 (m, 2H, CH_{arom}), 7.43 – 7.37 (m, 1H, CH_{arom}), 6.00 (q, *J* = 7.0 Hz, 1H C*H*), 2.16 (s, 3H, CH_3), 1.56 (d, *J* = 7.0 Hz, 3H, CH_3) ppm. ^{13}**C NMR** (126 MHz, $CDCl_3$): δ = 196.5 (C), 170.5 (C), 146.4 (C), 139.8 (C), 133.1 (C), 129.2 (2 x CH), 129.1 (2 x CH), 128.5 (CH), 127.5 (2 x CH), 127.4 (2 x CH), 71.5 (CH), 20.8 (CH_3), 17.3 (CH_3) ppm. **HRMS** (ESI): *m/z* = 291.0997 calcd. for $C_{17}H_{16}O_3Na^+$ $[M+Na]^+$, found: 291.0997. **IR** (neat): ν = 1734*s*, 1688*s*, 1605*m*, 1445*w*, 1404*w*, 1369*m*, 1236*s*, 1130*m*, 1088*m*, 1038*m*, 974*m*, 939*m*, 866*w*, 843*m*, 770*m*, 741*m*, 694*s*, 631*w* cm^{-1}. $[\alpha]_D^{20}$ = –15.7 (c = 0.40, $CHCl_3$).

(*R*)-1-(4-Methoxyphenyl)-1-oxopropan-2-yl acetate ((*R*)-5k)

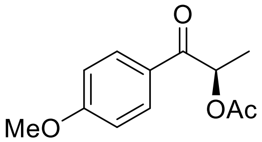

Acetyl enol ether **4k** (62 mg, 0.30 mmol) and hypervalent iodine reagent **6h** (316 mg, 0.38 mmol, 1.25 equiv.) were dissolved in CH_2Cl_2 (1.5 mL) under nitrogen atmosphere. After the addition of $BF_3{\cdot}OEt_2$ (11 µL, 0.090 mmol, 30 mol%) the solution was stirred at room temperature for 2 h. Subsequently, saturated aqueous $Na_2S_2O_3$ (5 mL) was added and the resulting mixture was extracted with CH_2Cl_2 (3 x 10 mL) The combined organic layers dried over anhydrous $MgSO_4$ and concentrated under vacuum. The crude product was purified by flash column chromatography (*n*-hexane:EtOAc = 90:10) to give (*R*)-**5k** as a pale yellow solid in 81% (54 mg, 0.24 mmol) yield.

^{1}**H NMR** (500 MHz, $CDCl_3$): δ = 7.97 – 7.91 (m, 2H, CH_{arom}), 6.98 – 6.92 (m, 2H, CH_{arom}), 5.94 (q, *J* = 7.0 Hz, 1H, C*H*), 3.87 (s, 3H, CH_3), 2.14 (s, 3H, CH_3), 1.52 (d, *J* = 7.0 Hz, 3H, CH_3) ppm. ^{13}**C NMR** (126 MHz, $CDCl_3$): δ = 195.4 (C), 170.6 (C), 164.0 (C), 131.0 (2 x CH), 127.3 (C), 114.1 (2 x CH),

71.3 (CH), 55.7 (CH_3), 20.9 (CH_3), 17.5 (CH_3) ppm. The spectroscopic data are in agreement with the literature.[15]

(*R*)-2-Oxo-1,2-diphenylethyl acetate ((*R*)-5l)

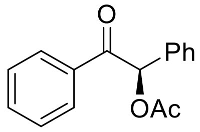

Acetyl enol ether **4l** (71 mg, 0.30 mmol) was reacted for 20 h according to **GP7** to give (*R*)-**5l** as a colourless solid in 52% yield (40 mg, 0.16 mmol) after flash column chromatography (*n*-hexane:EtOAc = 95:5).

^{1}H NMR (500 MHz, $CDCl_3$): δ = 7.97 – 7.92 (m, 2H, CH_{arom}), 7.53 – 7.46 (m, 3H, CH_{arom}), 7.43 – 7.33 (m, 5H, CH_{arom}), 6.88 (s, 1H, C*H*), 2.21 (s, 3H, CH_3) ppm. **^{13}C NMR** (126 MHz, $CDCl_3$): δ = 193.8 (C), 170.6 (C), 134.7 (C), 133.7 (C), 133.6 (CH), 129.5 (CH), 129.3 (2 x CH), 128.9 (2 x CH), 128.8 (2 x CH), 128.8 (2 x CH), 77.8 (CH), 20.9 (CH_3) ppm. The spectroscopic data are in agreement with the literature.[17]

(*R*)-3-Methyl-1-oxo-1-phenylbutan-2-yl acetate ((*R*)-5m)

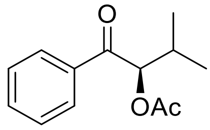

Acetyl enol ether **4m** (61 mg, 0.30 mmol) was reacted for 20 h according to **GP7** to give (*R*)-**5m** as a pale yellow oil in 9% yield (6 mg, 0.03 mmol) after flash column chromatography (*n*-hexane:EtOAc = 100:0 → 95:5).

^{1}H NMR (500 MHz, $CDCl_3$): δ = 7.97 – 7.92 (m, 2H, CH_{arom}), 7.61 – 7.55 (m, 1H, CH_{arom}), 7.51 – 7.45 (m, 2H, CH_{arom}), 5.74 (d, *J* = 4.7 Hz, 1H, C*H*), 2.32 – 2.25 (m, 1H, C*H*), 2.17 (s, 3H, CH_3), 1.04 (d, *J* = 6.9 Hz, 3H, CH_3), 0.93 (d, *J* = 6.8 Hz, 3H, CH_3) ppm. **^{13}C NMR** (126 MHz, $CDCl_3$): δ = 196.9 (C), 171.0 (C), 135.8 (C), 133.6 (CH), 128.9 (2 x CH), 128.5 (2 x CH), 79.5 (CH), 30.3 (CH), 20.8 (CH_3), 19.7 (CH_3), 17.1 (CH_3) ppm.

(*R*)-1-Oxo-1-phenylbutan-2-yl acetate ((*R*)-5n)

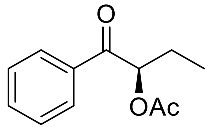

Acetyl enol ether **4n** (57 mg, 0.30 mmol) was reacted according to **GP7** to give (*R*)-**5n** as a colourless oil in 84% yield (51 mg, 0.25 mmol) after flash column chromatography (*n*-hexane:EtOAc = 90:10).

^{1}H NMR (500 MHz, $CDCl_3$): δ = 7.96 – 7.92 (m, 2H, CH_{arom}), 7.61 – 7.56 (m, 1H, CH_{arom}), 7.50 – 7.44 (m, 2H, CH_{arom}), 5.82 (dd, *J* = 8.2, 4.3 Hz, 1H, C*H*), 2.16 (s, 3H, CH_3), 2.01 – 1.91 (m, 1H, CH_2), 1.91 – 1.80 (m, 1H, CH_2), 1.02 (t, *J* = 7.4 Hz, 3H, CH_3) ppm. **^{13}C NMR** (126 MHz, $CDCl_3$): δ = 196.7 (C), 170.8 (C), 135.0 (C), 133.7 (CH), 128.9 (2 x CH), 128.5 (2 x CH), 76.5 (CH), 24.9 (CH_2), 20.8 (CH_3), 10.0 (CH_3) ppm. **$[\alpha]_D^{20}$** = −9.6 (c = 0.40, $CHCl_3$). The spectroscopic data are in agreement with the literature.[18]

(*R*)-1-Oxo-1,2,3,4-tetrahydronaphthalen-2-yl acetate ((*R*)-5o)

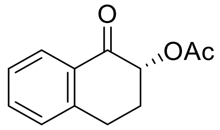

Acetyl enol ether **4o** (56 mg, 0.30 mmol) was reacted according to **GP7** to give (*R*)-**5o** as a pale yellow solid in 62% yield (38 mg, 0.19 mmol) after flash column chromatography (*n*-hexane:EtOAc = 95:5).

^{1}H NMR (500 MHz, $CDCl_3$): δ = 8.02 (d, *J* = 7.7 Hz, 1H, CH_{arom}), 7.50 (t, *J* = 7.5 Hz, 1H, CH_{arom}), 7.32 (t, *J* = 7.7 Hz, 1H, CH_{arom}), 7.25 (d, *J* = 7.5 Hz, 1H, CH_{arom}), 5.54 (dd, *J* = 13.5, 5.1 Hz, 1H, C*H*), 3.26 – 3.15 (m, 1H, CH_2), 3.13 – 3.04 (m, 1H, CH_2), 2.44 – 2.35 (m, 1H, CH_2), 2.33 – 2.24 (m, 1H, CH_2), 2.22 (s, 3H, CH_3) ppm. **^{13}C NMR** (126 MHz, $CDCl_3$): δ = 193.1 (C), 170.3 (C), 143.2 (C), 134.0 (CH), 131.7 (C), 128.8 (CH), 127.9 (CH), 127.1 (CH), 74.7 (C), 29.3 (CH_2), 28.1 (CH_2), 21.0 (CH_3) ppm. **$[\alpha]_D^{20}$** = +10.3 (c = 0.42, $CHCl_3$). The spectroscopic data are in agreement with the literature.[19]

(*R*)-1-Oxo-1-(thiophen-2-yl)propan-2-yl acetate ((*R*)-5p)

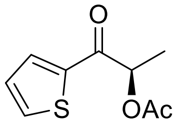

Acetyl enol ether **4p** (55 mg, 0.30 mmol) was reacted according to **GP7** to give (*R*)-**5p** as a colourless oil in 92% yield (55 mg, 0.28 mmol) after flash column chromatography (*n*-hexane:EtOAc = 90:10).

^{1}H NMR (500 MHz, $CDCl_3$): δ = 7.80 (dd, *J* = 3.8, 1.1 Hz, 1H, CH_{arom}), 7.69 (dd, *J* = 4.9, 1.1 Hz, 1H, CH_{arom}), 7.15 (dd, *J* = 4.9, 3.8 Hz, 1H, CH_{arom}), 5.73 (q, *J* = 7.0 Hz, 1H, C*H*), 2.14 (s, 3H, CH_3), 1.56 (d, *J* = 7.0 Hz, 3H, CH_3) ppm. **^{13}C NMR** (126 MHz, $CDCl_3$): δ = 189.9 (C), 170.4 (C), 140.6 (C), 134.6 (CH), 132.8 (CH), 128.4 (CH), 72.5 (CH), 20.9 (CH_3), 17.8 (CH_3) ppm.

1-Cyano-2-oxo-2-phenylethyl acetate ((*rac*)-5q)

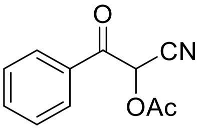

Acetyl enol ether **4q** (56 mg, 0.3 mmol) and (diacetoxyiodo)benzene (122 mg, 0.38 mmol, 1.25 equiv.) were dissolved in CH_2Cl_2 (1.5 mL) and $BF_3{\cdot}OEt_2$ (74 µL, 0.6 mmol, 2.0 equiv.) was added. After the solution was stirred at room temperature for 20 h, saturated aqueous $Na_2S_2O_3$ (5 mL) was added and the resulting mixture was extracted with CH_2Cl_2 (3 x 5 mL). The combined organic layers dried over anhydrous $MgSO_4$ and concentrated under vacuum. The crude mixture was purified by flash column chromatography (*n*-hexane:EtOAc = 90:10) to afford (*rac*)-**5q** as a pale yellow oil in 30% (18 mg, 0.089 mmol) yield.

^{1}H NMR (500 MHz, $CDCl_3$): δ = 8.00 – 7.96 (m, 2H, CH_{arom}), 7.72 – 7.68 (m, 1H, CH_{arom}), 7.58 – 7.54 (m, 2H, CH_{arom}), 6.55 (s, 1H, C*H*), 2.27 (s, 3H, CH_3) ppm. **^{13}C NMR** (126 MHz, $CDCl_3$): δ = 184.5 (C), 168.7 (C), 135.4 (CH), 132.2 (C), 129.4 (2 x CH), 129.0 (2 x CH), 113.2 (C), 63.2 (C), 20.3 (CH_3) ppm. **HRMS** (EI): *m/z* = 203.0582 calcd. for $C_{11}H_9NO_3^+$ $[M]^+$, found: 203.0574. **IR** (neat): ν = 2345*w*, 1769*s*, 1717*s*, 1598*m*, 1541*w*, 1450*m*, 1373*m*, 1217*s*, 1086*m*, 1045*w*, 943*w*, 760*w*, 692*m* cm^{-1}.

(2*S*)-2-[(4-Methylbenzenesulfonyl)oxy]-1-phenylpropan-1-one ((*S*)-S5)

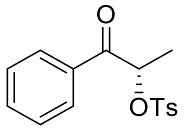

Acetyl enol ether **4a** (55 mg, 0.30 mmol) was reacted according to **GP7** with TsOH·H_2O (17 mg, 0.090 mmol, 30 mol%) instead of BF_3·OEt_2 to give side product **((*S*)-S5** as a colourless oil in 28% yield (26 mg, 0.085 mmol) after flash column chromatography (*n*-hexane:EtOAc = 95:5). **$[\alpha]_D^{20}$** = −3.4 (c = 0.41, $CHCl_3$).

^{1}H NMR (400 MHz, $CDCl_3$): δ = 7.90 – 7.85 (m, 2H, CH_{arom}), 7.77 – 7.72 (m, 2H, CH_{arom}), 7.62 – 7.56 (m, 1H, CH_{arom}), 7.49 – 7.42 (m, 2H, CH_{arom}), 7.29 – 7.25 (m, 2H, CH_{arom}), 5.78 (q, *J* = 6.9 Hz, 1H, C*H*), 2.41 (s, 3H, CH_3), 1.60 (d, *J* = 6.9 Hz, 3H, CH_3) ppm. **^{13}C NMR** (101 MHz, $CDCl_3$): δ = 195.0 (C), 145.1(C), 134.0 (CH), 133.8 (C), 133.6 (C), 129.9 (2 x CH), 128.9 (2 x CH), 128.9 (2 x CH), 128.1 (2 x CH), 77.5 (CH), 21.8 (CH_3), 18.9 (CH_3) ppm. **$[\alpha]_D^{20}$** = −3.4 (c = 0.41, $CHCl_3$). The spectroscopic data are in agreement with the literature.[20]

7 Kinetic Studies

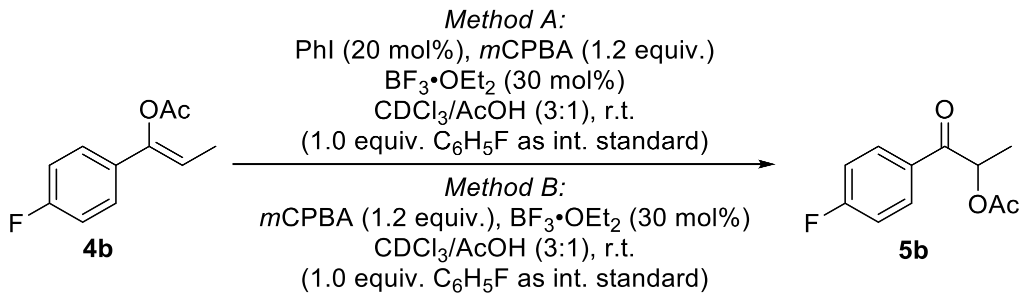

Method A: Acetyl enol ether **4b** (19 mg, 0.10 mmol) was dissolved in $CHCl_3$ (0.37 mL) and AcOH (0.13 mL) in a *Young* NMR tube and iodobenzene (4.1 mg, 0.020 mmol, 20 mol%), fluorobenzene (9.6 mg, 0.10 mmol, 1.0 equiv.), $BF_3{\cdot}OEt_2$ (4 µL, 0.03 mmol, 30 mol%) and *m*CPBA (27 mg, 0.12 mmol, 1.2 equiv., 77% purity) were added. The NMR tube was sealed and the reaction was followed by ^{19}F NMR over a period of 1.25 hours (Table S2).

Table S2: Conversion of **4b** in presence of iodobenzene over a period of 1.25 hours.

Time [h]	Conversion of 4b [%]
0.00	0
0.10	37
0.25	63
0.50	85
1.00	97
1.25	100

Method B: Acetyl enol ether **4b** (19 mg, 0.10 mmol) was dissolved in $CHCl_3$ (0.37 mL) and AcOH (0.13 mL) in a *Young* NMR tube and fluorobenzene (9.6 mg, 0.10 mmol, 1.0 equiv.), $BF_3 \cdot OEt_2$ (4 µL, 0.03 mmol, 30 mol%) and *m*CPBA (27 mg, 0.12 mmol, 1.2 equiv., 77% purity) were added. The NMR tube was sealed and the reaction was followed by ^{19}F NMR over a period of 15 hours (Table S3). The corresponding diagram can be found in Scheme S1.

Table S3: Conversion of **4b** in absence of iodobenzene over a period of 15 hours.

Time [h]	Conversion of 4b [%]
0.0	0
0.5	29
1.0	45
2.0	62
3.0	71
4.0	77
6.0	82
8.0	84
10	86
12	87
15	88

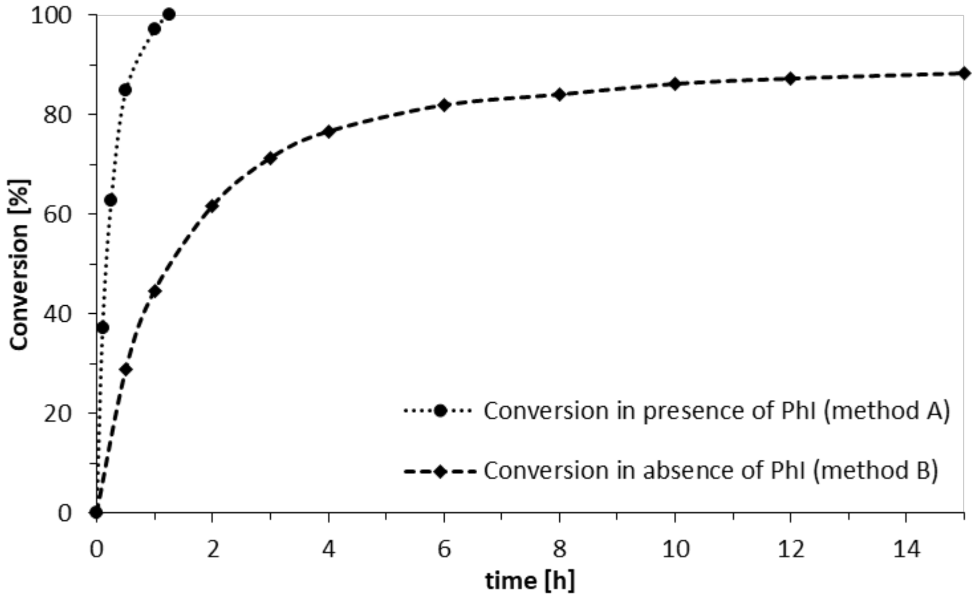

Scheme S1: Reaction kinetics of **4b** in the presence (method A) and absence of the iodobenzene as catalyst (method B).

8 NMR spectra

(*Z*)-1-Phenyl-1-triethylsiloxy-1-propene (3)

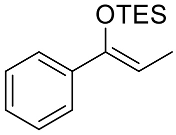

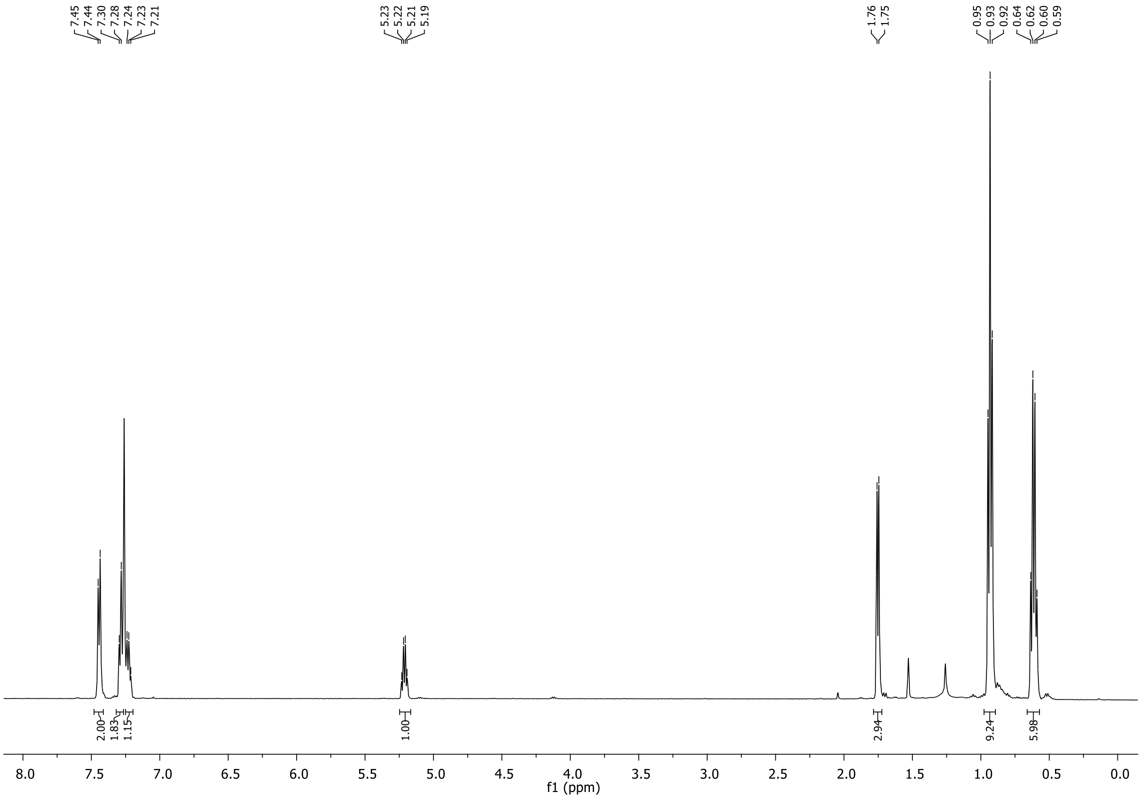

(*Z*)-1-Phenylprop-1-en-1-yl acetate (4a)

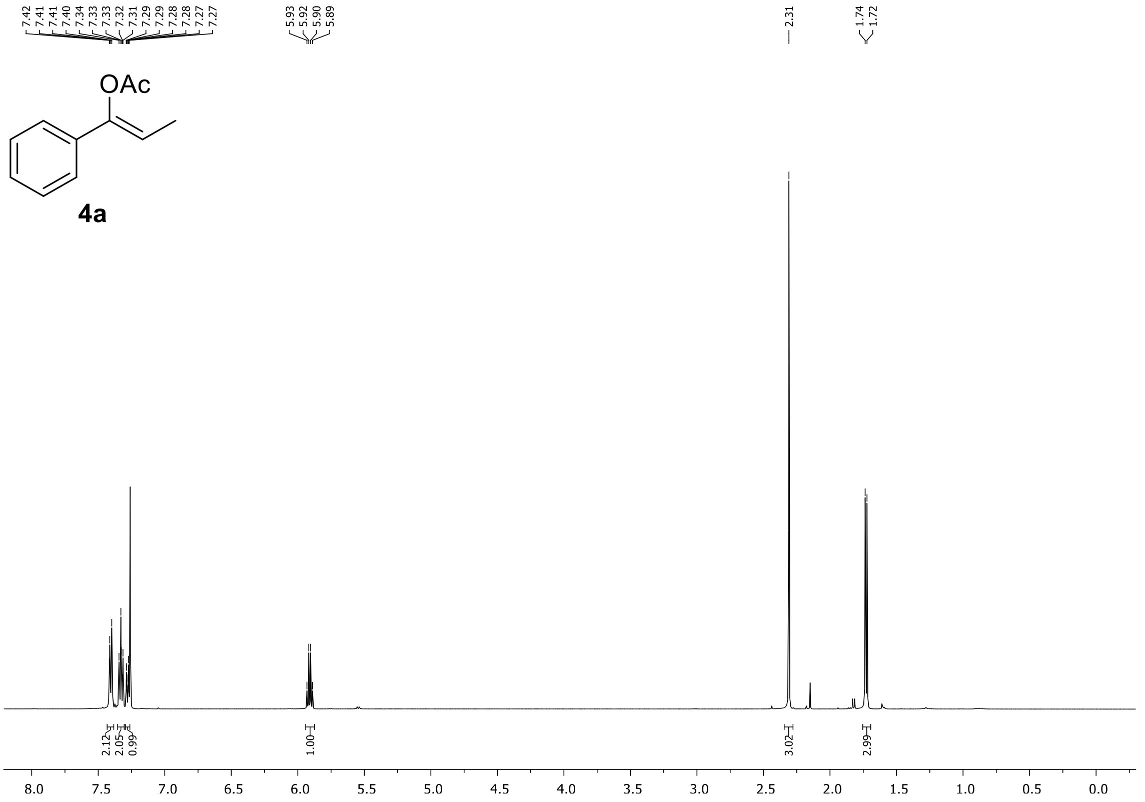

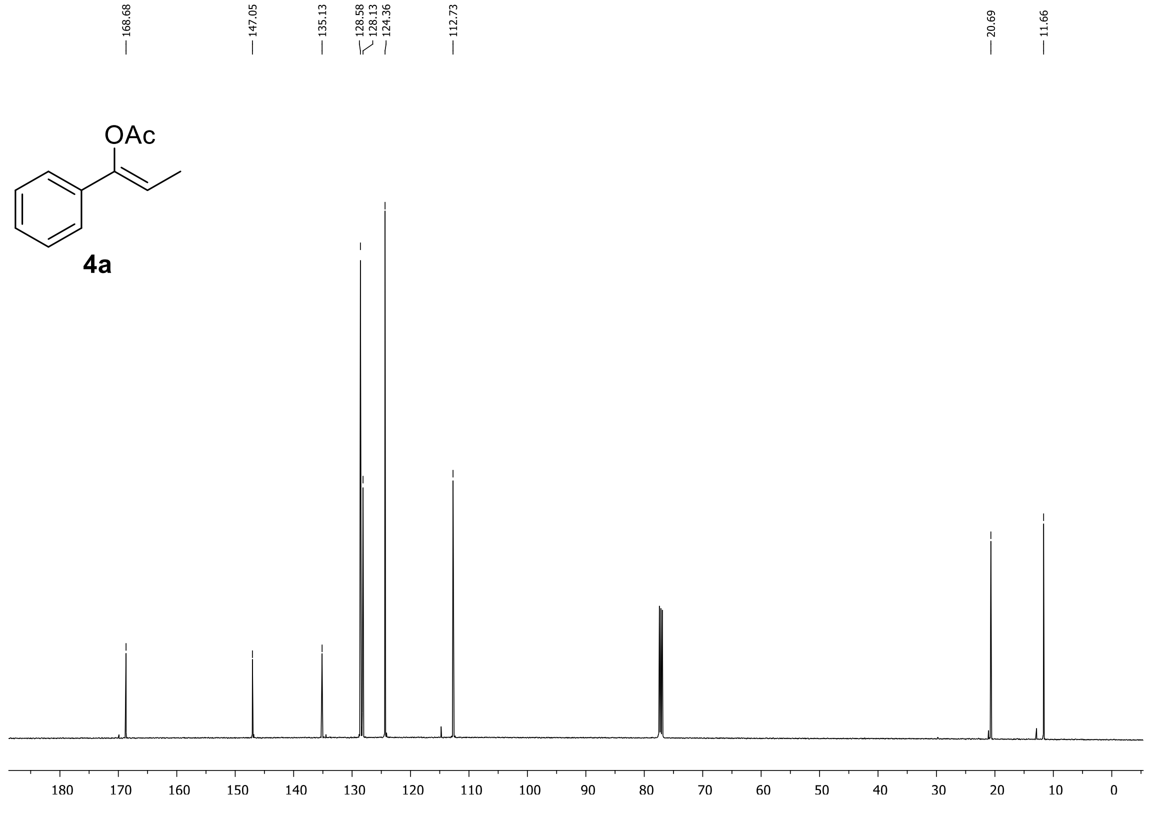

(*Z*)-1-(4-Fluorophenyl)prop-1-en-1-yl acetate (4b)

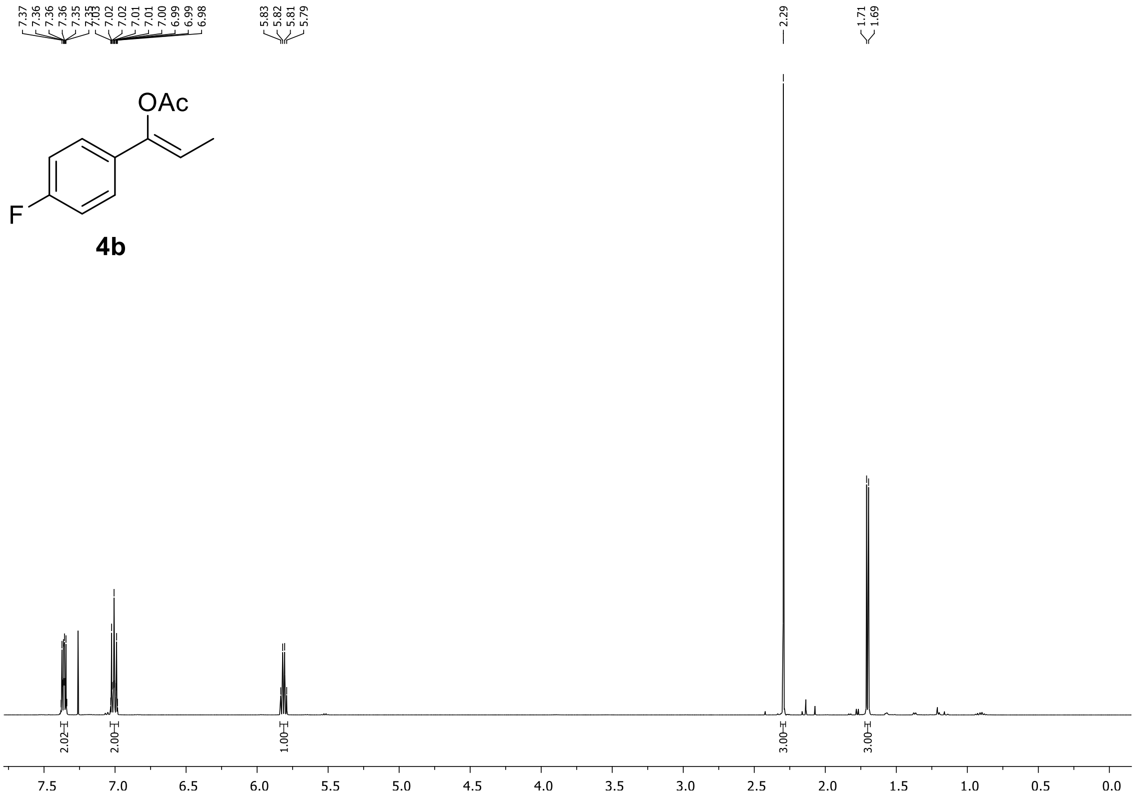

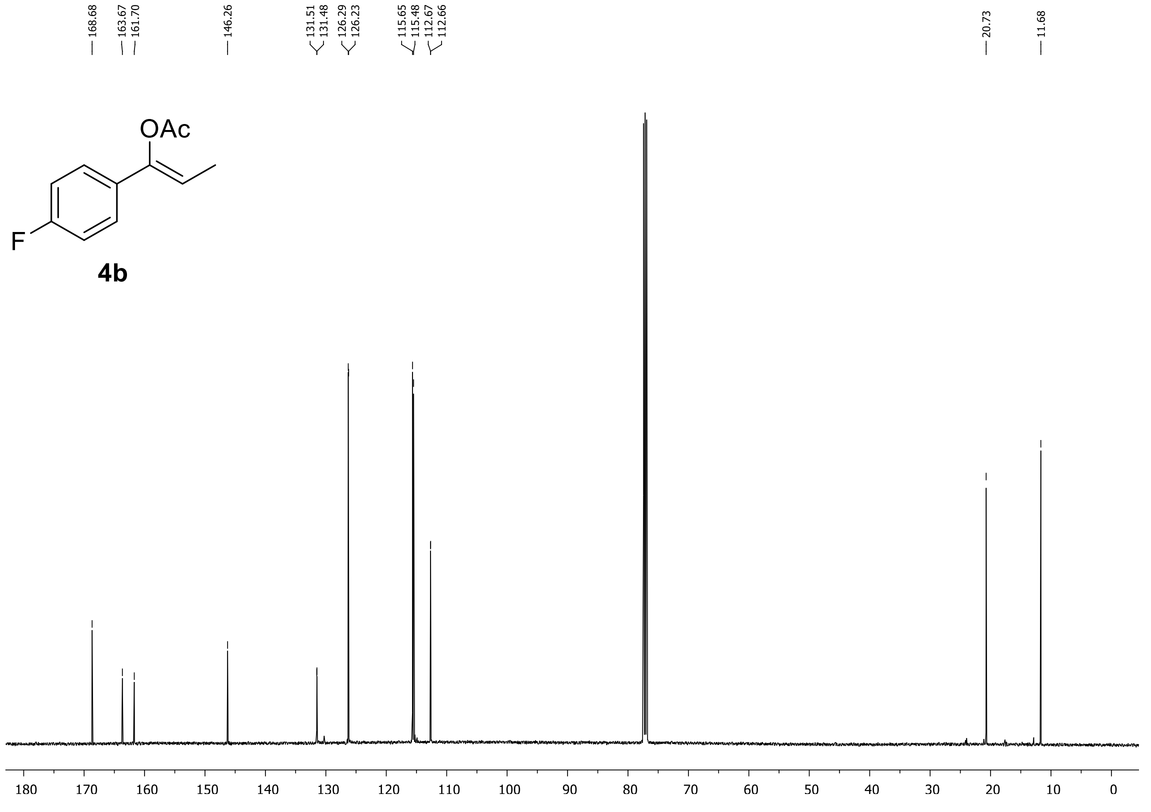

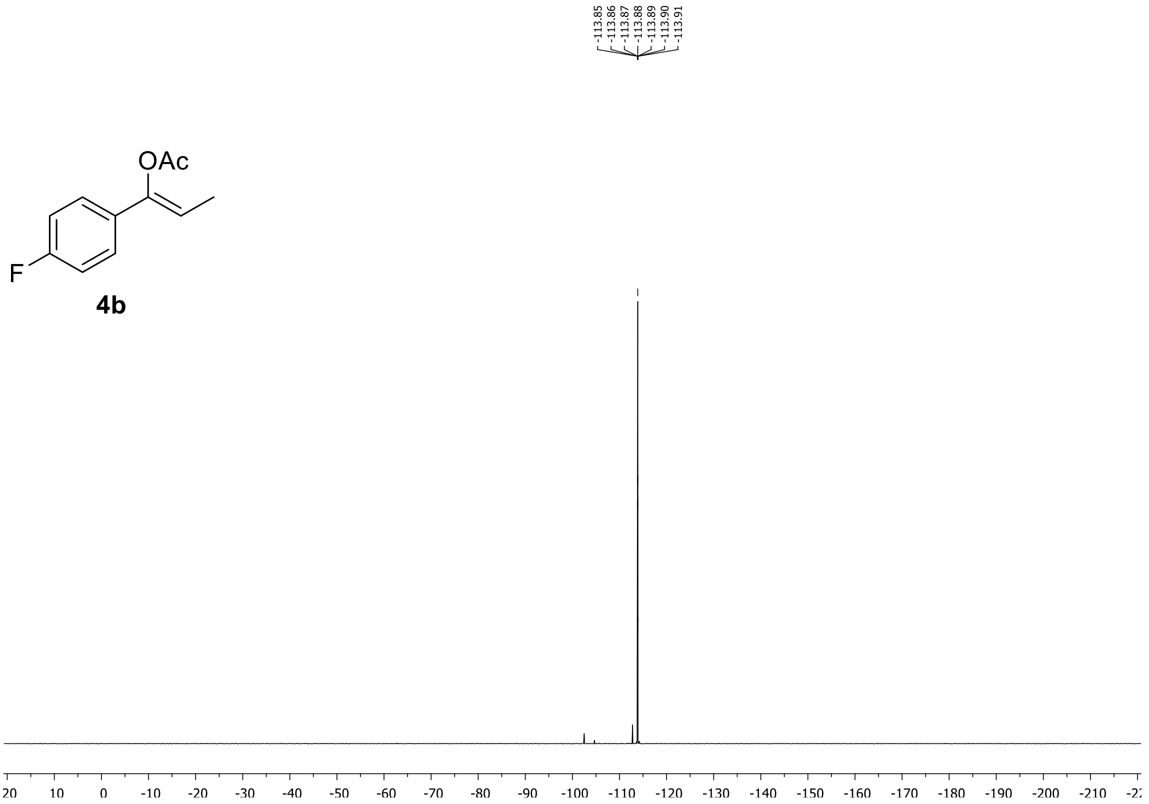

(*Z*)-1-(3-Fluorophenyl)prop-1-en-1-yl acetate (4c)

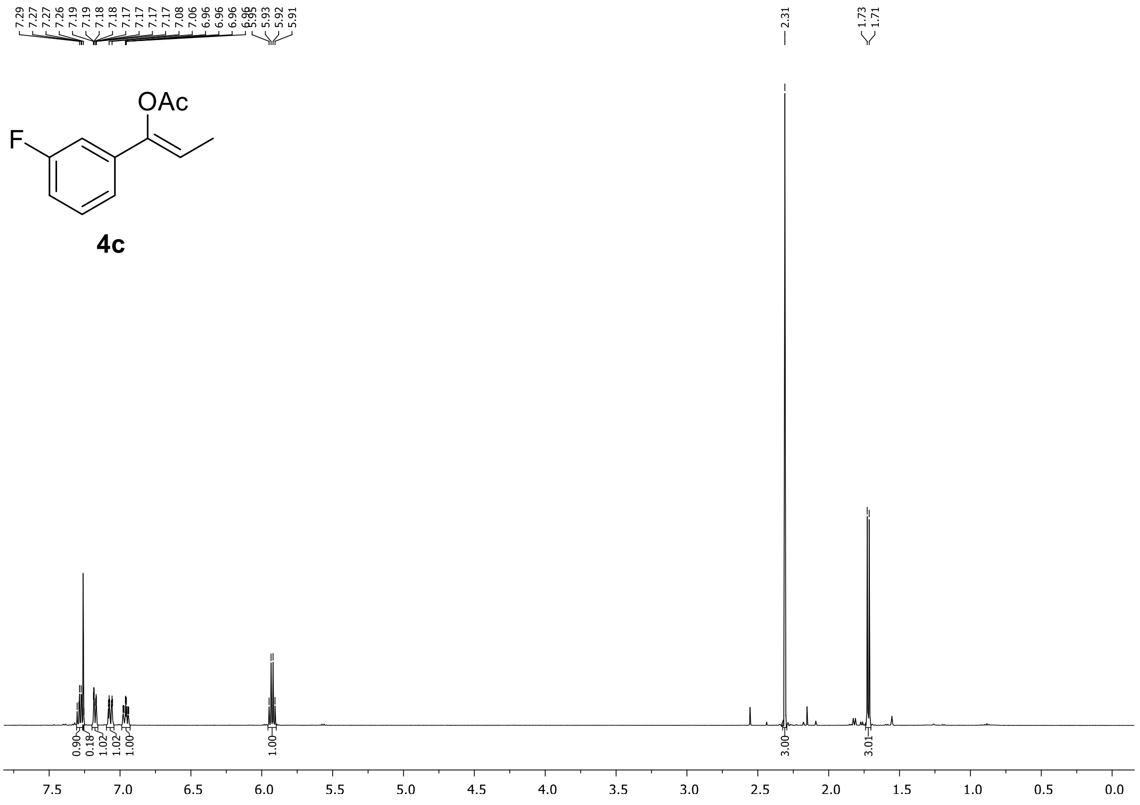

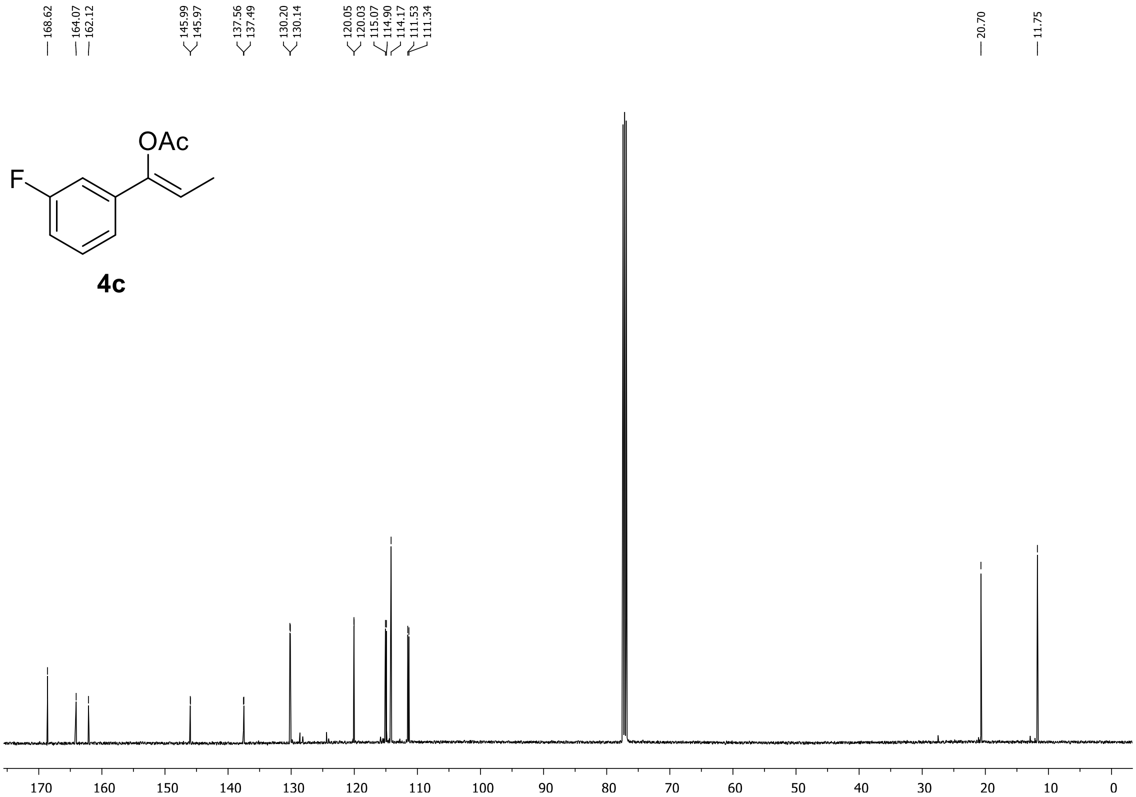
168.62
164.07
162.12
145.99
145.97
137.56
137.49
130.20
130.14
120.05
120.03
115.07
114.90
114.17
111.53
111.34
20.70
11.75
OAc
F
4c
170 160 150 140 130 120 110 100 90 80 70 60 50 40 30 20 10 0

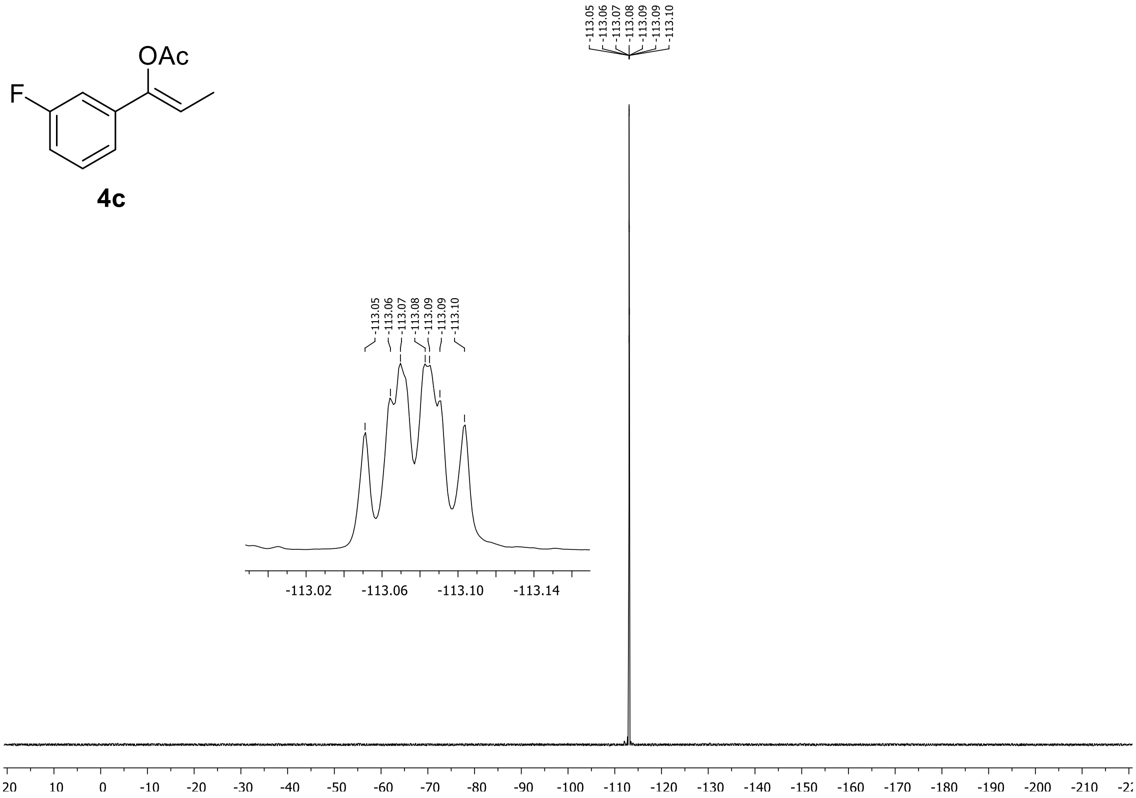
-113.05
-113.06
-113.07
-113.08
-113.09
-113.09
-113.10
OAc
F
4c
-113.02 -113.06 -113.10 -113.14
20 10 0 -10 -20 -30 -40 -50 -60 -70 -80 -90 -100 -110 -120 -130 -140 -150 -160 -170 -180 -190 -200 -210

(*Z*)-1-(4-Bromophenyl)prop-1-en-1-yl acetate (4d)

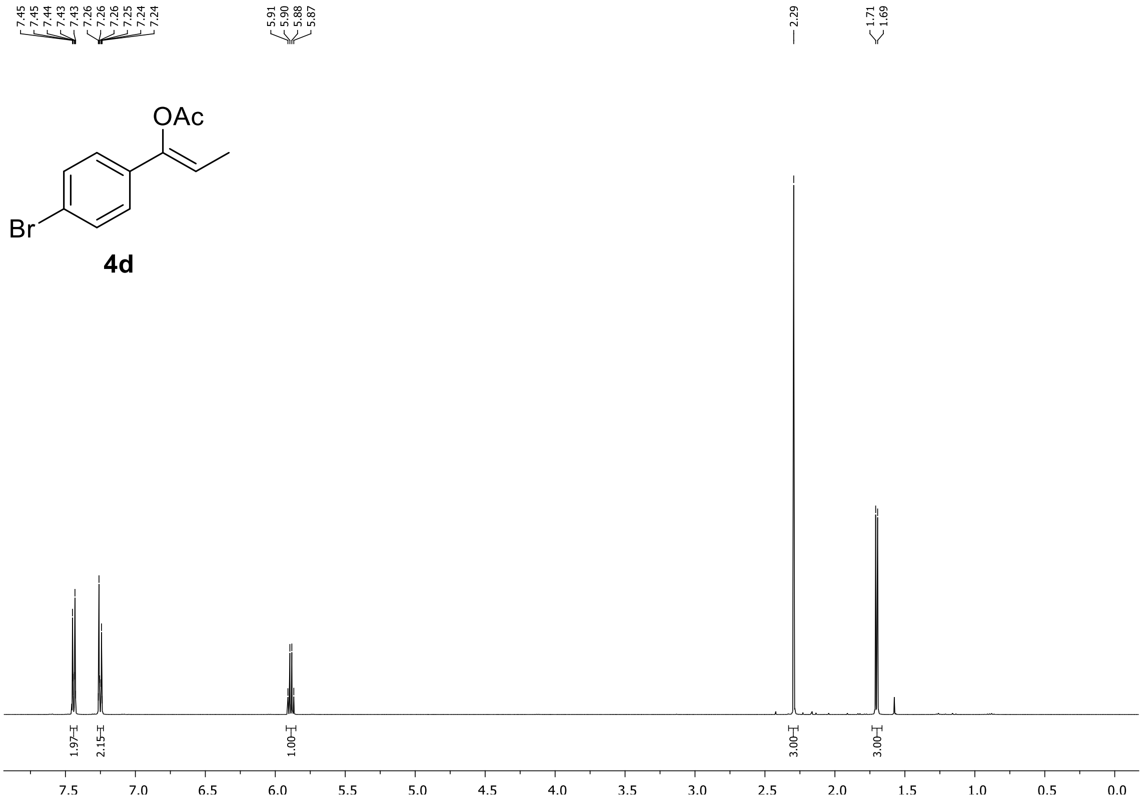

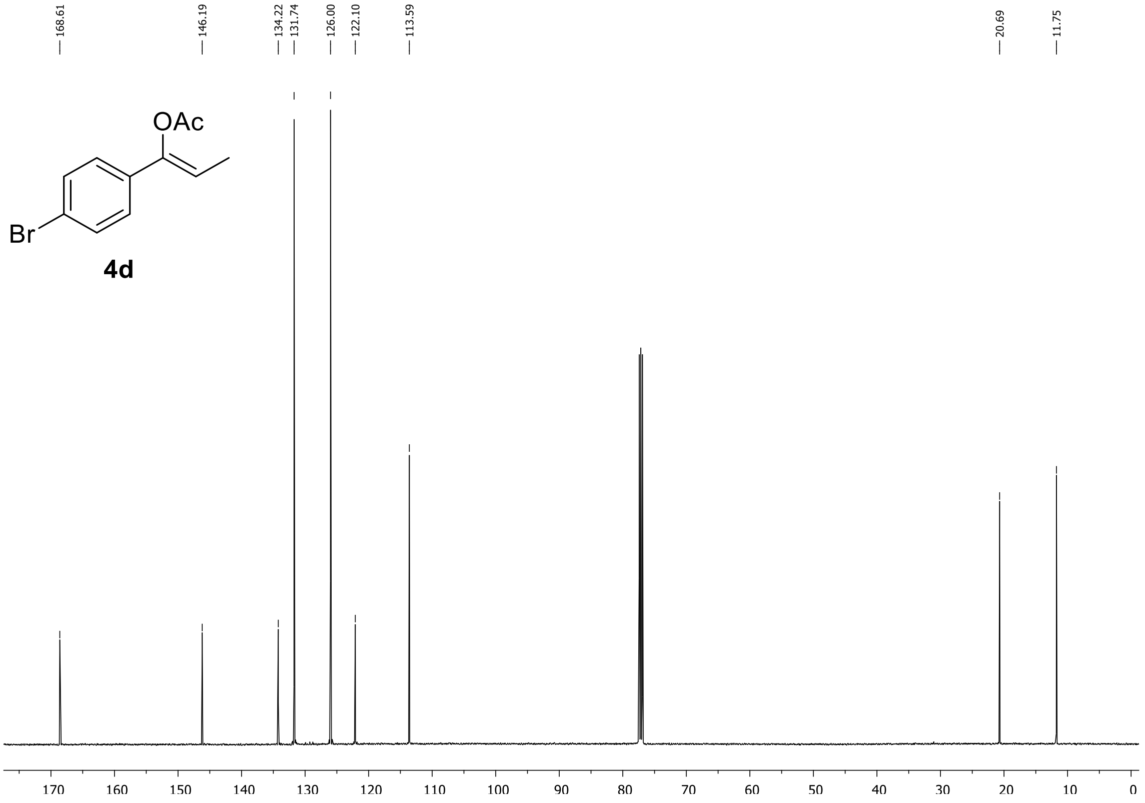

(*Z*)-1-(3-(Trifluoromethyl)phenyl)prop-1-en-1-yl acetate (4e)

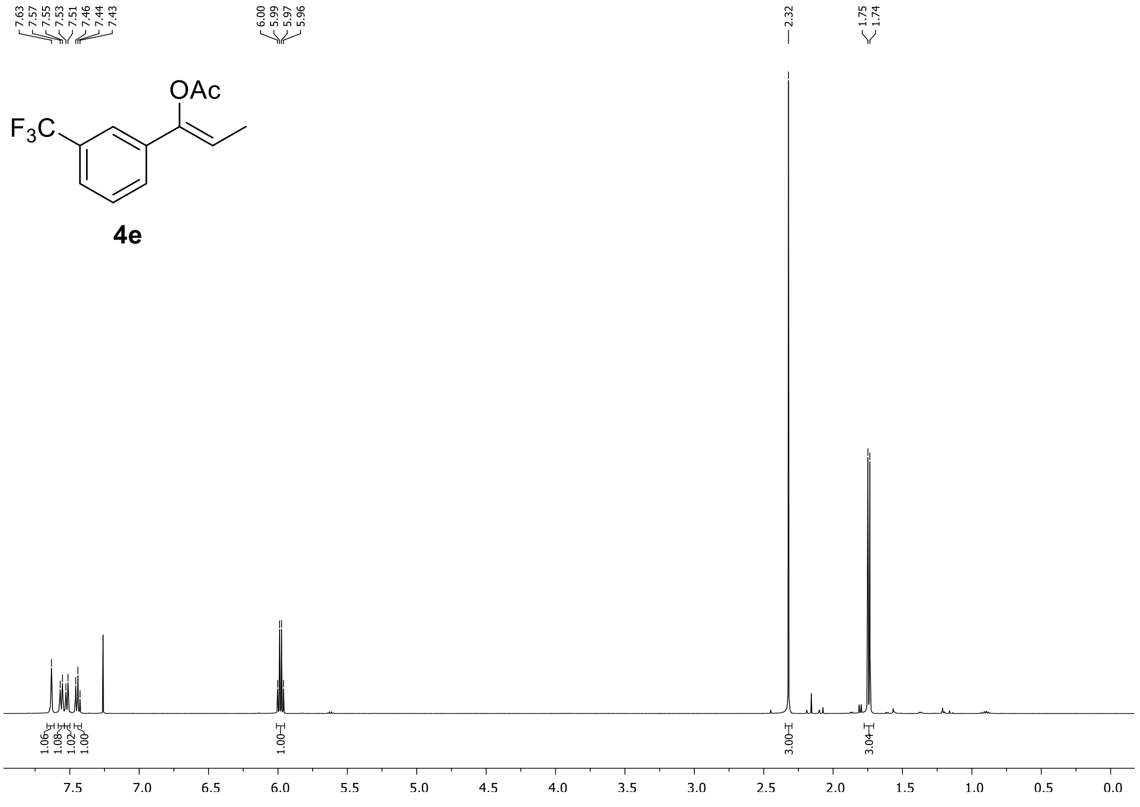

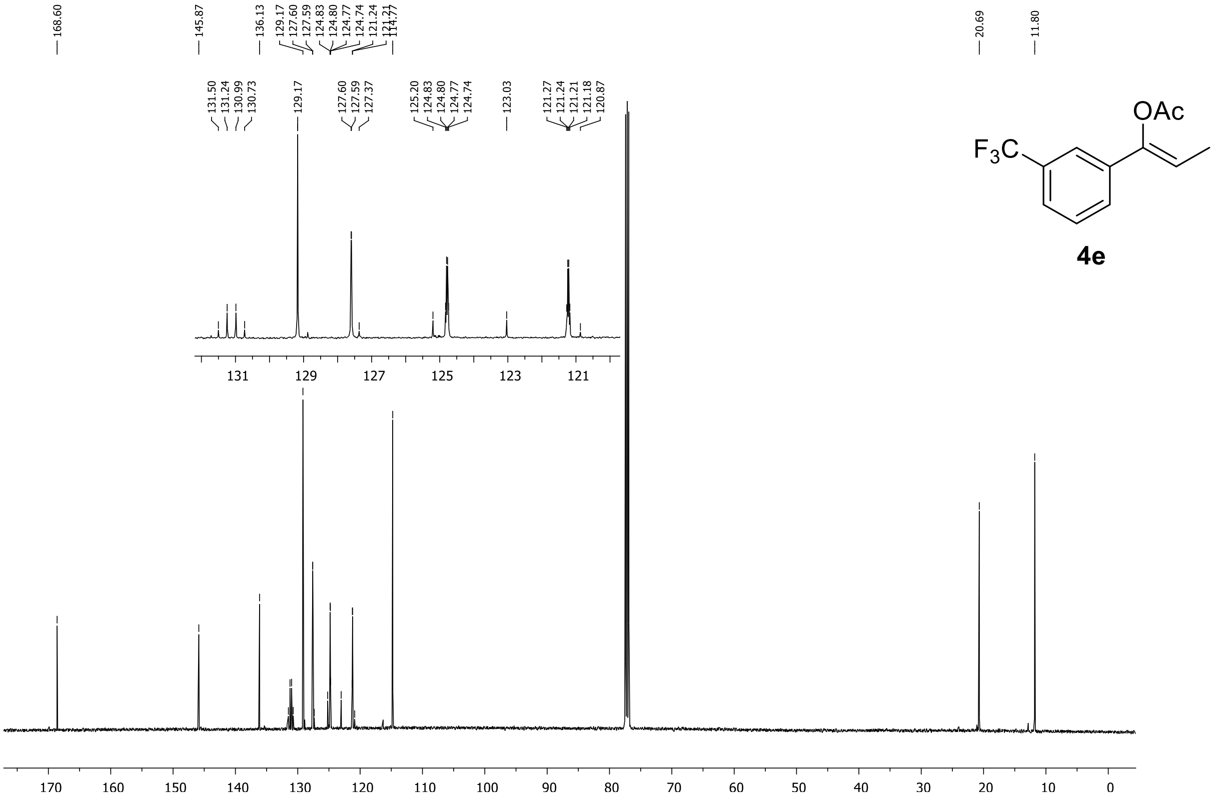

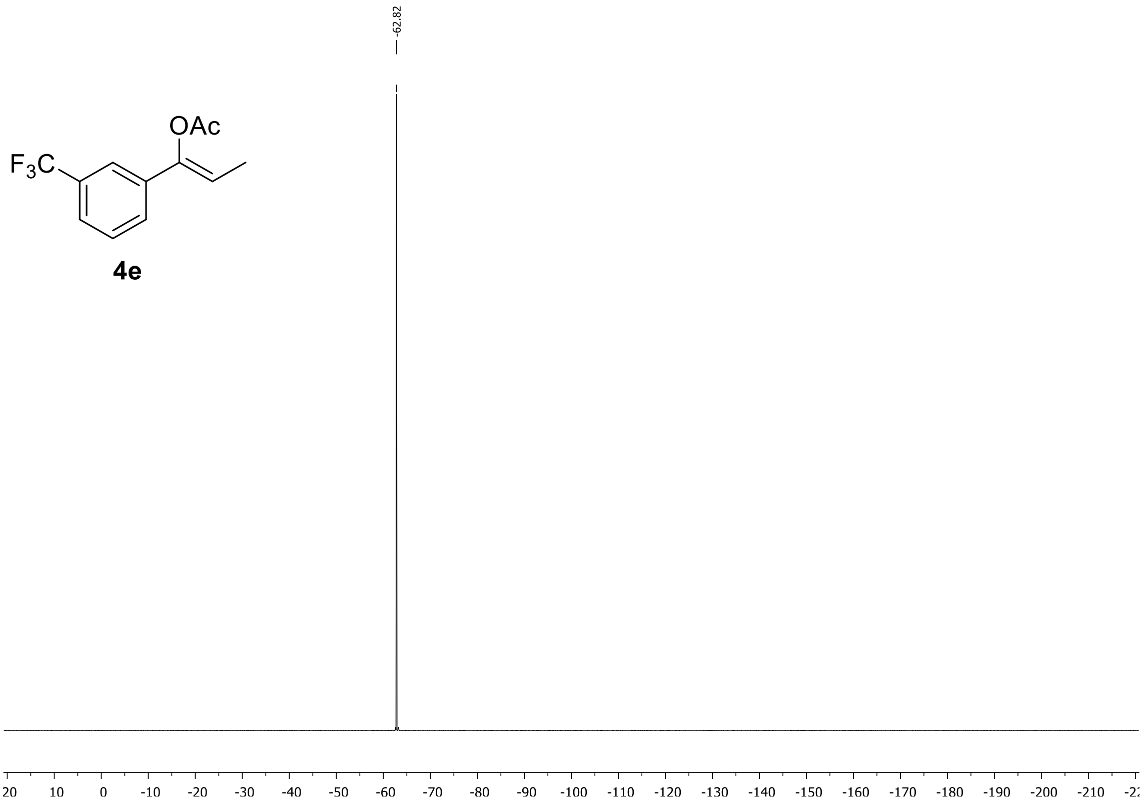

1-(2-(Trifluoromethyl)phenyl)prop-1-en-1-yl acetate (4f)

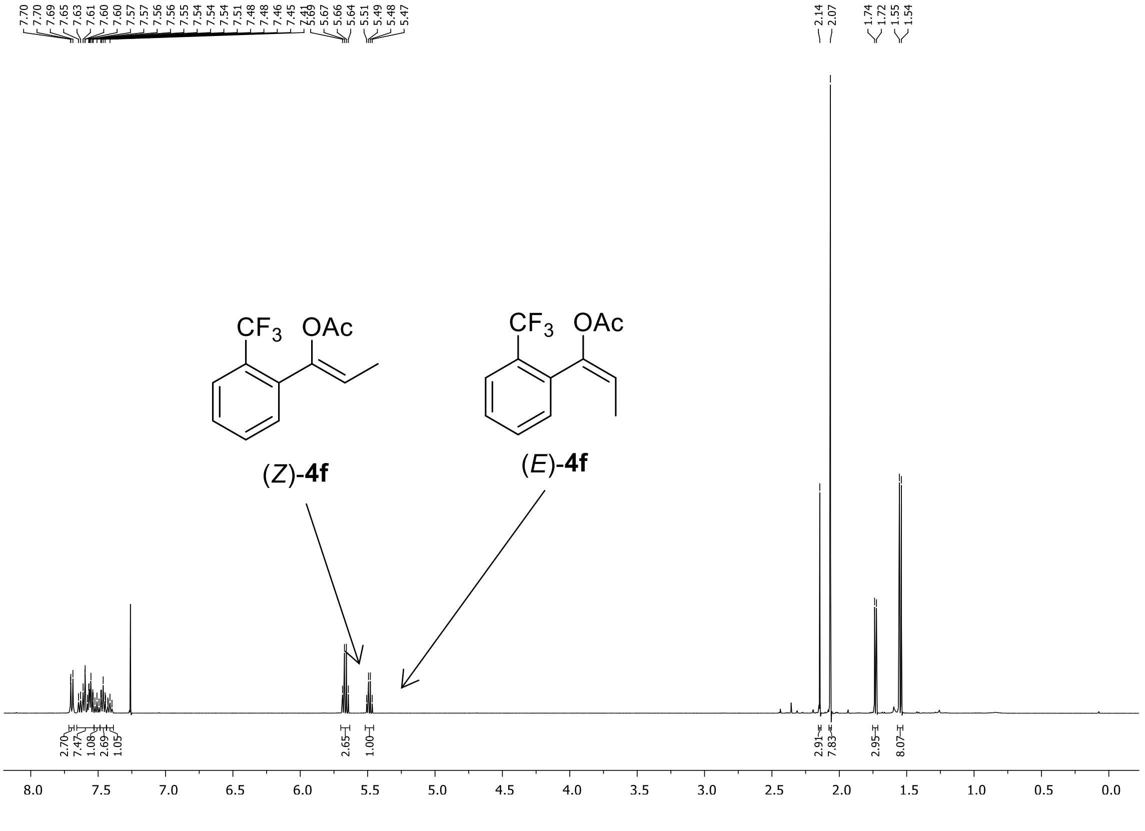

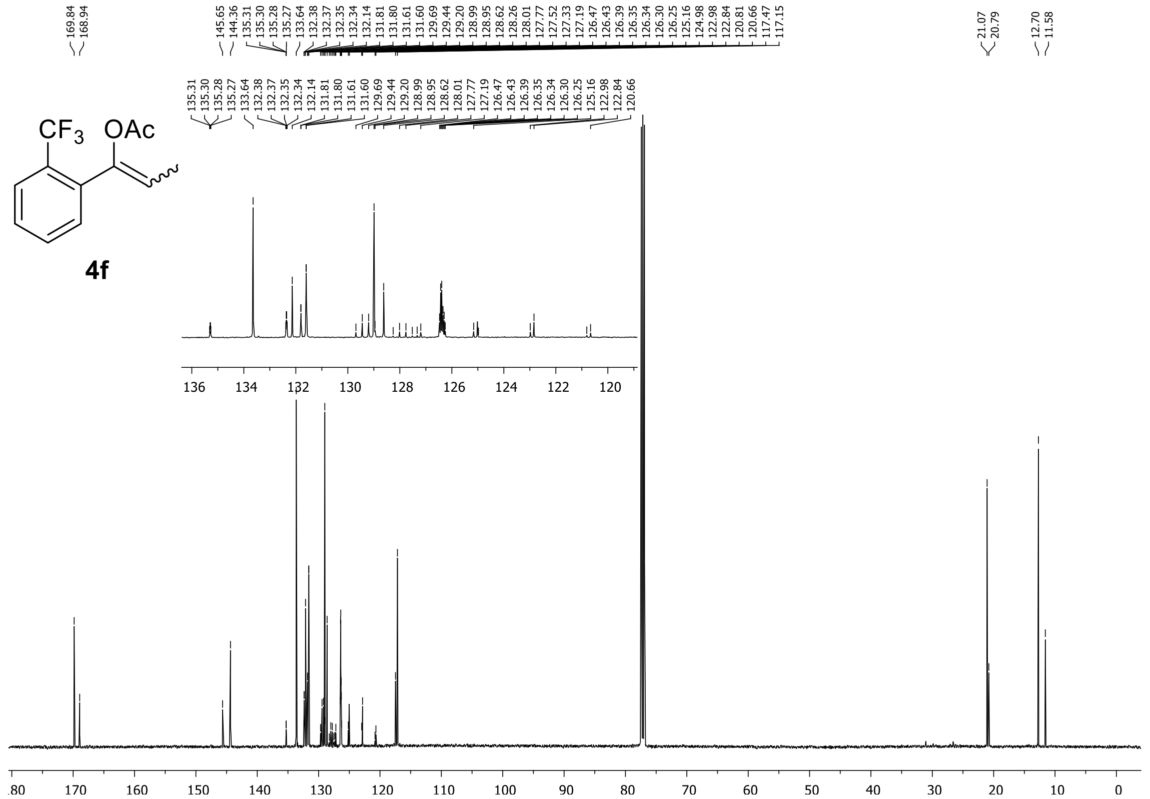
169.84
168.94
145.65
144.36
135.31
135.30
135.28
135.27
133.64
132.38
132.37
132.35
132.34
132.14
131.81
131.80
131.61
131.60
129.69
129.44
129.20
128.99
128.95
128.62
128.26
128.01
127.77
127.52
127.33
127.19
126.47
126.43
126.39
126.35
126.34
126.30
126.25
125.16
124.98
122.98
122.84
120.81
120.66
117.47
117.15
21.07
20.79
12.70
11.58
CF3 OAc
4f

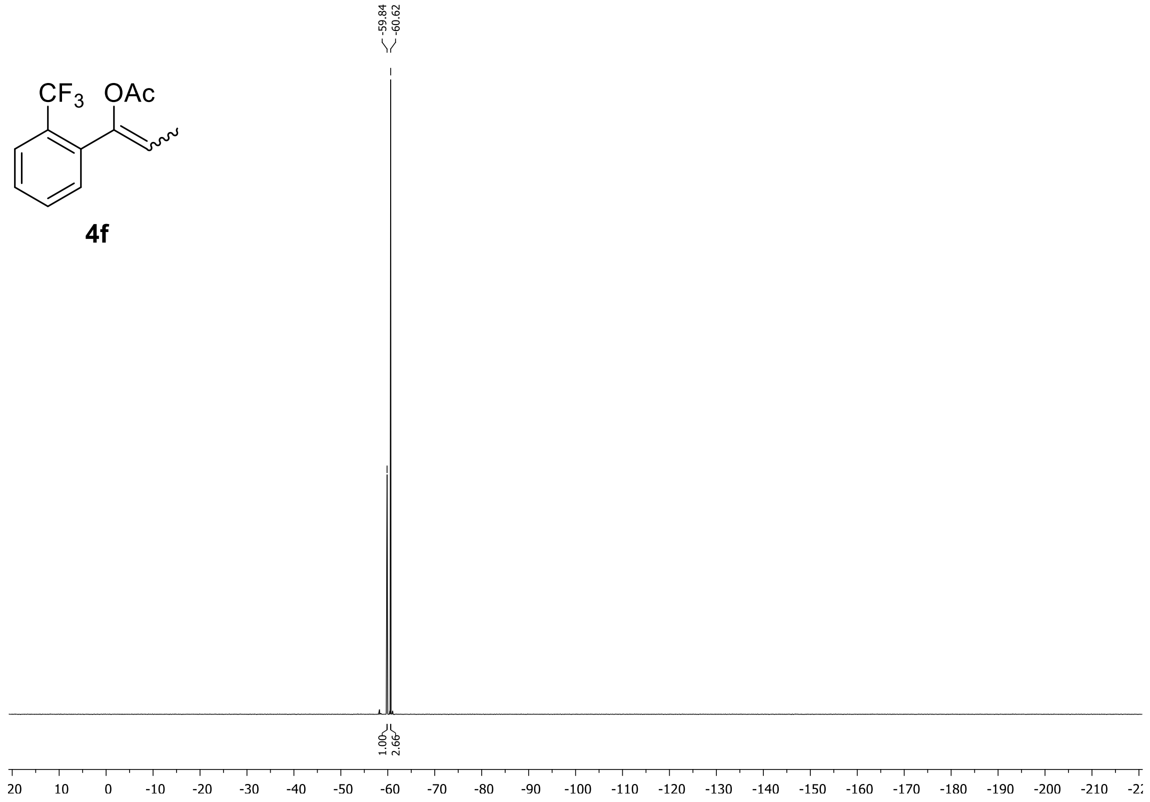
-59.84
-60.62
CF3 OAc
4f
1.00
2.66

(*Z*)-1-(3-Nitrophenyl)prop-1-en-1-yl acetate (4g)

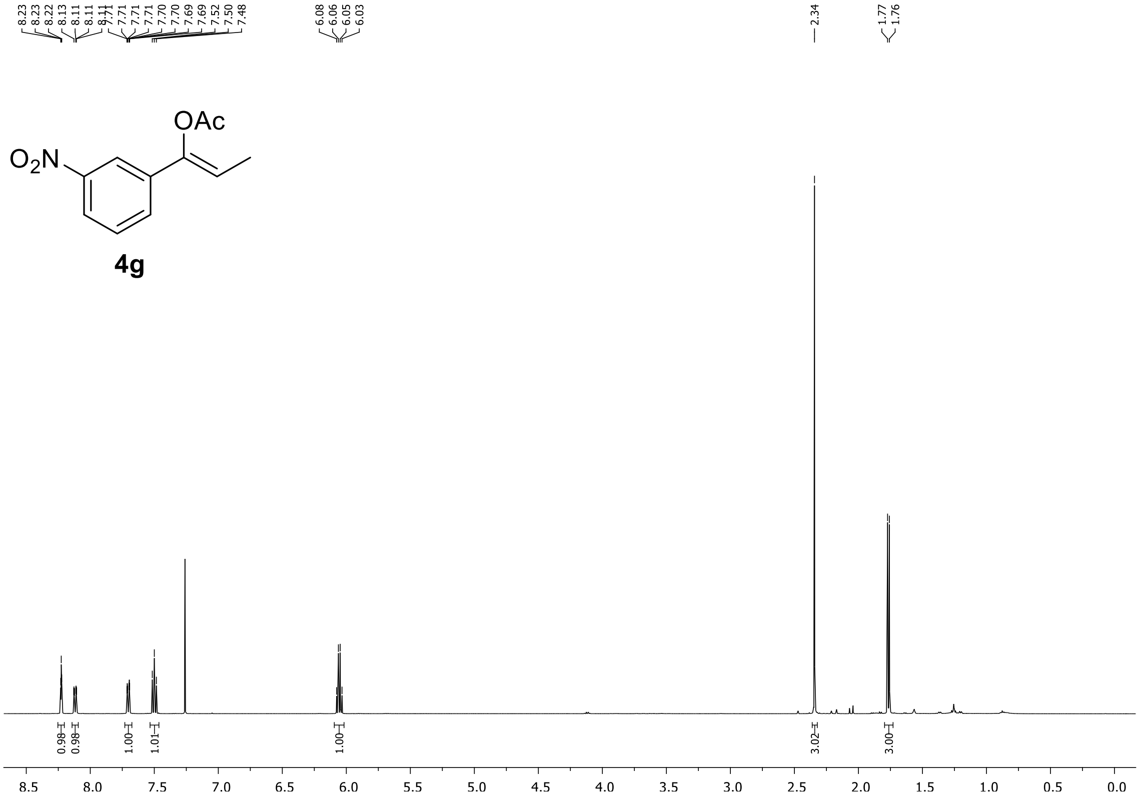

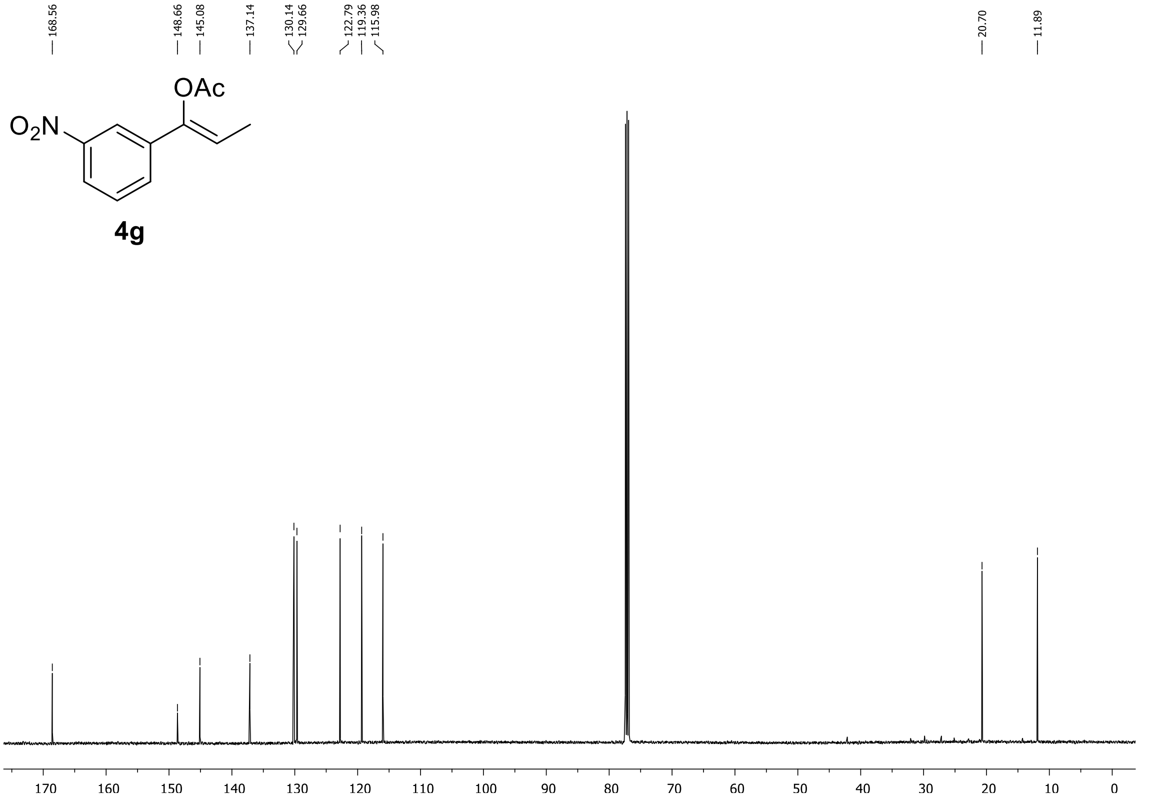

(*Z*)-1-(*p*-Tolyl)prop-1-en-1-yl acetate (4h)

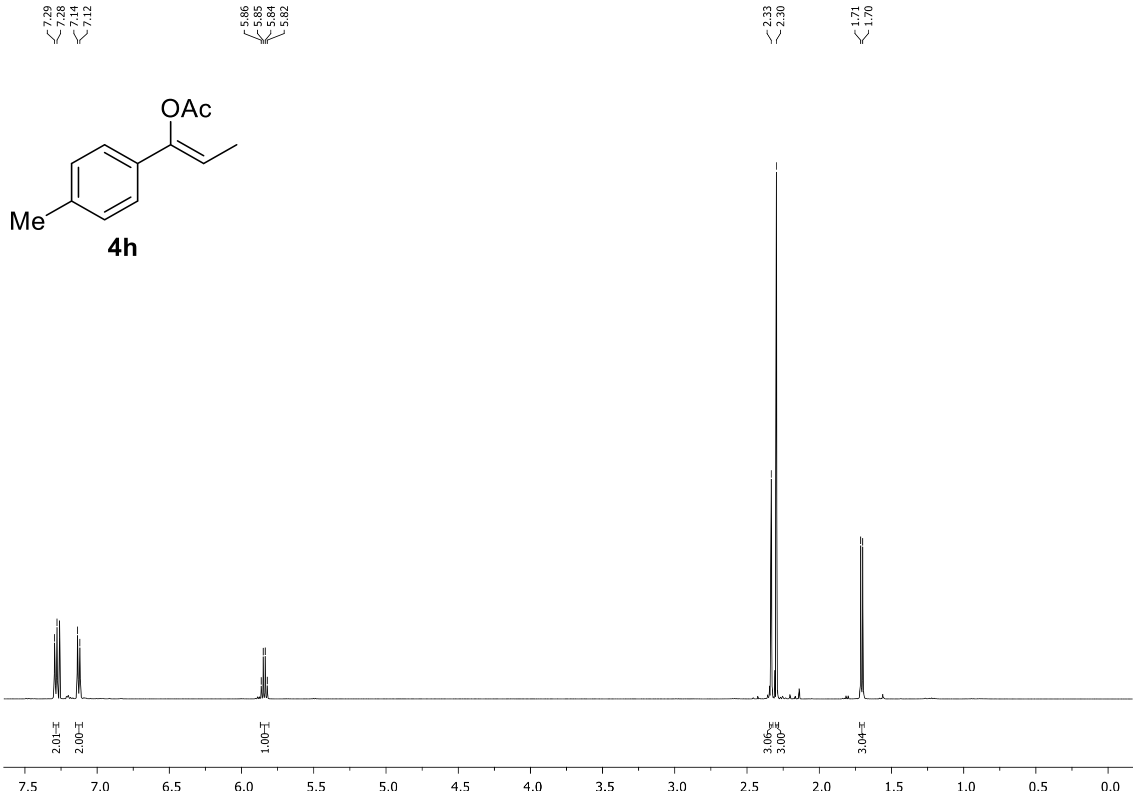

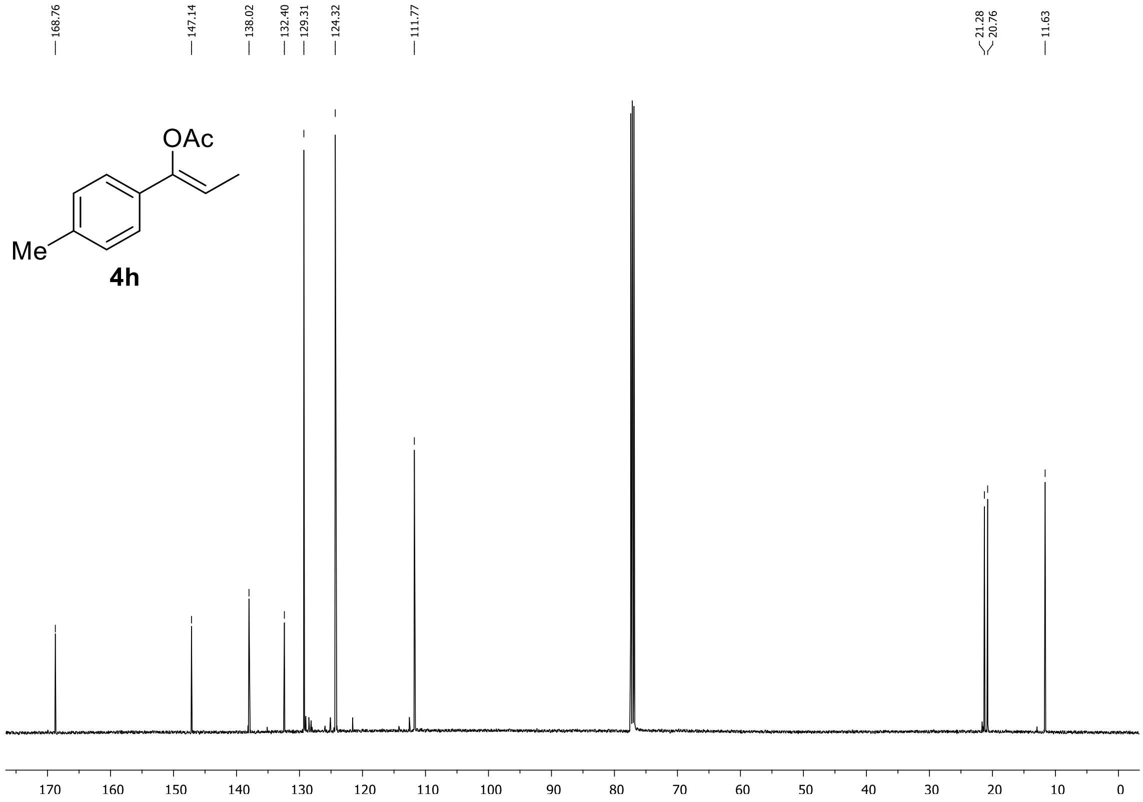

(*Z*)-1-(4-(*tert*-Butyl)phenyl)prop-1-en-1-yl acetate (4i)

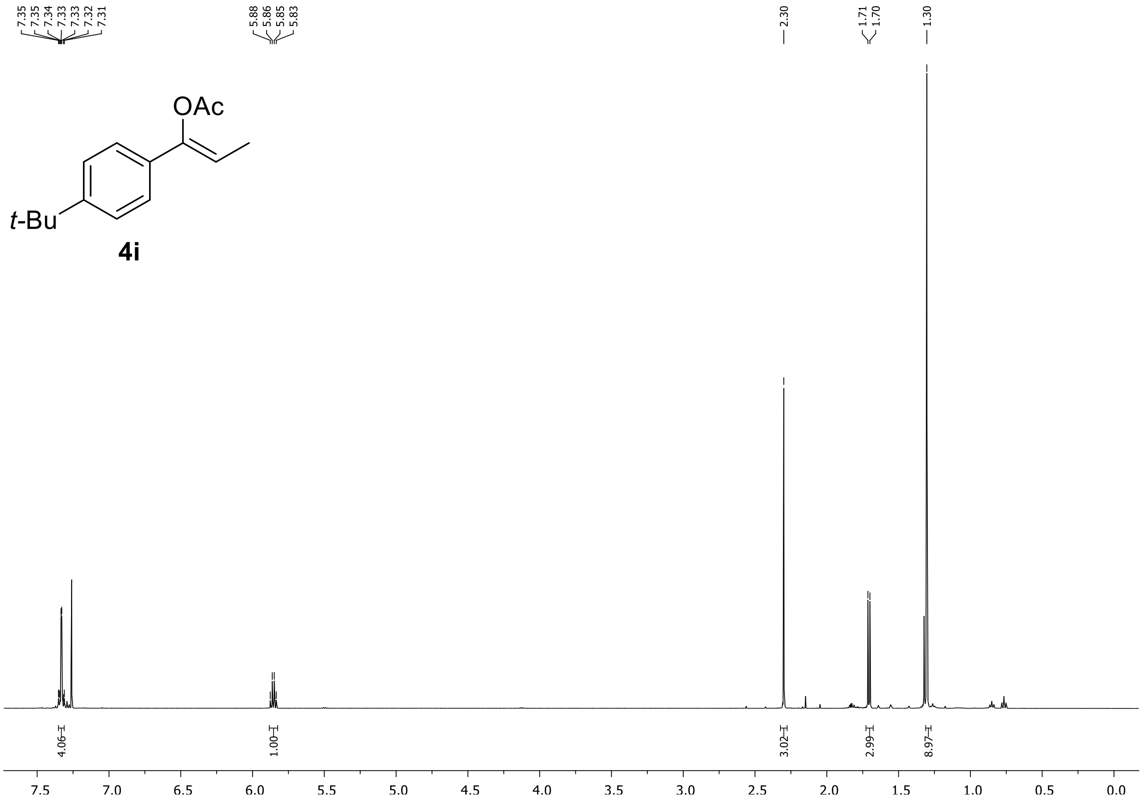

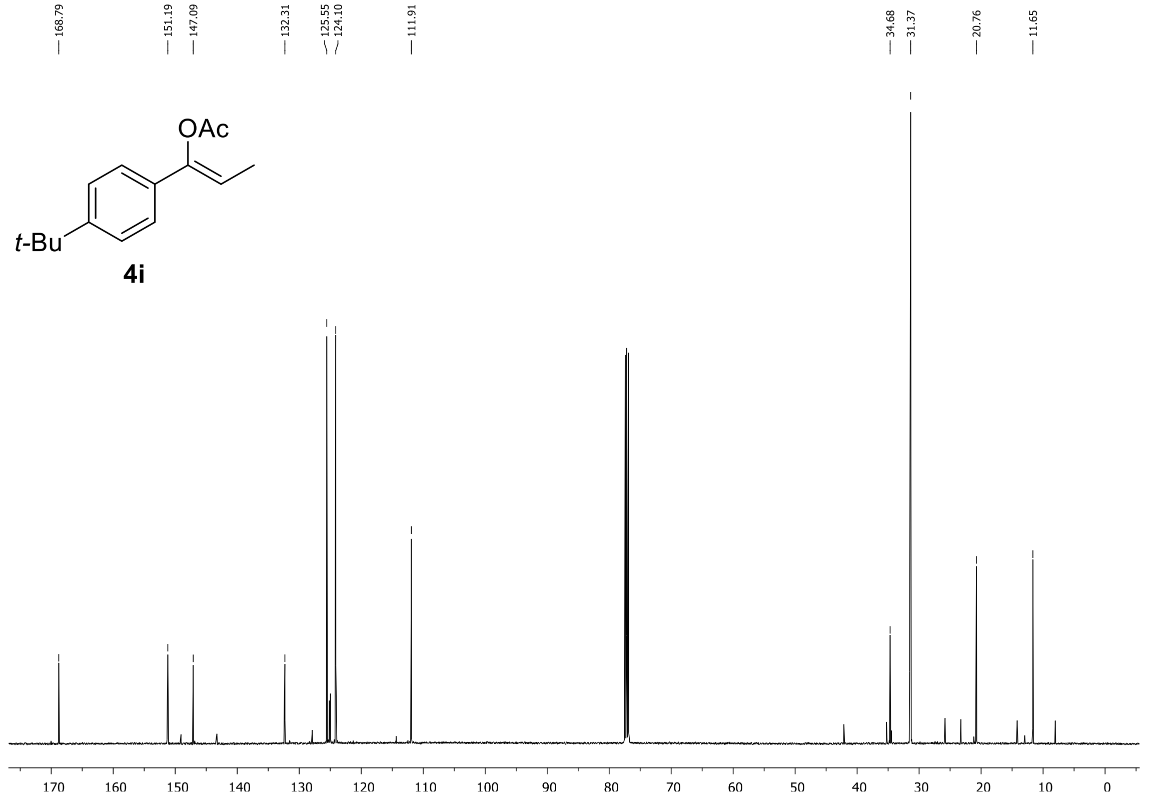

(*Z*)-1-([1,1'-Biphenyl]-4-yl)prop-1-en-1-yl acetate (4j)

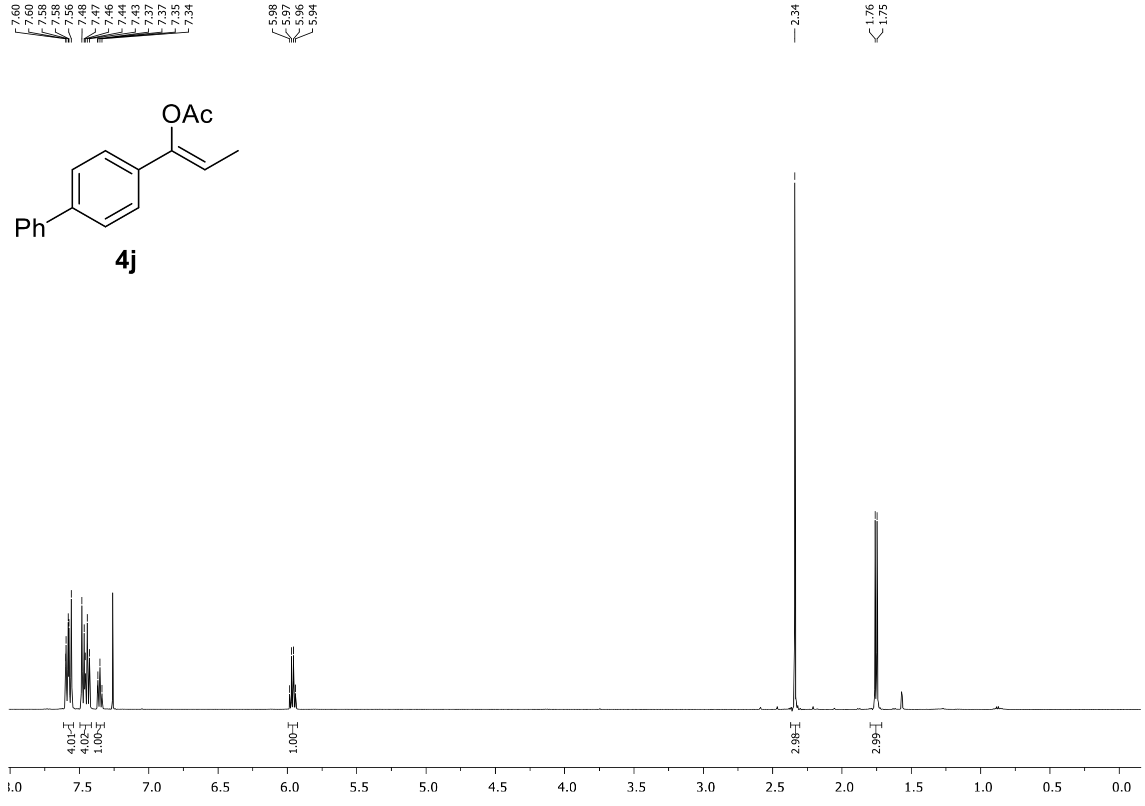

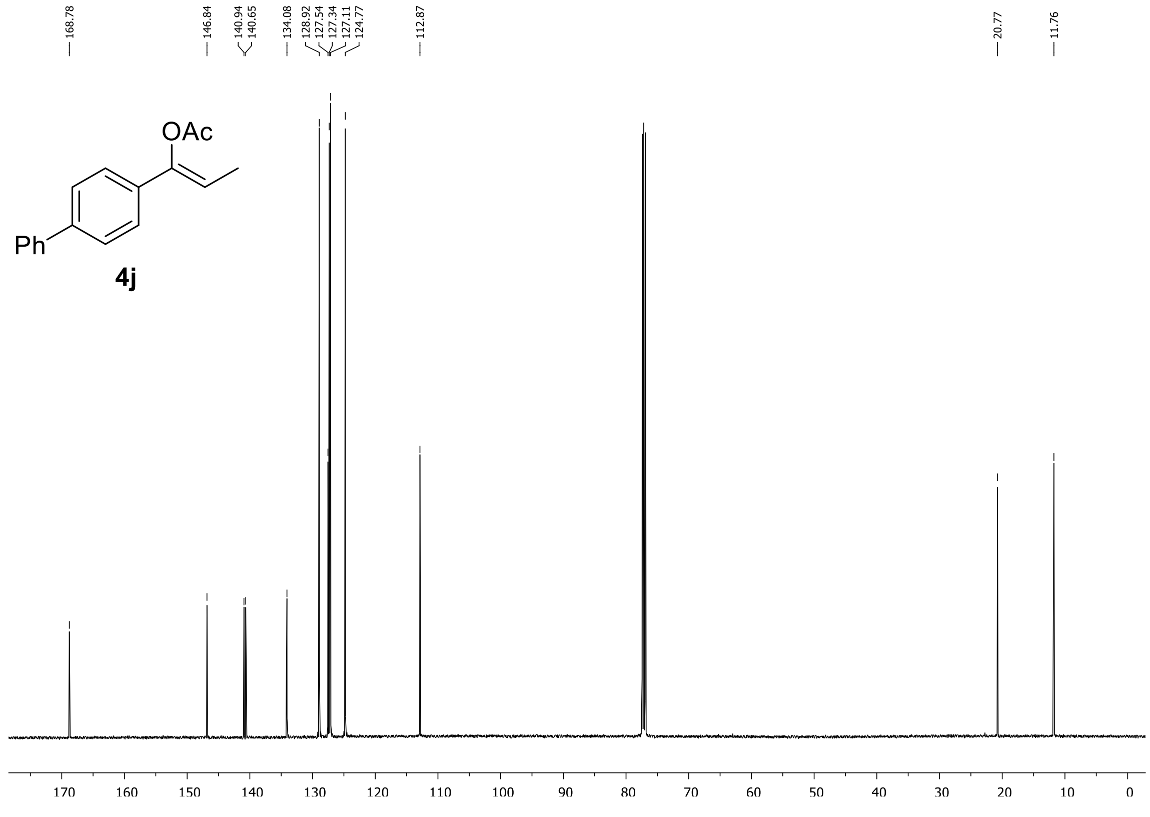

(*Z*)-1-(4-Methoxyphenyl)prop-1-en-1-yl acetate (4k)

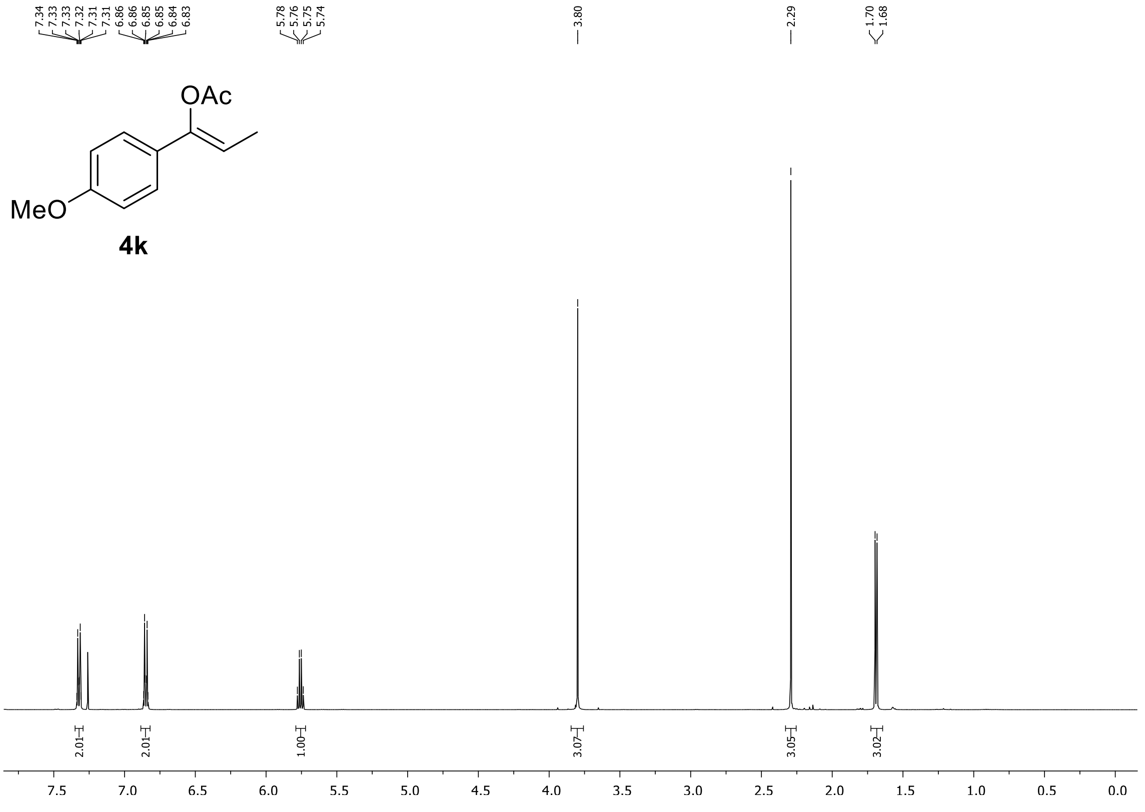

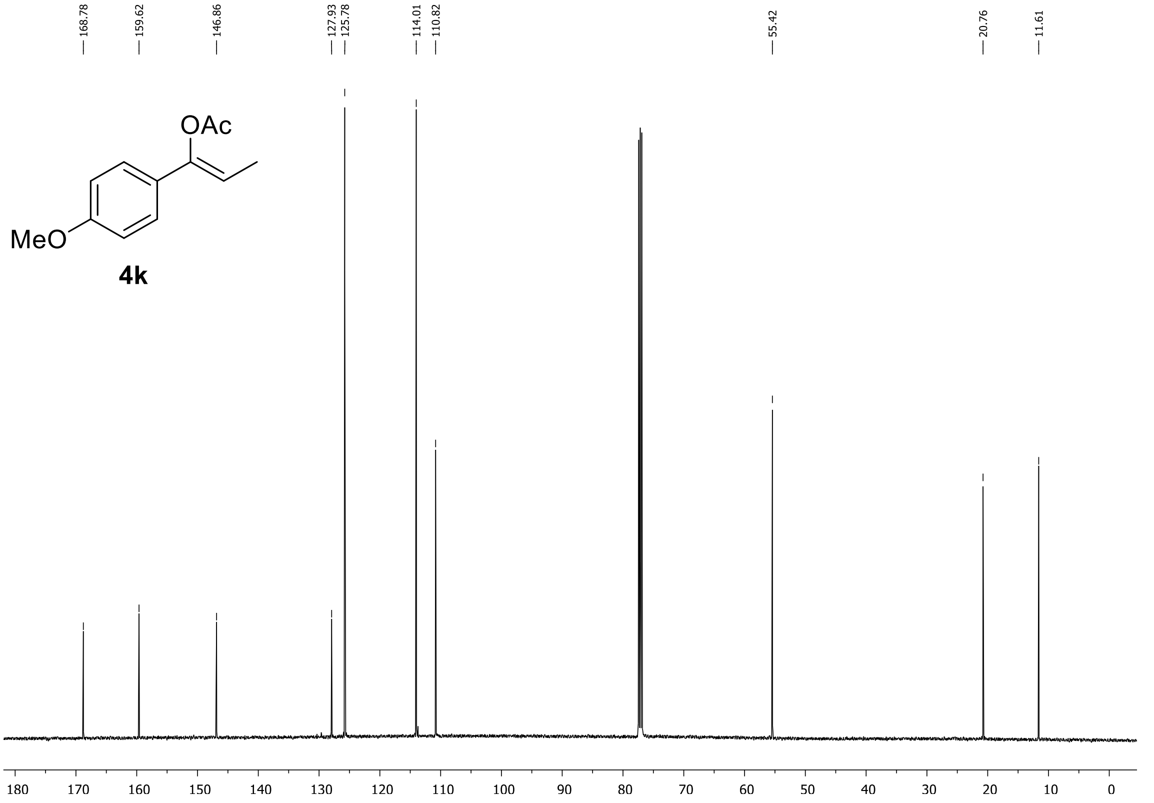

(*Z*)-1,2-Diphenylvinyl acetate (4l)

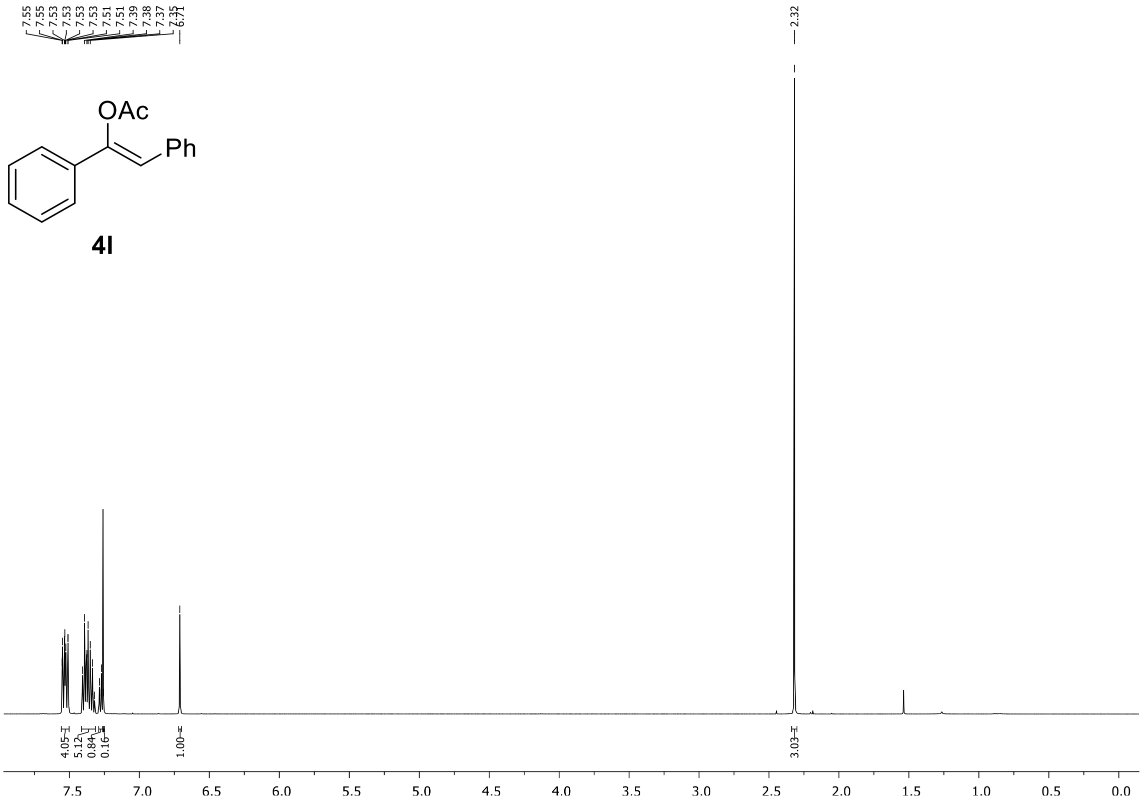

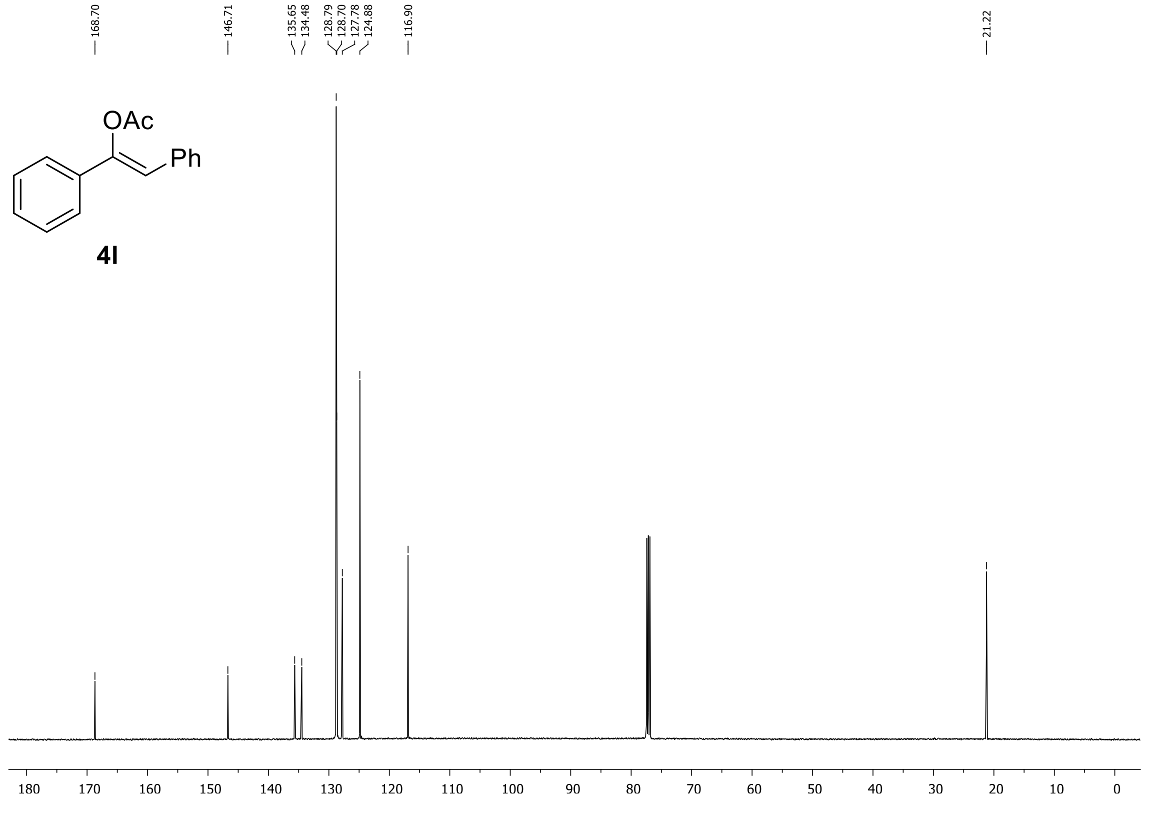

(*Z*)-3-Methyl-1-phenylbut-1-en-1-yl acetate (4m)

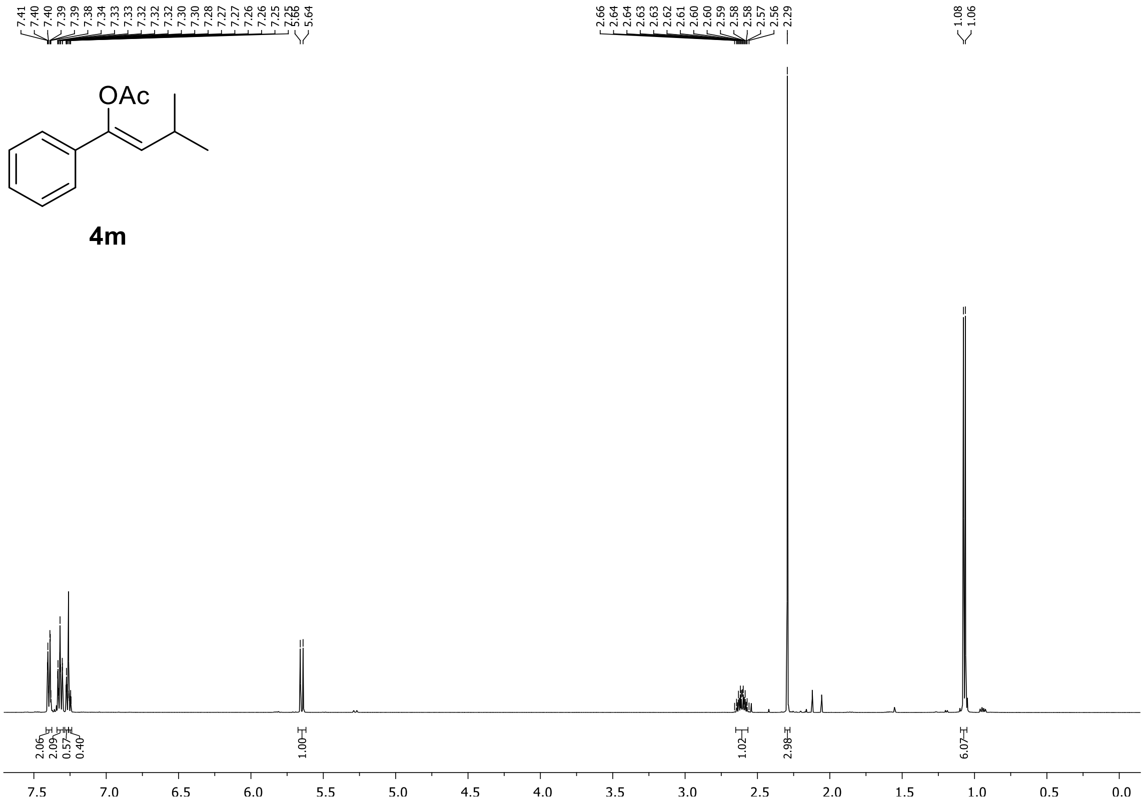

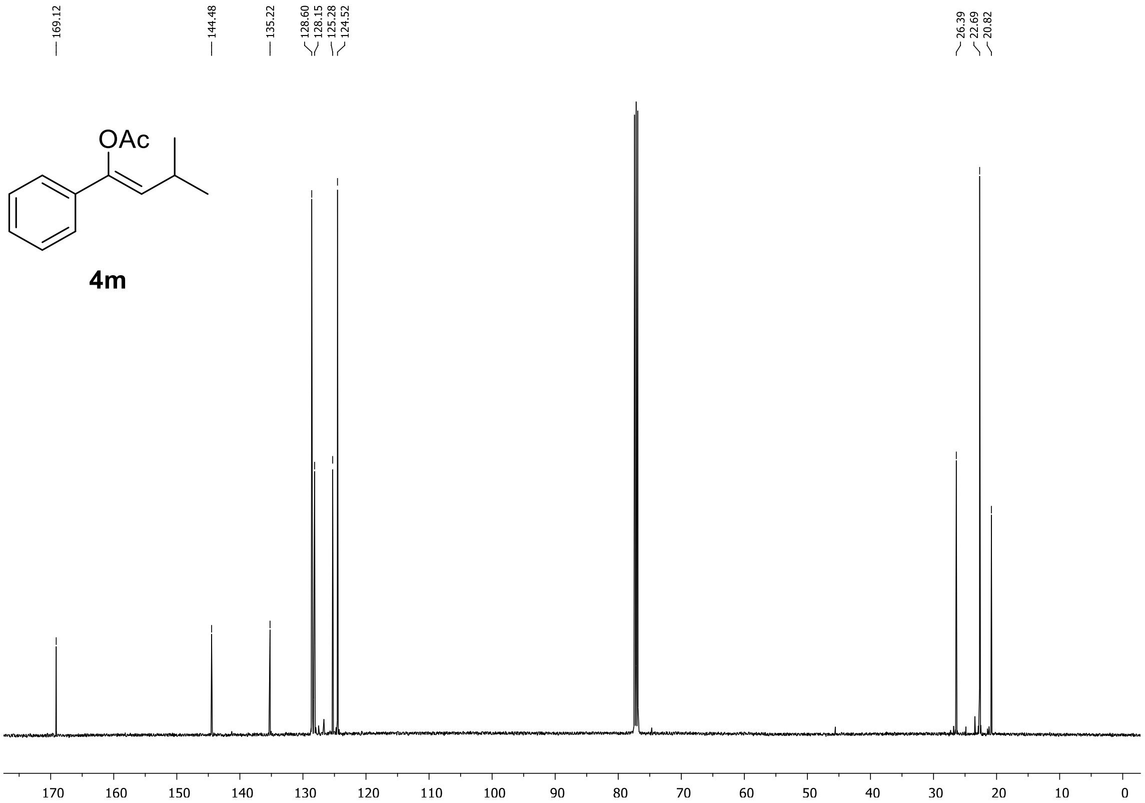

(*Z*)-1-Phenylbut-1-en-1-yl acetate (4n)

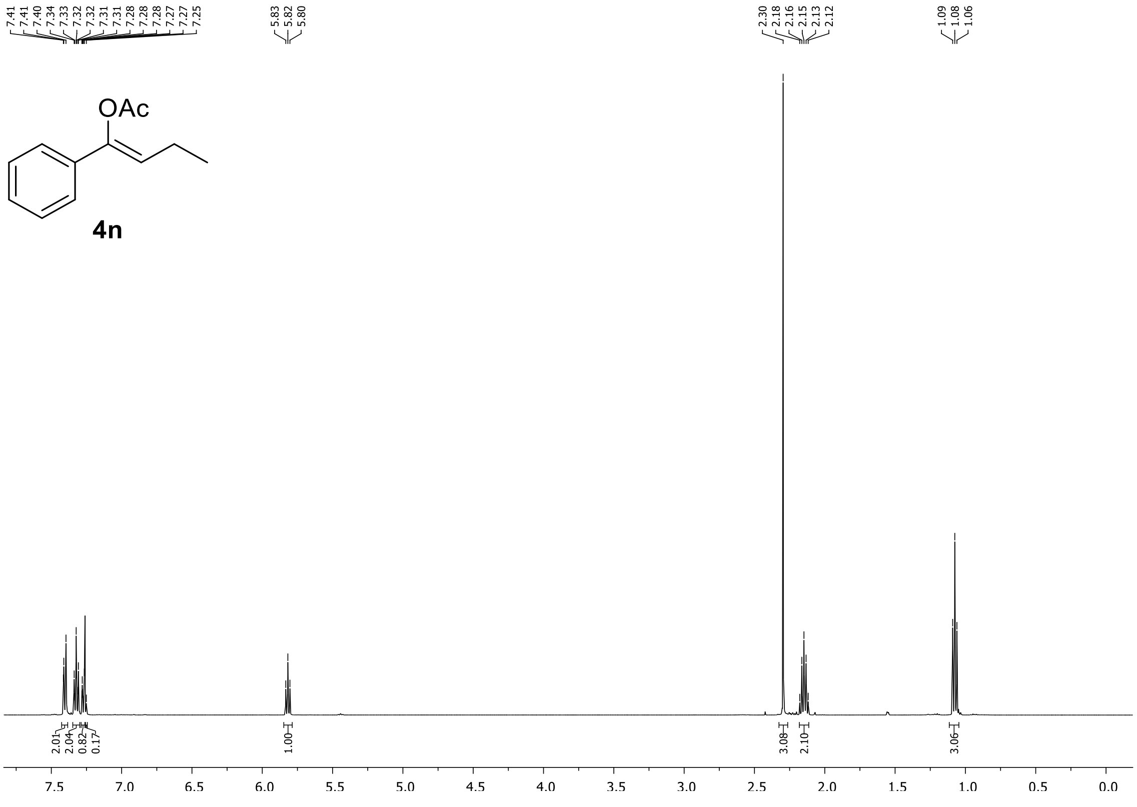

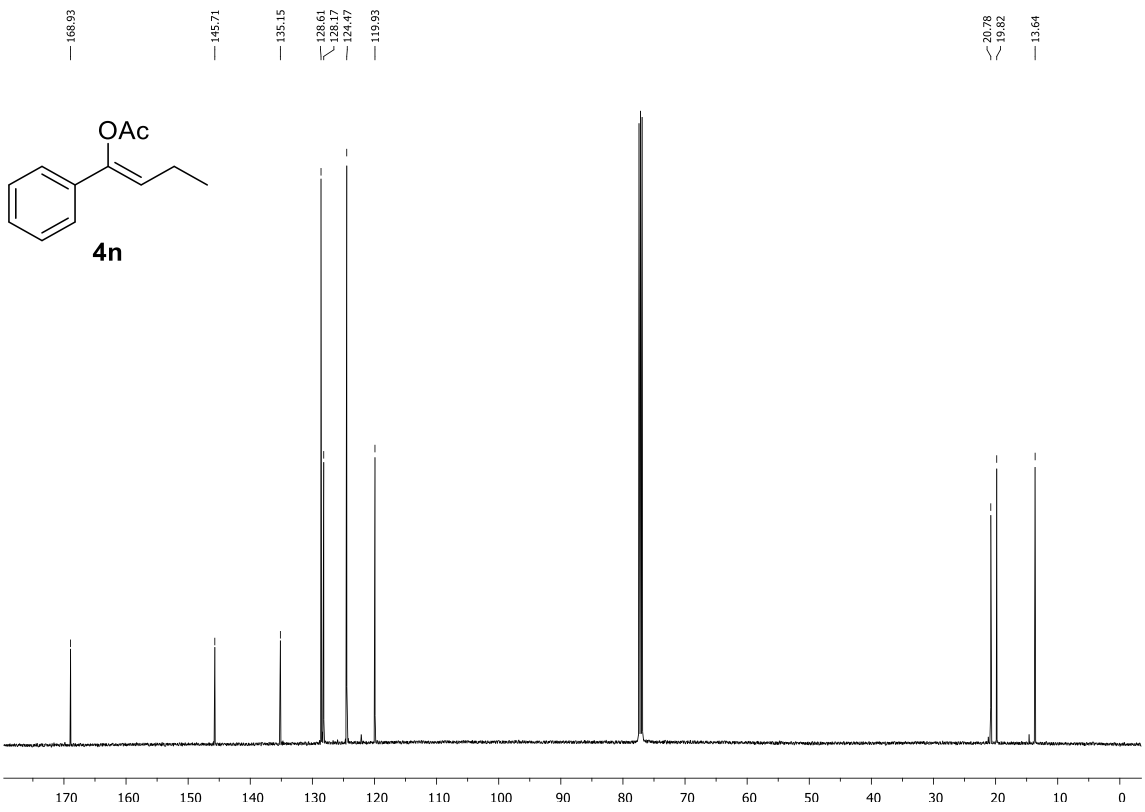

3,4-Dihydronaphthalen-1-yl acetate (4o)

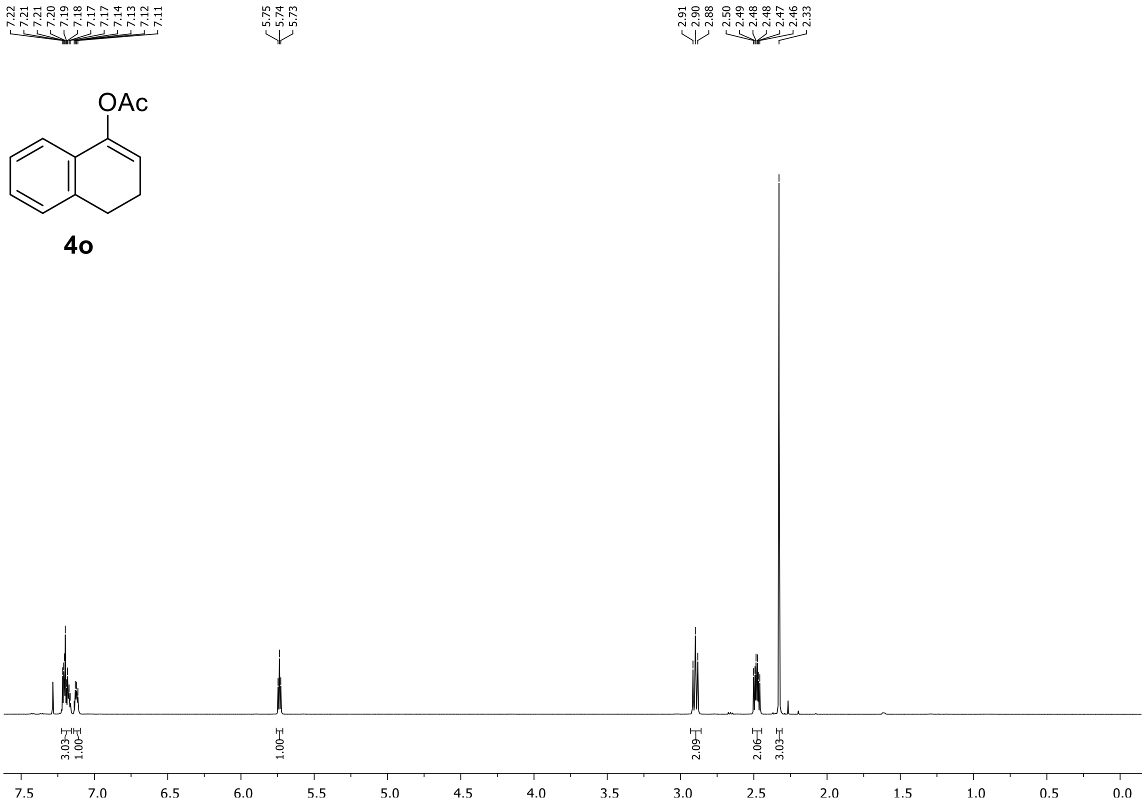

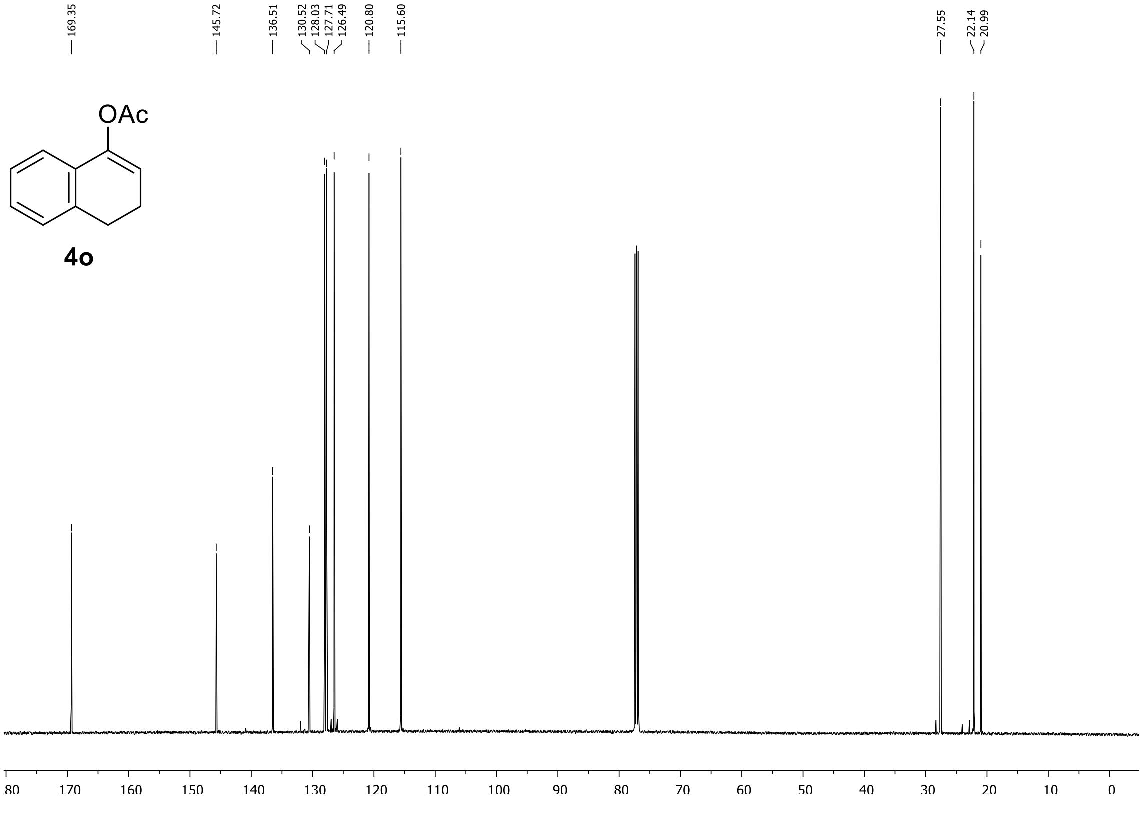

(*Z*)-1-(Thiophen-2-yl)prop-1-en-1-yl acetate (4p)

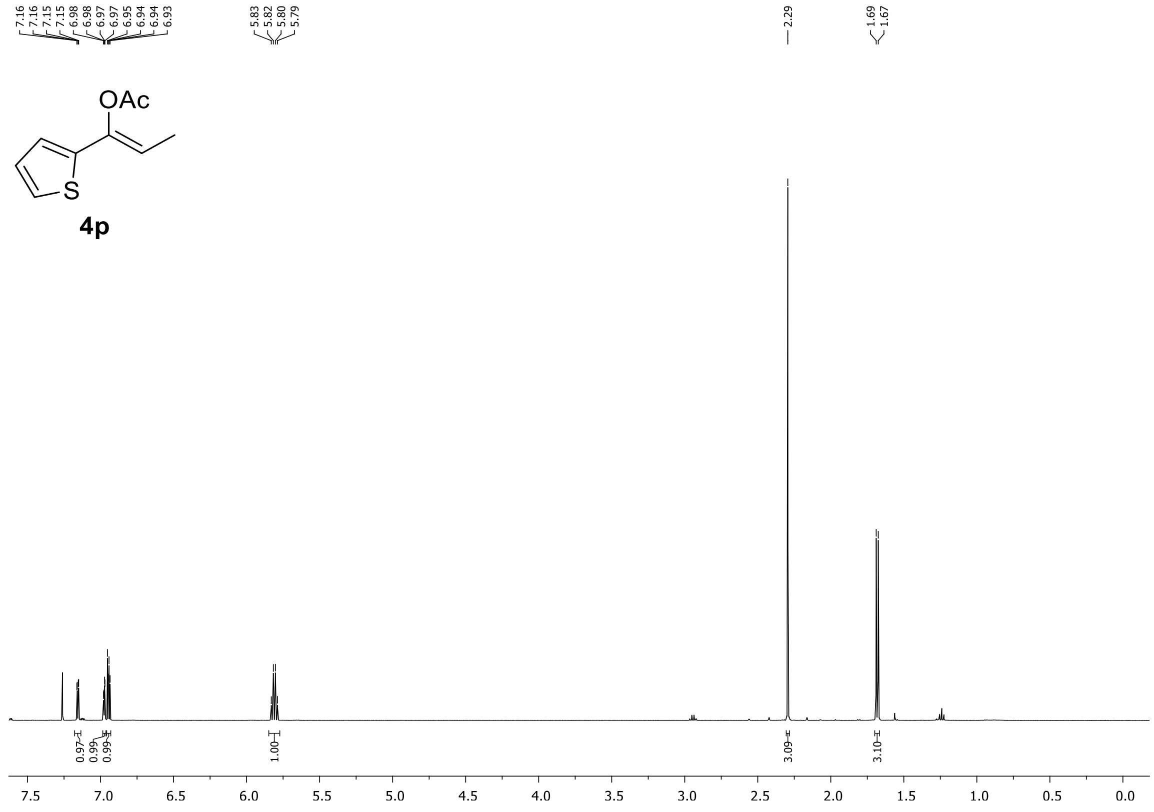

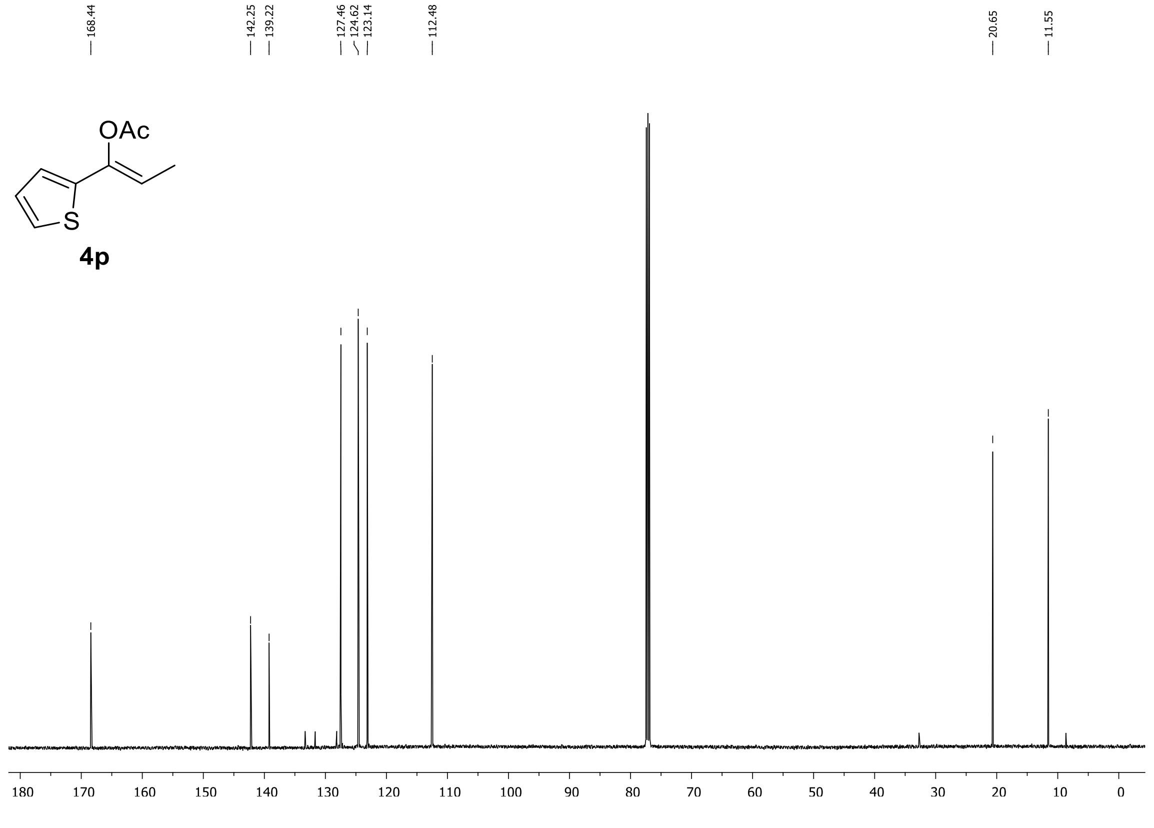

2-Cyano-1-phenylvinyl acetate (4q)

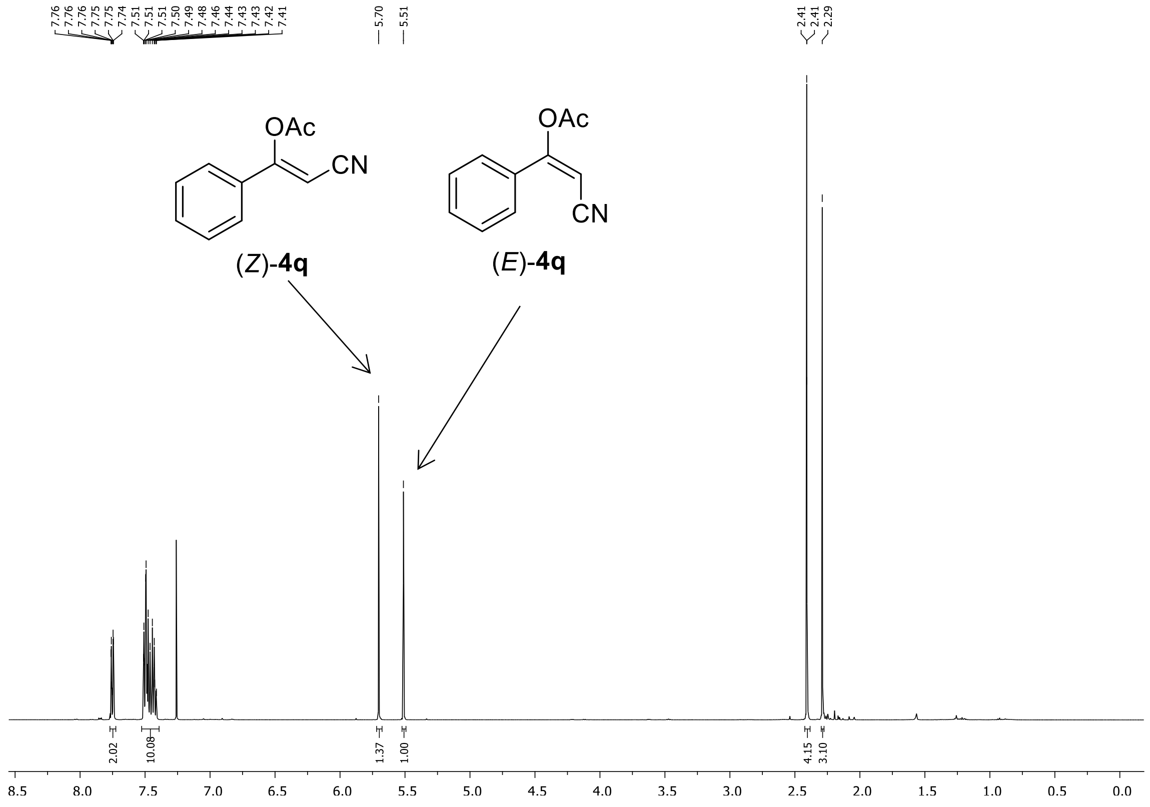

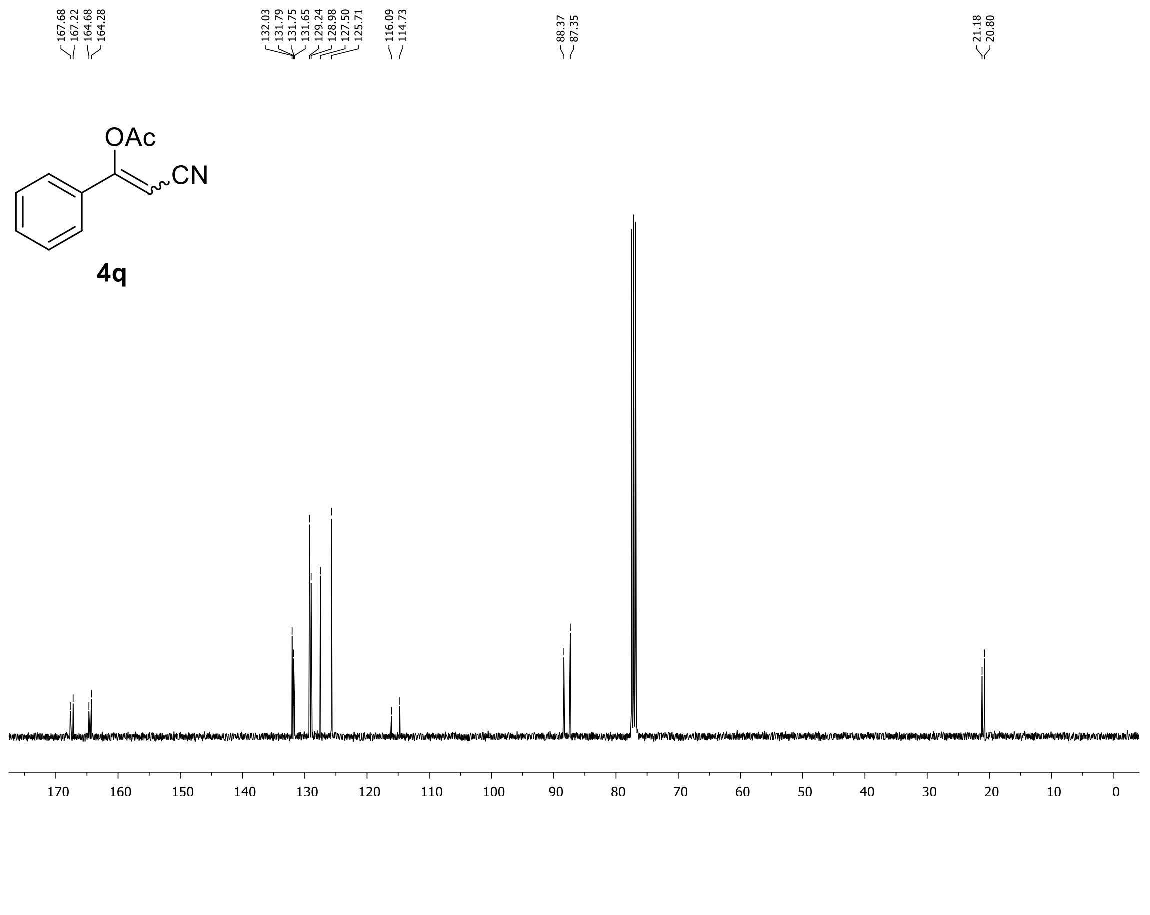

2-Iodoresorcinol (S1a)

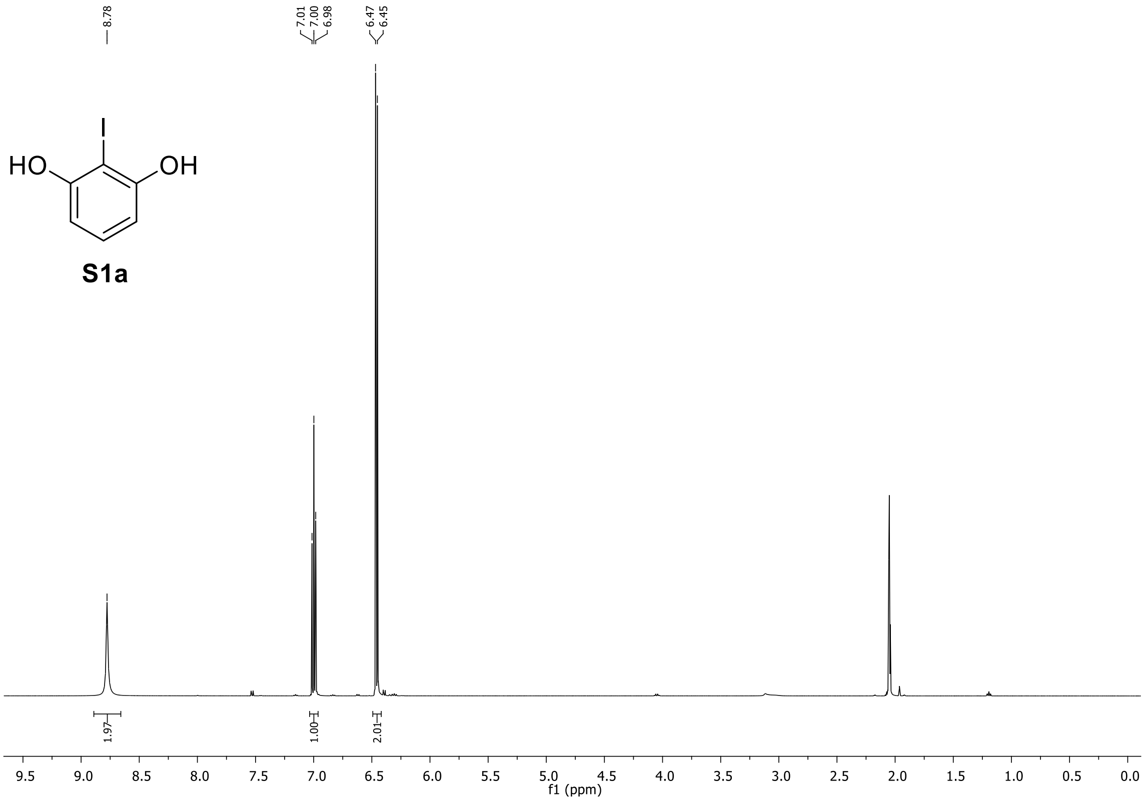

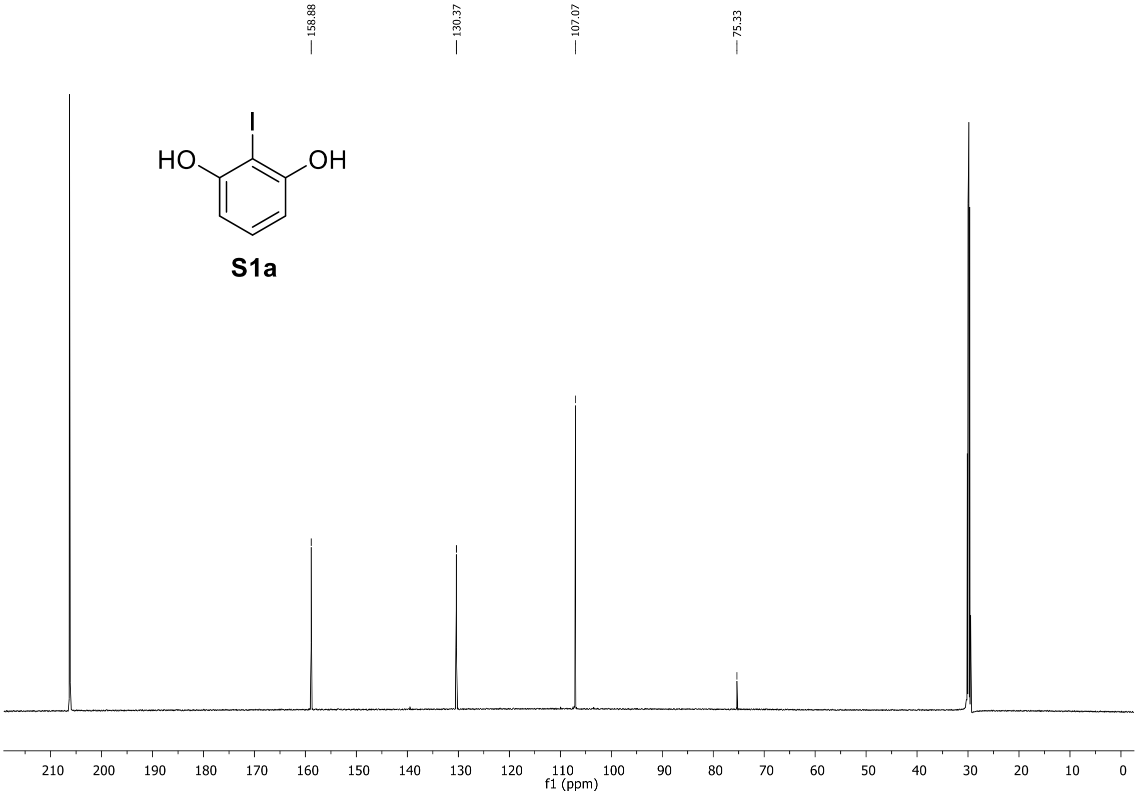

2-Iodo-5-methylbenzene-1,3-diol (S1b)

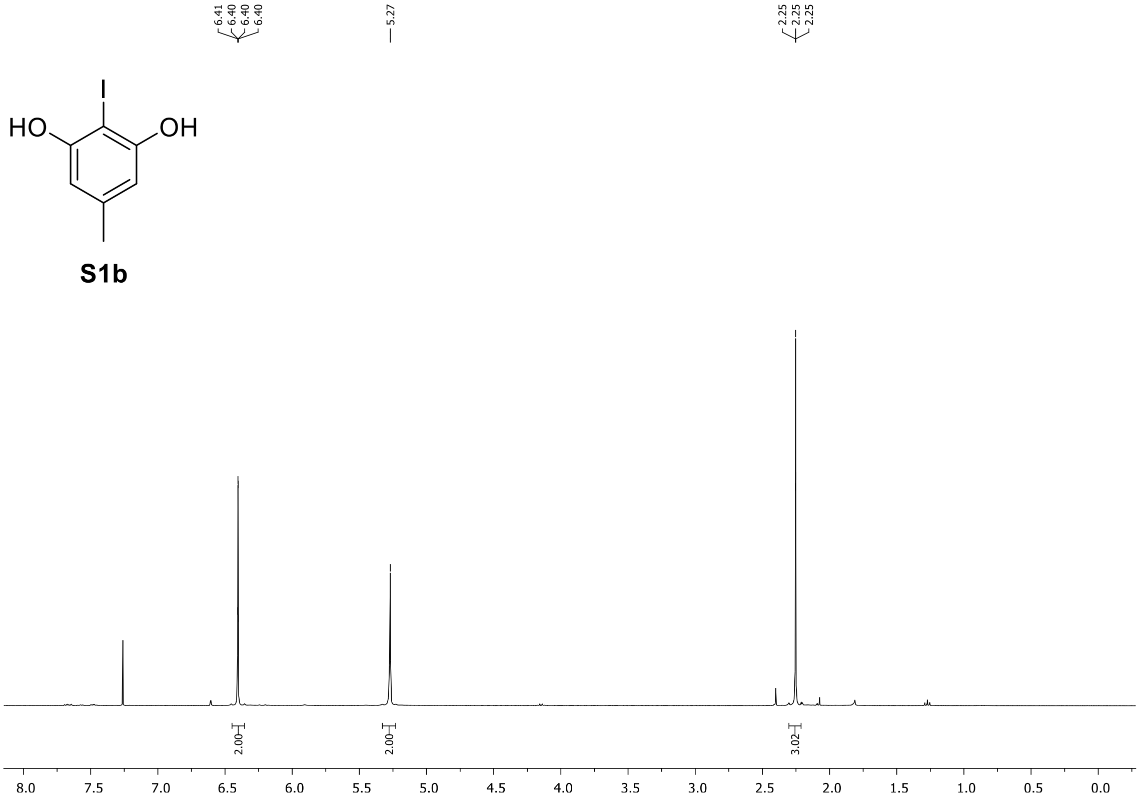

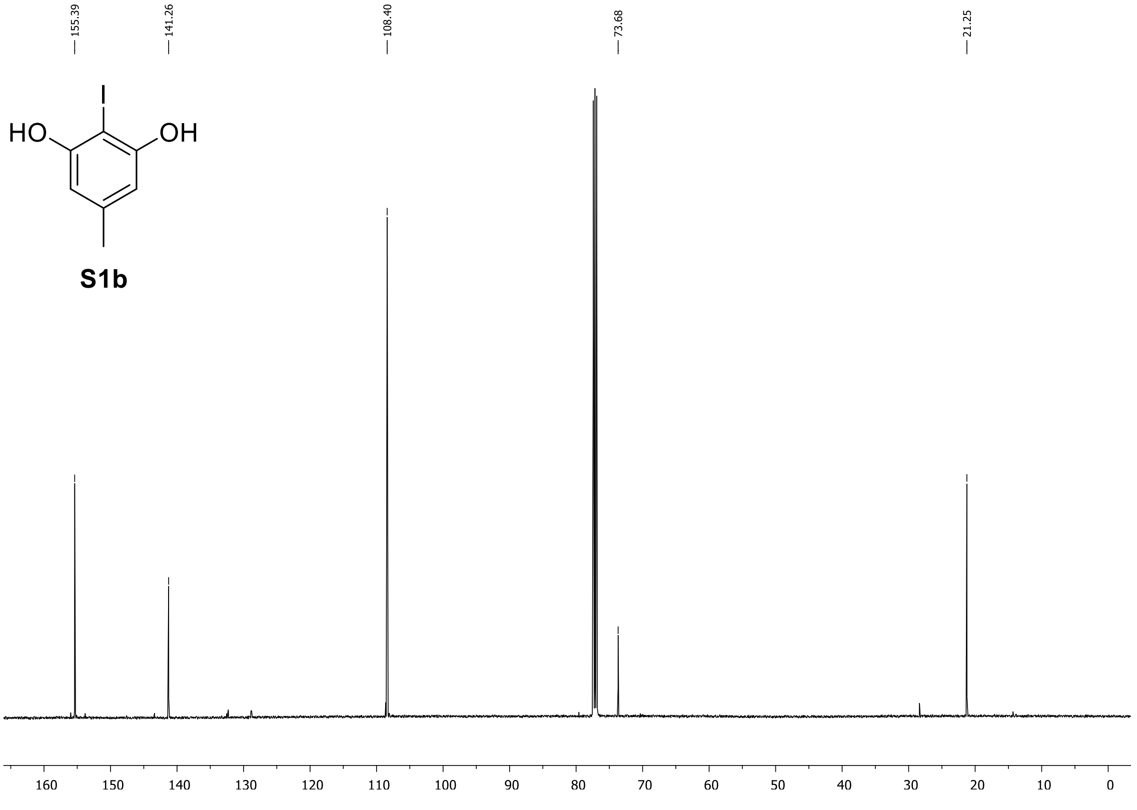

5-Bromo-2-iodobenzene-1,3-diol (S1c)

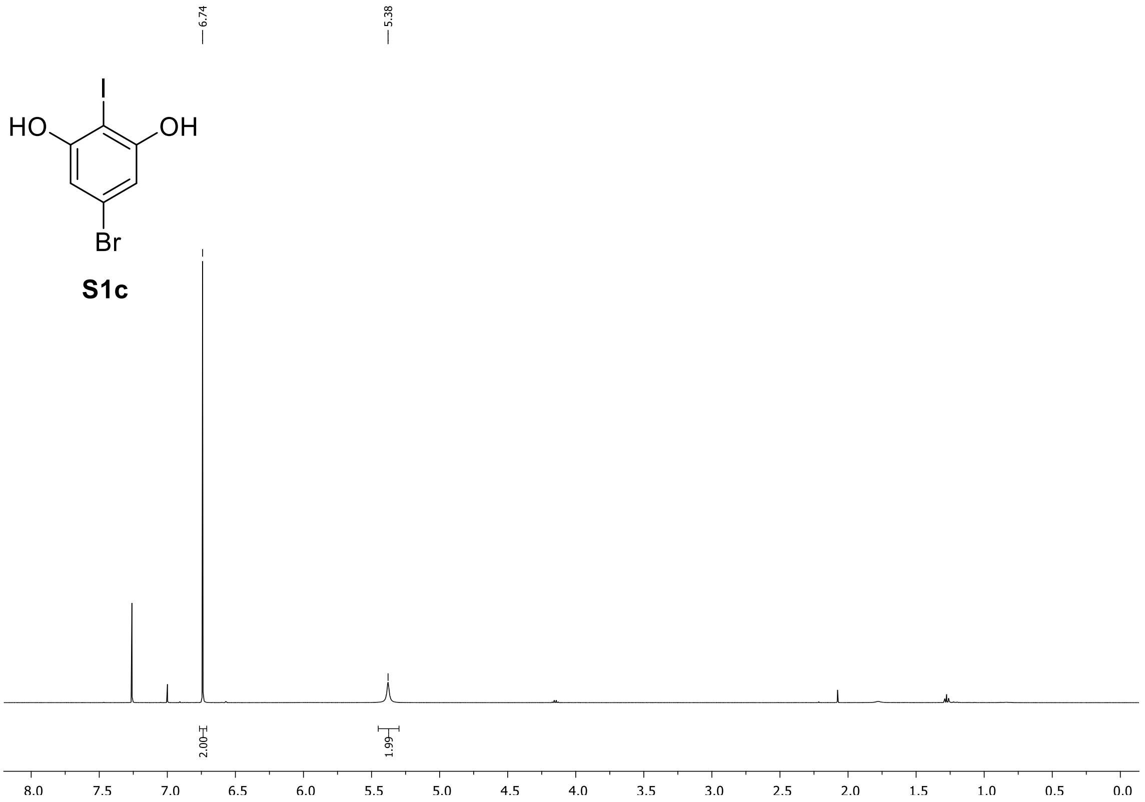

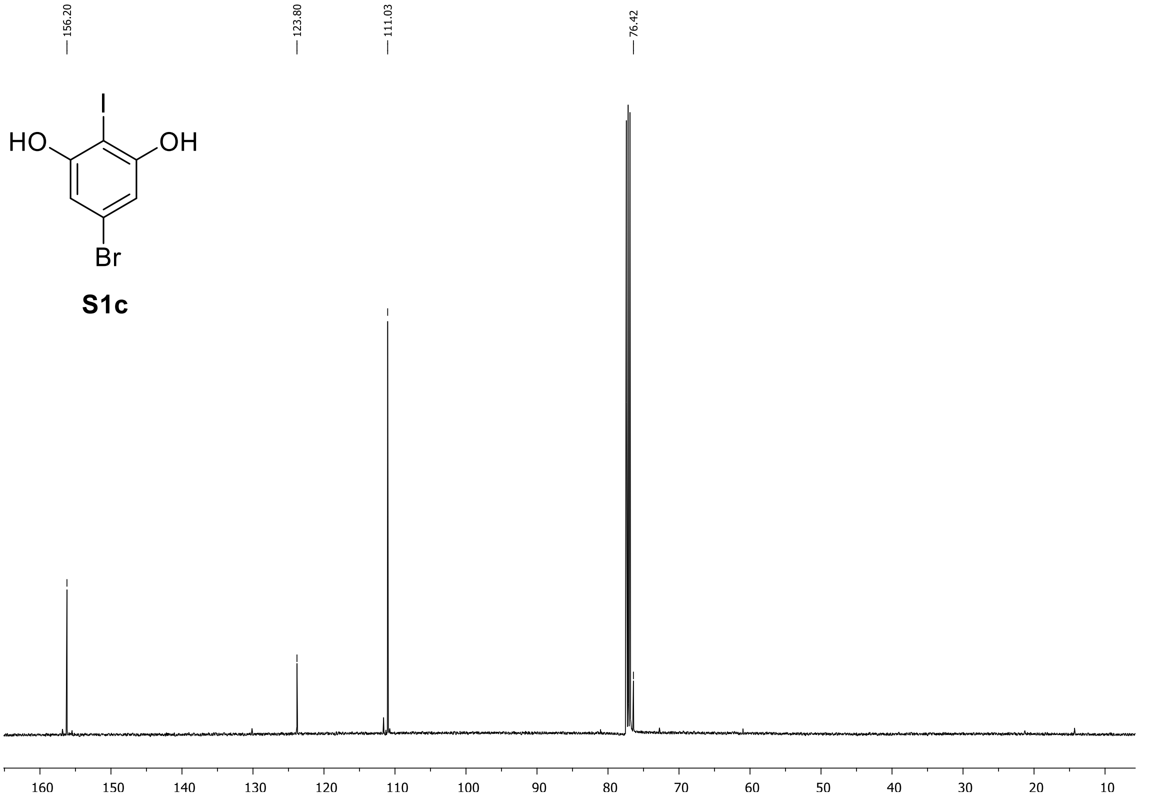

1-(3,5-Dihydroxy-4-iodophenyl)ethan-1-one (S1d)

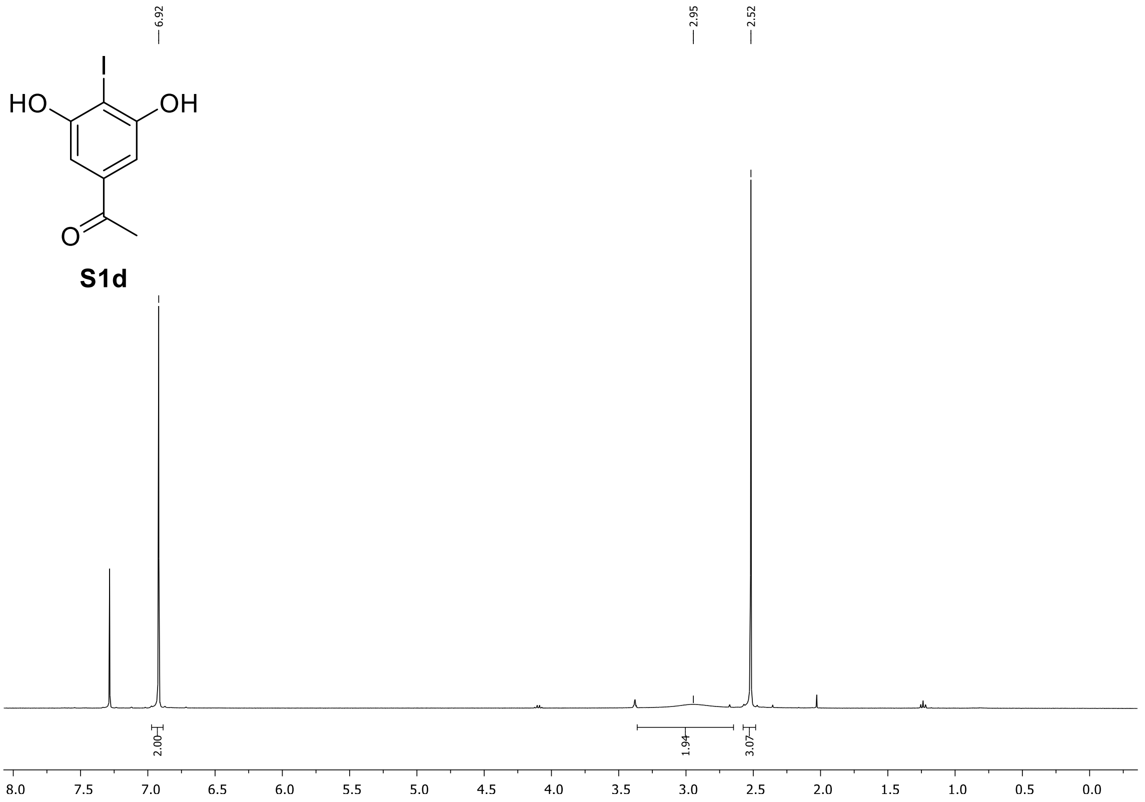

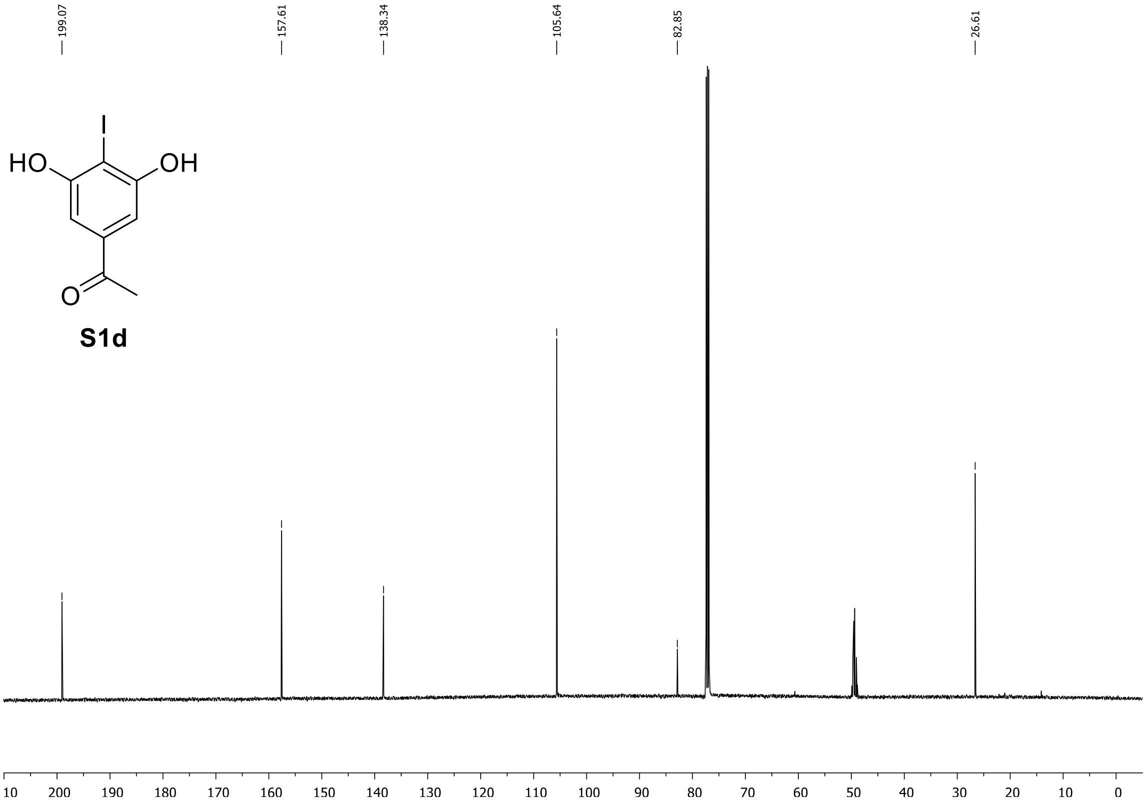

Dimethyl 2,2'-((2-iodo-1,3-phenylene)bis(oxy))(2*R*,2'*R*)-dipropionate (S2a)

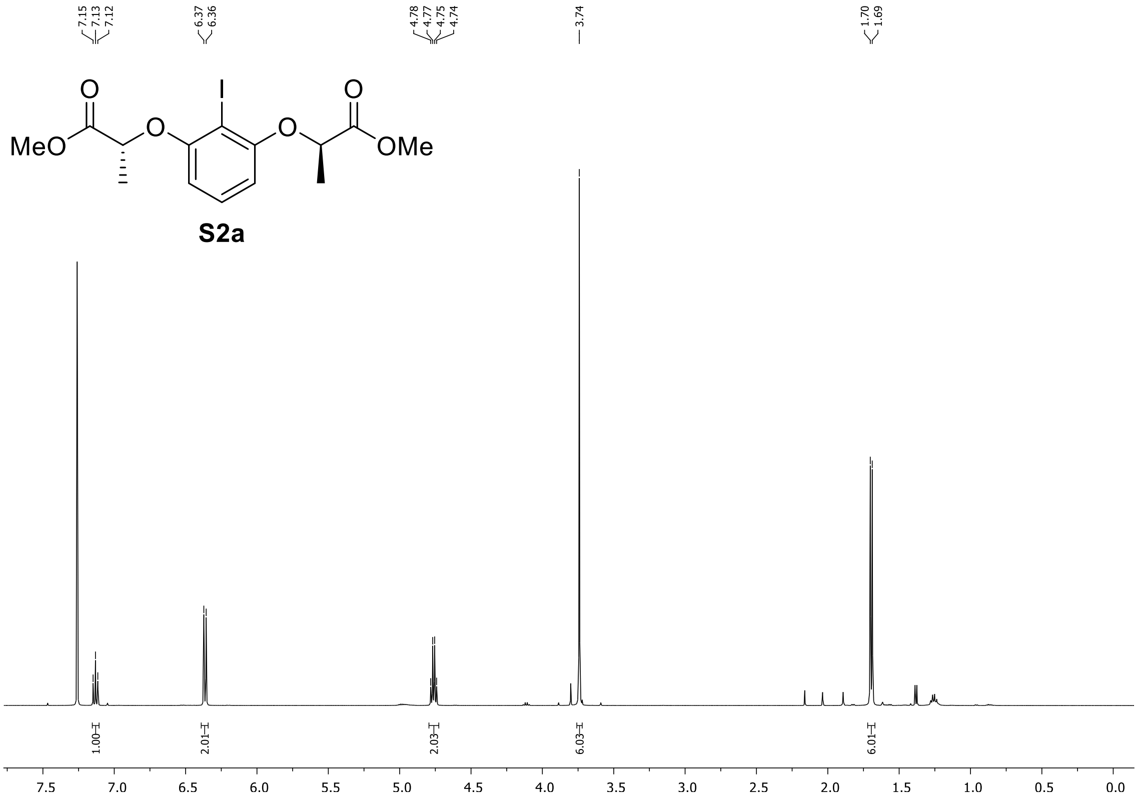

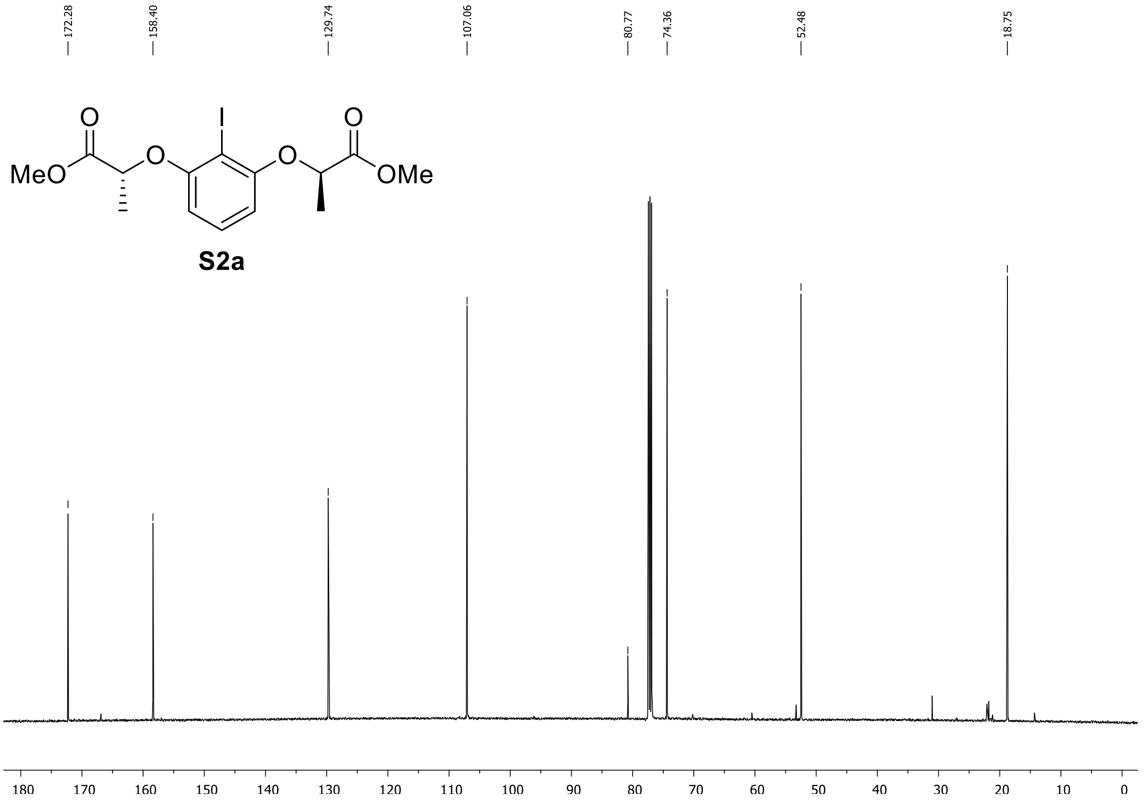

Dimethyl 2,2'-((2-iodo-1,3-phenylene)bis(oxy))(2*R*,2'*R*)-bis(3-phenylpropanoate) (S2b)

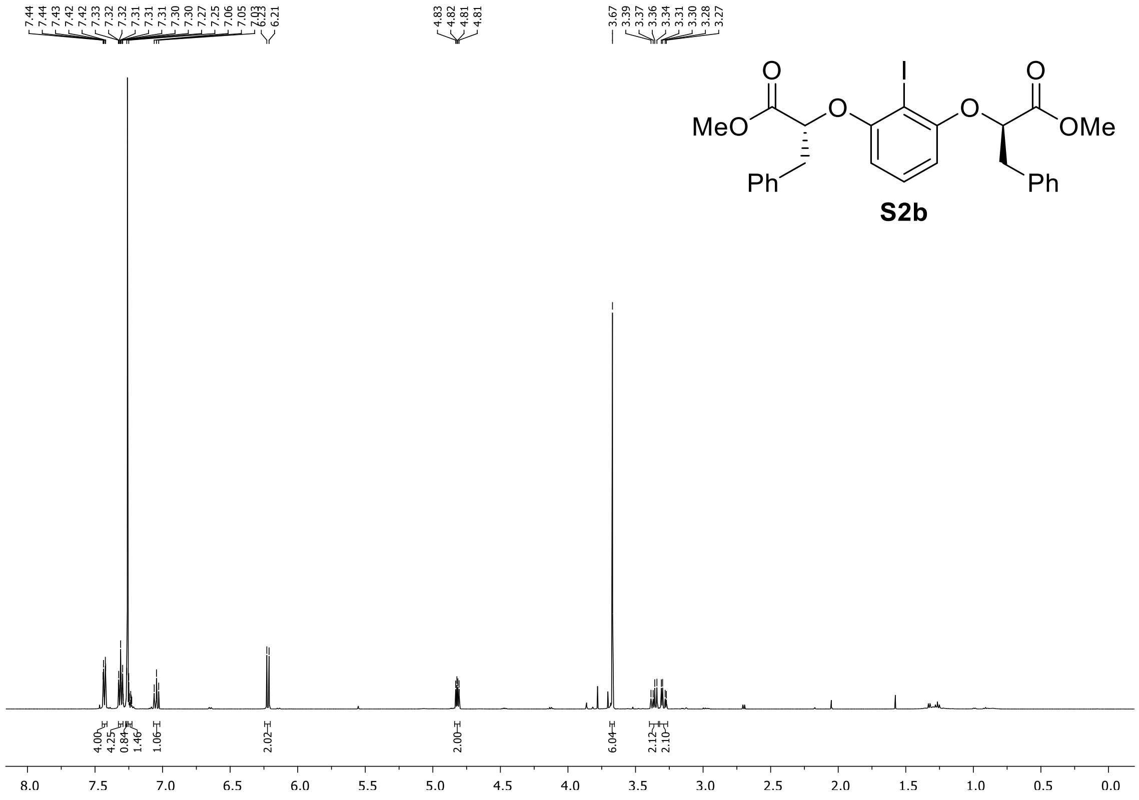

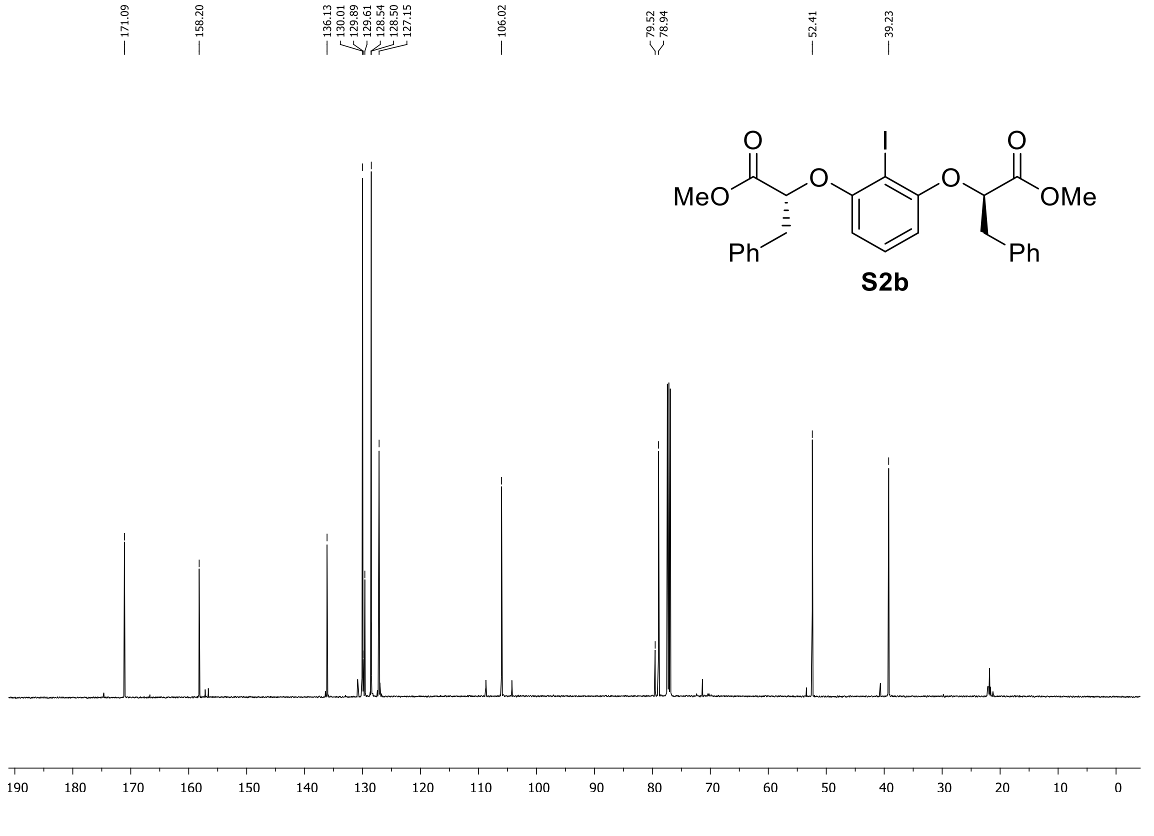

Dimethyl 2,2'-((2-iodo-5-methyl-1,3-phenylene)bis(oxy))(2*R*,2'*R*)-dipropionate (S2c)

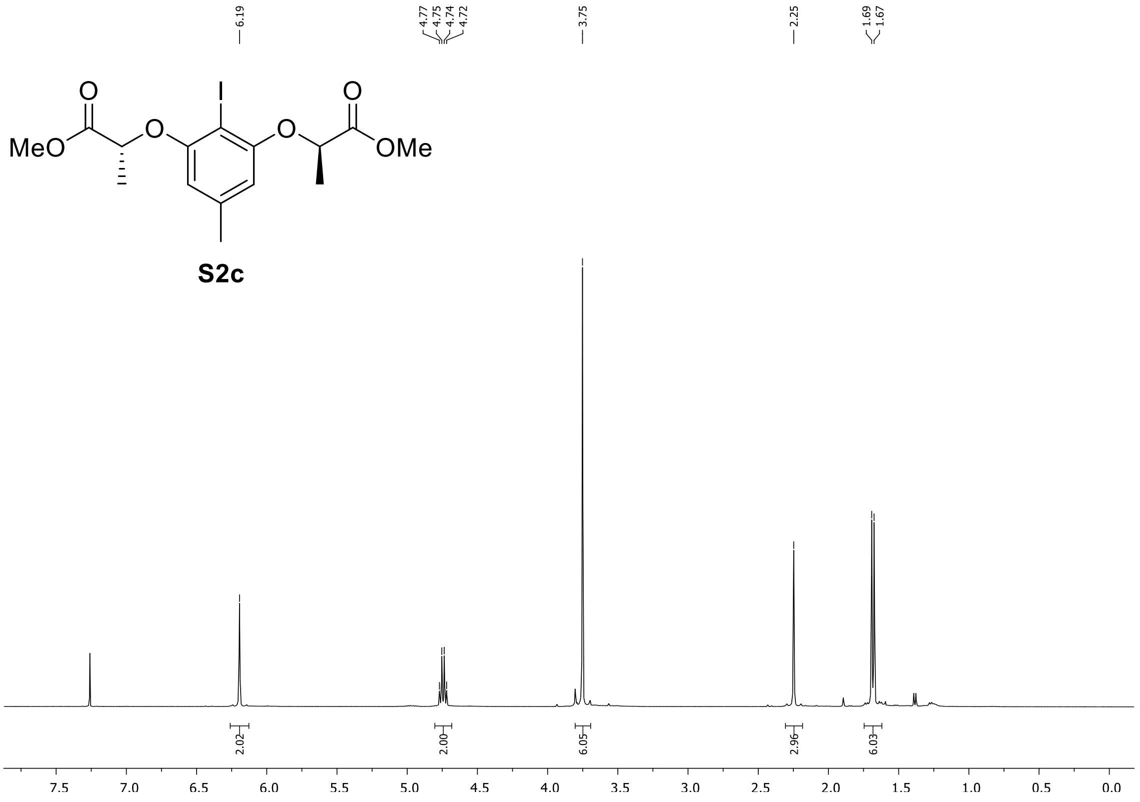

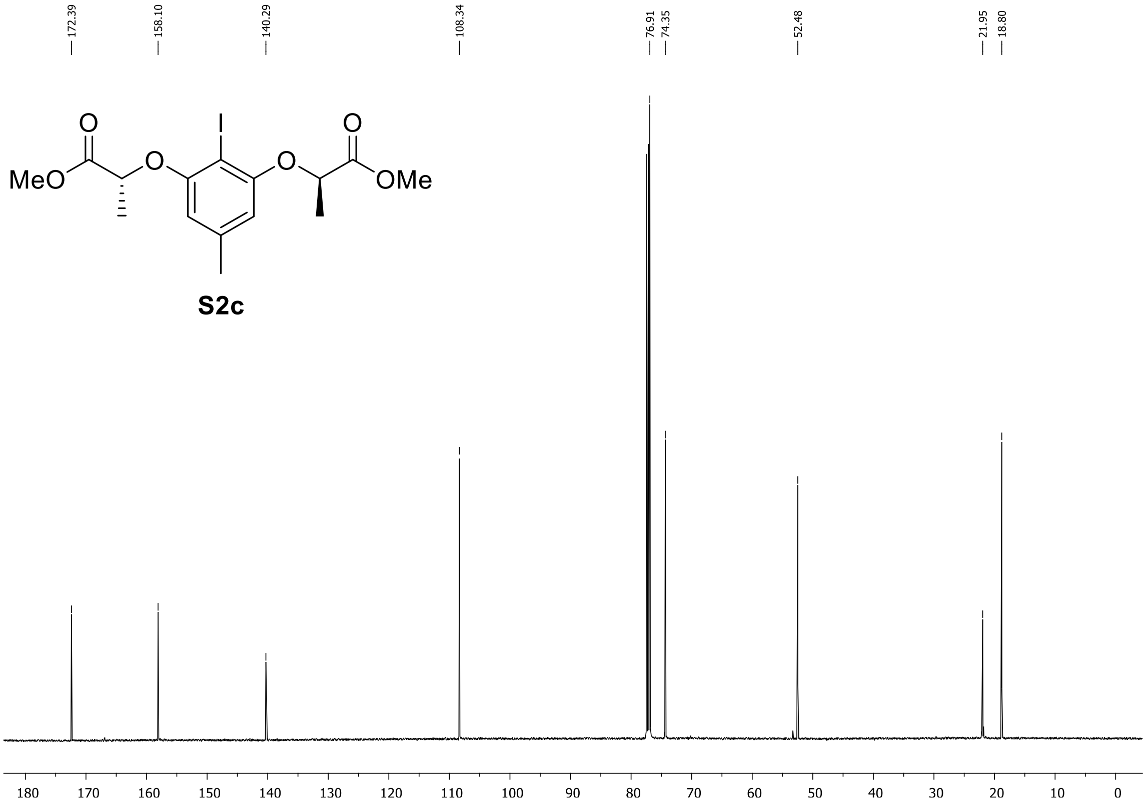

Dimethyl 2,2'-((5-bromo-2-iodo-1,3-phenylene)bis(oxy))(2*R*,2'*R*)-dipropionate (S2d)

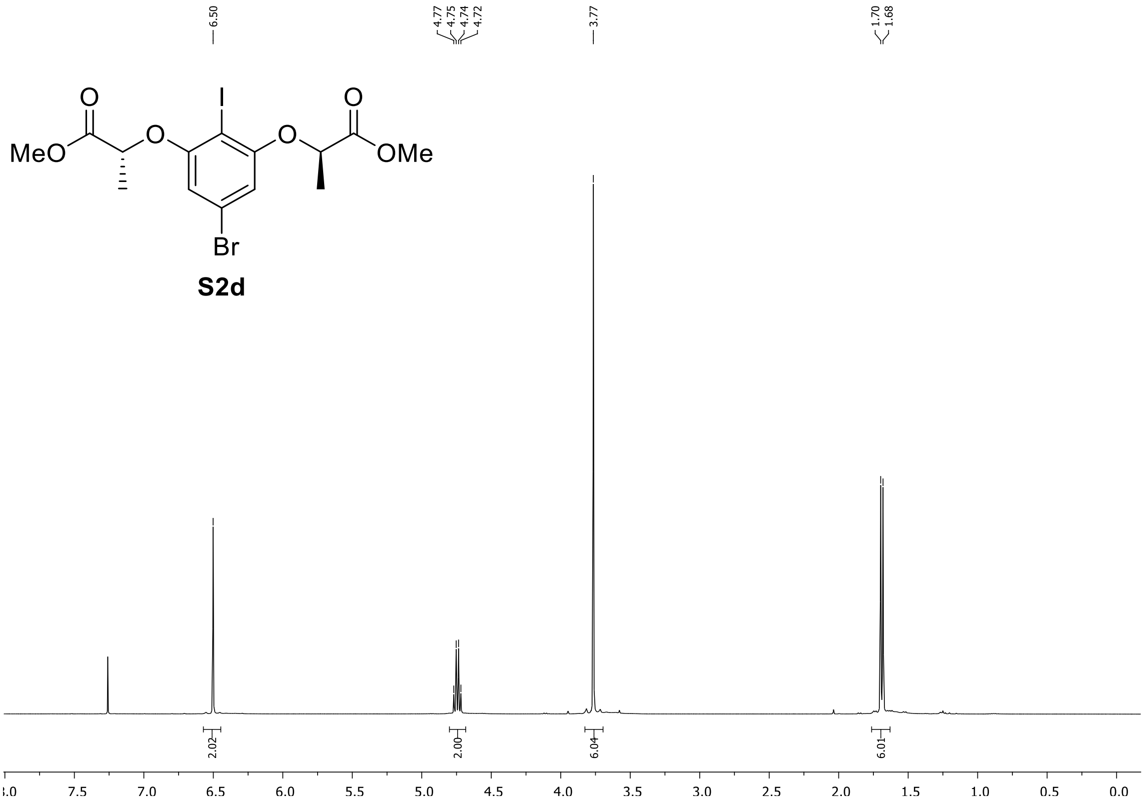

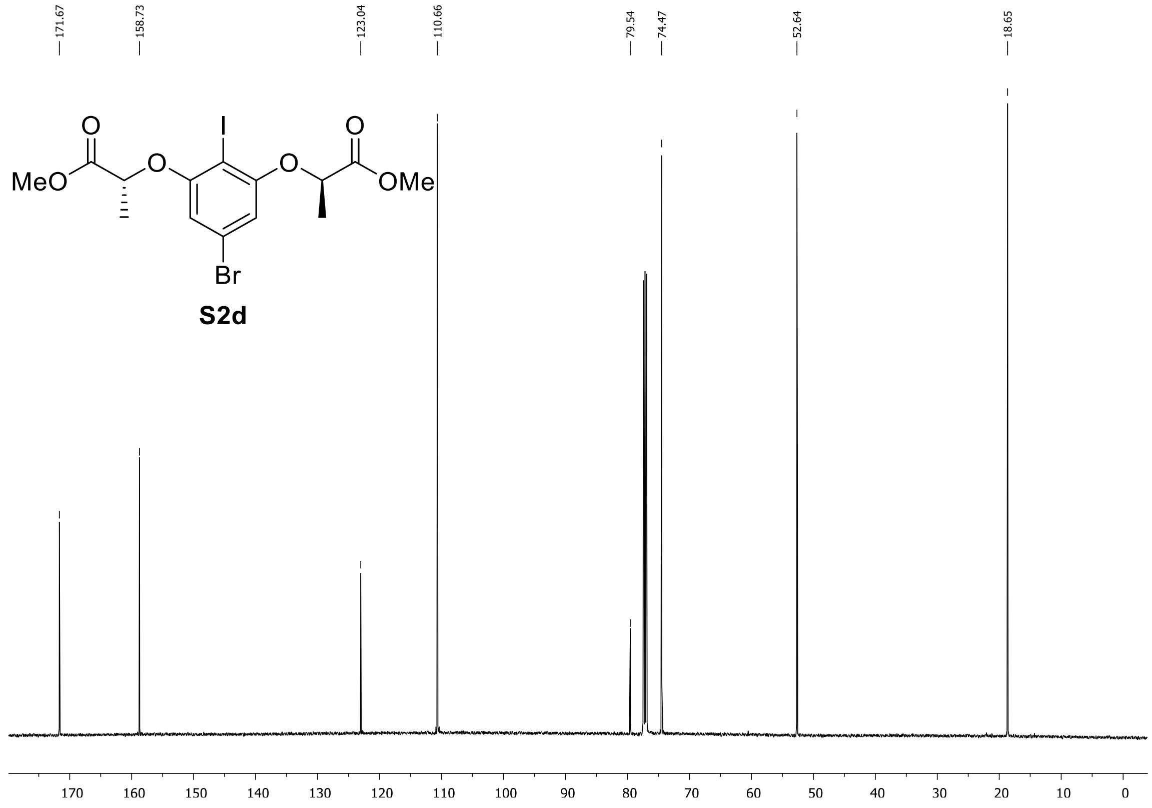

Dimethyl 2,2'-((5-acetyl-2-iodo-1,3-phenylene)bis(oxy))(2*R*,2'*R*)-dipropionate (S2e)

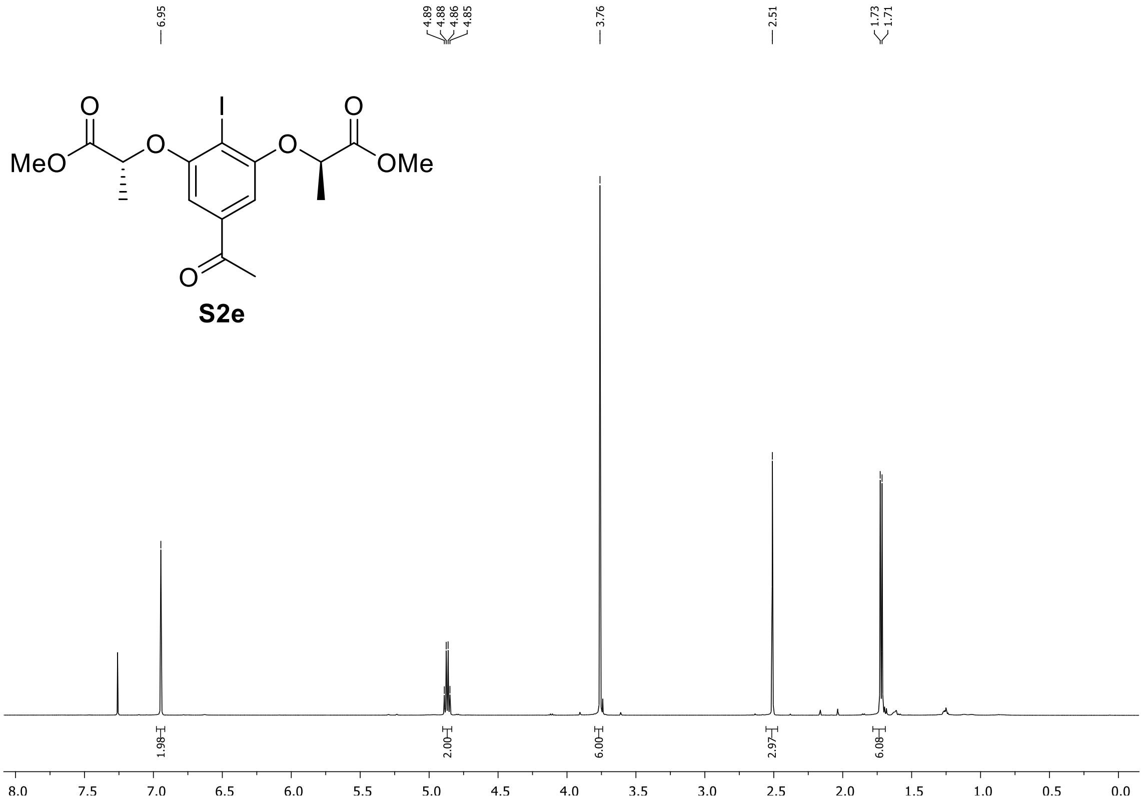

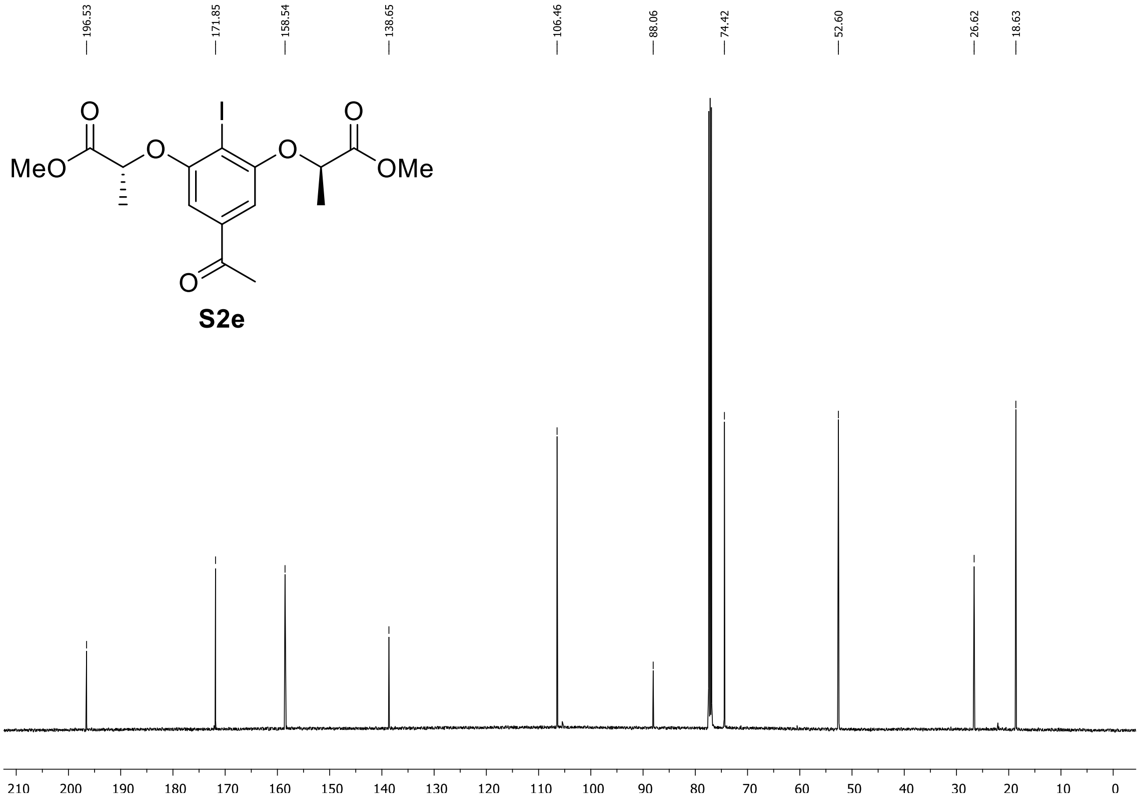

(2*R*,2'*R*)-2,2'-((2-Iodo-1,3-phenylene)bis(oxy))dipropionic acid (S3a)

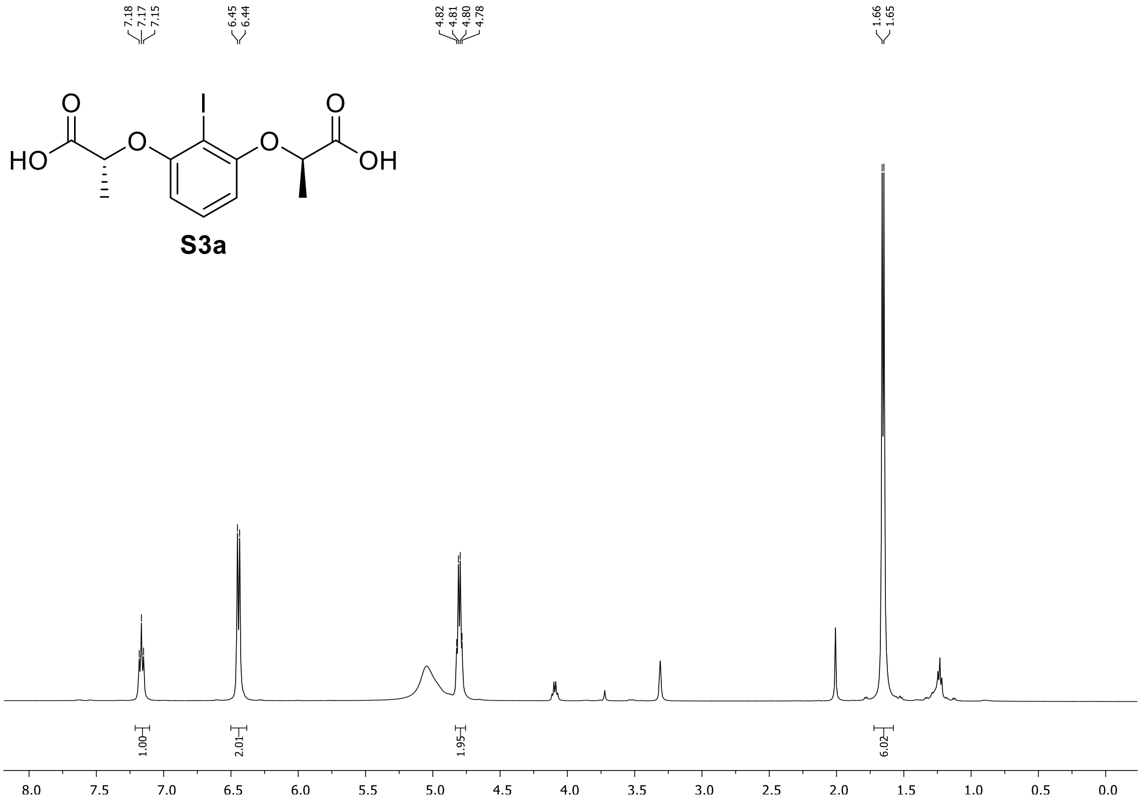

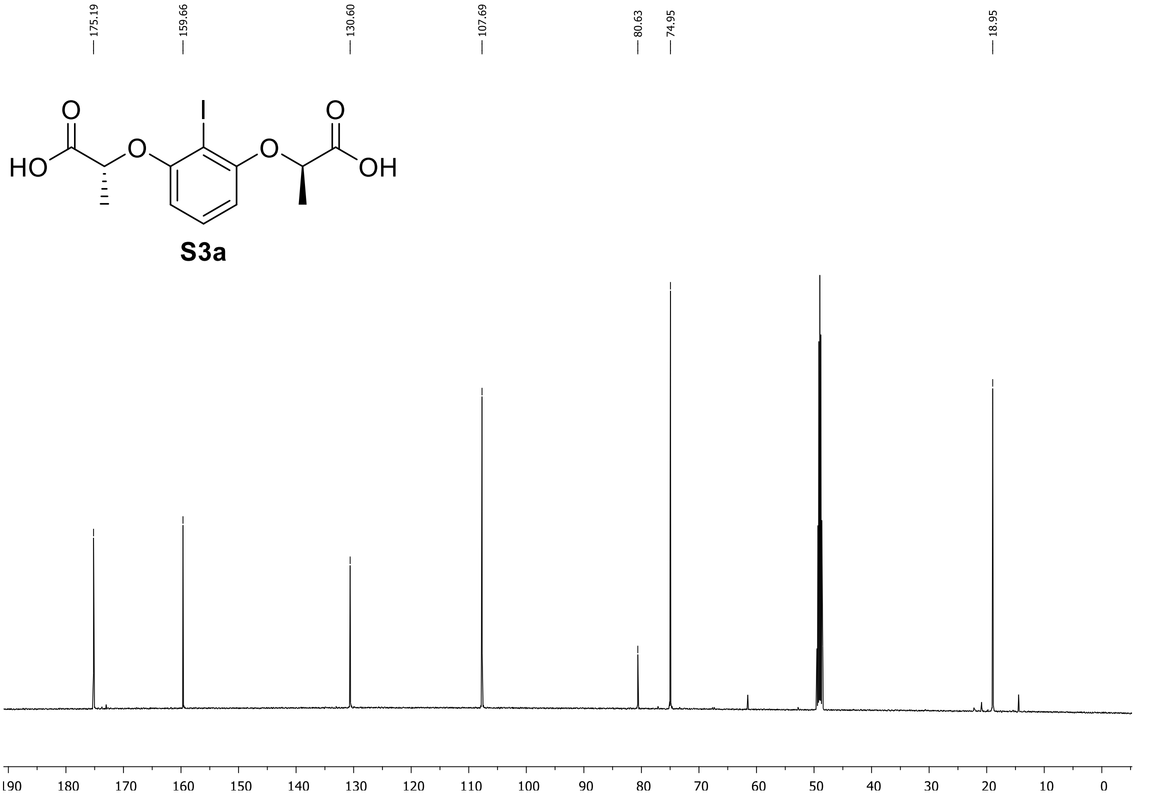

(2*R*,2'*R*)-2,2'-((2-Iodo-1,3-phenylene)bis(oxy))bis(3-phenylpropanoic acid) (S3b)

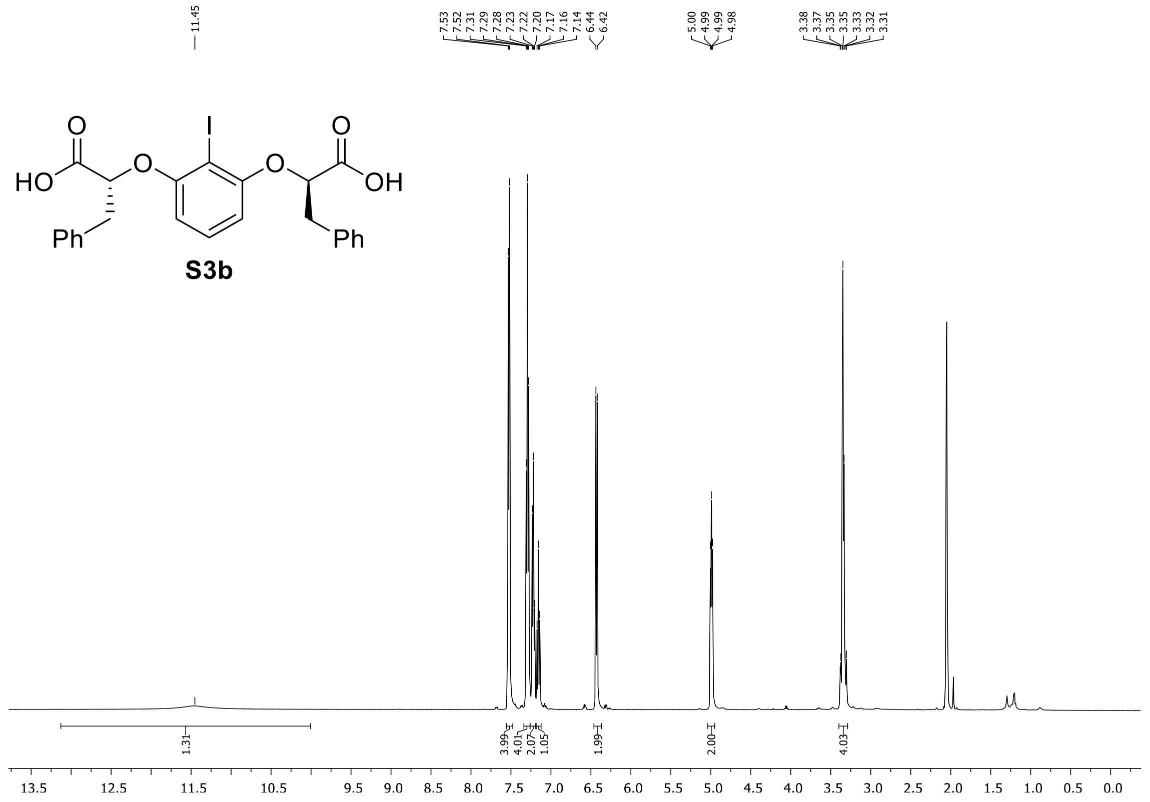

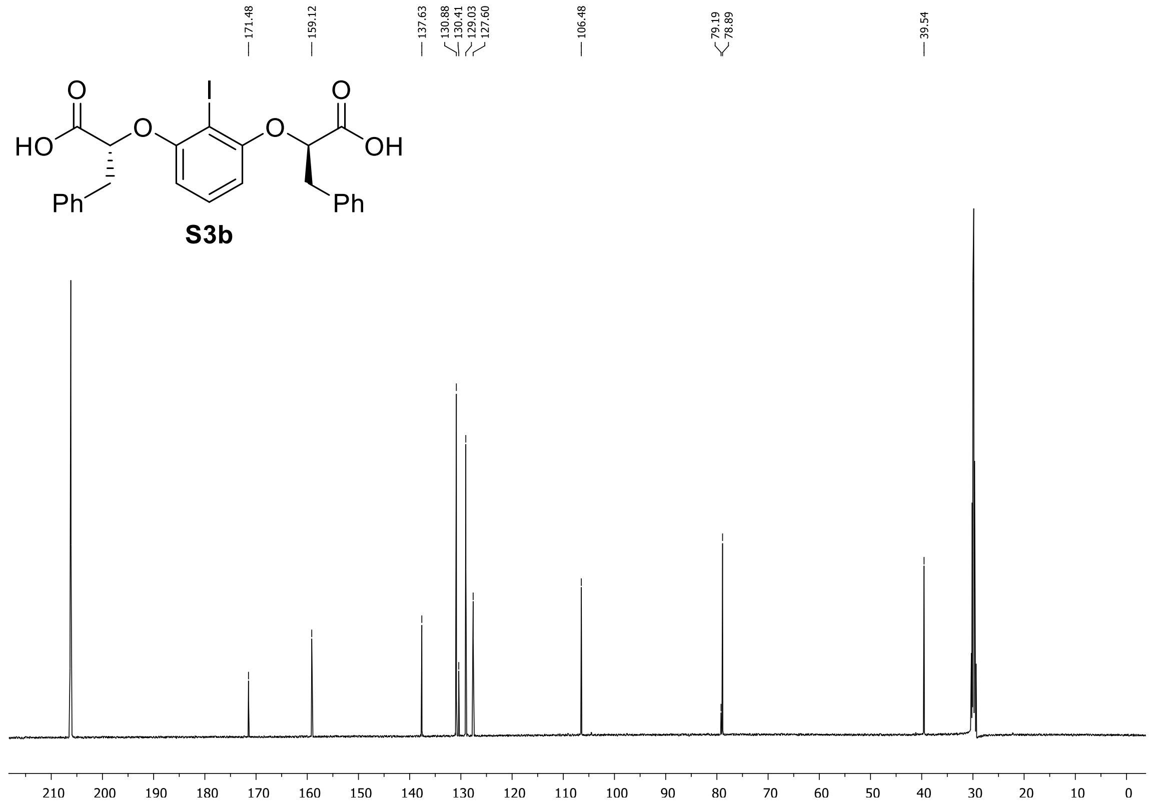

(2*R*,2'*R*)-2,2'-((2-Iodo-5-methyl-1,3-phenylene)bis(oxy))dipropionic acid (S3c)

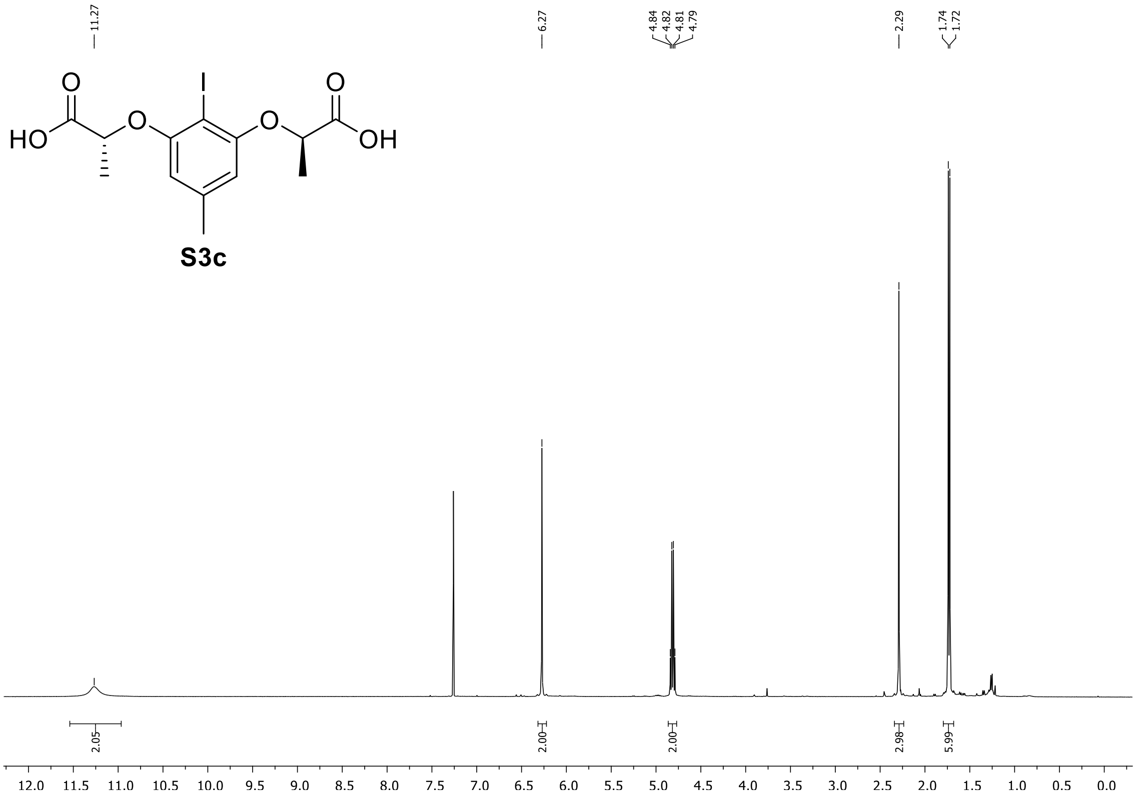

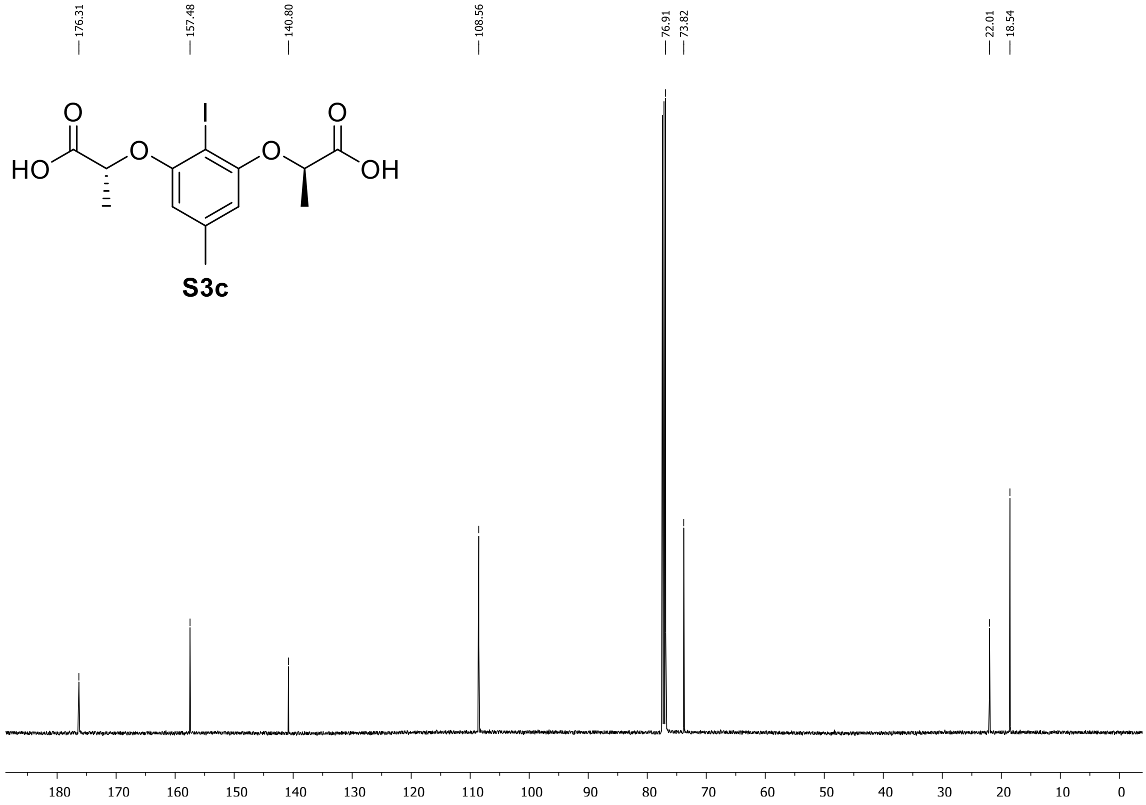

(2*R*,2'*R*)-2,2'-((5-Bromo-2-iodo-1,3-phenylene)bis(oxy))dipropionic acid (S3d)

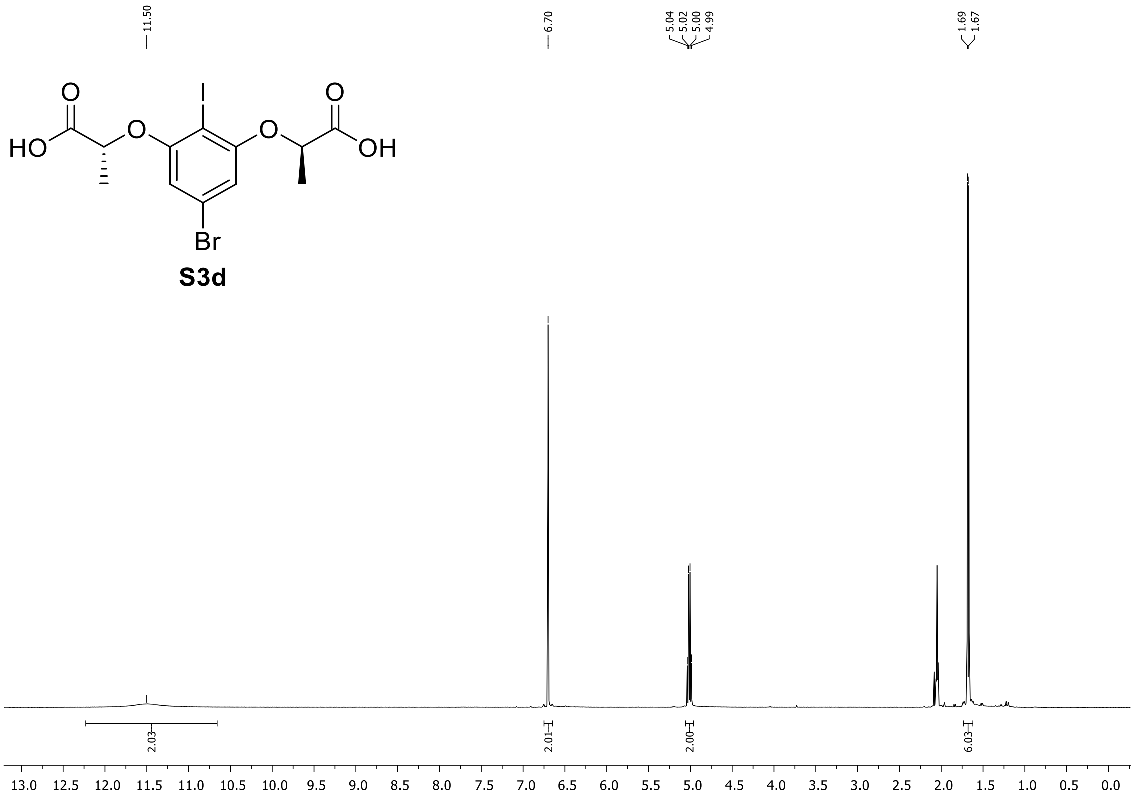

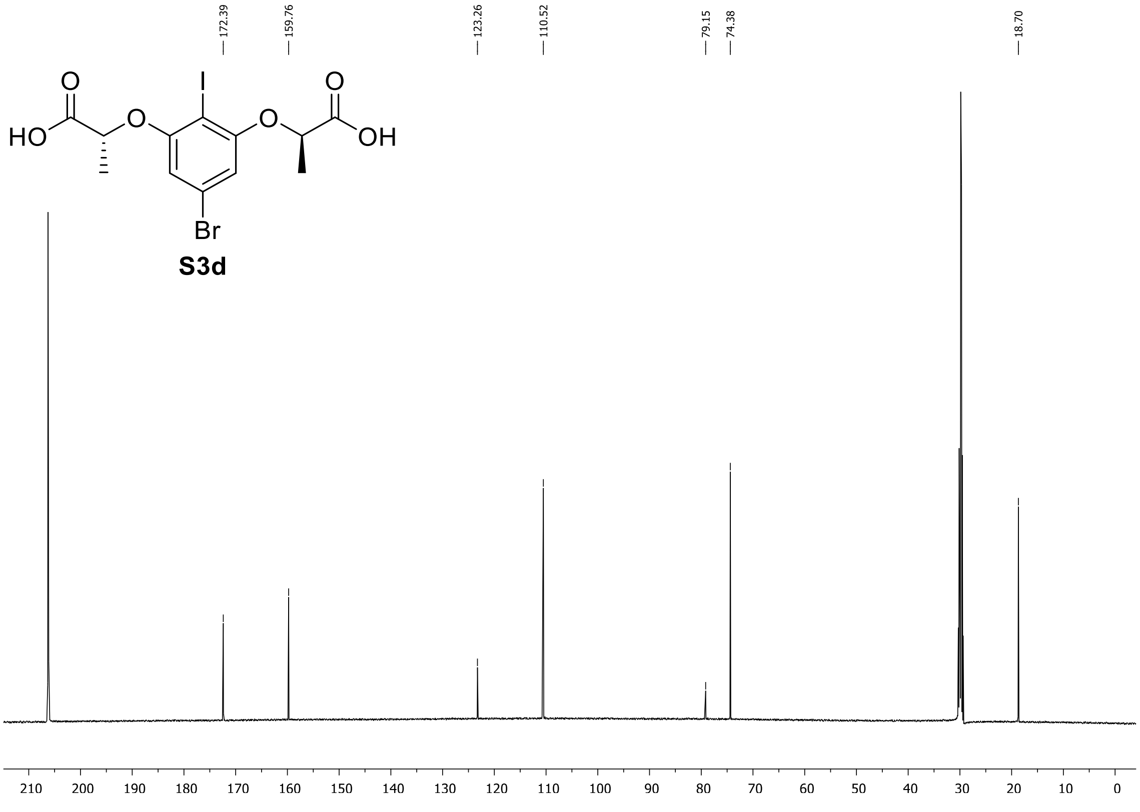

(2*R*,2'*R*)-2,2'-((5-Acetyl-2-iodo-1,3-phenylene)bis(oxy))dipropionic acid (S3e)

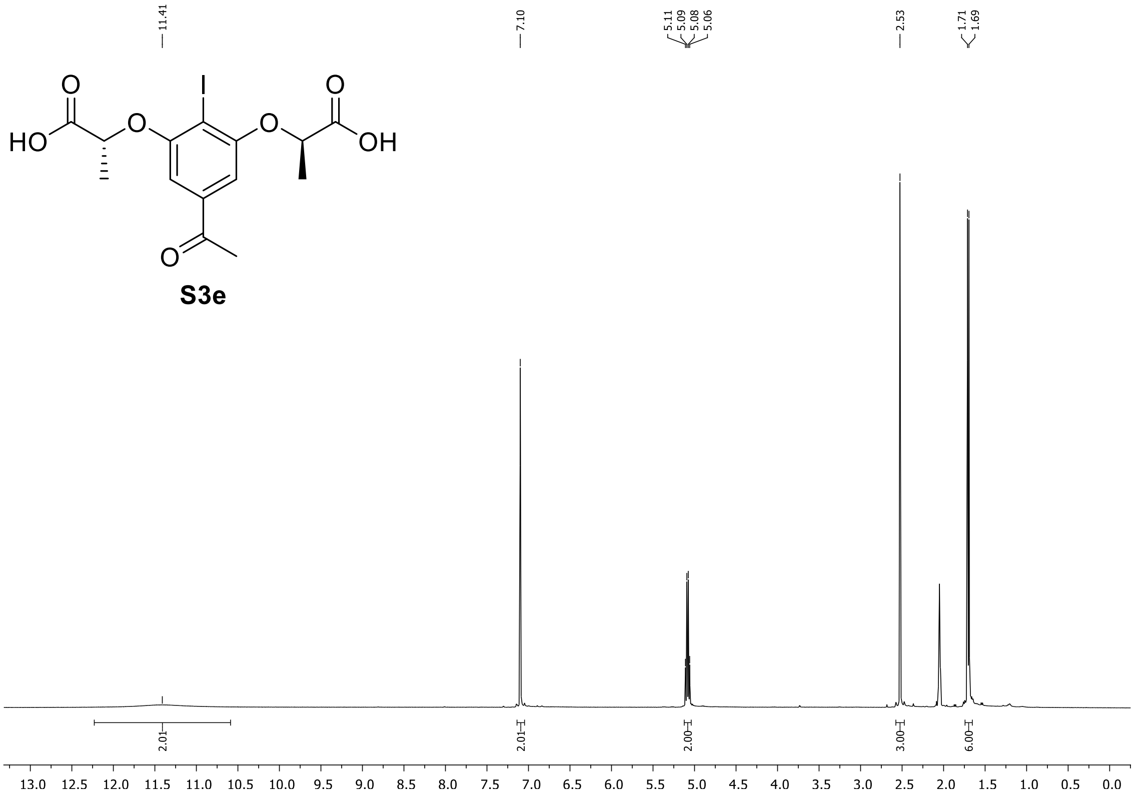

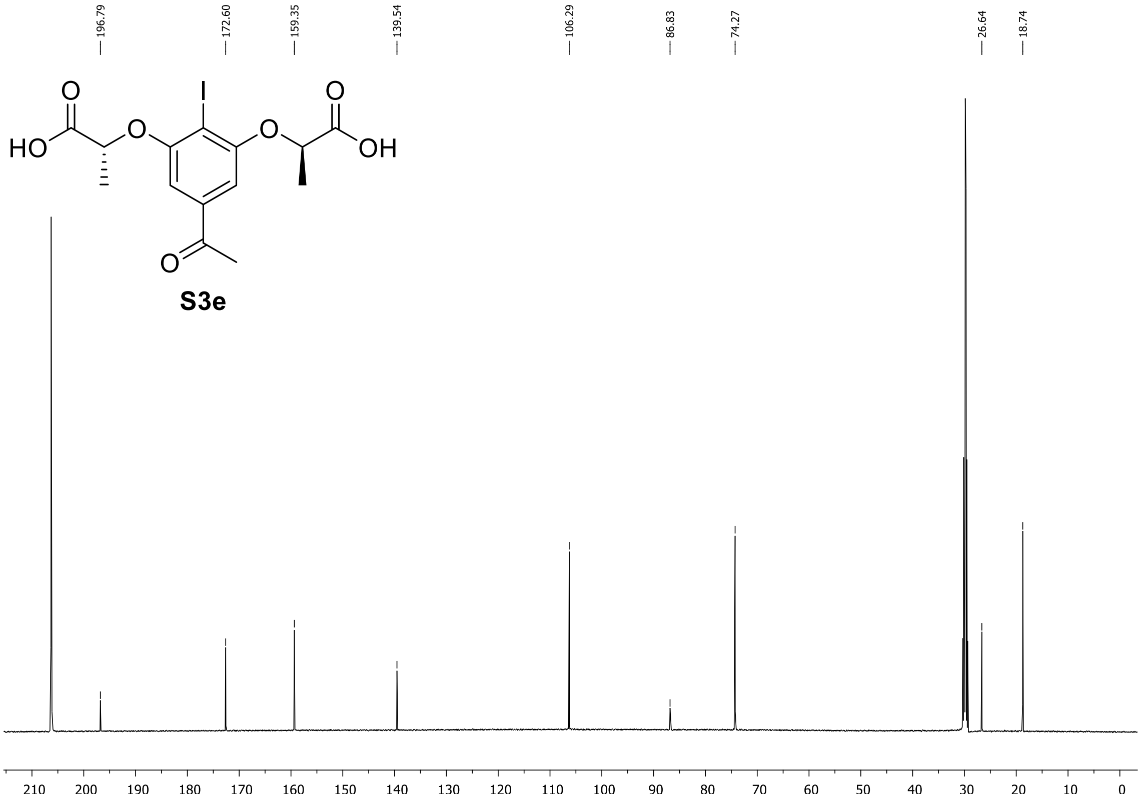

(2*R*,2'*R*)-2,2'-((2-Iodo-1,3-phenylene)bis(oxy))bis(*N*-methylpropanamide) (S4a)

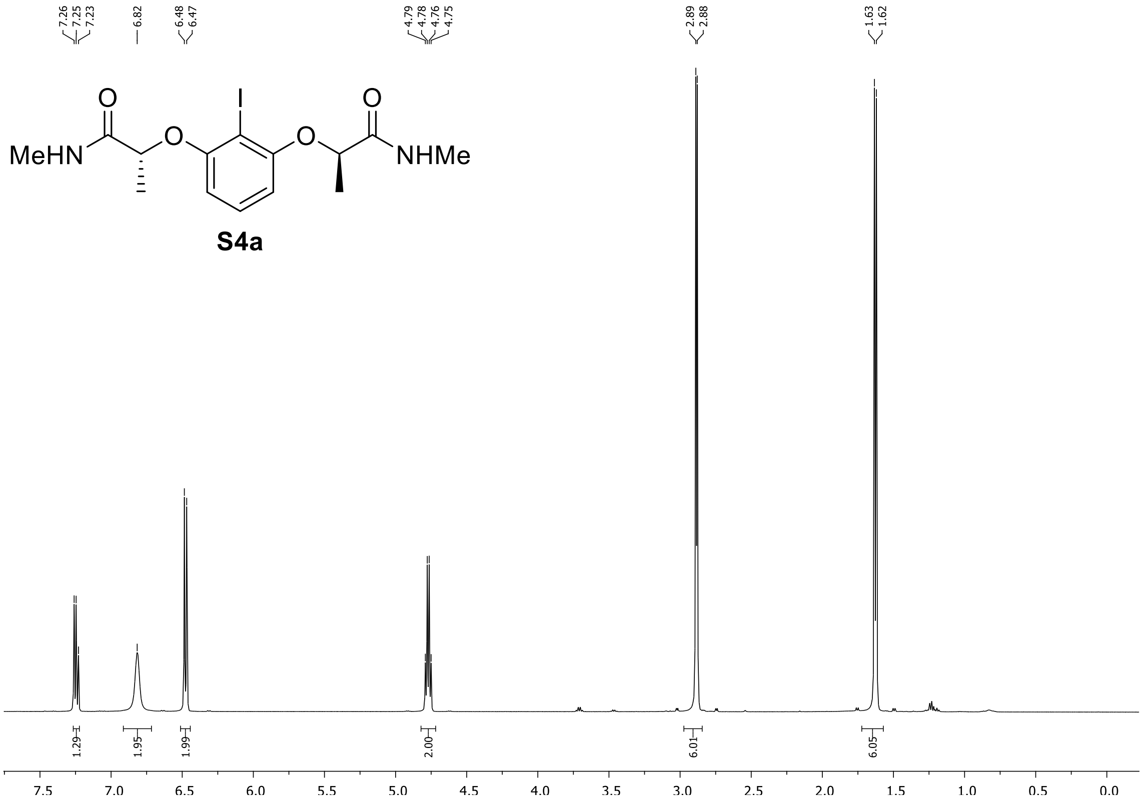

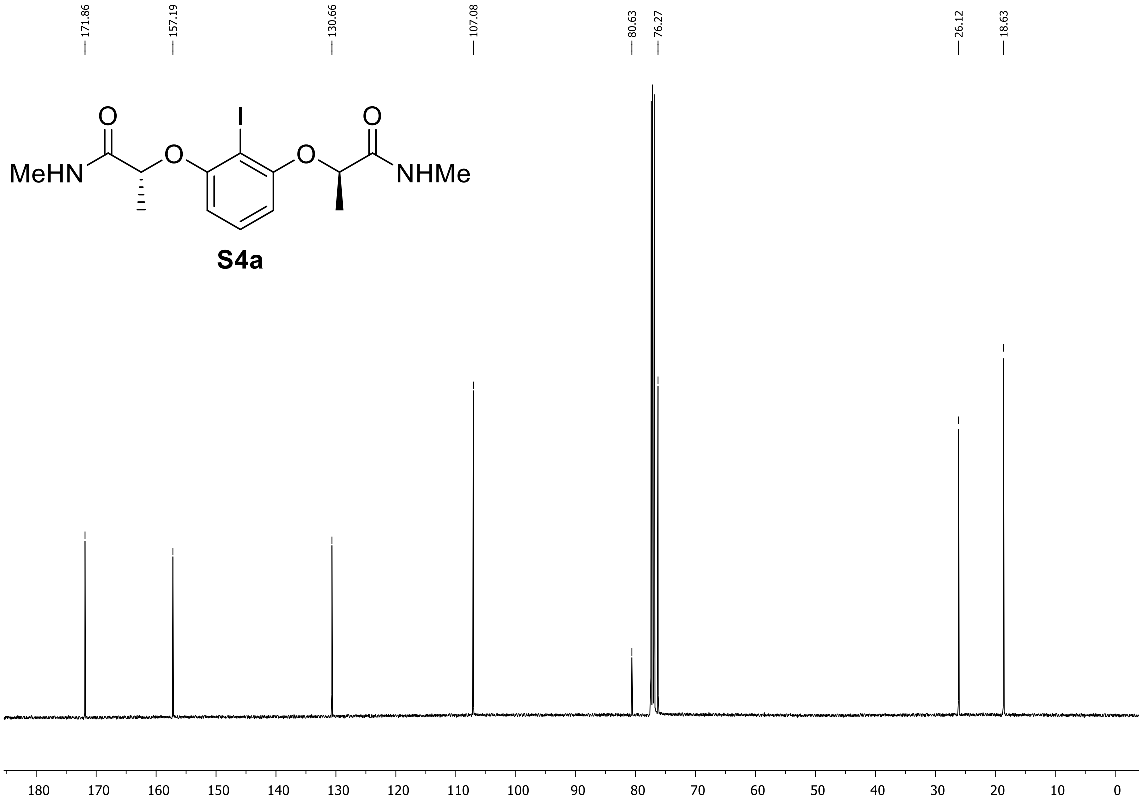

Dimesityl 2,2'-((2-iodo-1,3-phenylene)bis(oxy))(2*R*,2'*R*)-dipropionate (S4b)

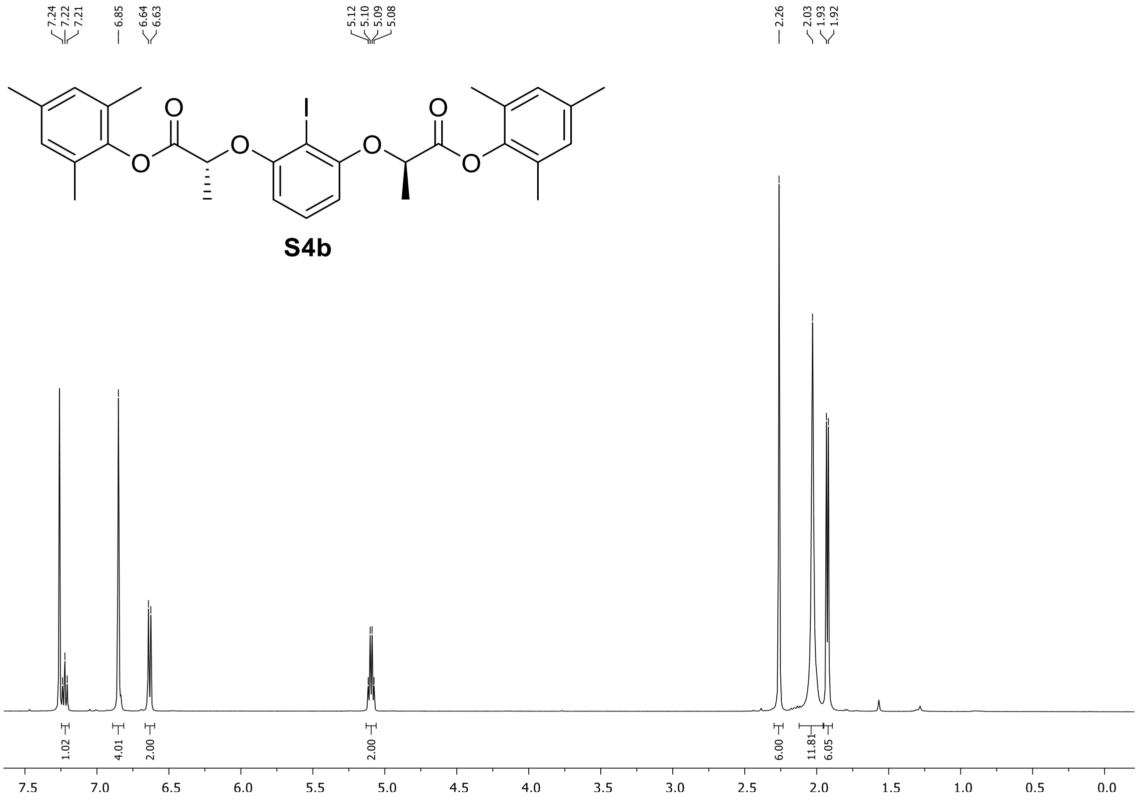

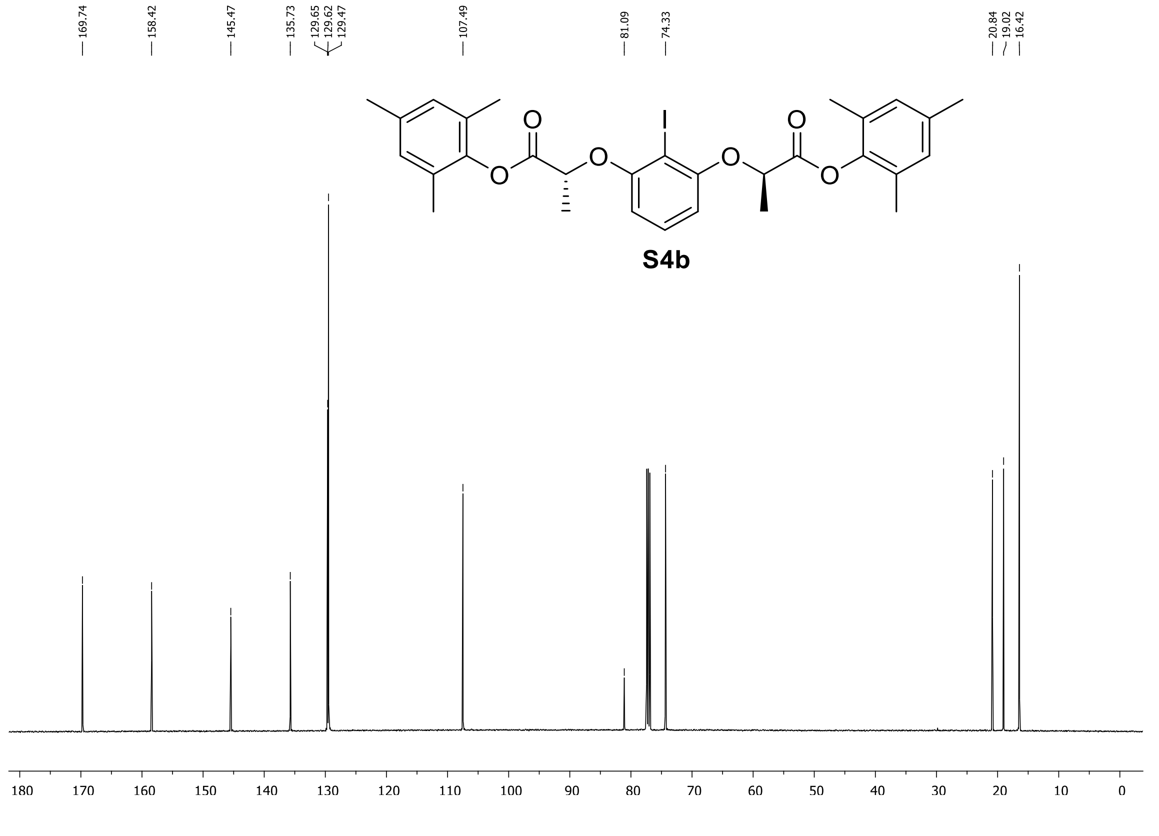

(2*R*,2'*R*)-2,2'-((2-Iodo-1,3-phenylene)bis(oxy))bis(*N*-mesitylpropanamide) (S4c)

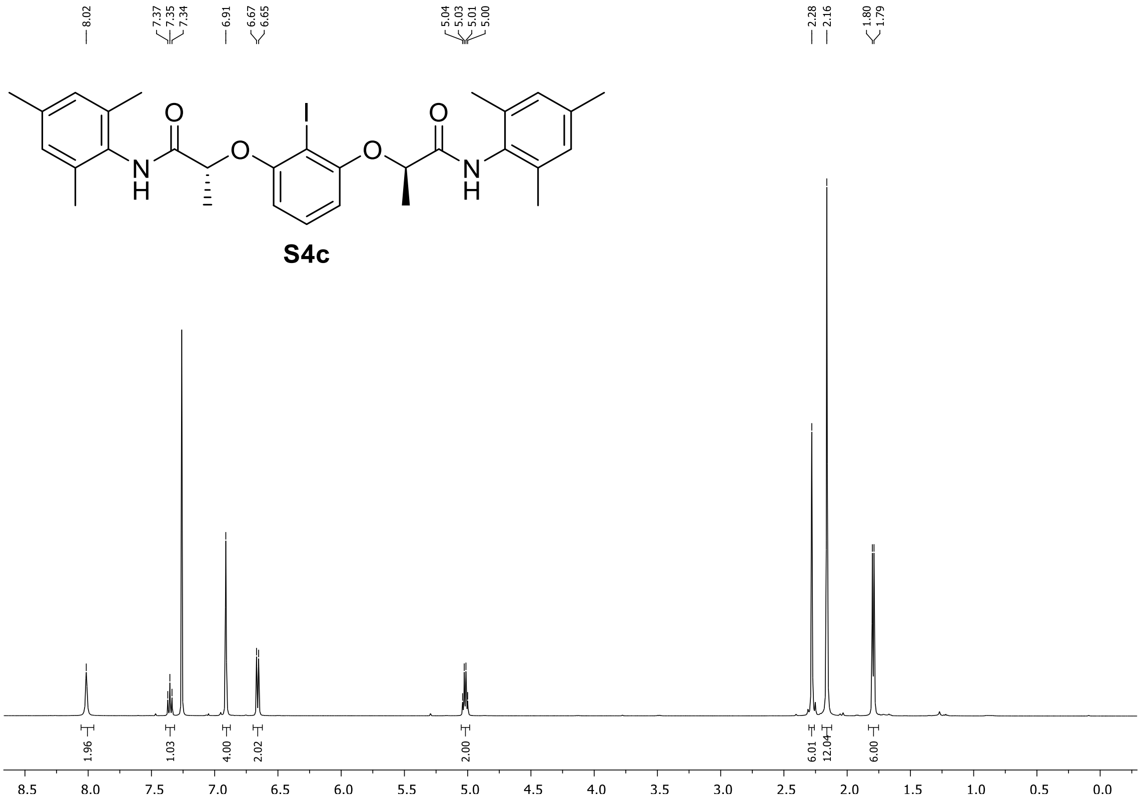

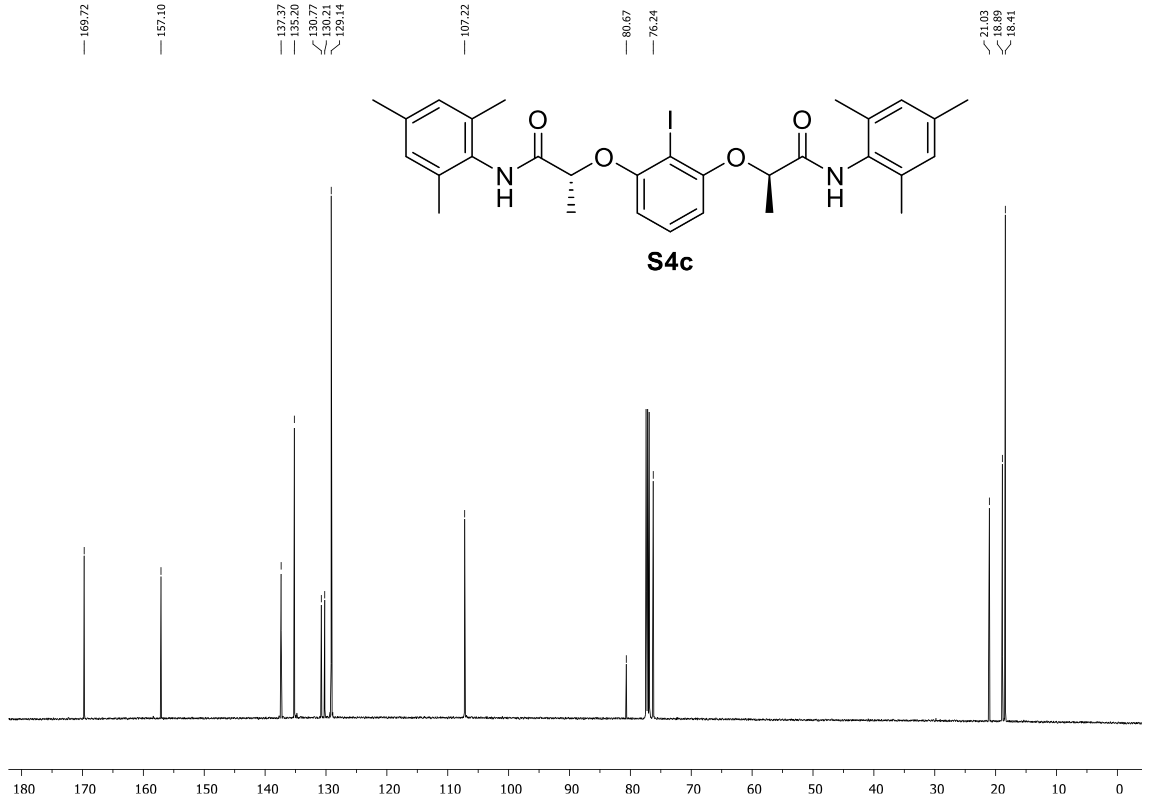

(2*R*,2'*R*)-2,2'-((2-Iodo-1,3-phenylene)bis(oxy))bis(*N*-(2,6-diisopropylphenyl)propanamide) (7a)

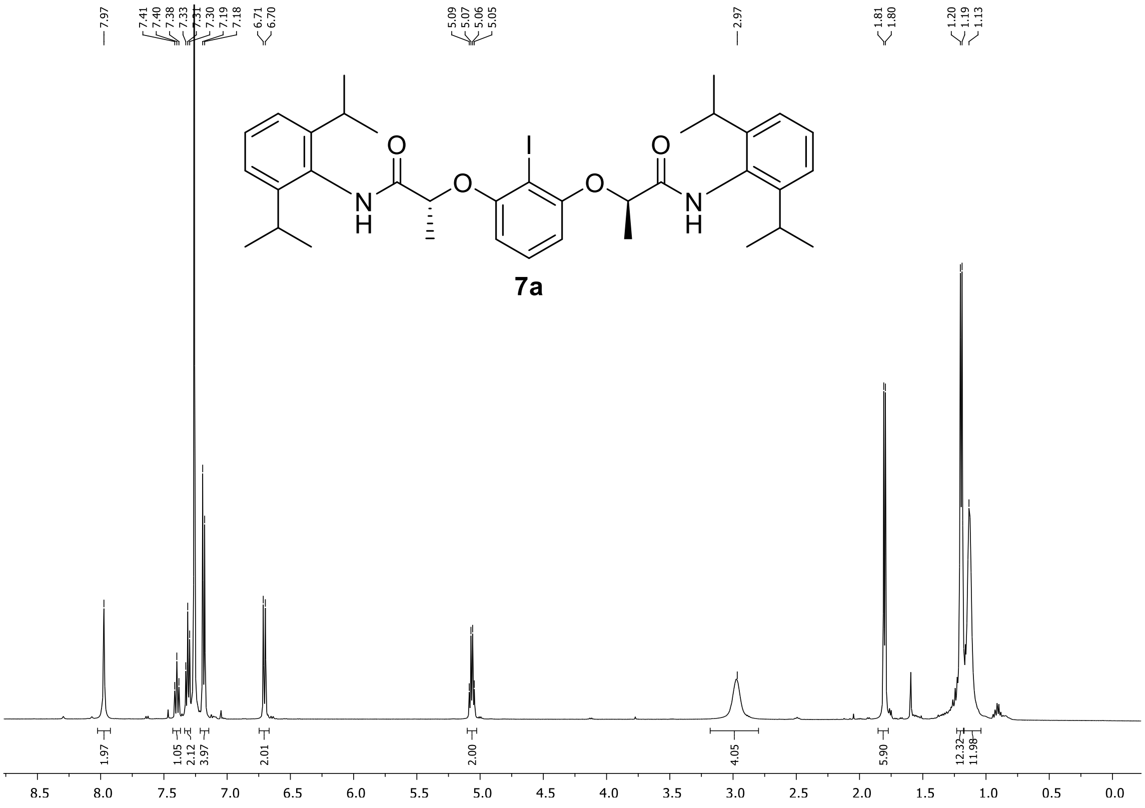

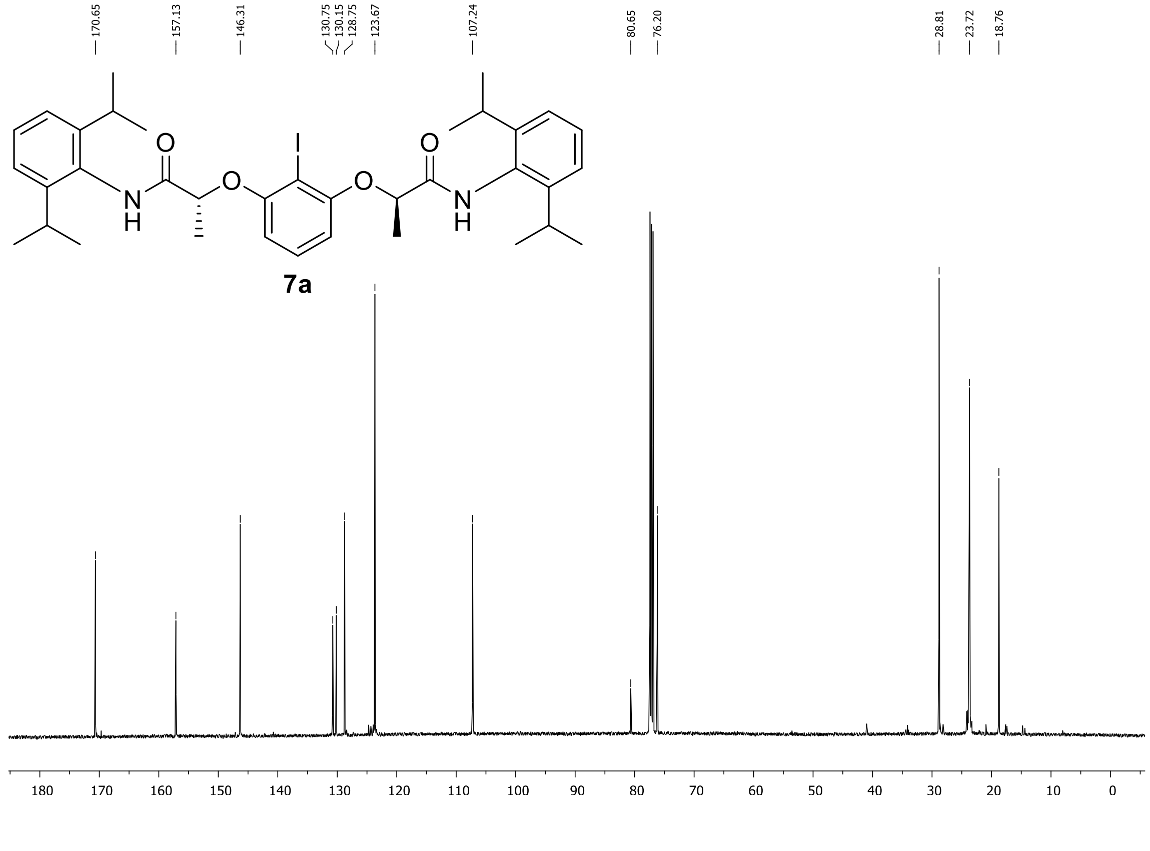

(2*R*,2'*R*)-2,2'-((2-Iodo-1,3-phenylene)bis(oxy))bis(*N*-(2,6-diisopropylphenyl)-3-phenyl-propanamide) (S4d)

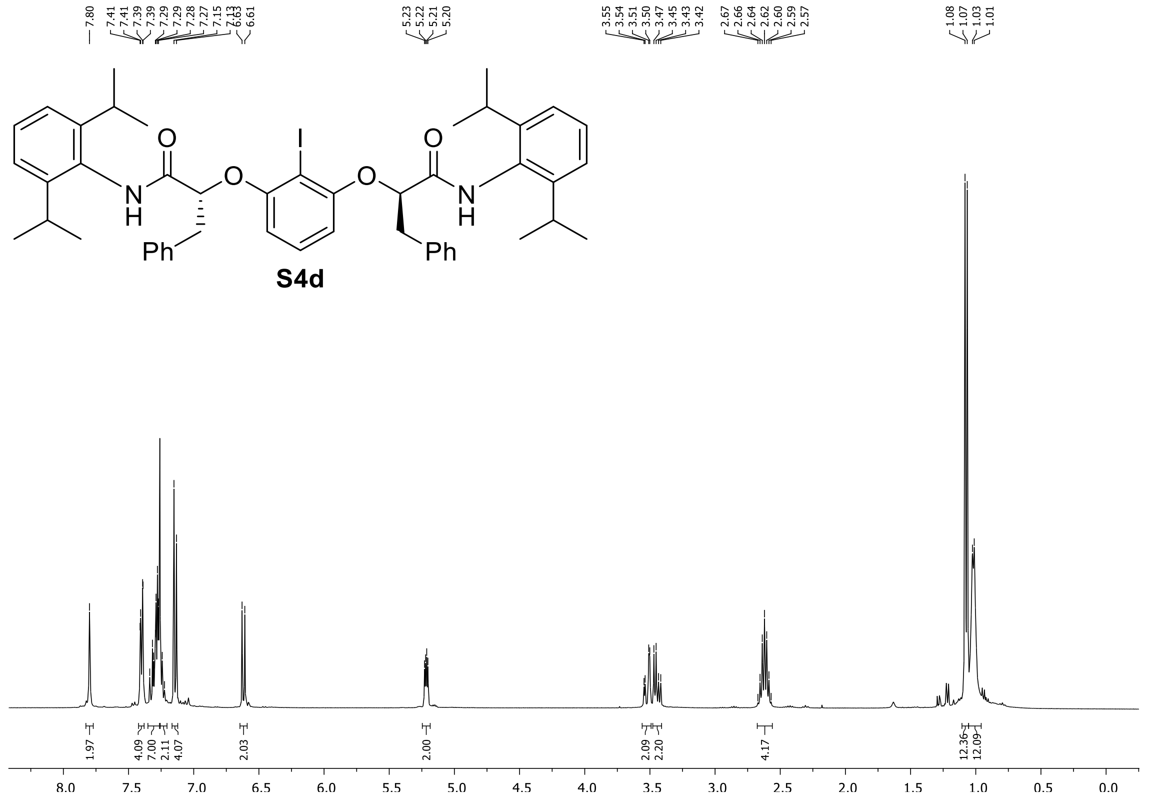

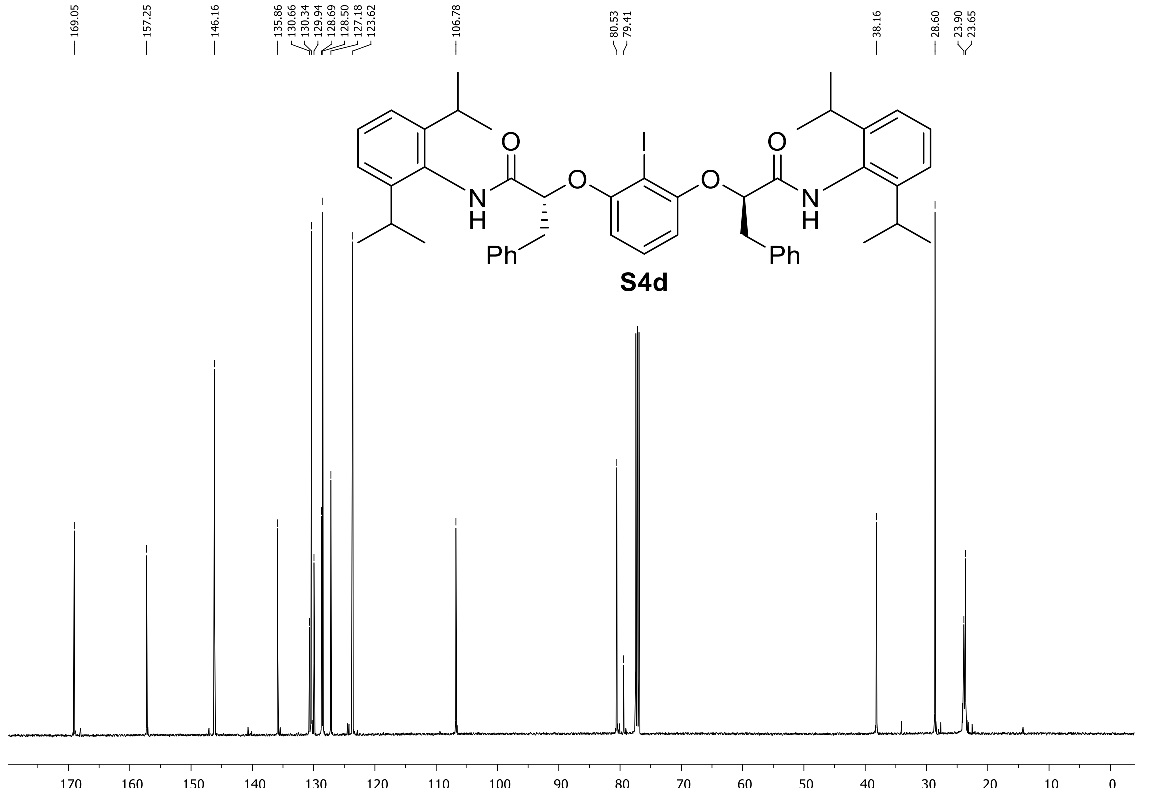

(2*R*,2'*R*)-2,2'-((2-Iodo-1,3-phenylene)bis(oxy))bis(*N,N*-diisopropylpropanamide) (S4e)

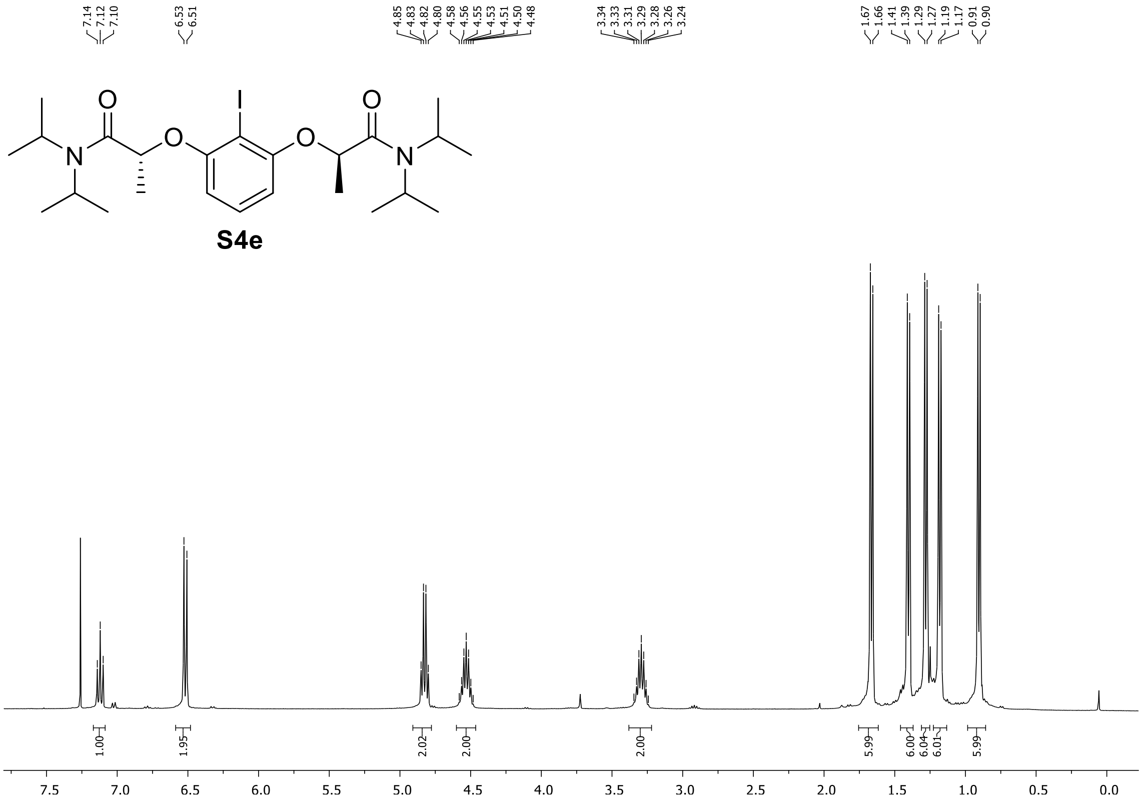

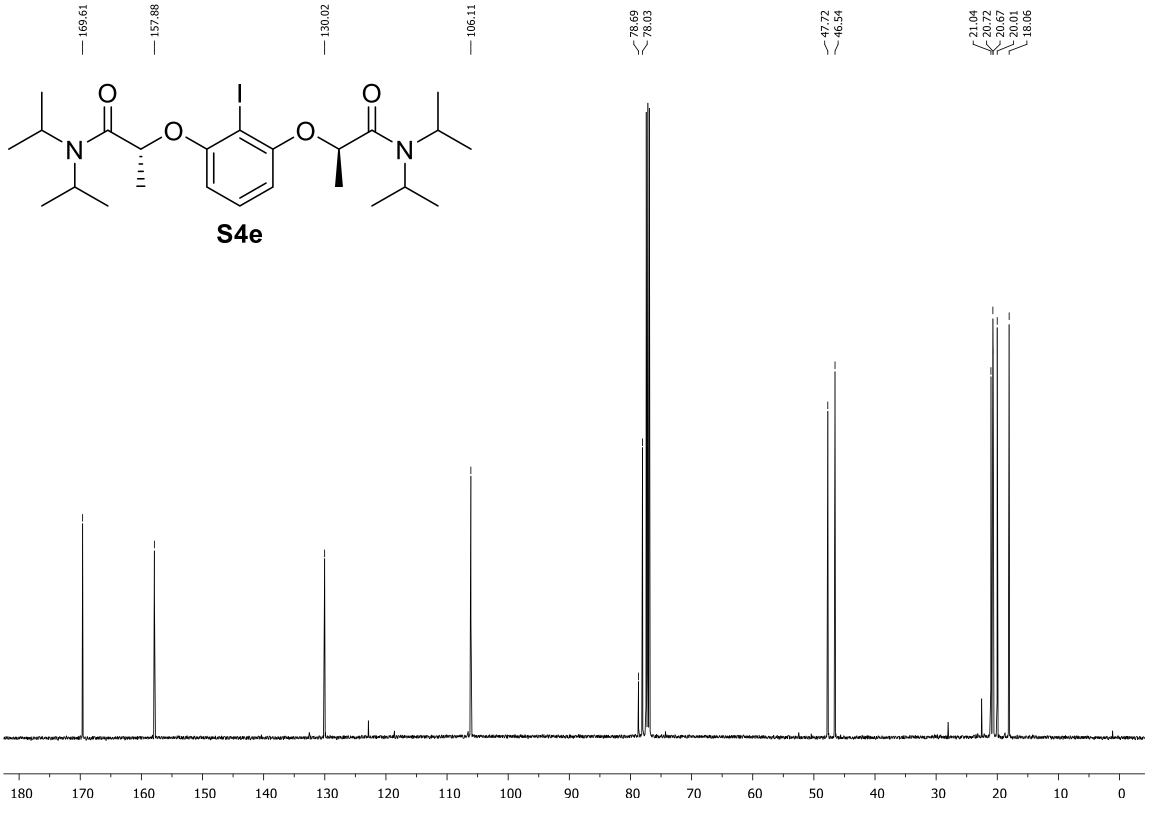

(2*R*,2'*R*)-2,2'-((2-Iodo-5-methyl-1,3-phenylene)bis(oxy))bis(*N*-(2,6-diisopropylphenyl)-propanamide) (7b)

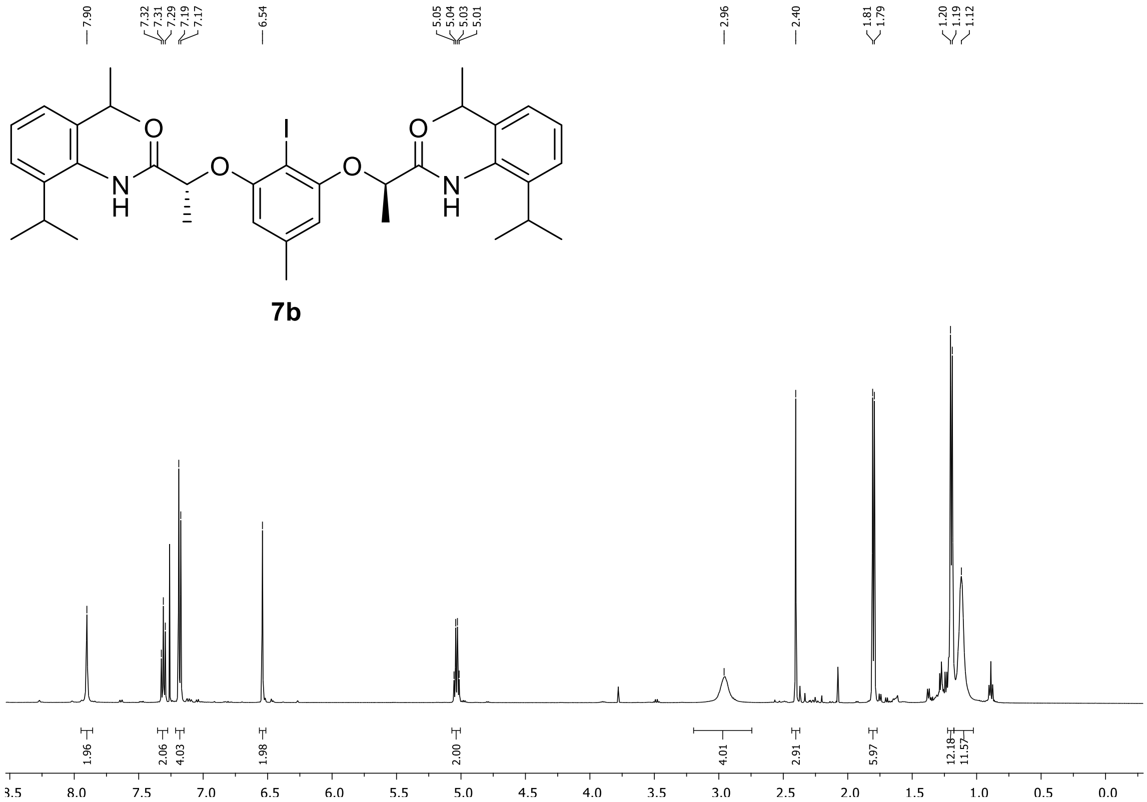

7b

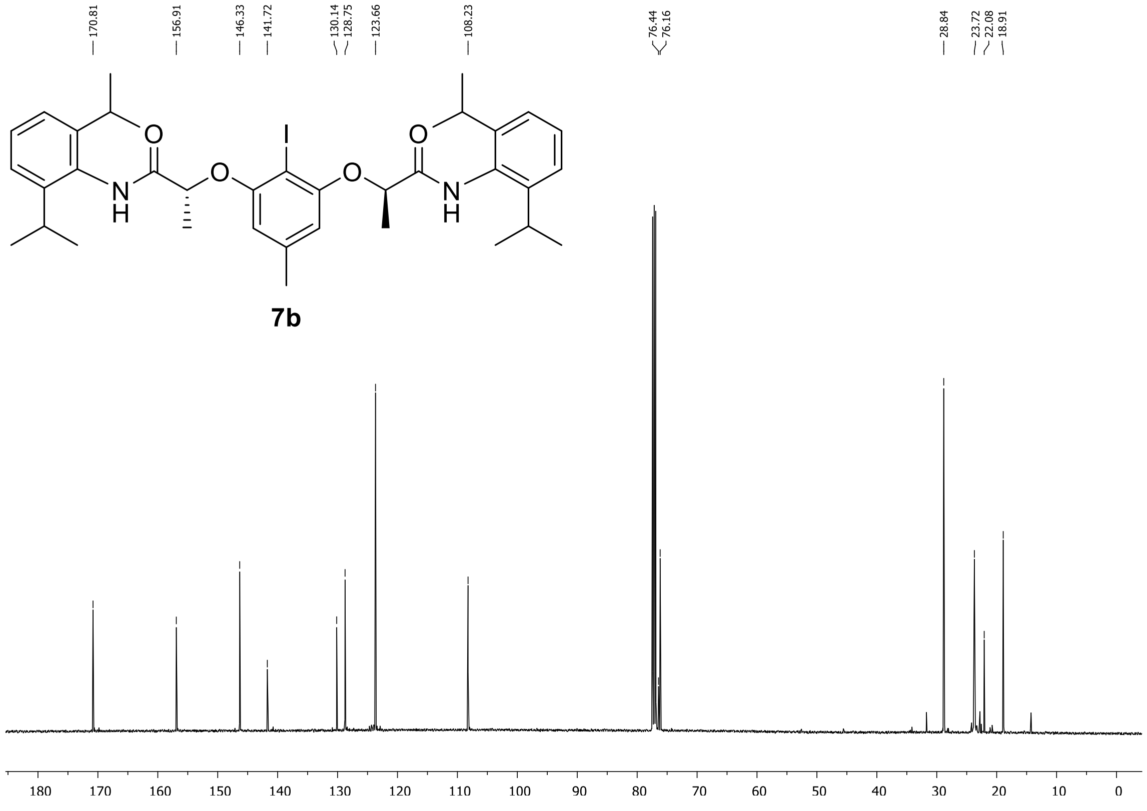

7b

(2*R*,2'*R*)-2,2'-((5-Bromo-2-iodo-1,3-phenylene)bis(oxy))bis(*N*-(2,6-diisopropylphenyl)-propanamide) (7c)

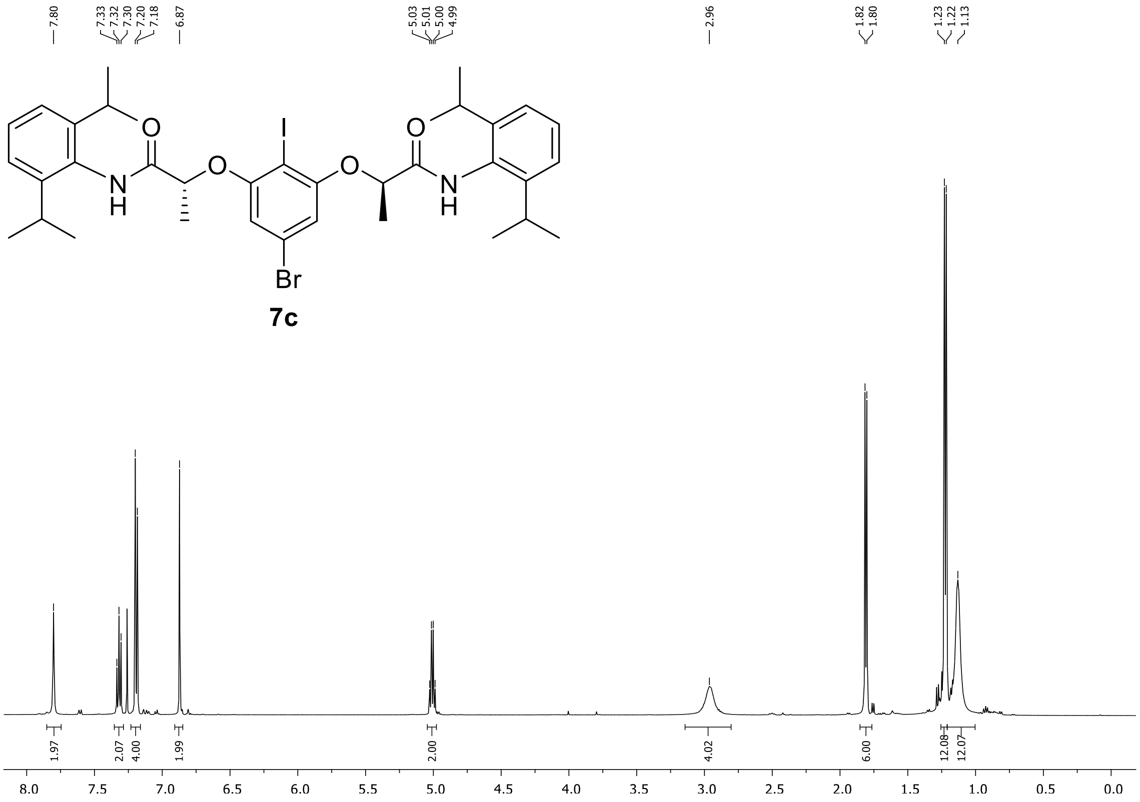

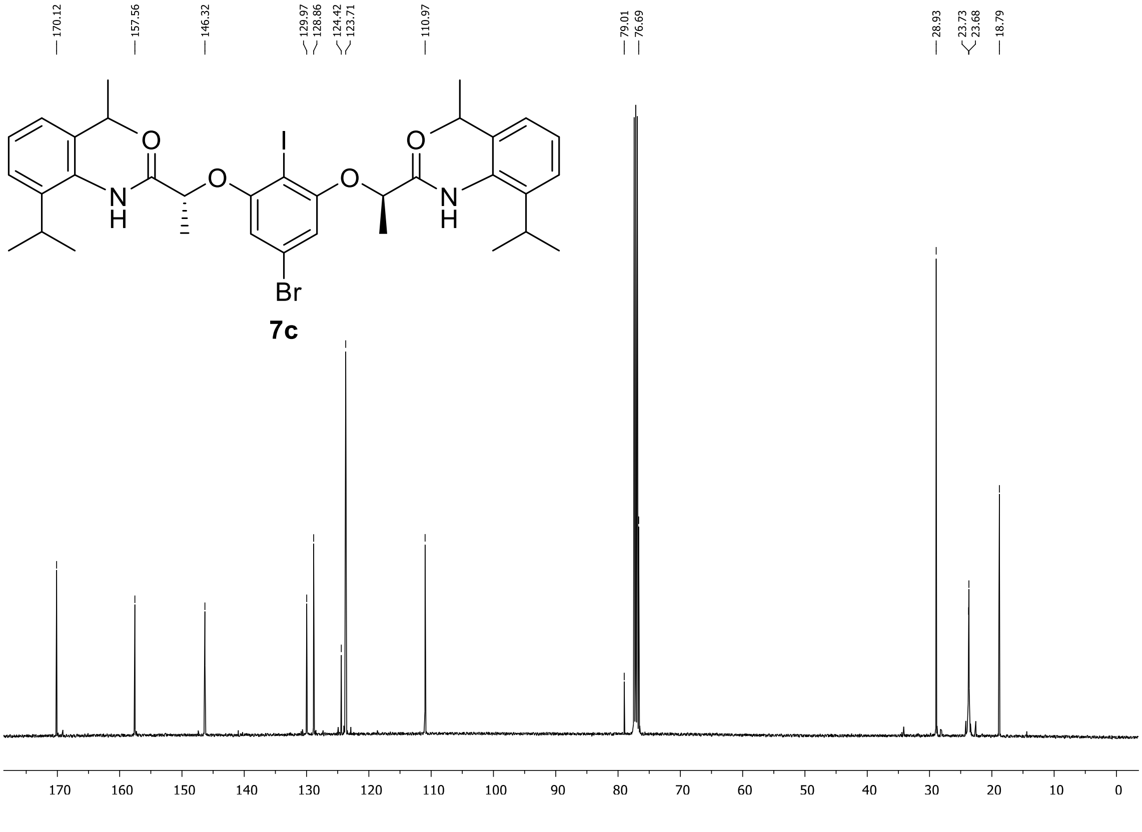

(2*R*,2'*R*)-2,2'-((5-Acetyl-2-iodo-1,3-phenylene)bis(oxy))bis(*N*-(2,6-diisopropylphenyl)-propanamide) (7d)

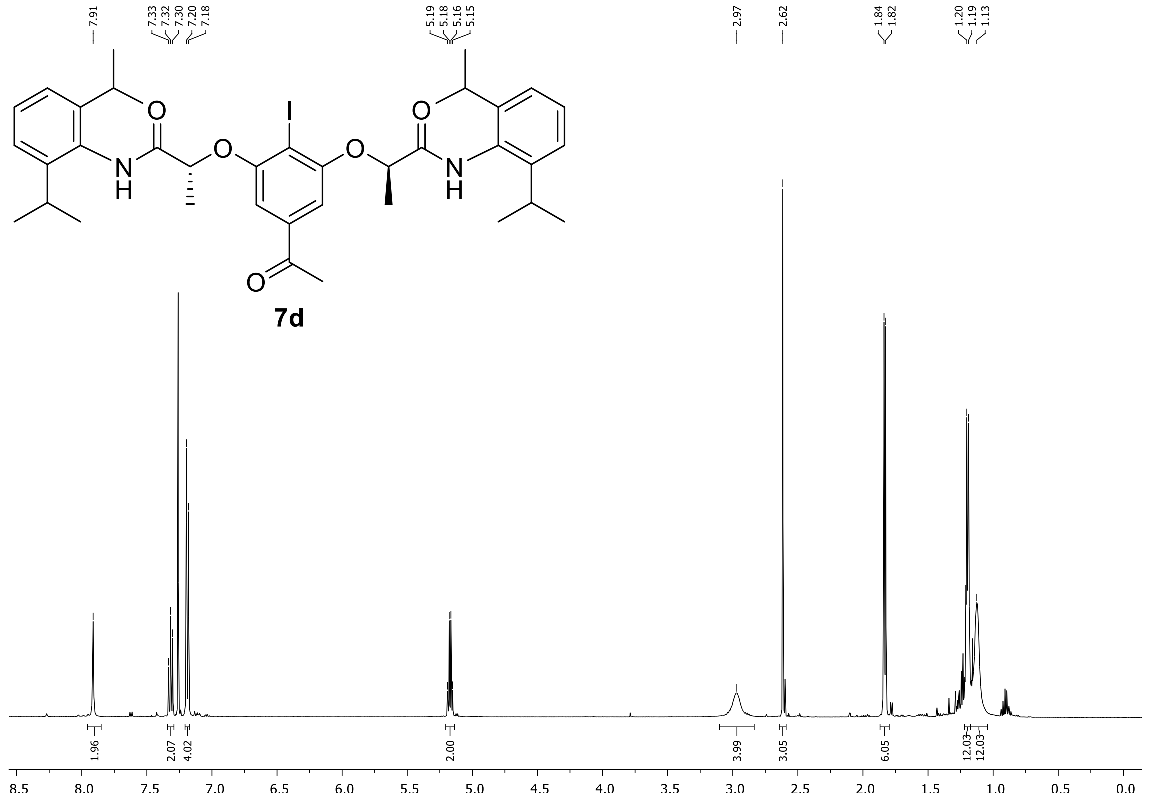

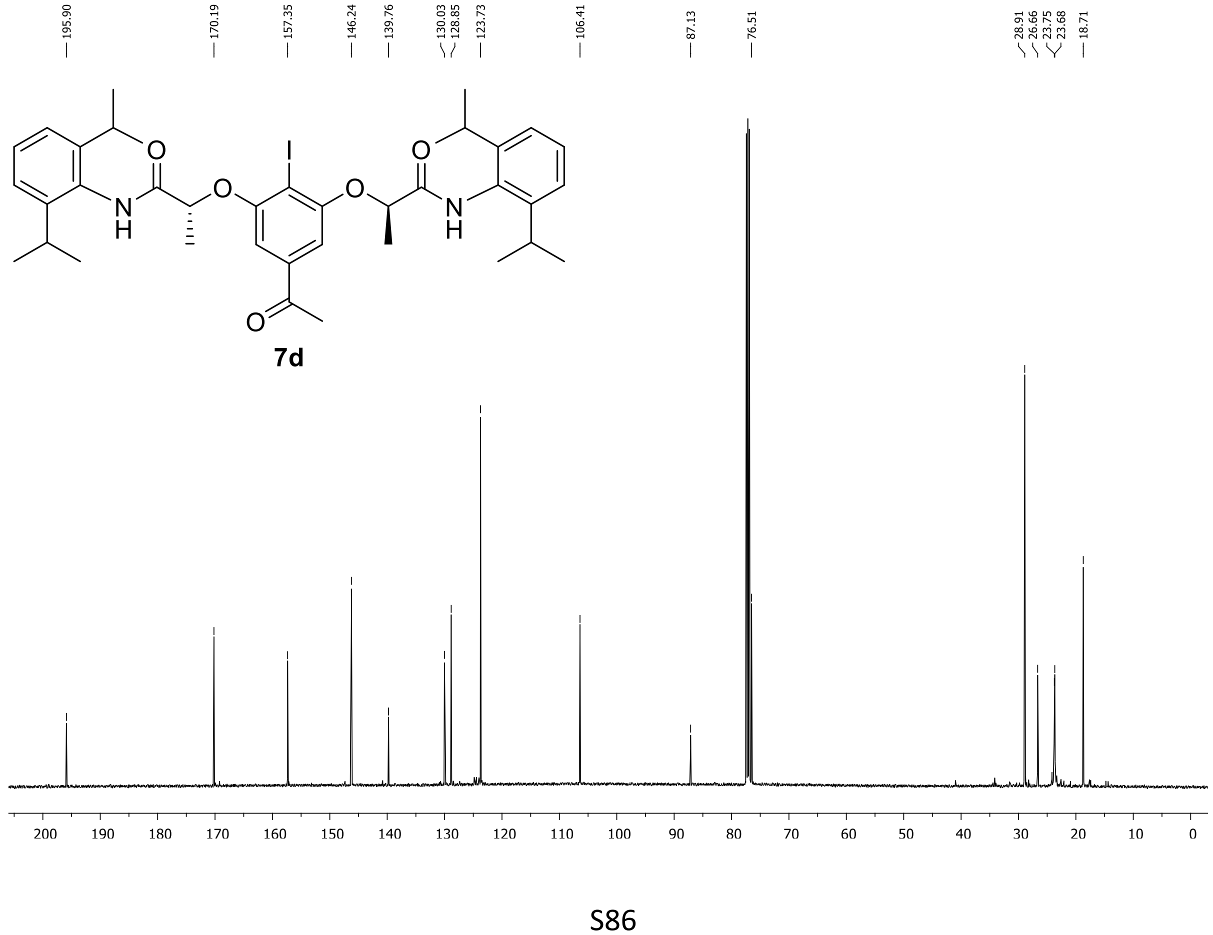

Dimethyl 2,2'-((2-(diacetoxy-λ^3-iodaneyl)-1,3-phenylene)bis(oxy))(2*R*,2'*R*)-dipropionate (6a)

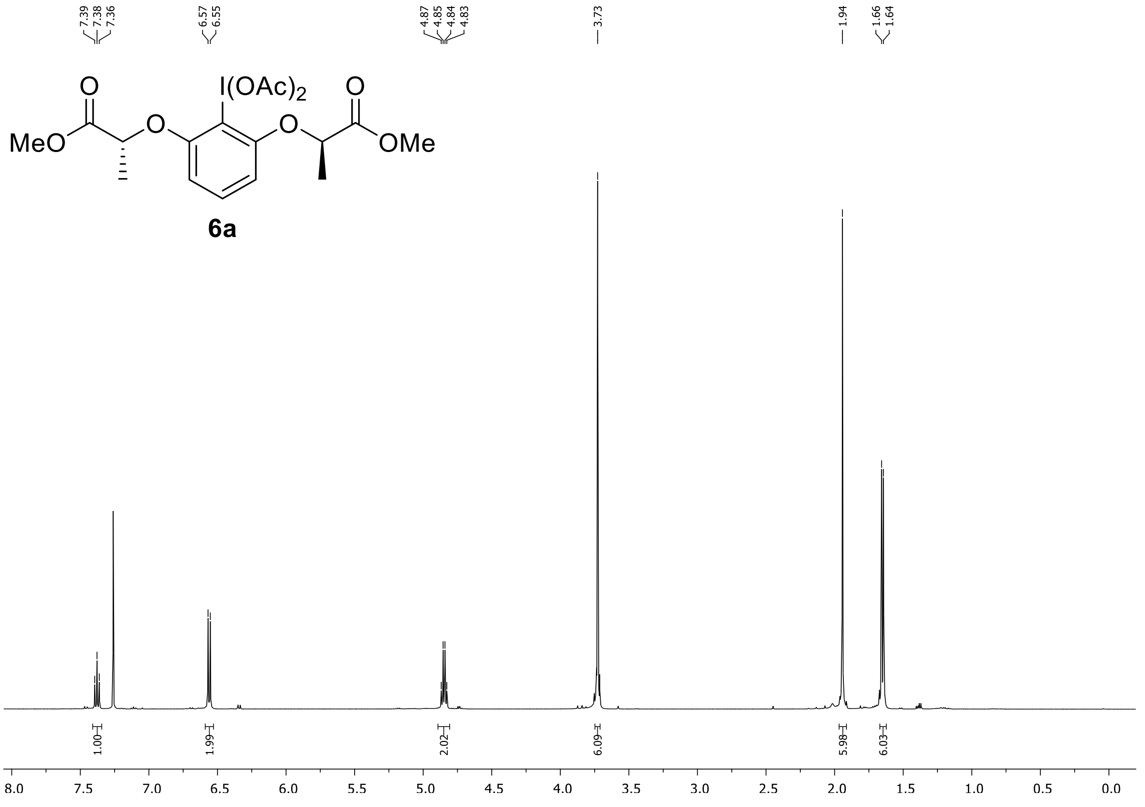

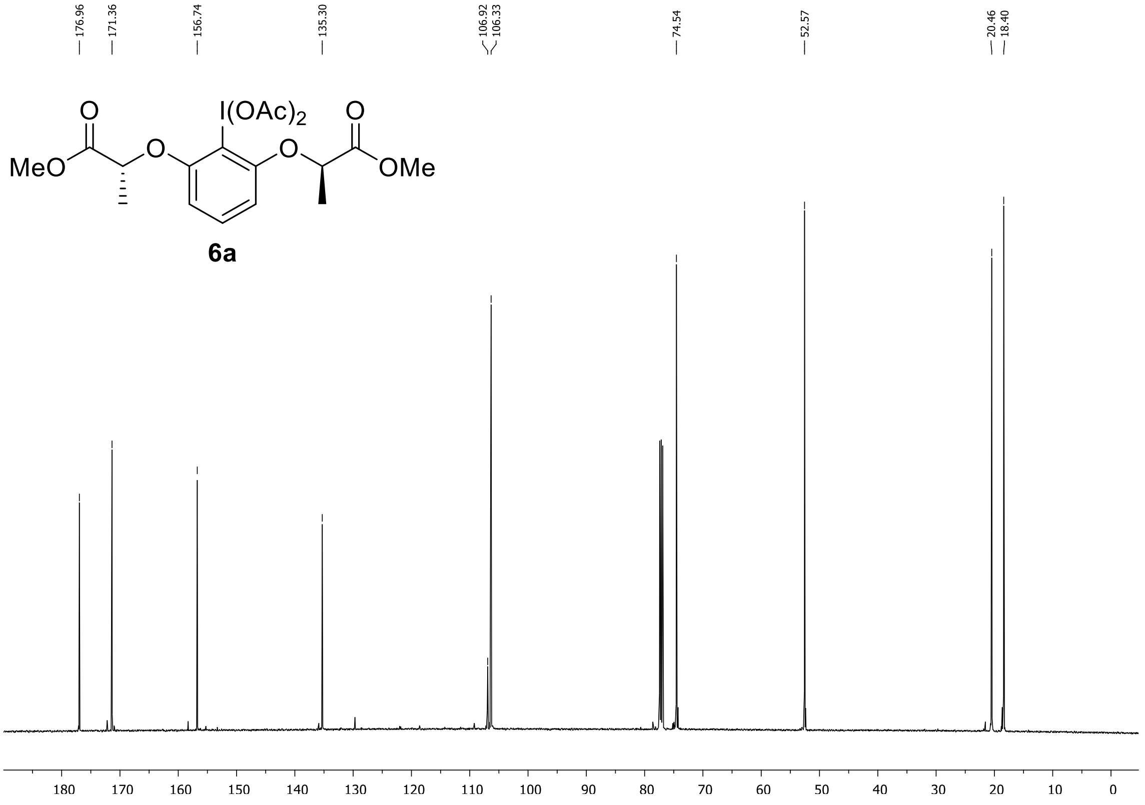

(2,6-Bis(((*R*)-1-(methylamino)-1-oxopropan-2-yl)oxy)phenyl)-λ^3-iodanediyl diacetate (6b)

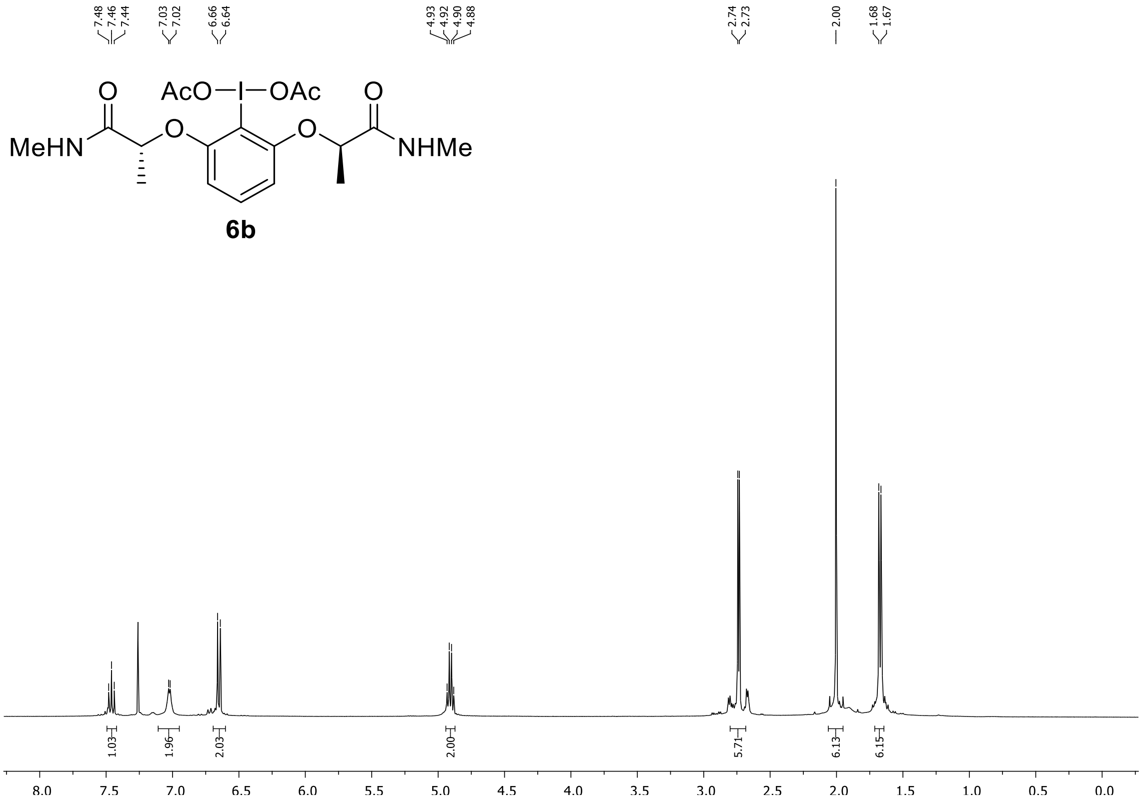

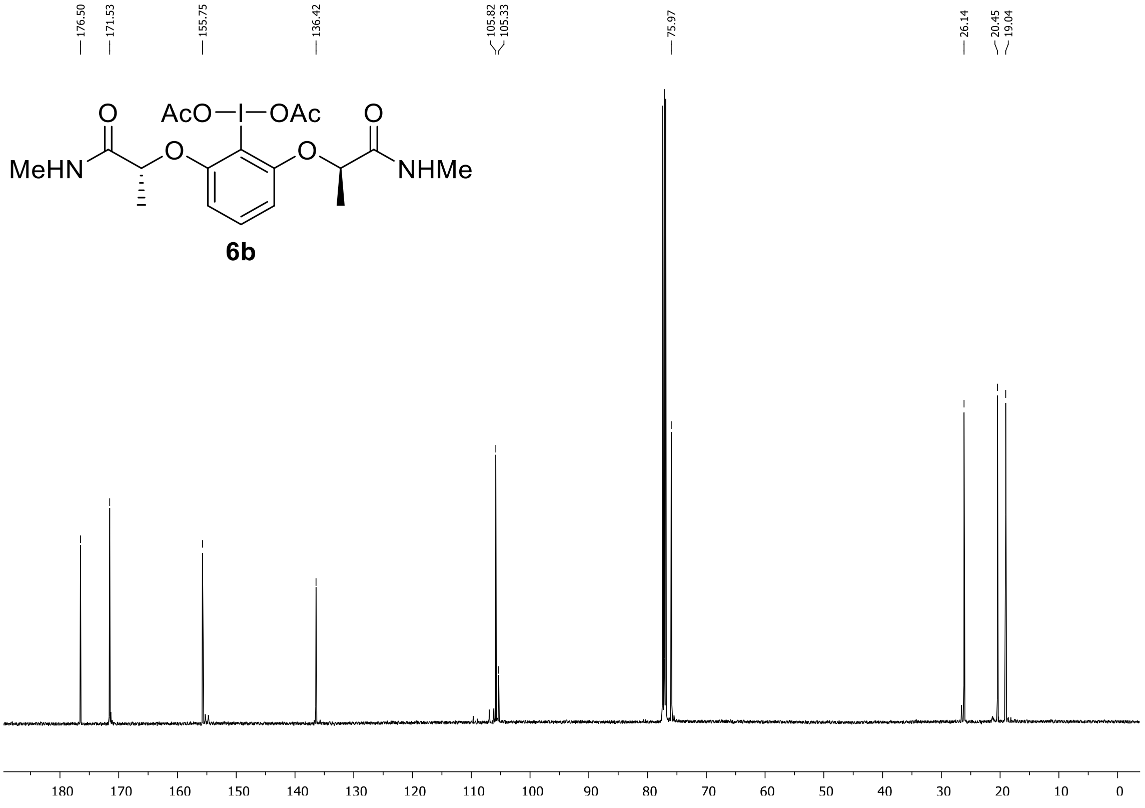

Dimesityl 2,2'-((2-(diacetoxy-λ^3-iodaneyl)-1,3-phenylene)bis(oxy))(2*R*,2'*R*)-dipropionate (6c)

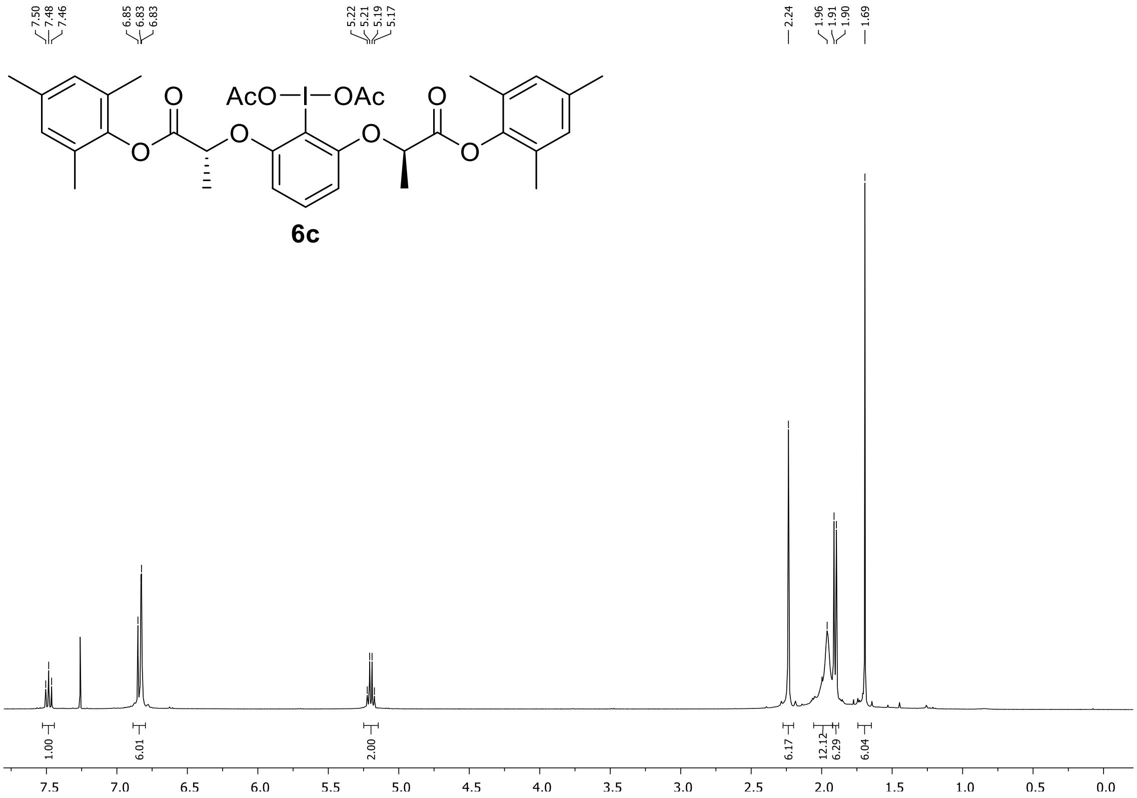

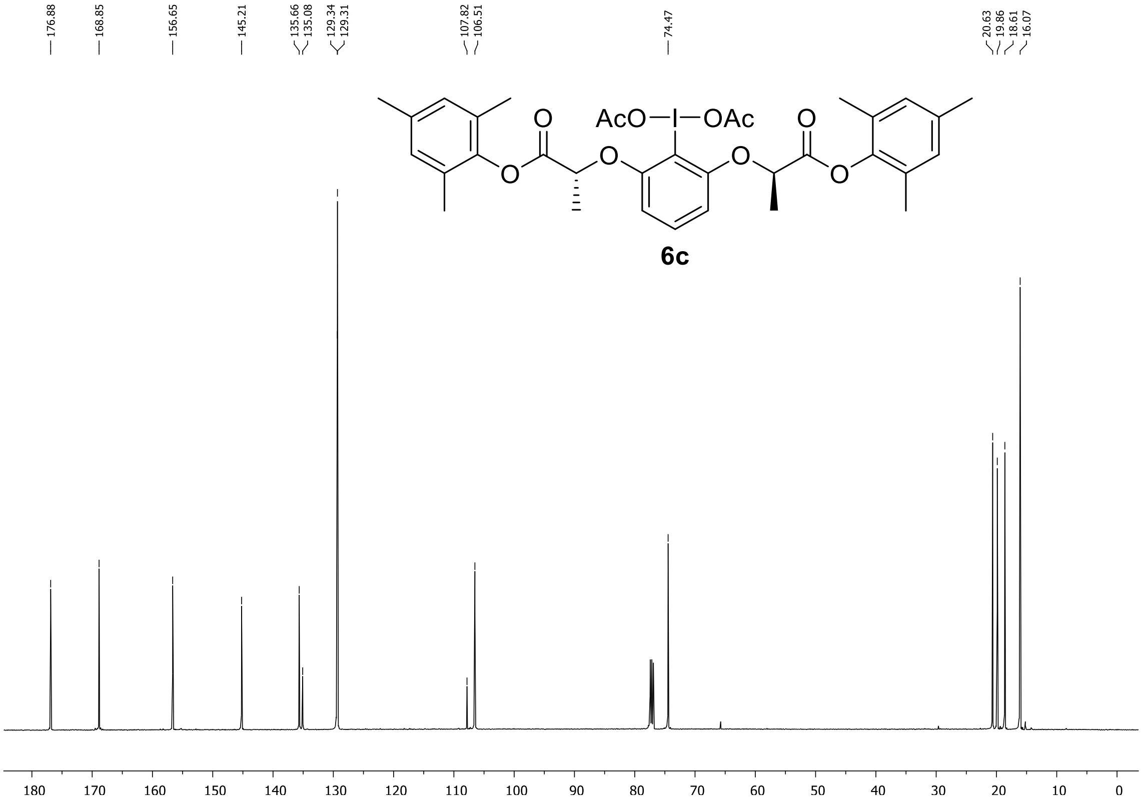

(2,6-Bis(((*R*)-1-(mesitylamino)-1-oxopropan-2-yl)oxy)phenyl)-λ^3-iodanediyl diacetate (6d)

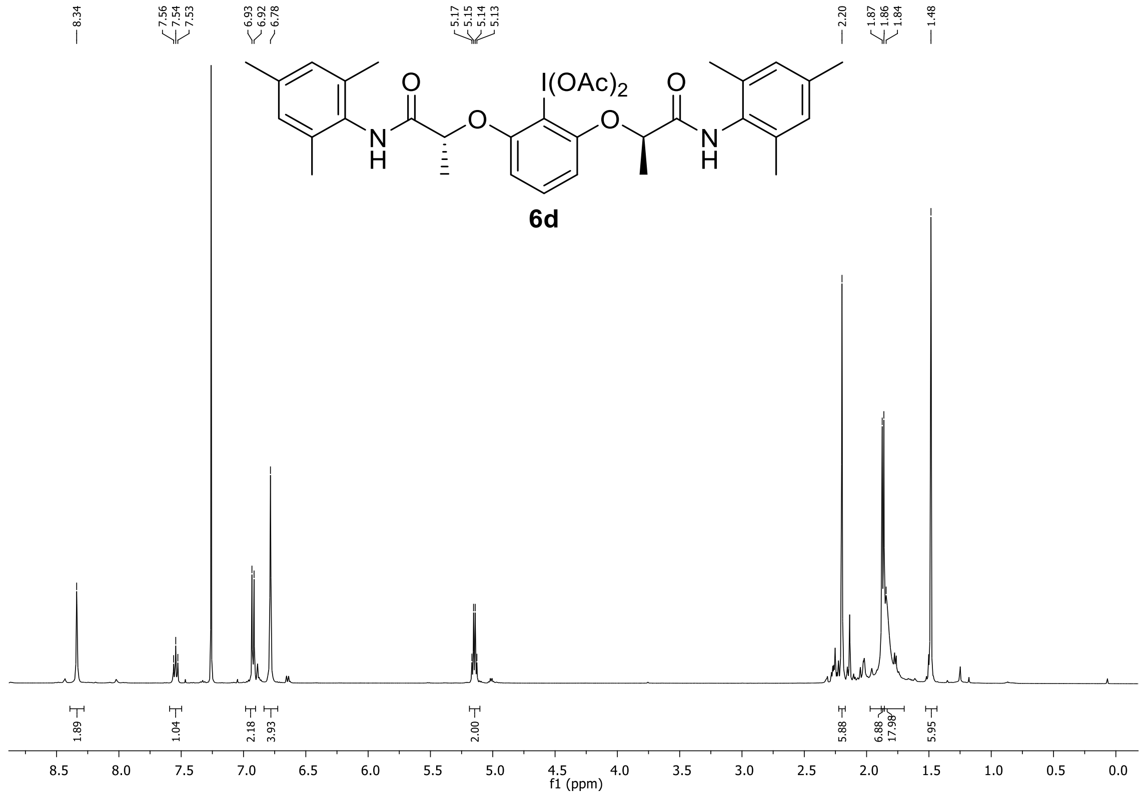

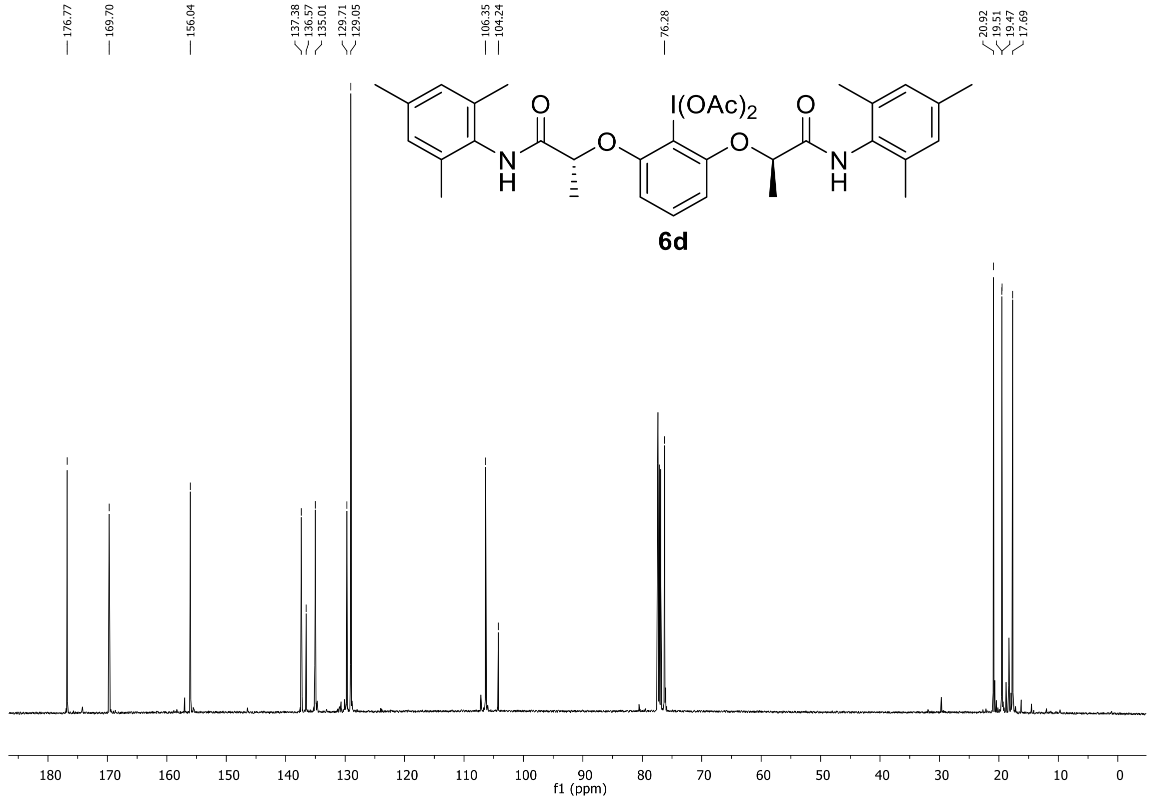

(2,6-Bis(((*R*)-1-((2,6-diisopropylphenyl)amino)-1-oxopropan-2-yl)oxy)phenyl)-λ^3-iodanediyl diacetate (6e)

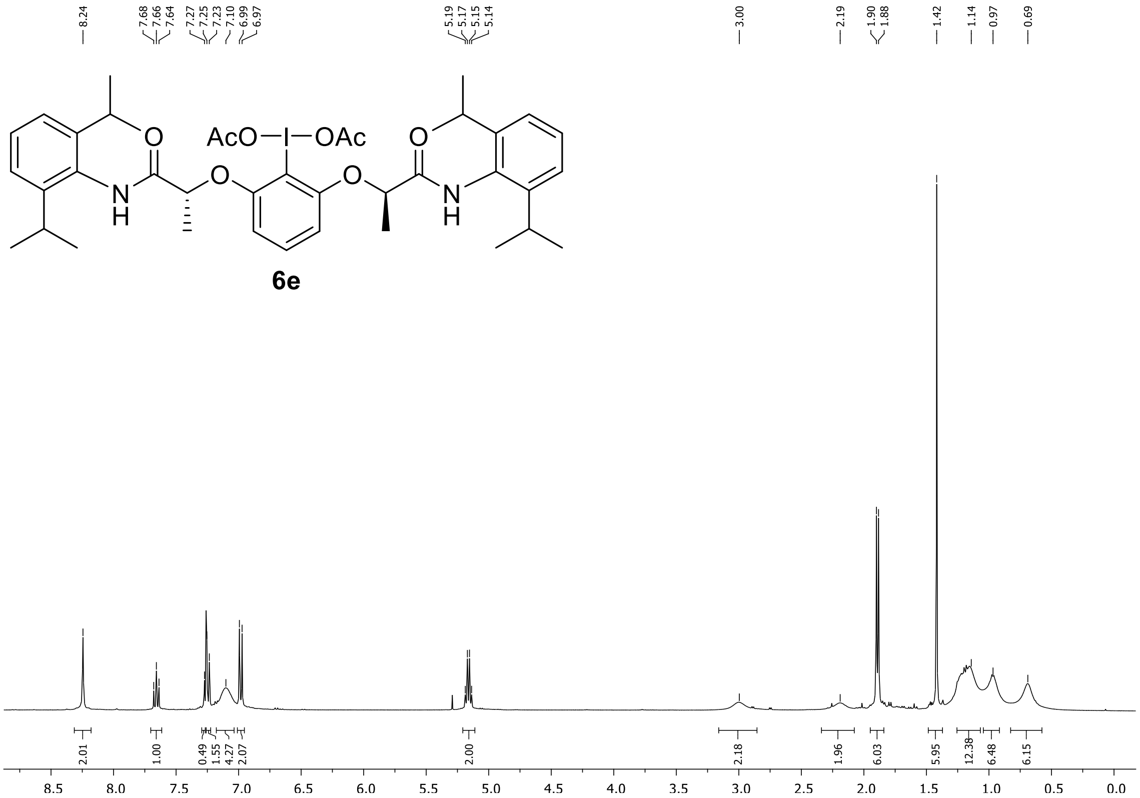

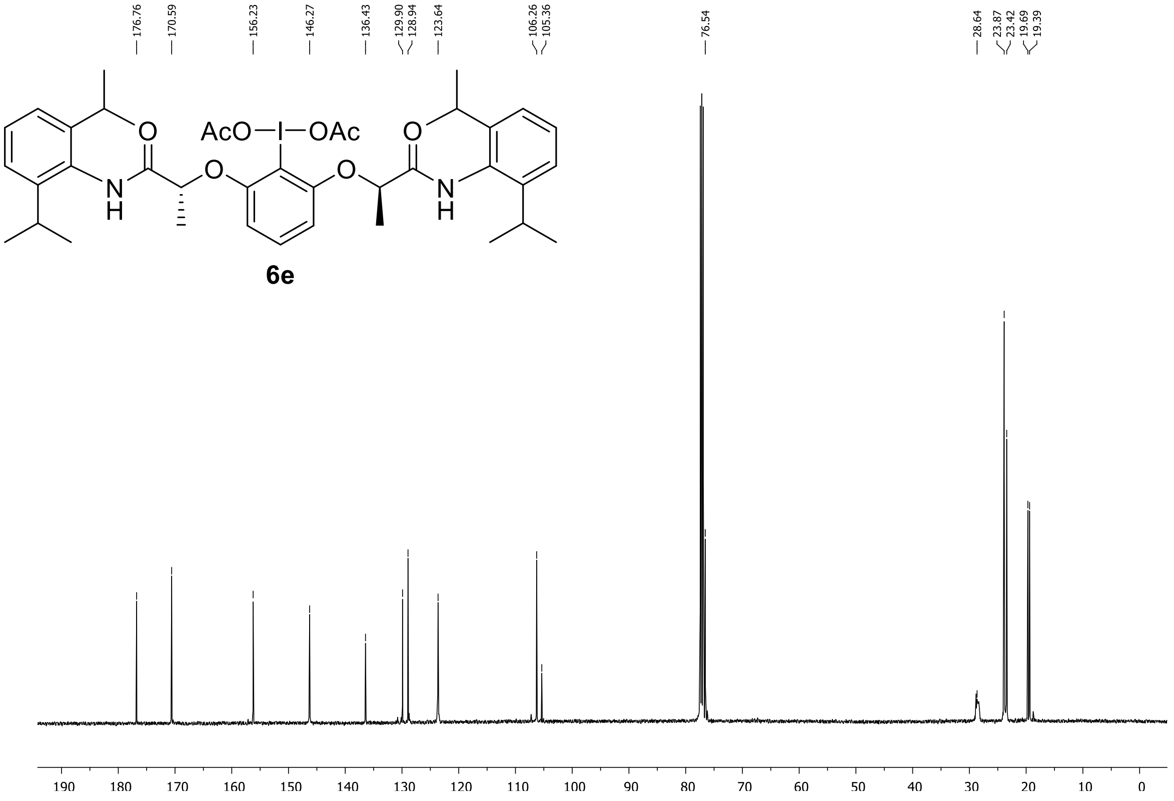

(2,6-Bis(((*R*)-1-((2,6-diisopropylphenyl)amino)-1-oxo-3-phenylpropan-2-yl)oxy)phenyl)-λ^3-iodanediyl diacetate (6f)

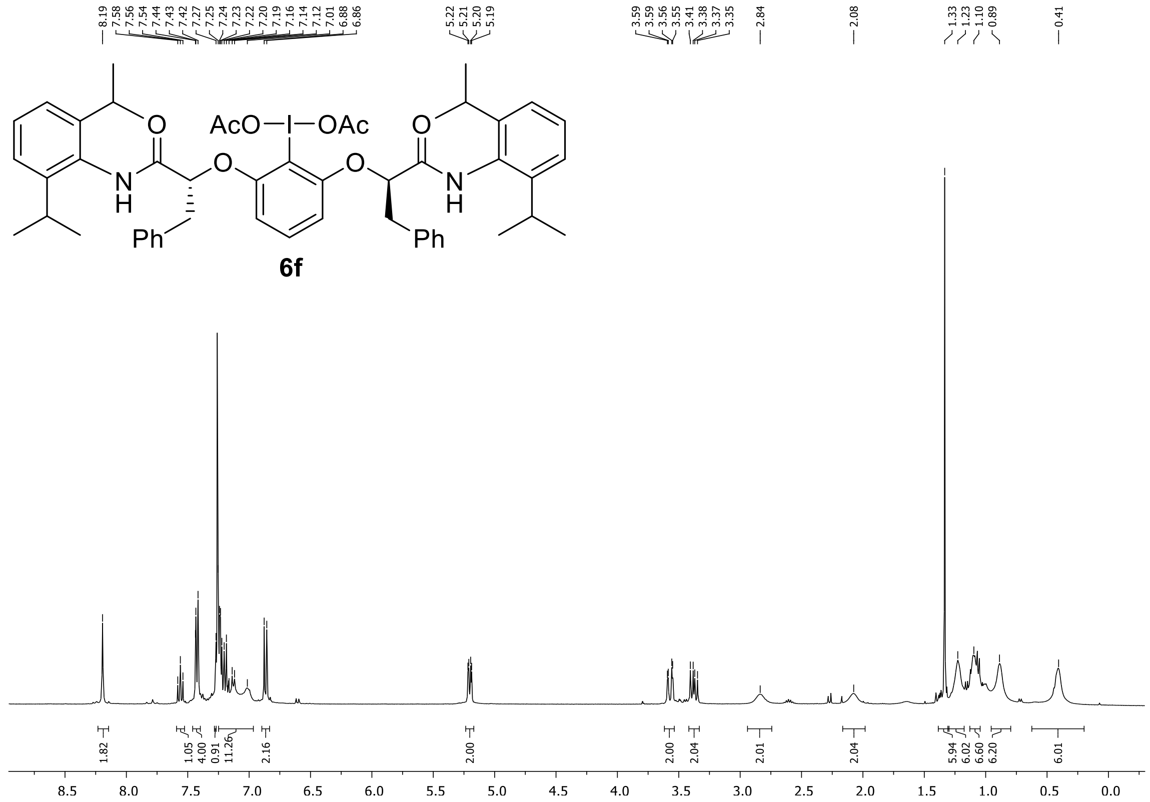

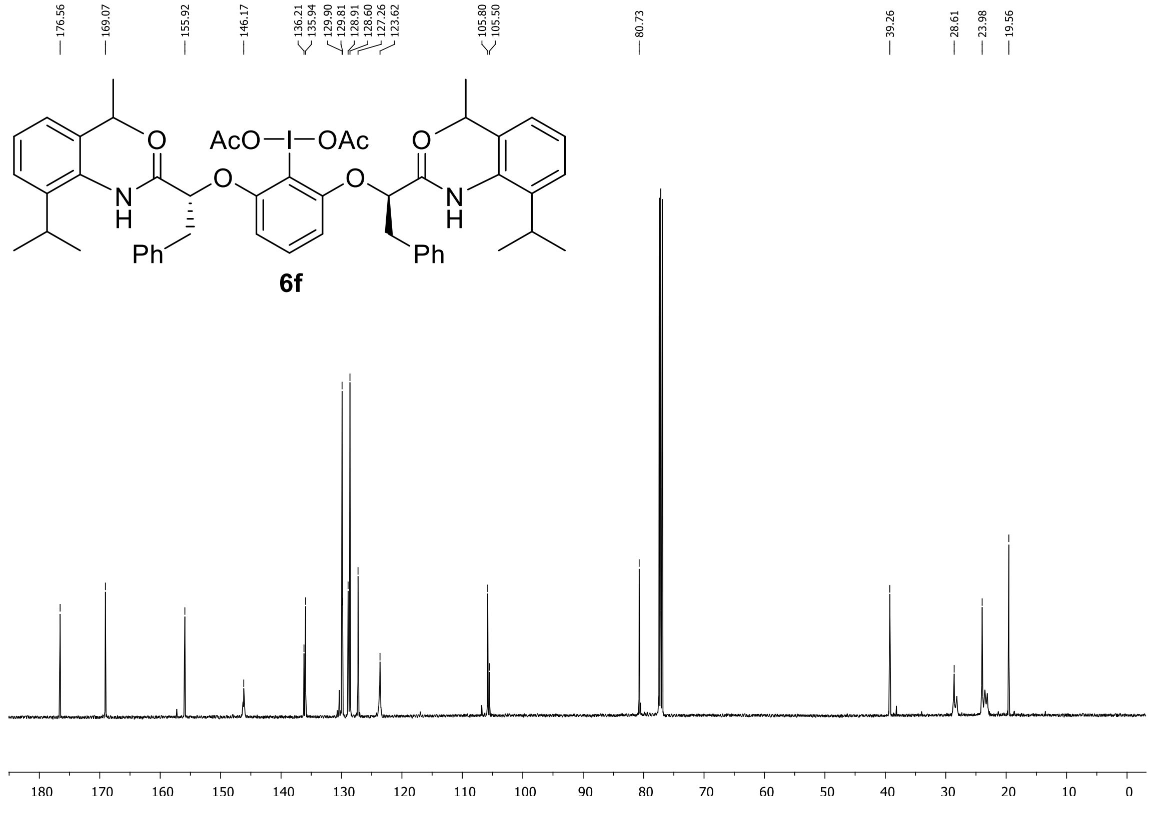

(2,6-Bis(((*R*)-1-(diisopropylamino)-1-oxopropan-2-yl)oxy)phenyl)-λ^3-iodanediyl diacetate (6g)

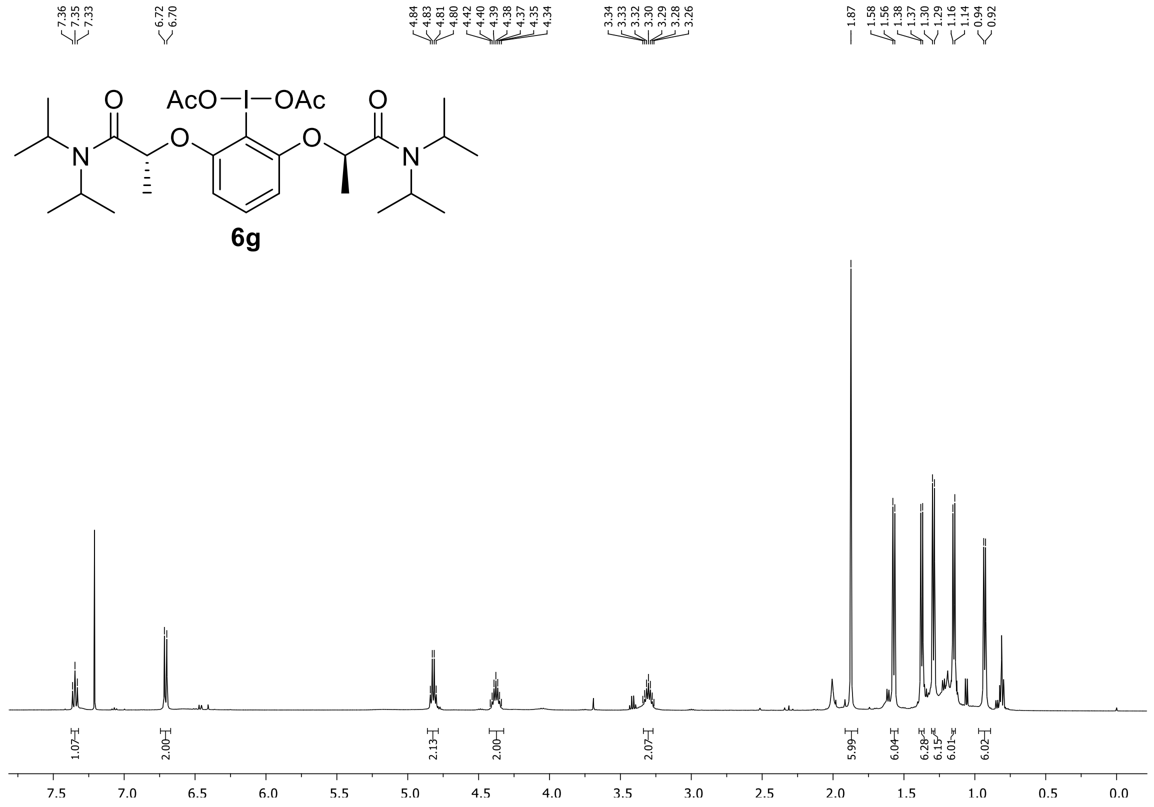

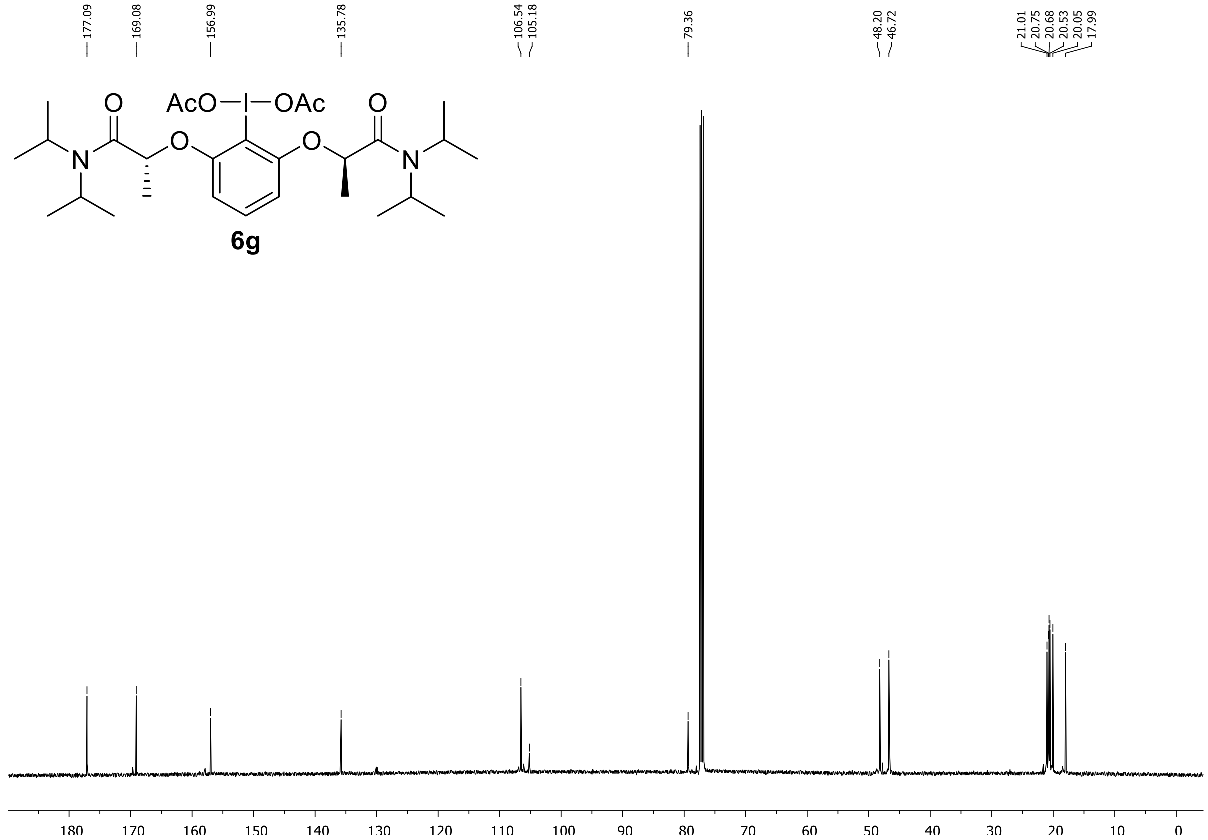

(2,6-Bis(((*R*)-1-((2,6-diisopropylphenyl)amino)-1-oxopropan-2-yl)oxy)-4-methylphenyl)-*λ*^{3}-iodanediyl diacetate (6h)

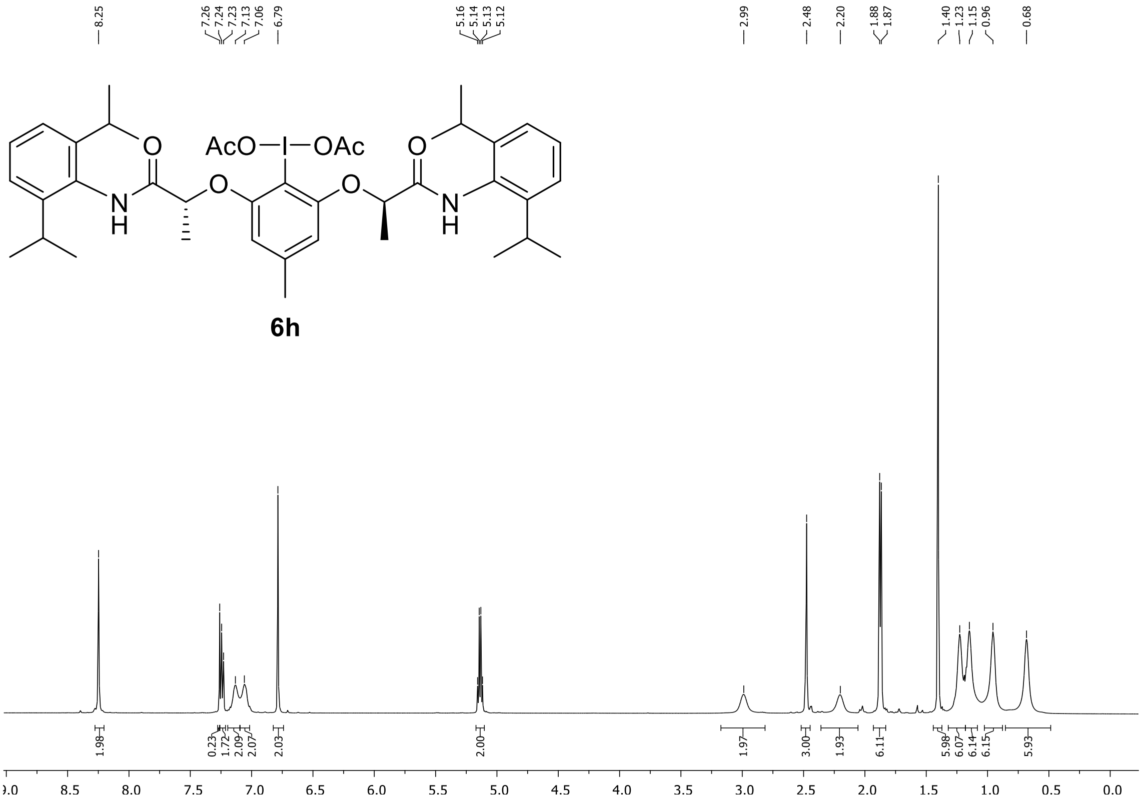

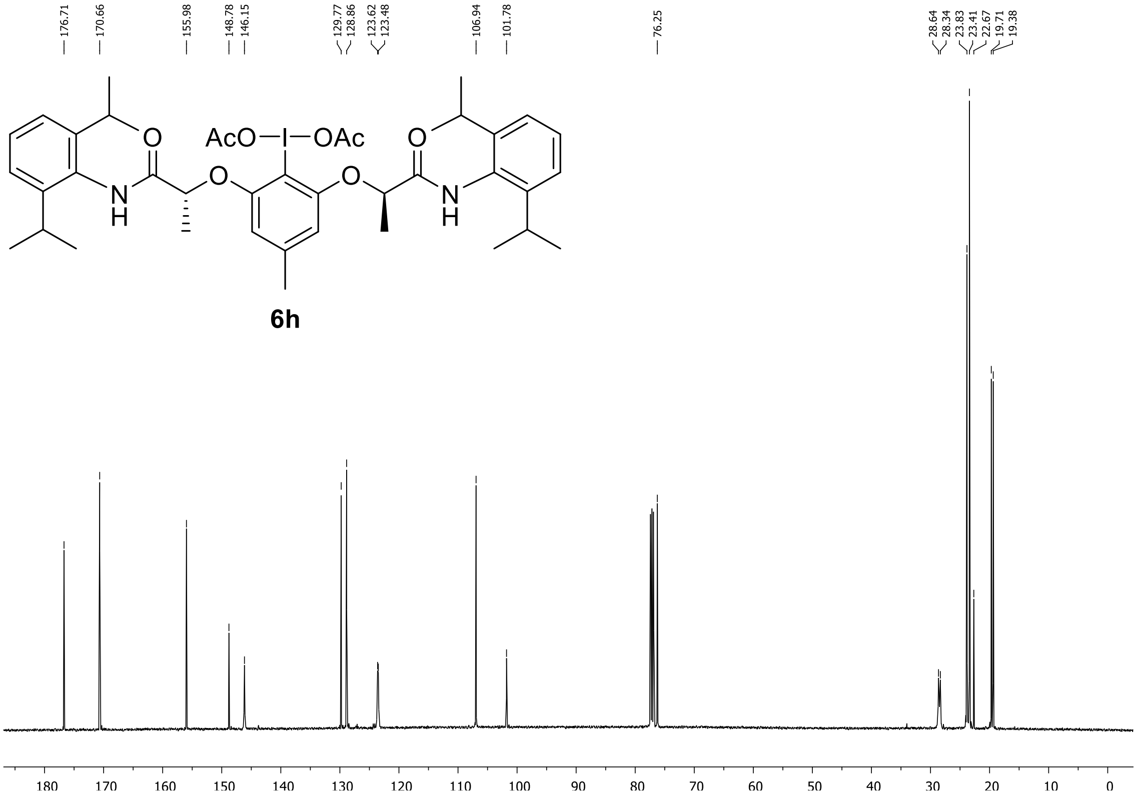

(4-Bromo-2,6-bis(((*R*)-1-((2,6-diisopropylphenyl)amino)-1-oxopropan-2-yl)oxy)phenyl)-λ^3-iodanediyl diacetate (6i)

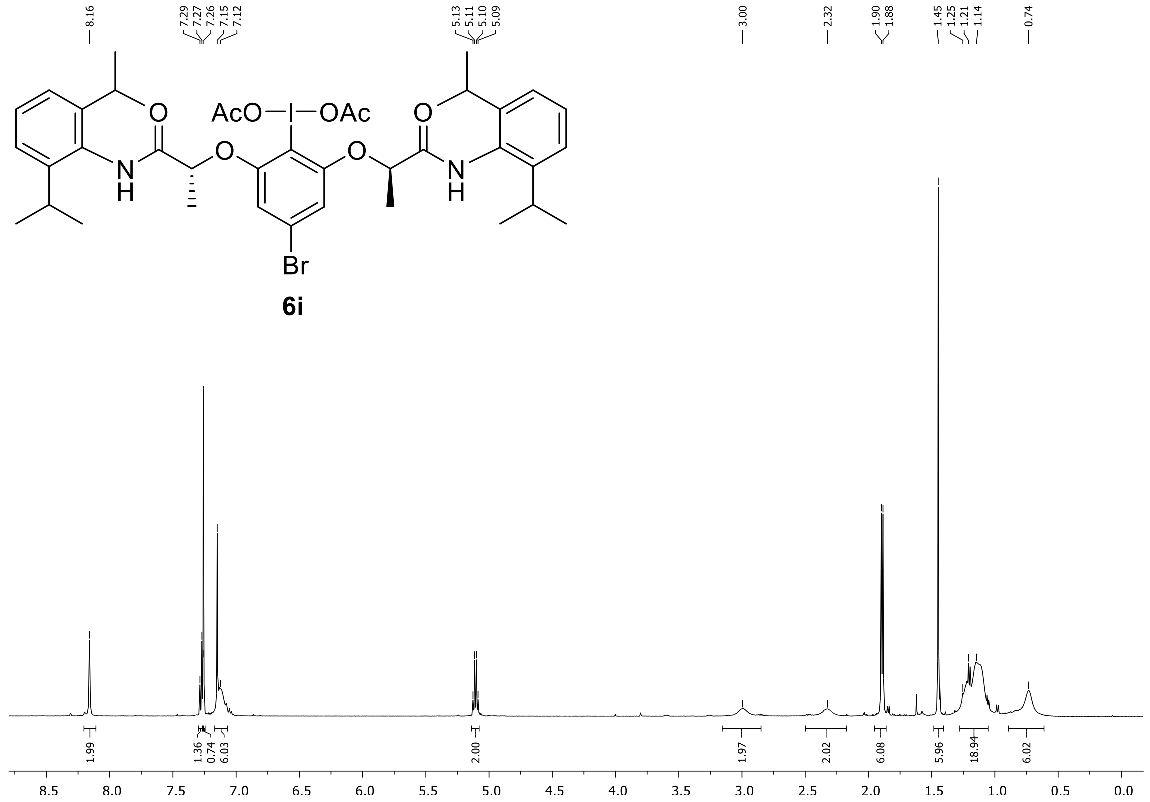

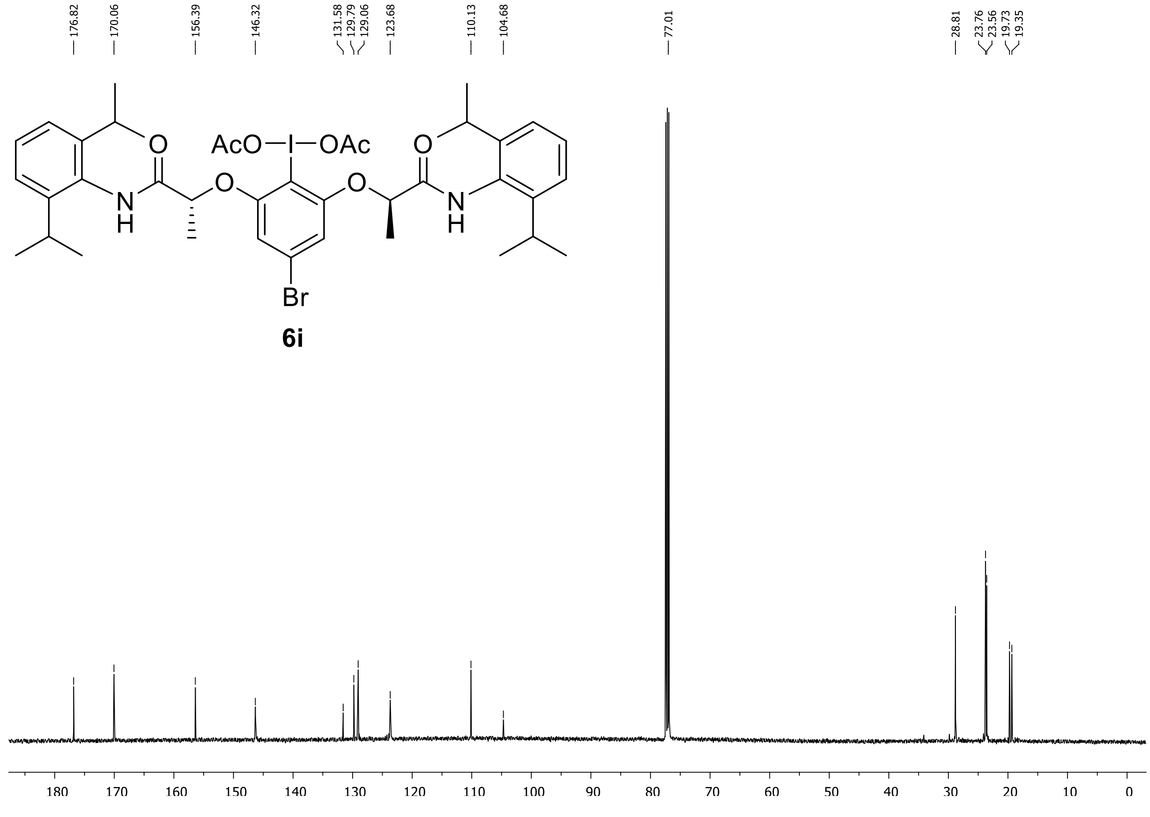

(4-Acetyl-2,6-bis(((*R*)-1-((2,6-diisopropylphenyl)amino)-1-oxopropan-2-yl)oxy)phenyl)-λ^3-iodanediyl diacetate (6j)

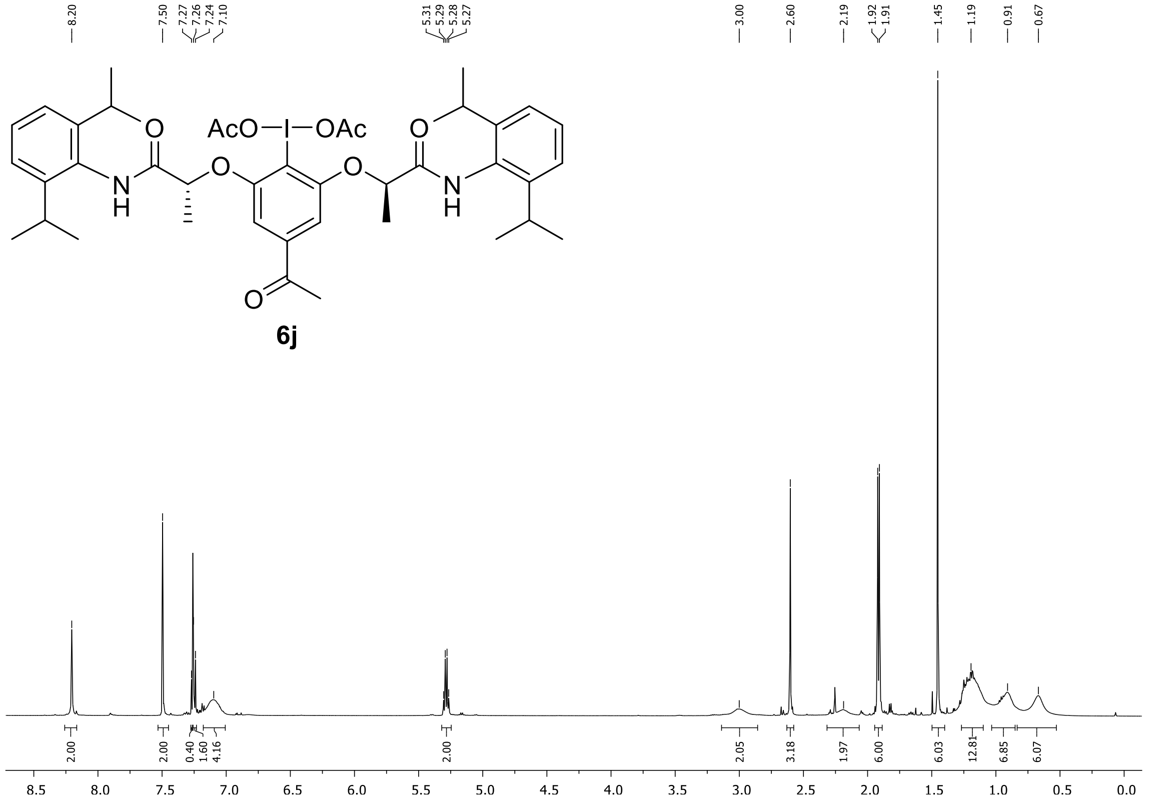

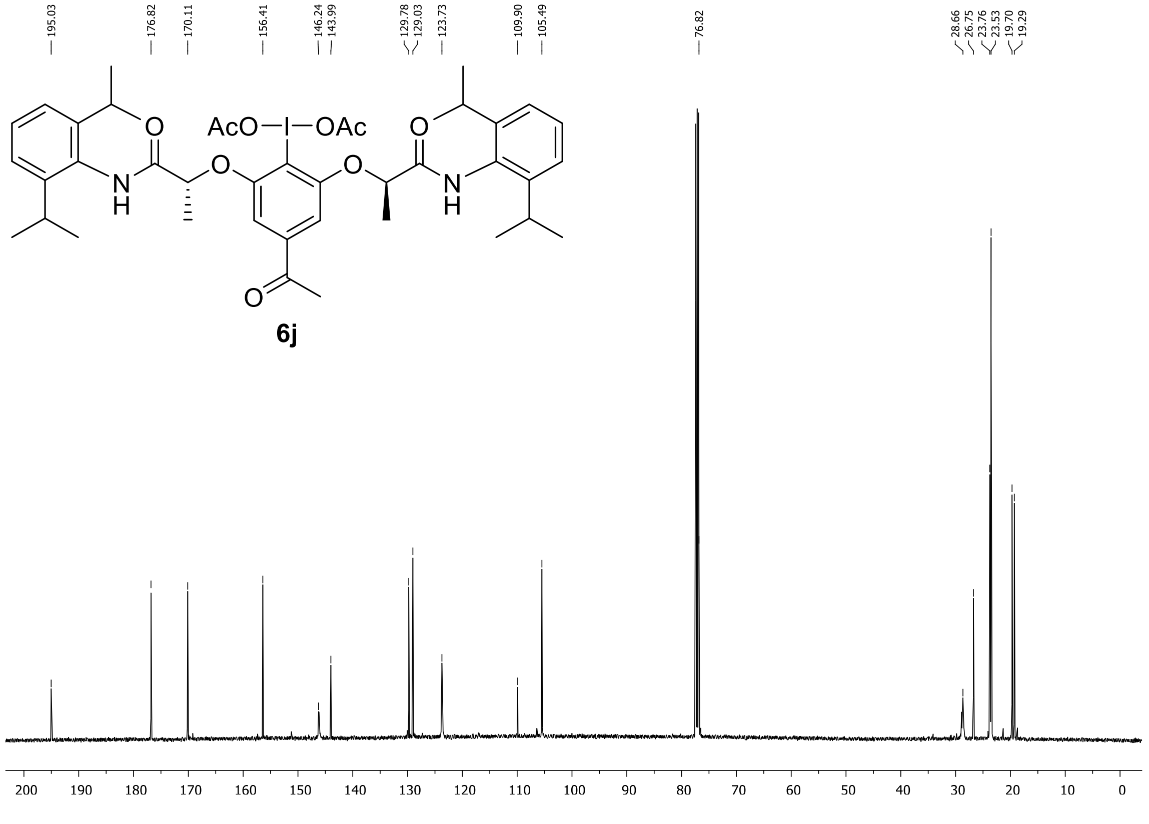

(*R*)-1-Oxo-1-phenylpropan-2-yl acetate ((*R*)-5a)

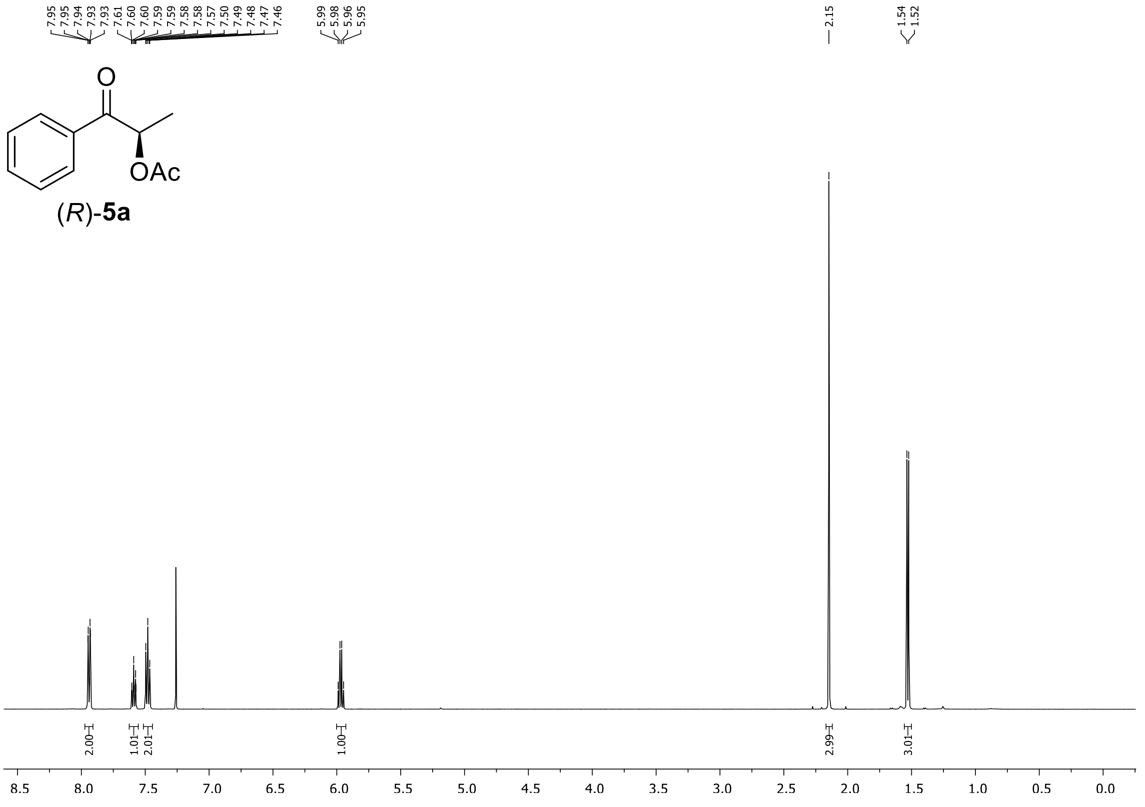

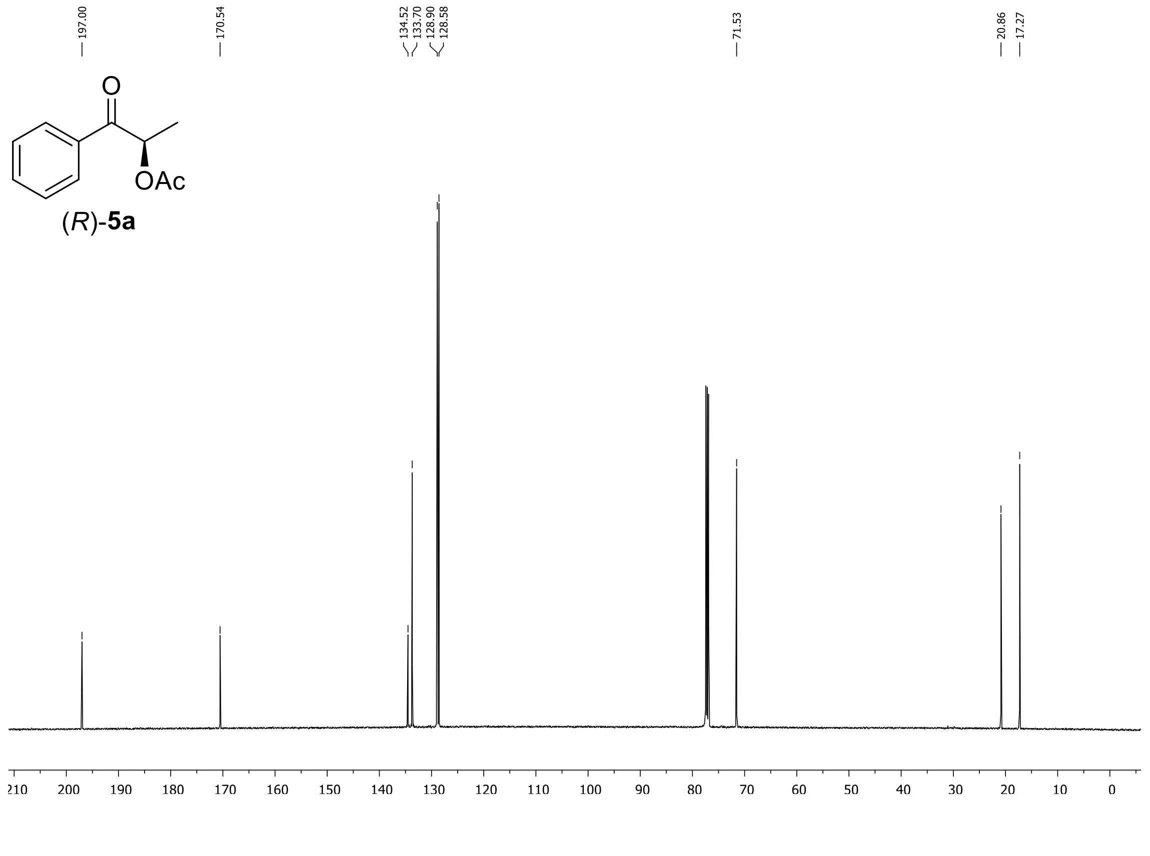

(*R*)-1-(4-Fluorophenyl)-1-oxopropan-2-yl acetate ((*R*)-5b)

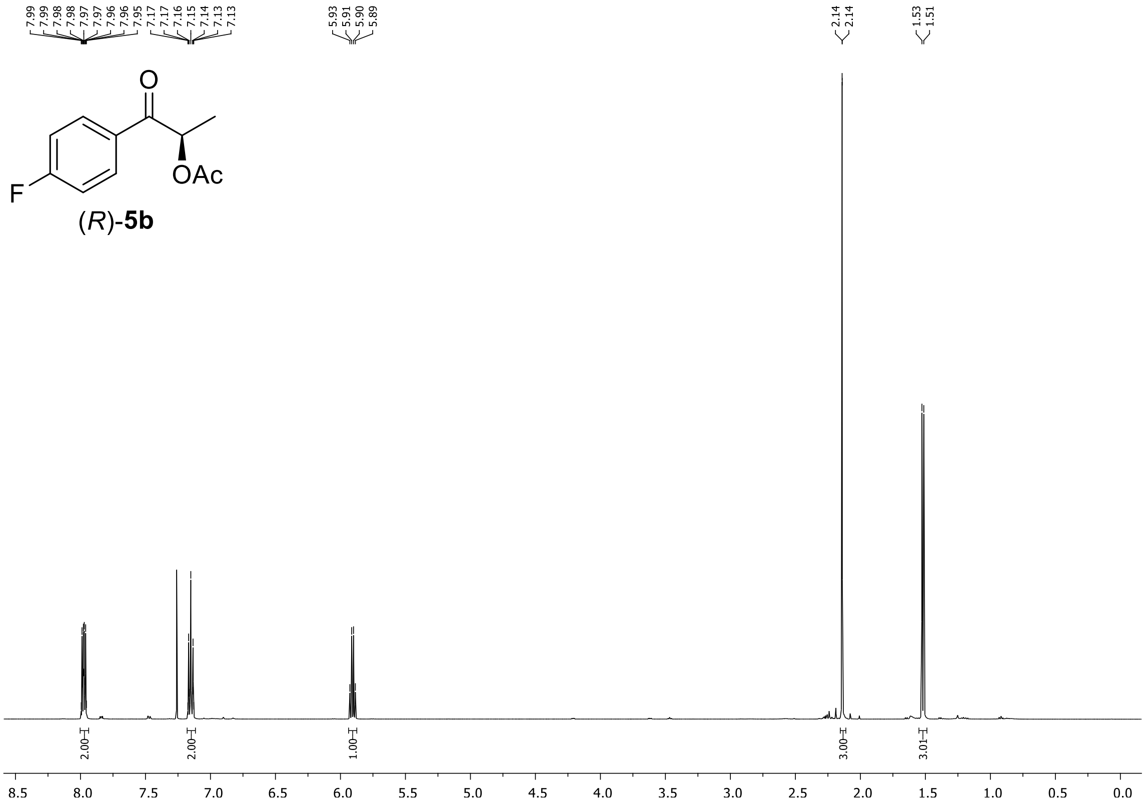

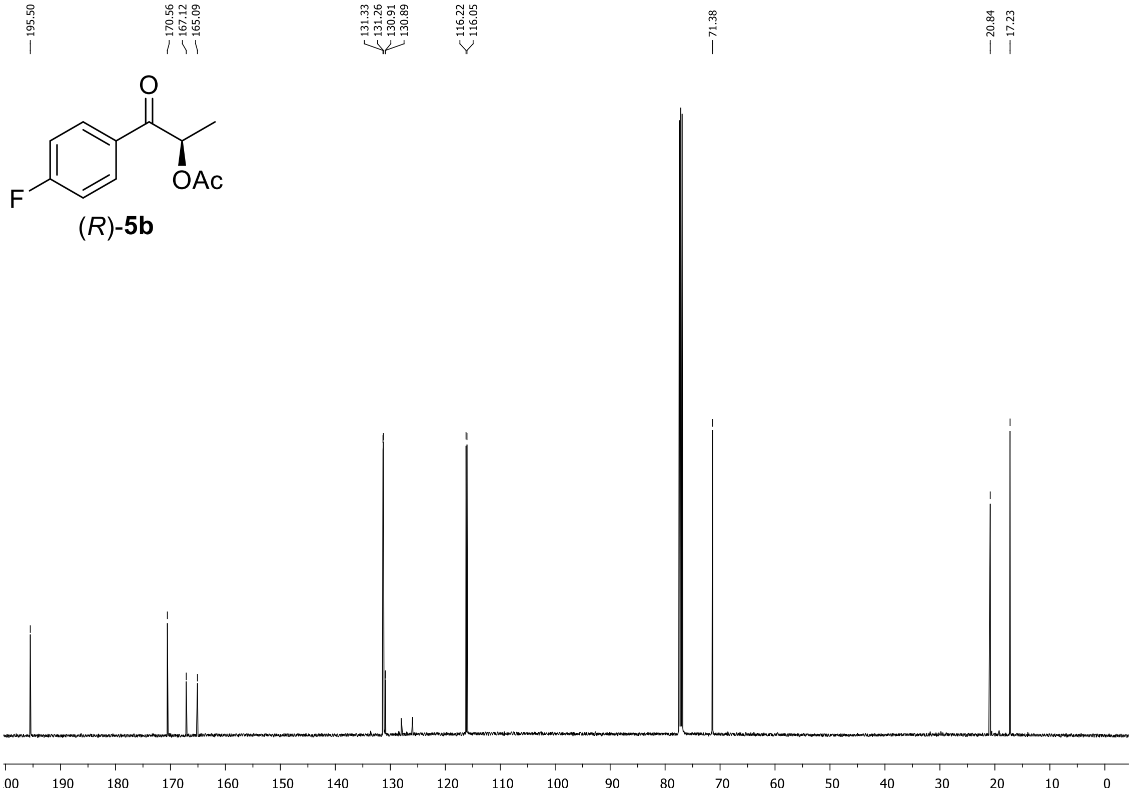

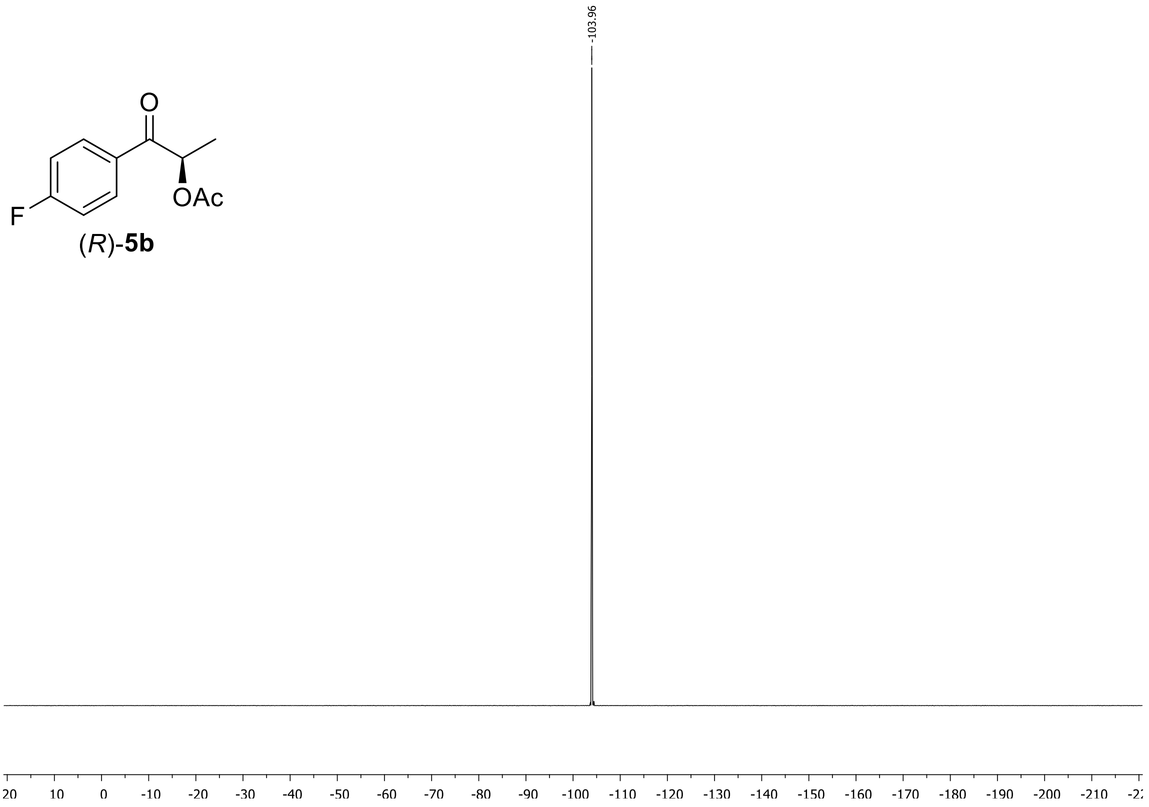

(*R*)-1-(3-Fluorophenyl)-1-oxopropan-2-yl acetate ((*R*)-5c)

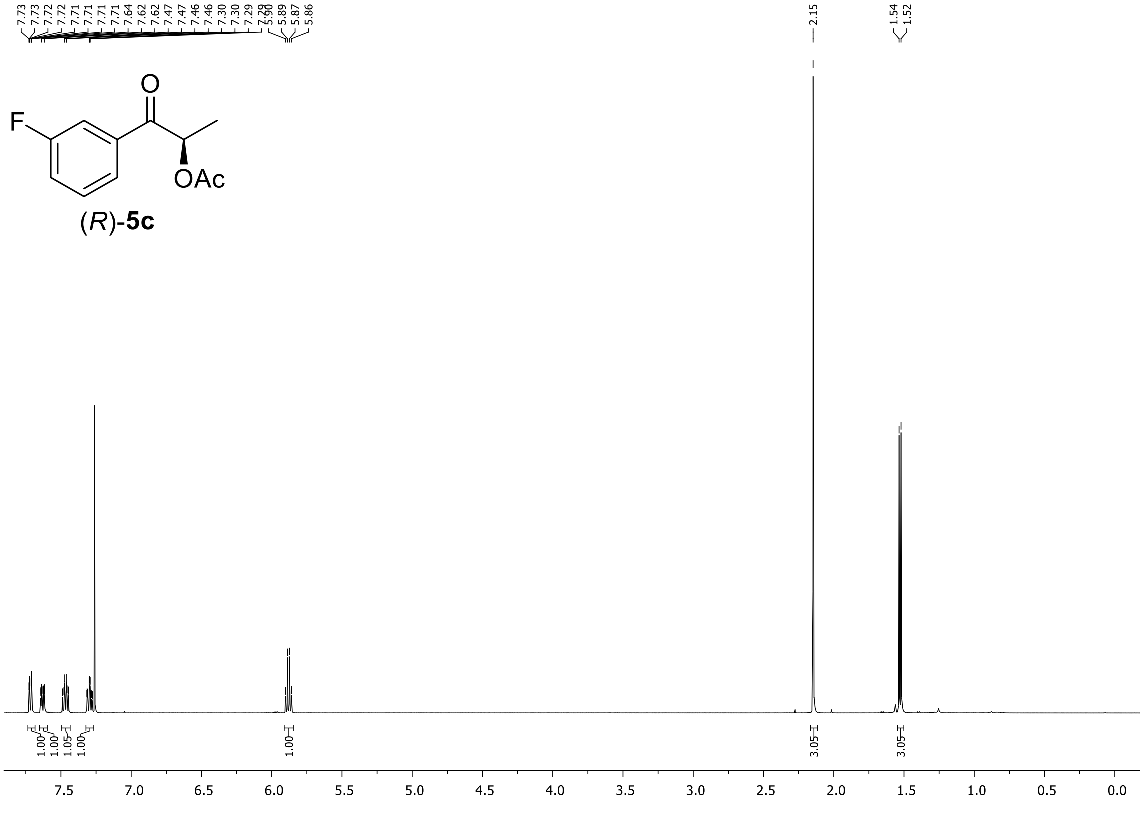

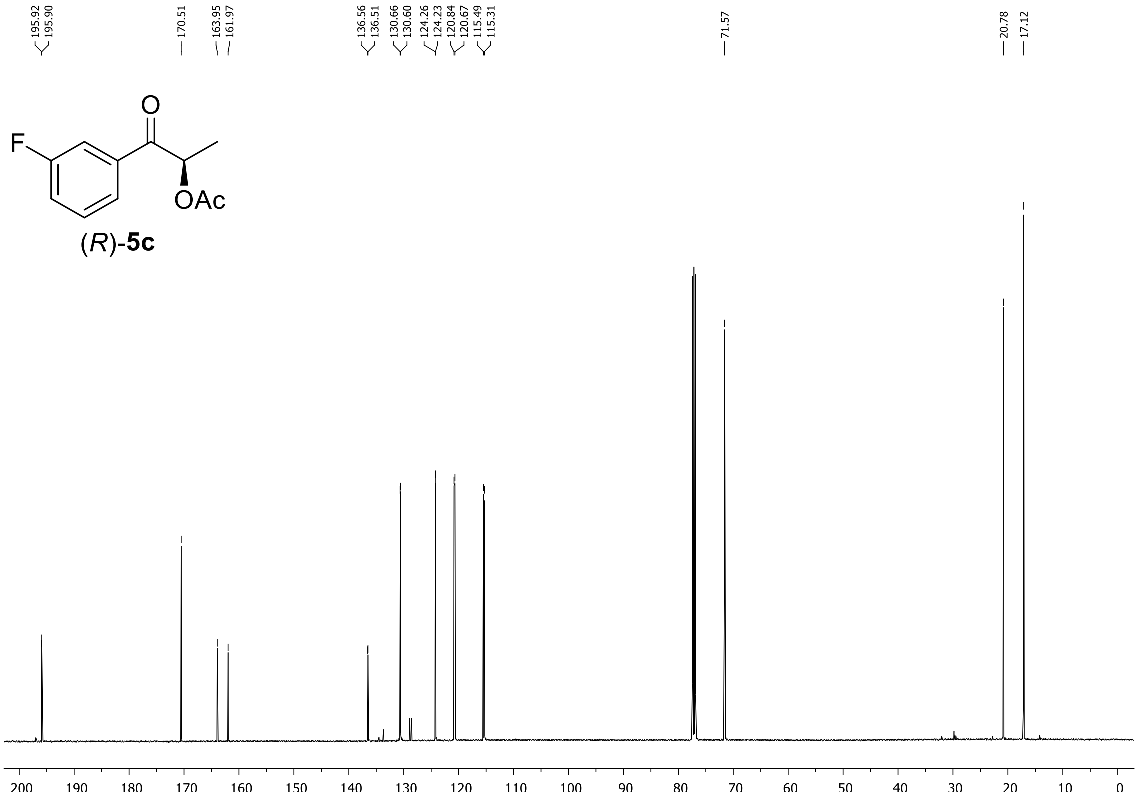

195.92
195.90
170.51
163.95
161.97
136.56
136.51
130.66
130.60
124.26
124.23
120.84
120.67
115.49
115.31
71.57
20.78
17.12
F
O
OAc
(R)-5c
200 190 180 170 160 150 140 130 120 110 100 90 80 70 60 50 40 30 20 10 0

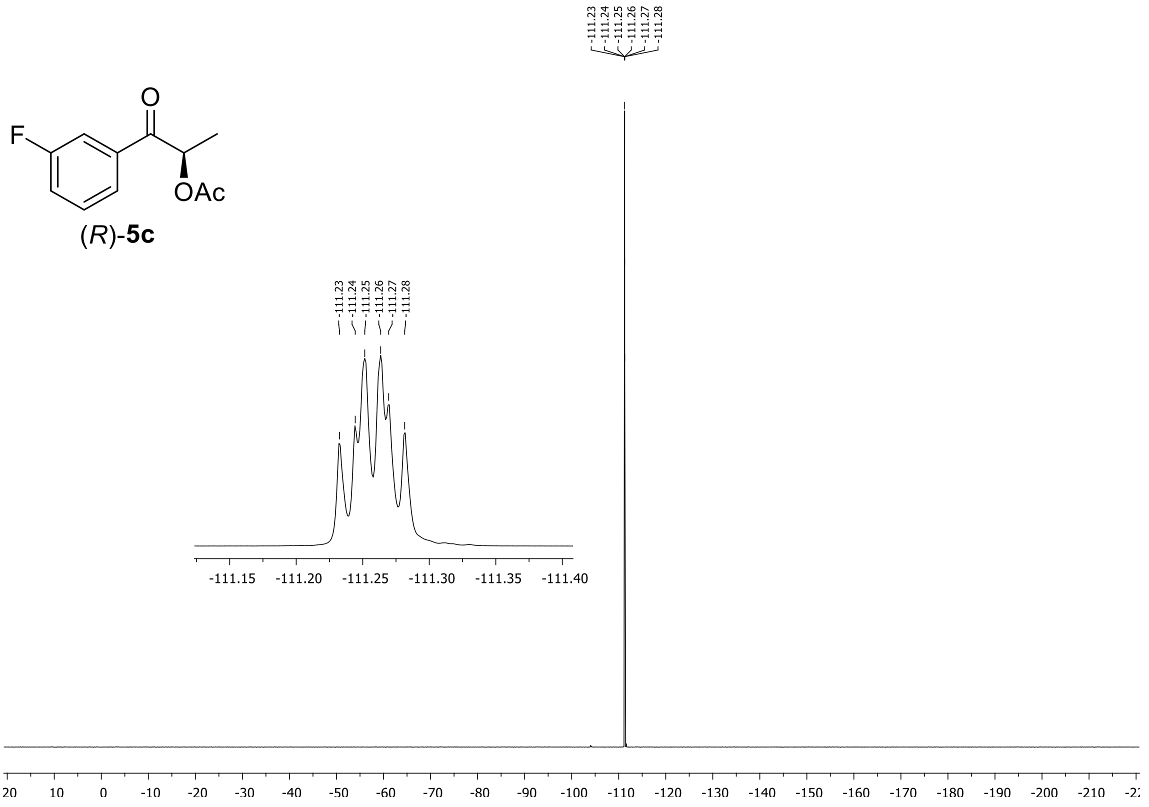

-111.23
-111.24
-111.25
-111.26
-111.27
-111.28
F
O
OAc
(R)-5c
-111.15 -111.20 -111.25 -111.30 -111.35 -111.40
20 10 0 -10 -20 -30 -40 -50 -60 -70 -80 -90 -100 -110 -120 -130 -140 -150 -160 -170 -180 -190 -200 -210

(*R*)-1-(4-Bromophenyl)-1-oxopropan-2-yl acetate ((*R*)-5d)

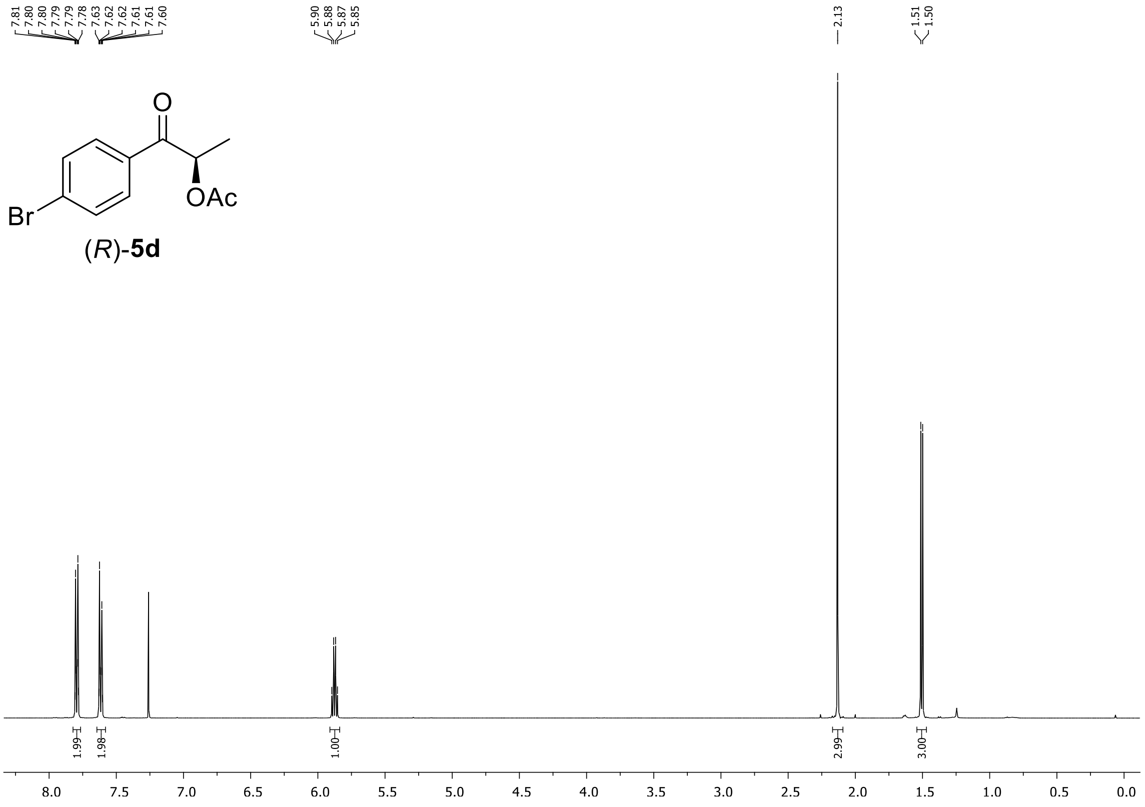

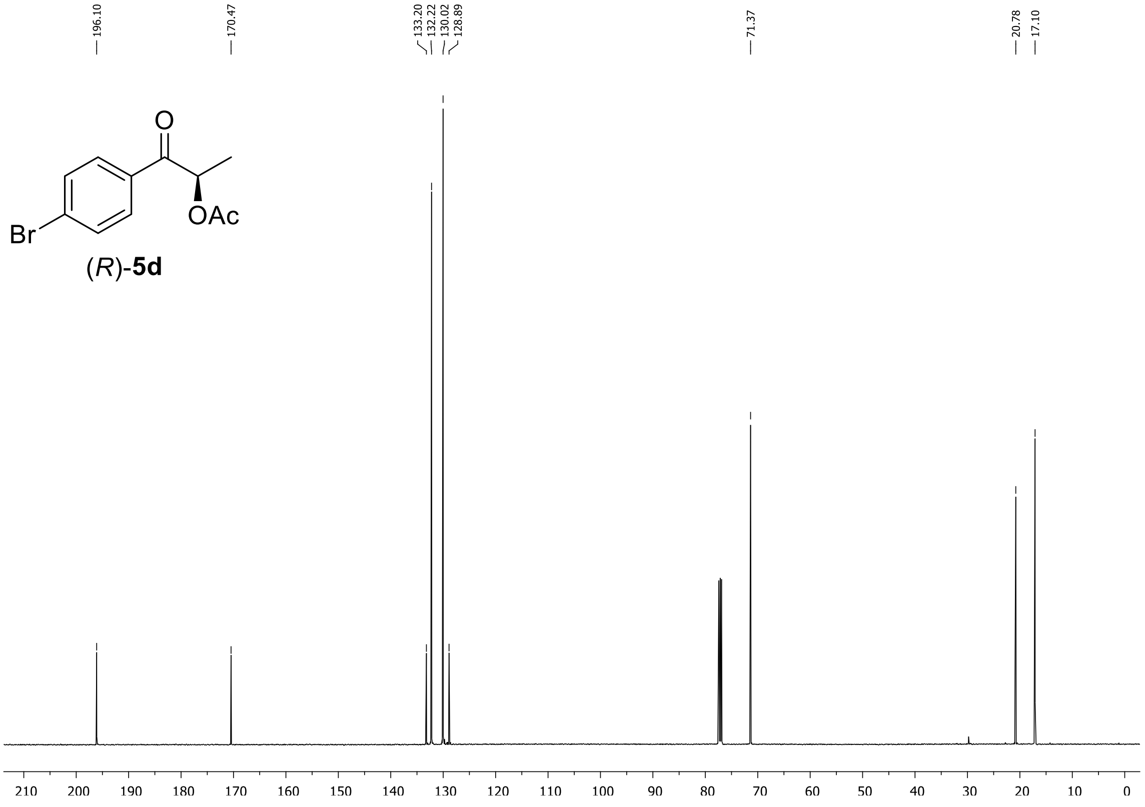

(*R*)-1-Oxo-1-(3-(trifluoromethyl)phenyl)propan-2-yl acetate ((*R*)-5e)

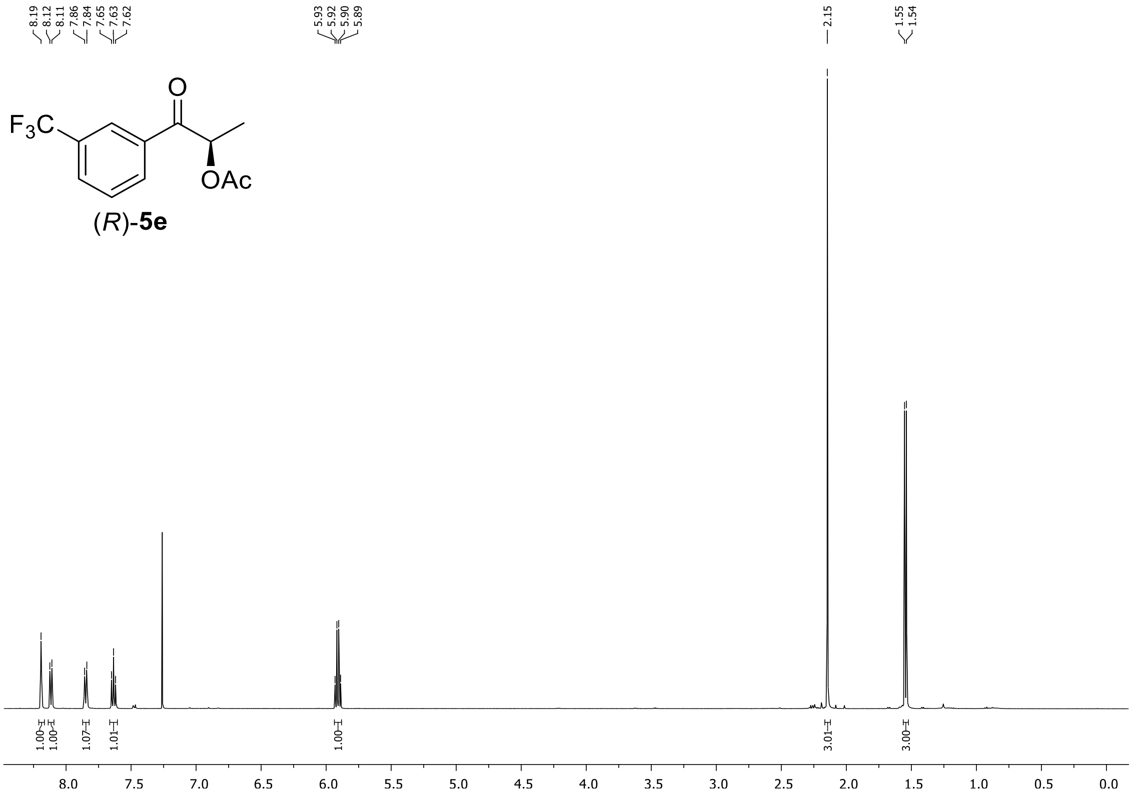

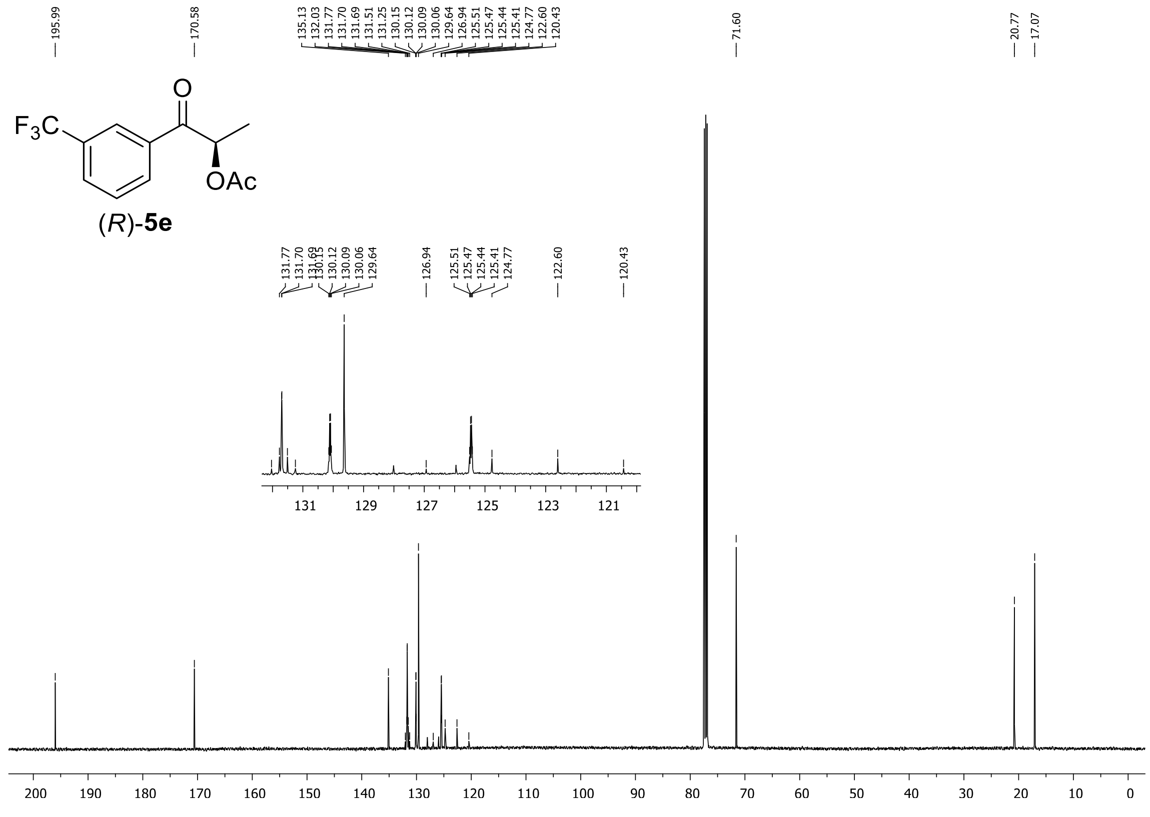

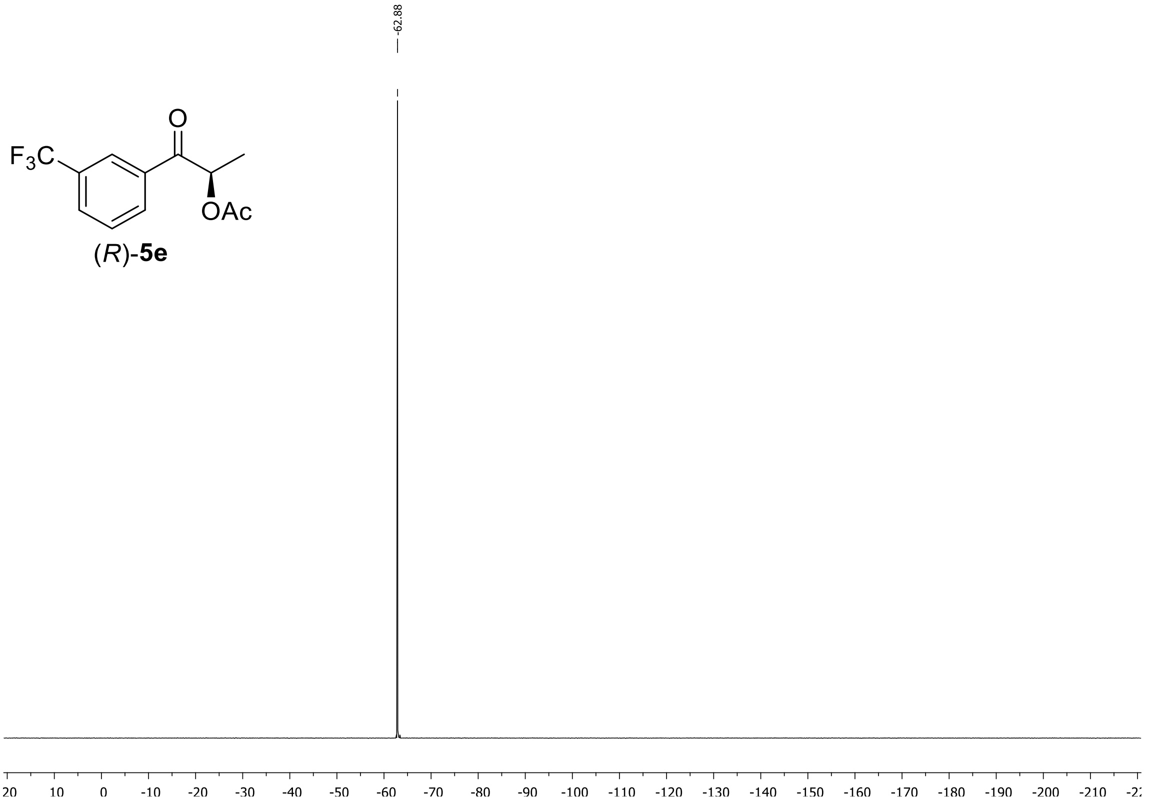

(*R*)-1-Oxo-1-(2-(trifluoromethyl)phenyl)propan-2-yl acetate ((*R*)-5f)

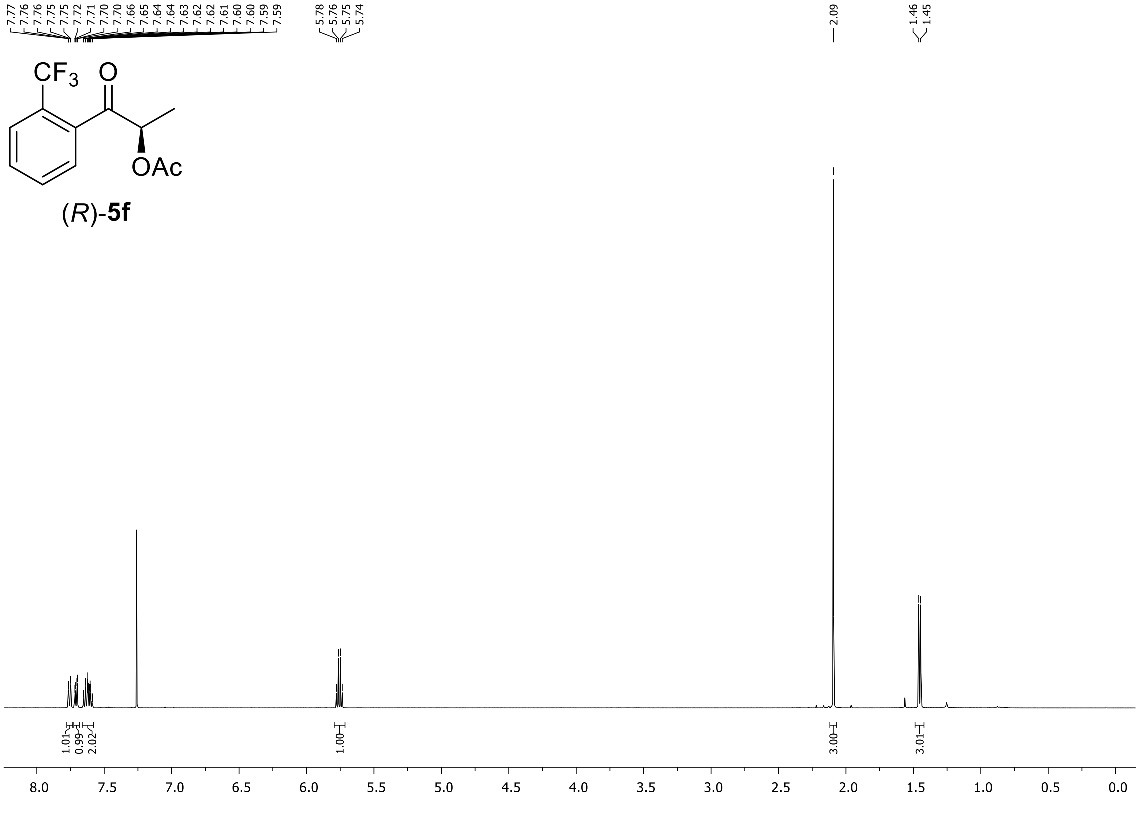

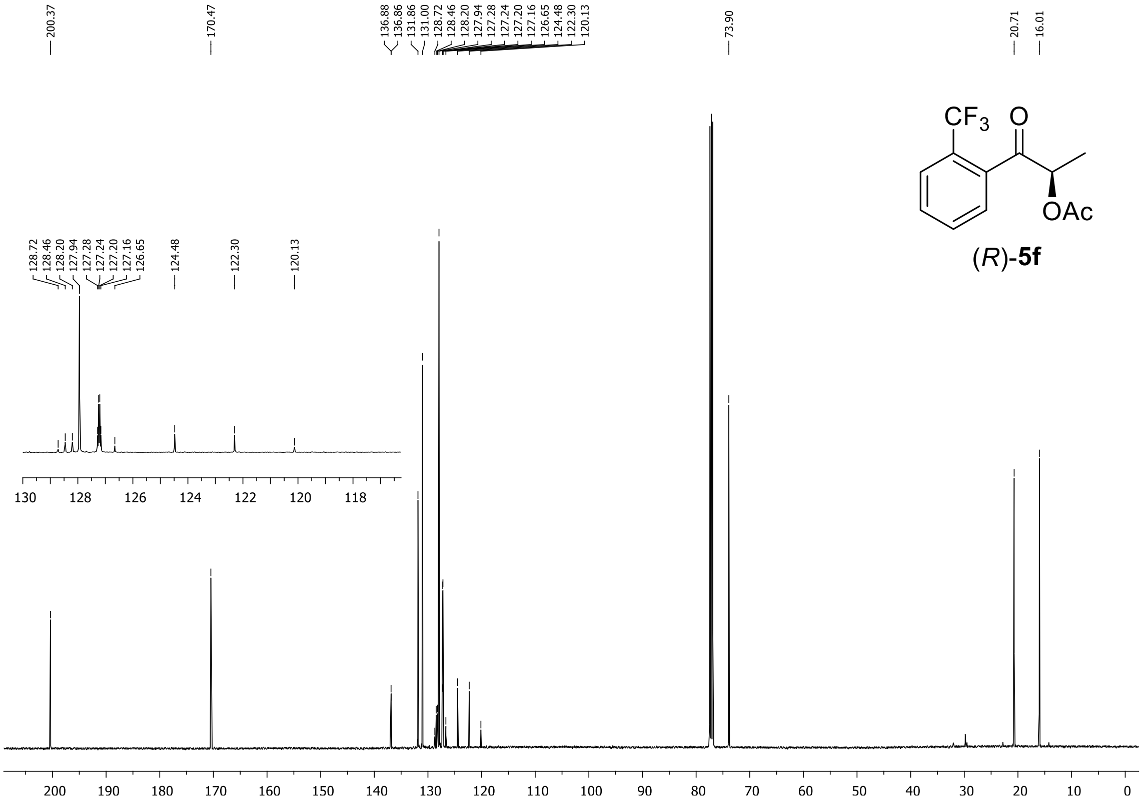
200.37
170.47
136.88
136.86
131.86
131.00
128.72
128.46
128.20
127.94
127.28
127.24
127.20
127.16
126.65
124.48
122.30
120.13
73.90
20.71
16.01
CF3 O
OAc
(R)-5f

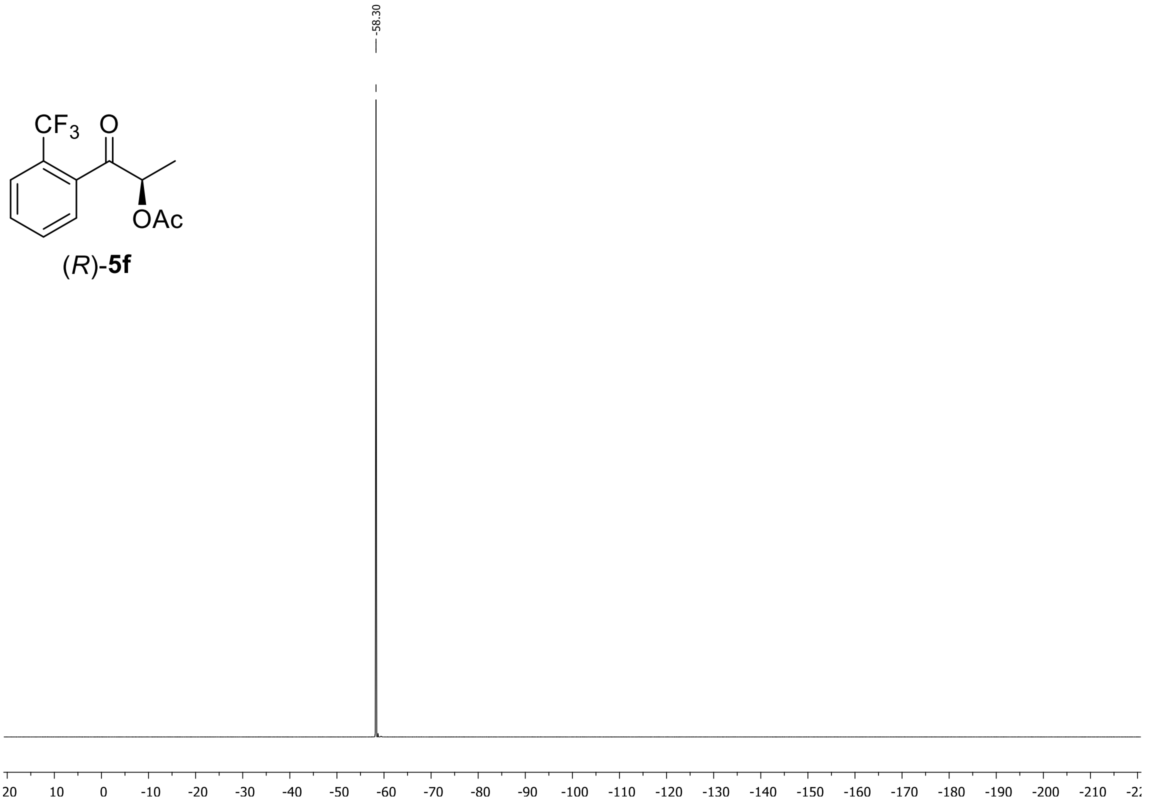
-58.30
CF3 O
OAc
(R)-5f

(*R*)-1-(3-Nitrophenyl)-1-oxopropan-2-yl acetate ((*R*)-5g)

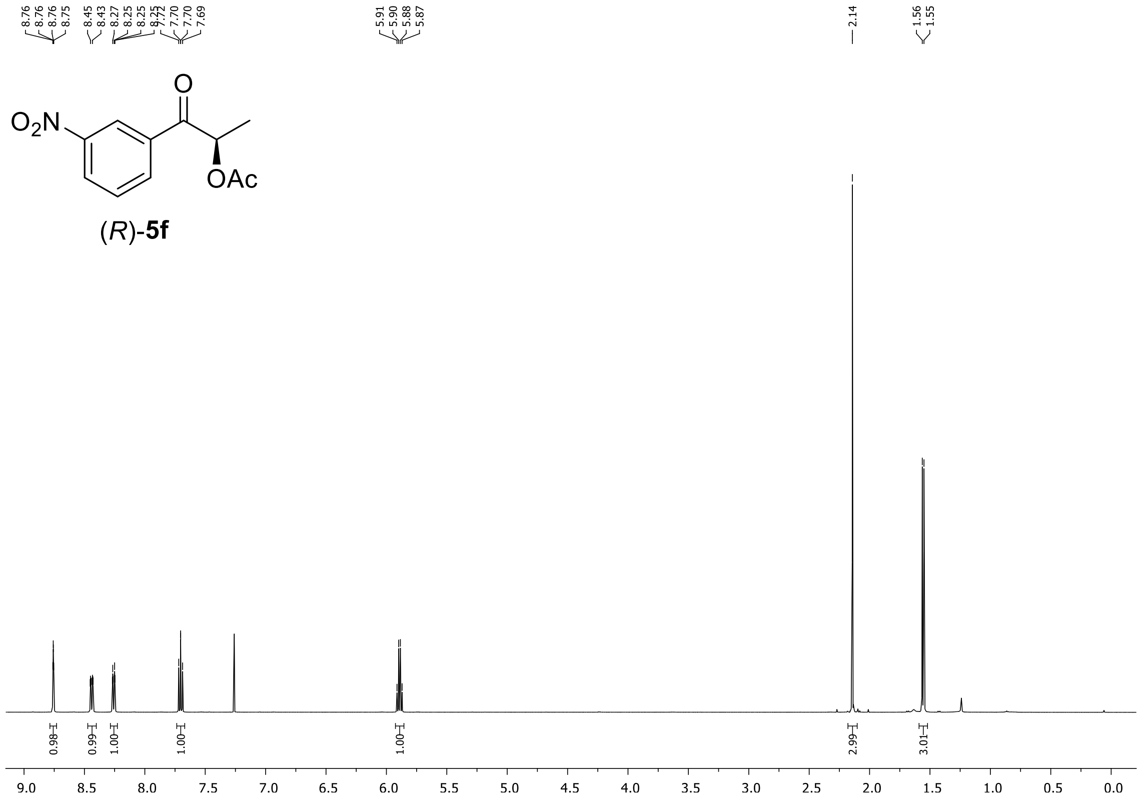

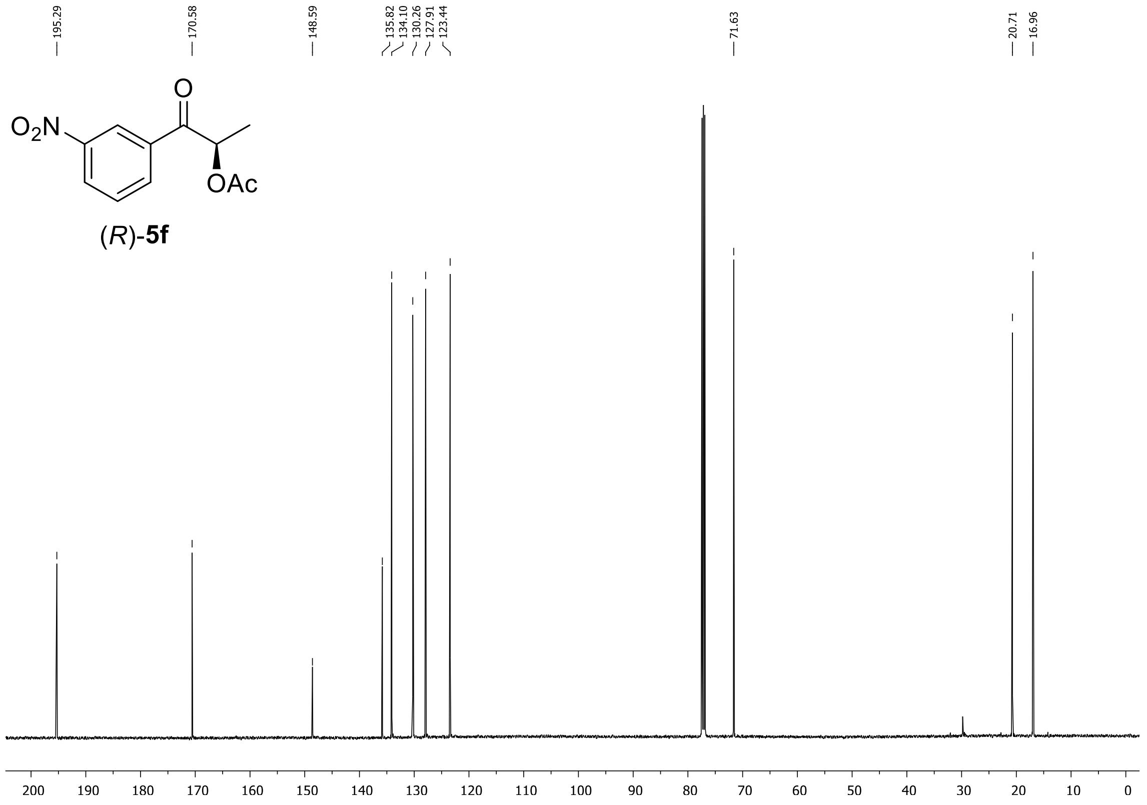

(*R*)-1-Oxo-1-(*p*-tolyl)propan-2-yl acetate ((*R*)-5h)

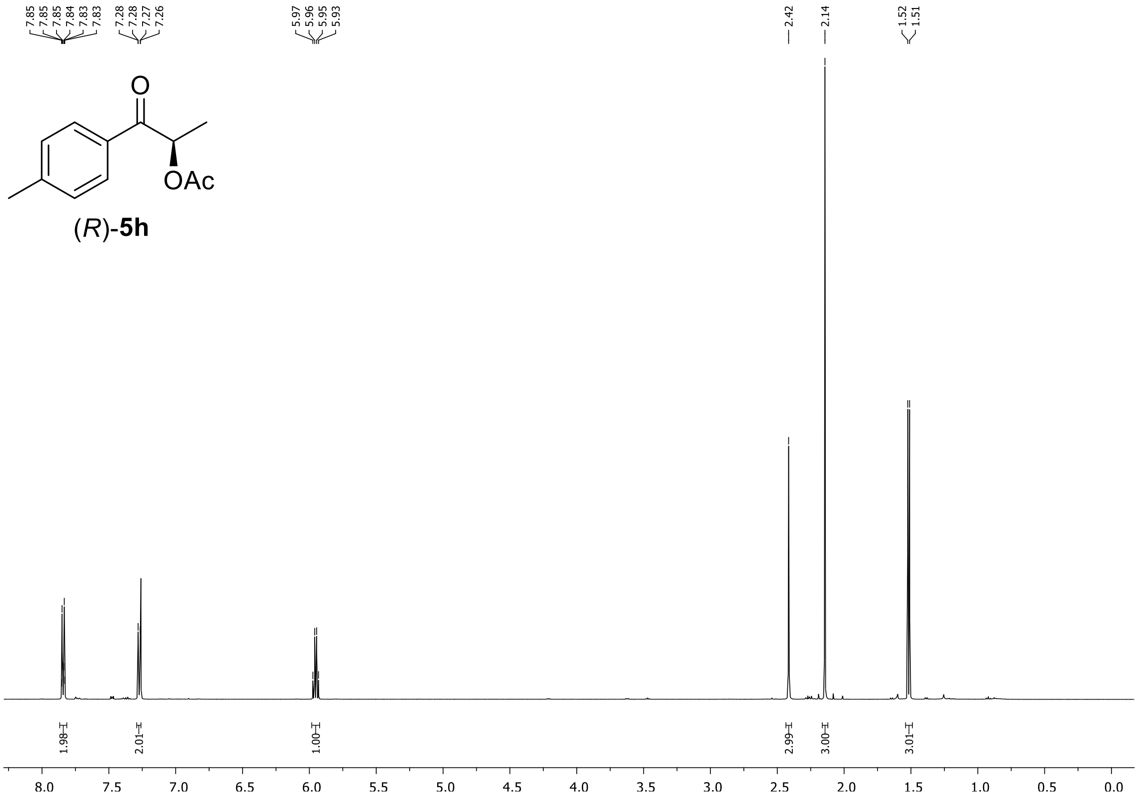

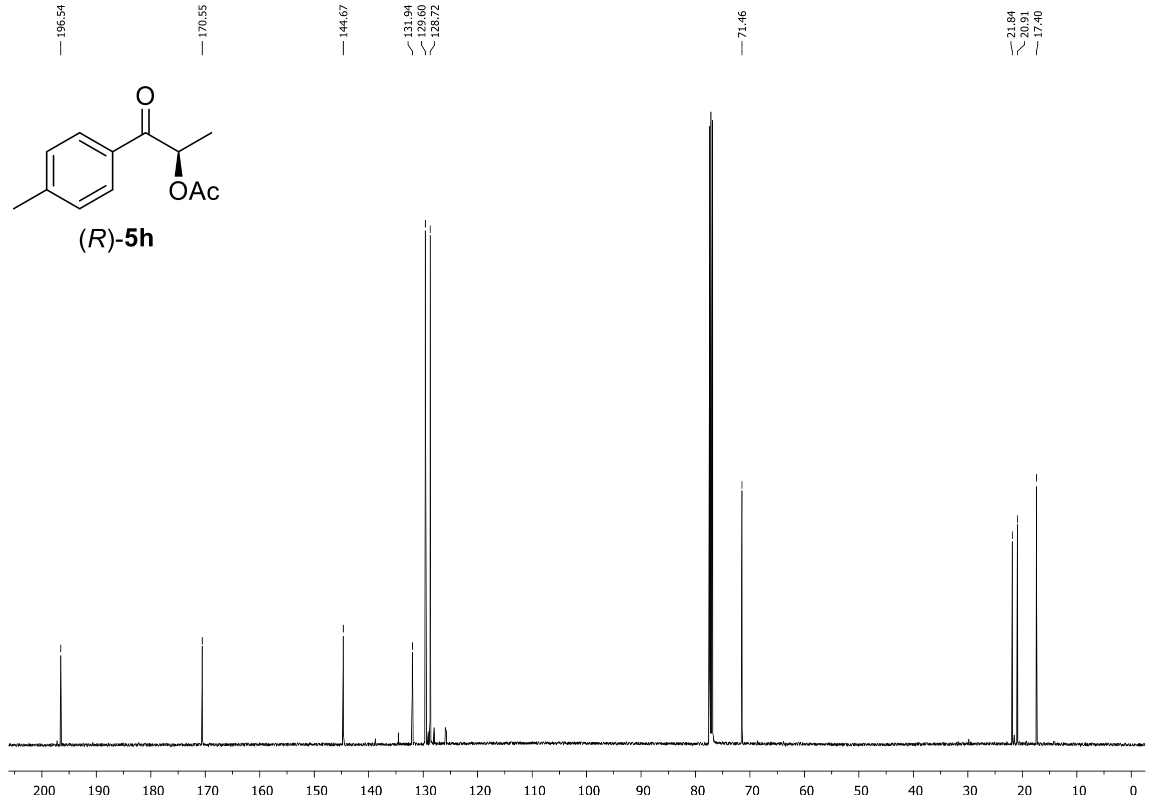

(*R*)-1-(4-(*tert*-Butyl)phenyl)-1-oxopropan-2-yl acetate ((*R*)-5i)

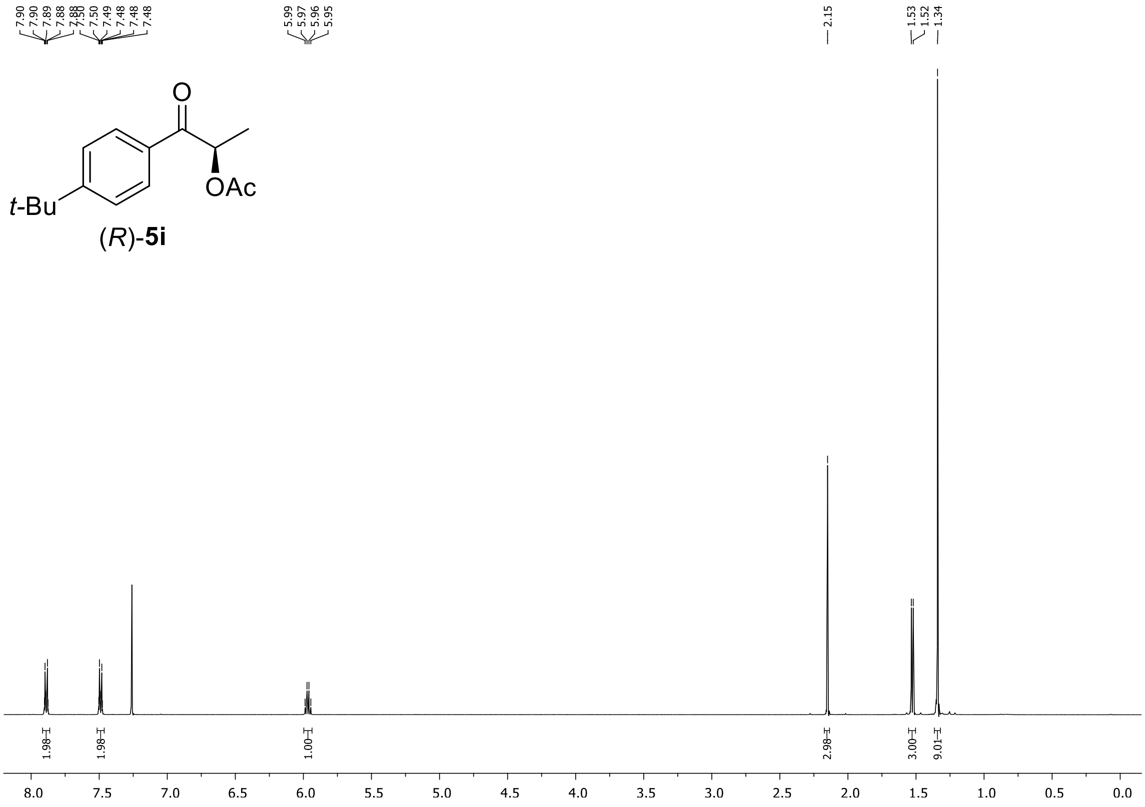

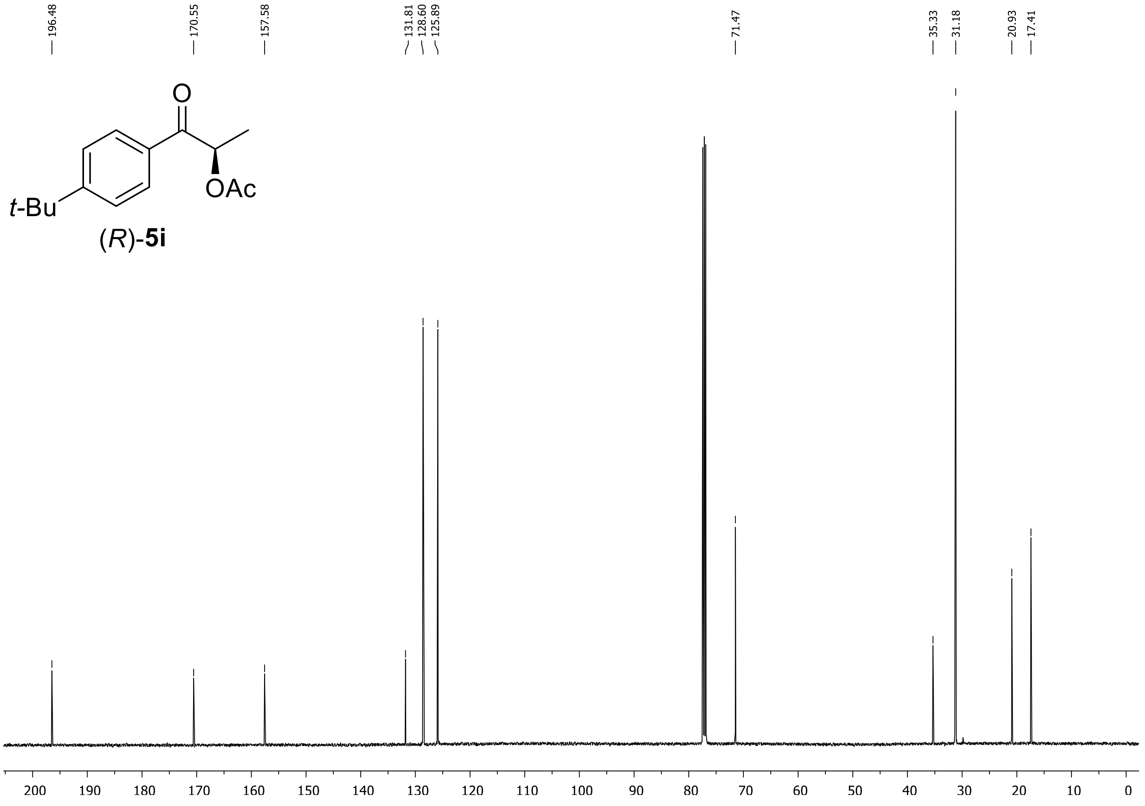

(*R*)-1-([1,1'-Biphenyl]-4-yl)-1-oxopropan-2-yl acetate ((*R*)-5j)

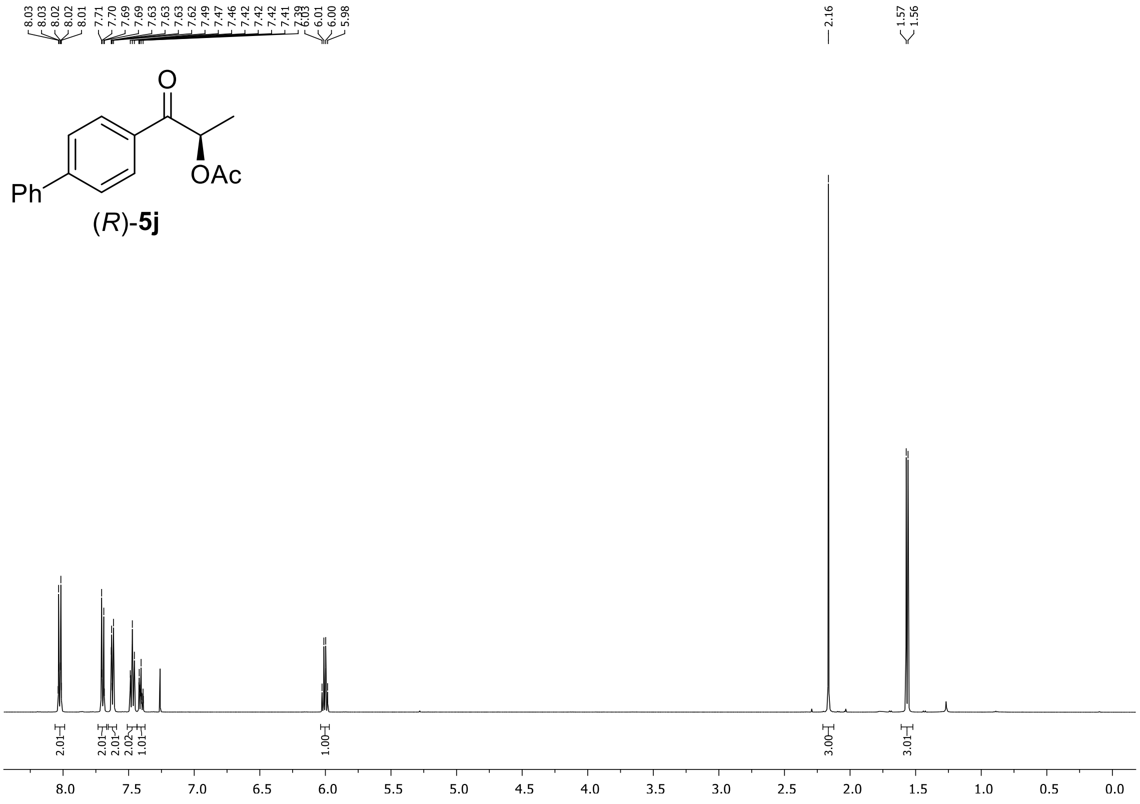

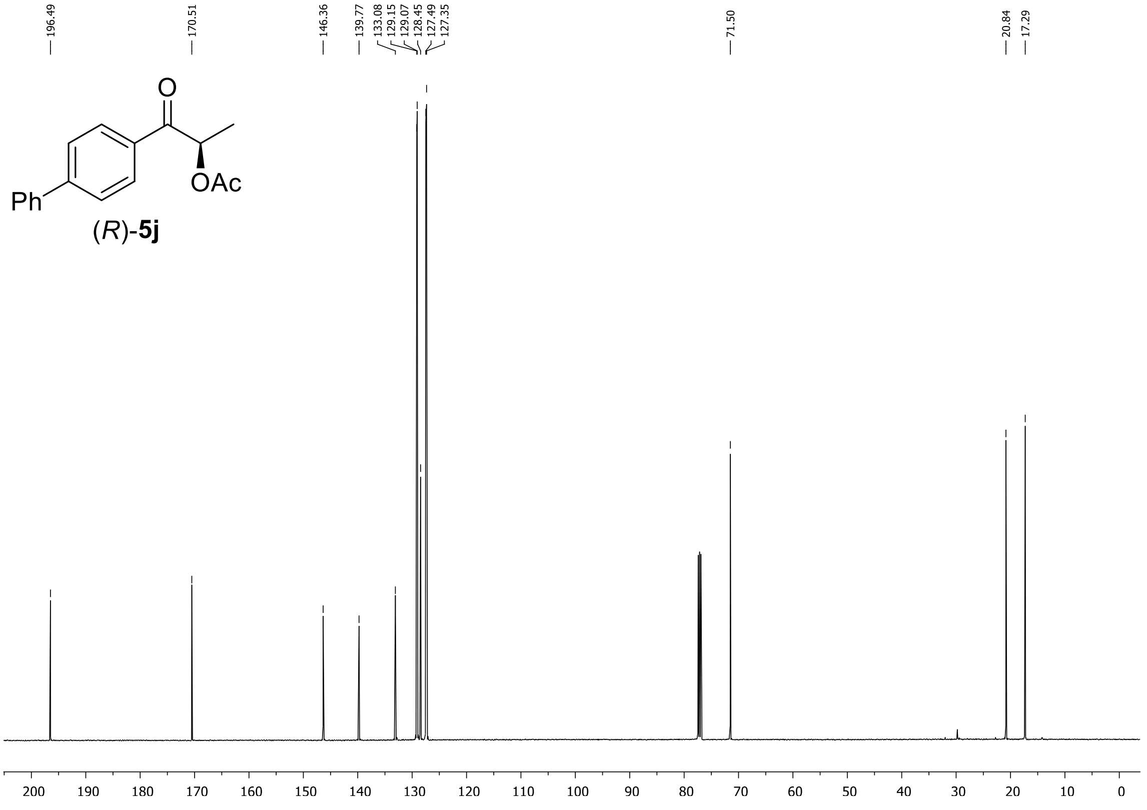

(*R*)-1-(4-Methoxyphenyl)-1-oxopropan-2-yl acetate ((*R*)-5k)

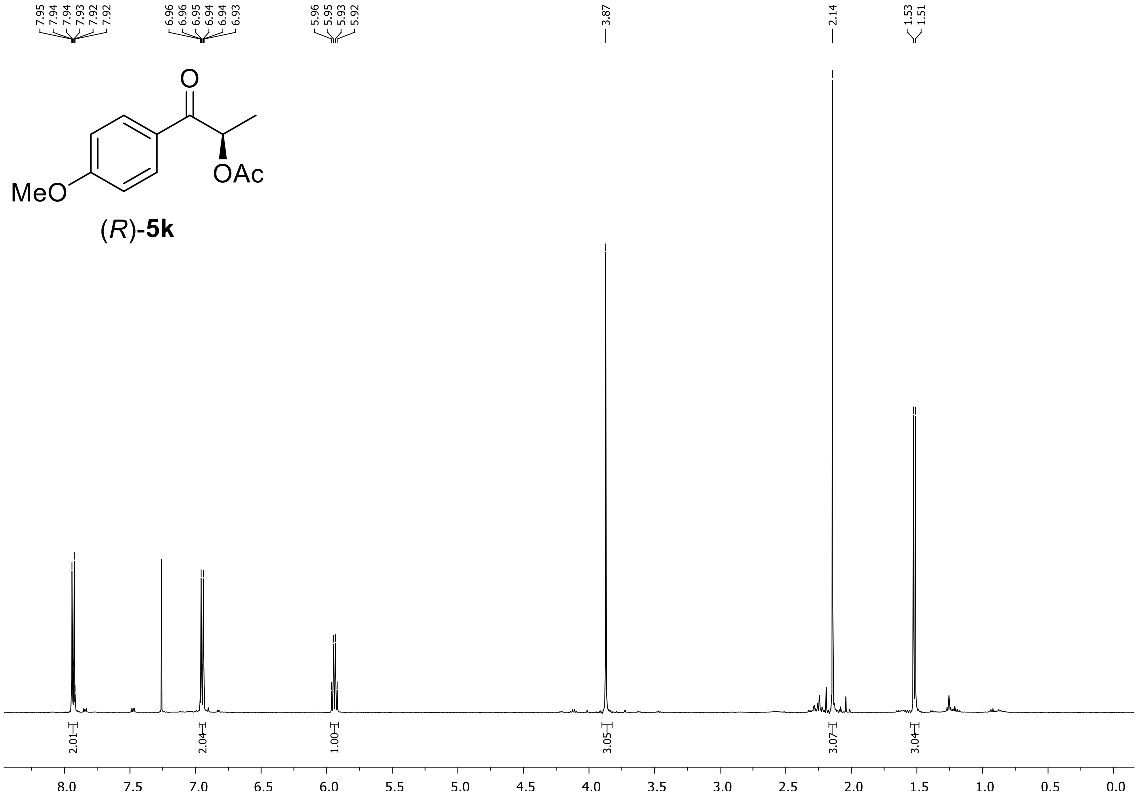

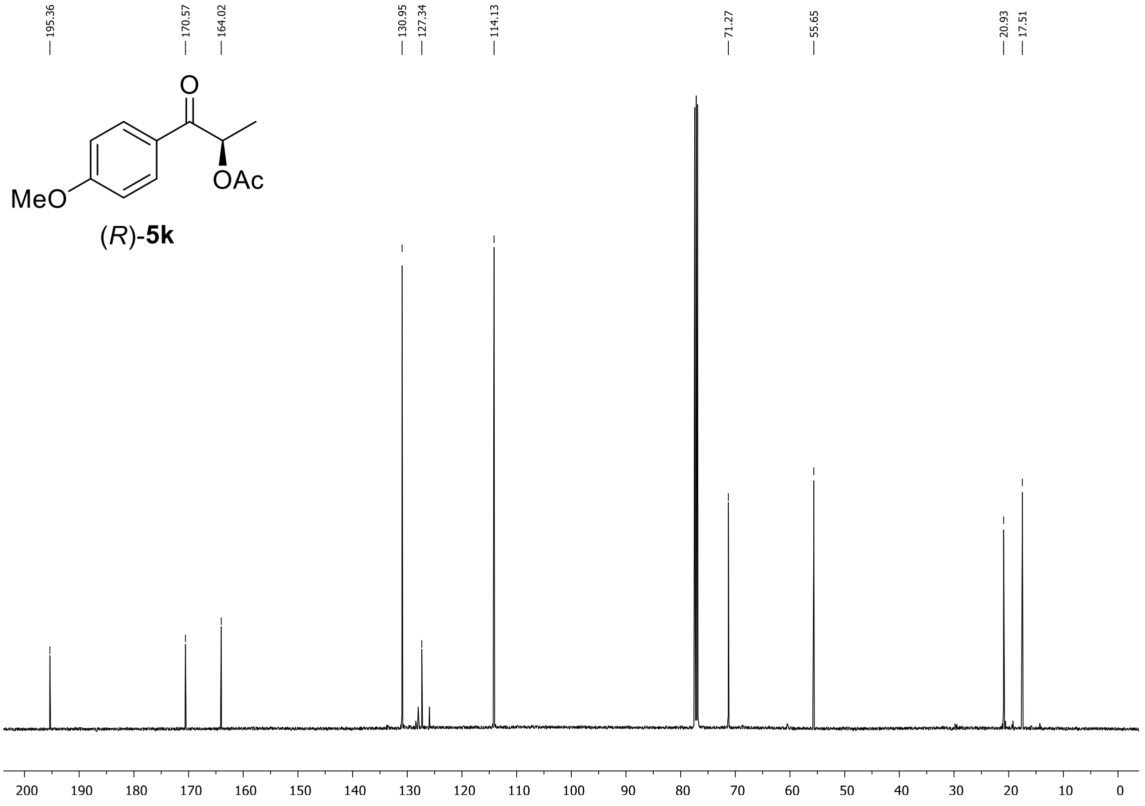

(*R*)-2-Oxo-1,2-diphenylethyl acetate ((*R*)-5l)

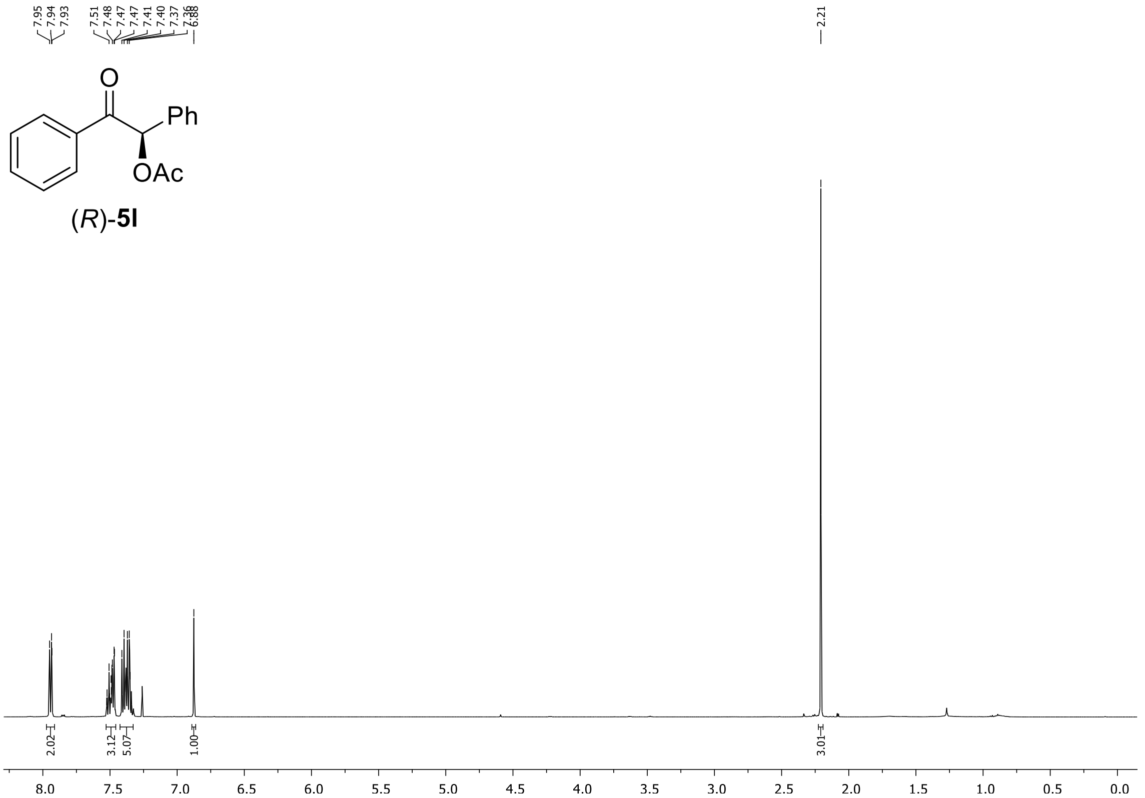

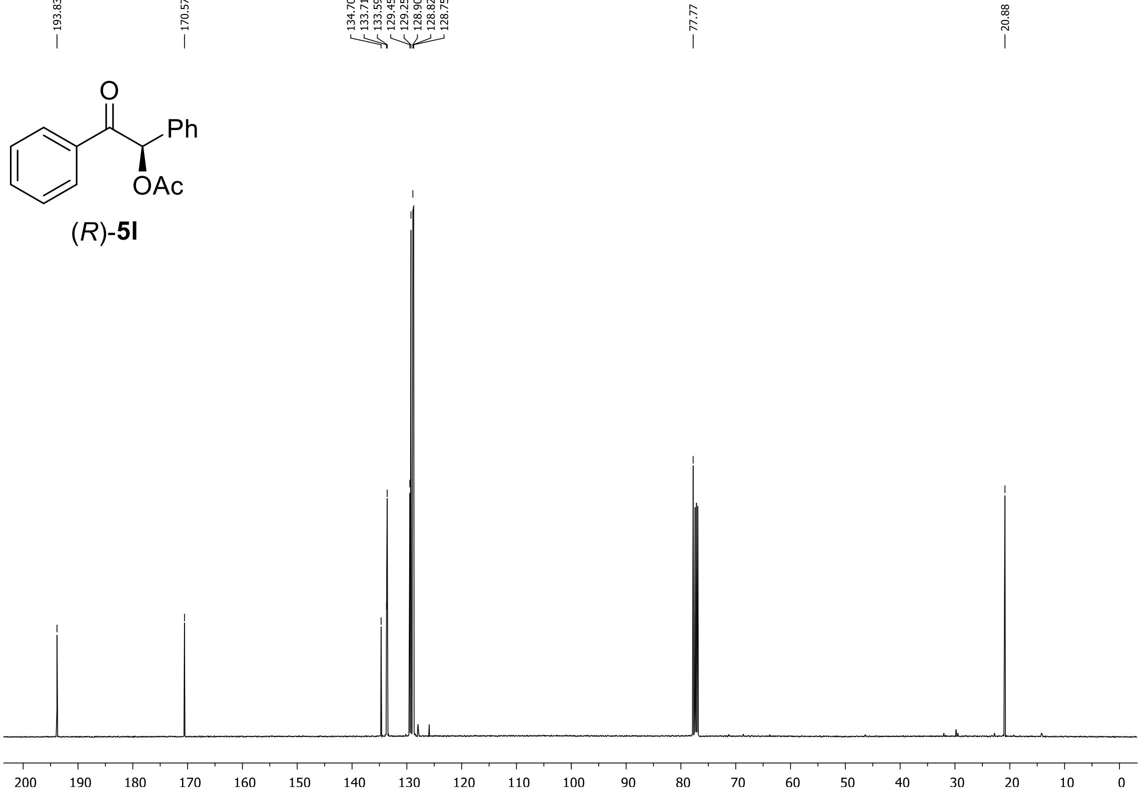

(*R*)-3-Methyl-1-oxo-1-phenylbutan-2-yl acetate ((*R*)-5m)

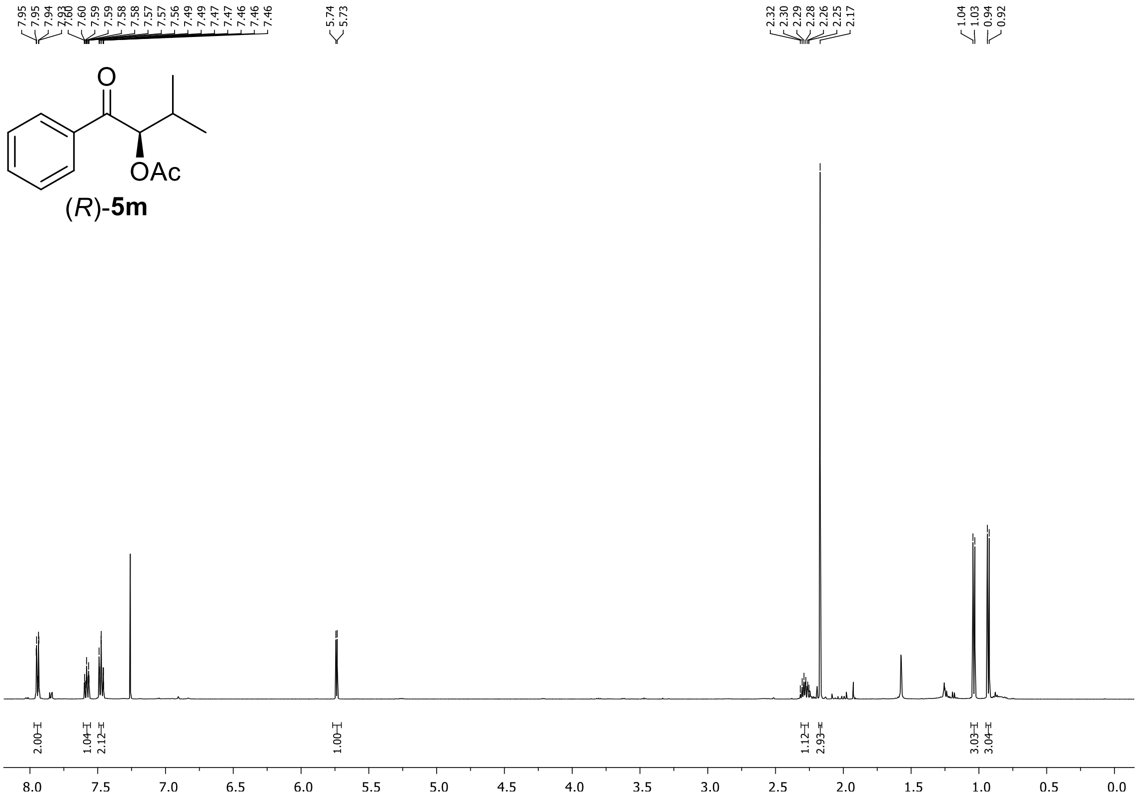

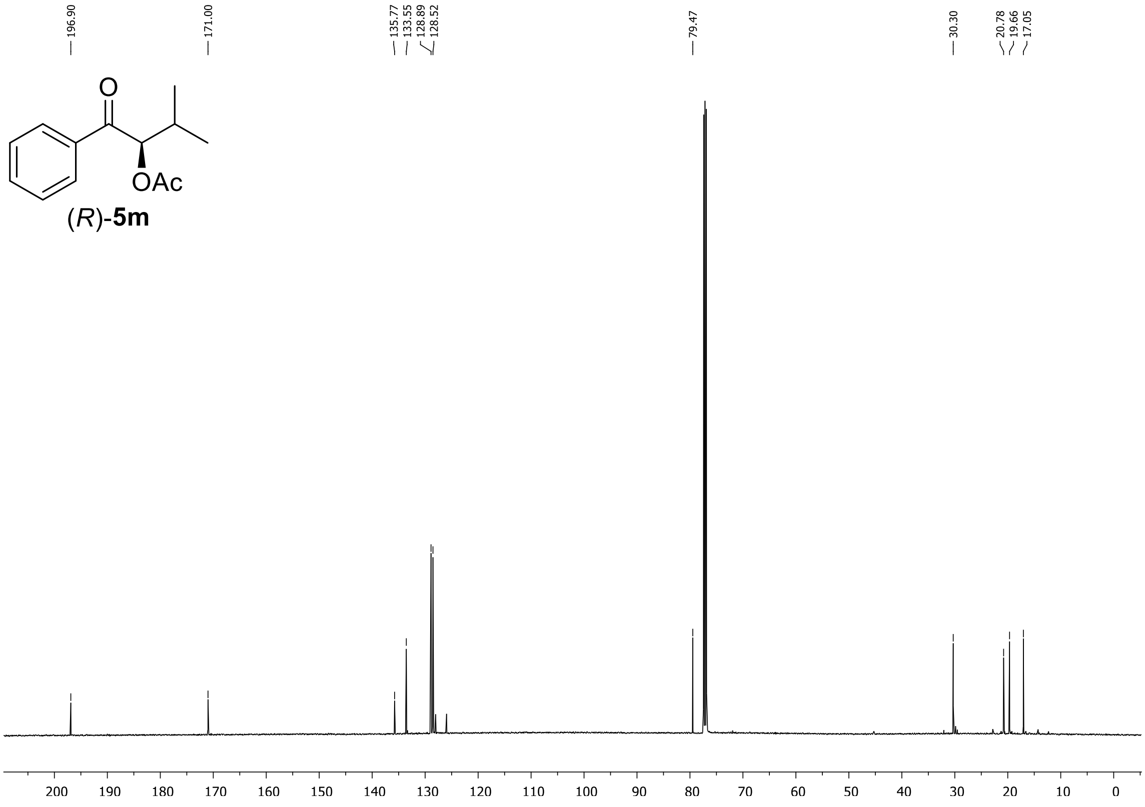

(*R*)-1-Oxo-1-phenylbutan-2-yl acetate ((*R*)-5n)

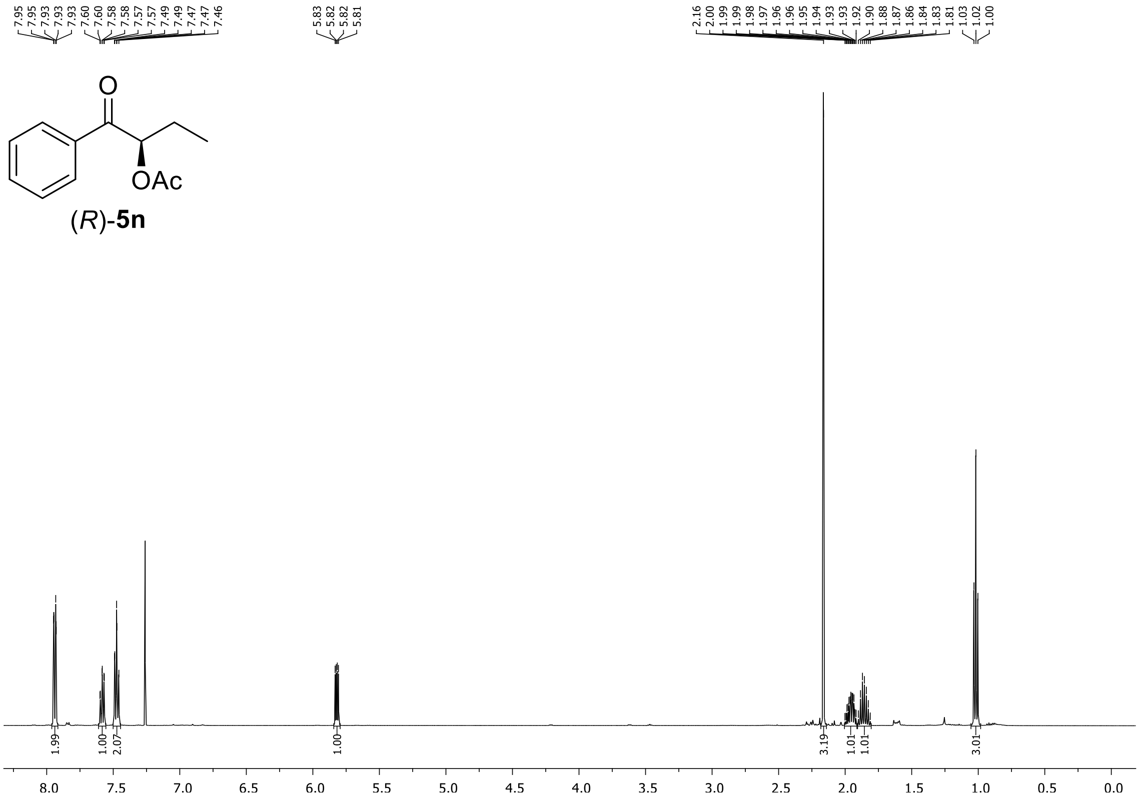

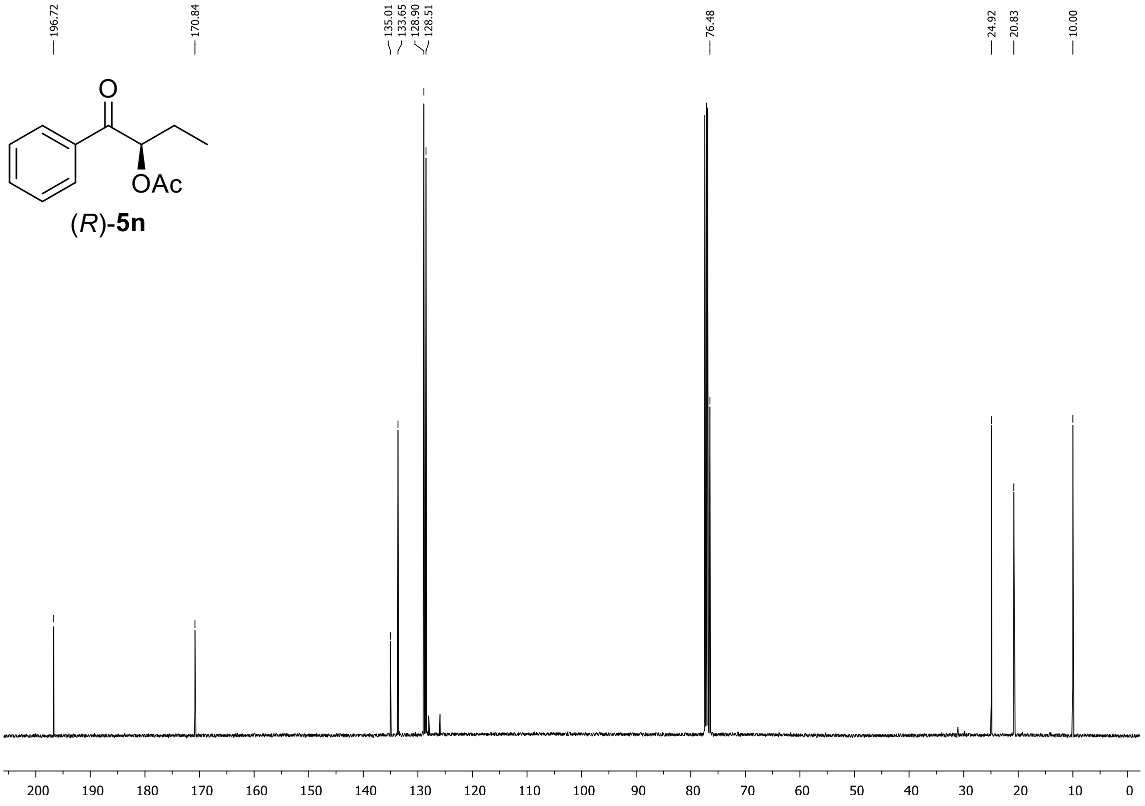

(*R*)-1-Oxo-1,2,3,4-tetrahydronaphthalen-2-yl acetate ((*R*)-5o)

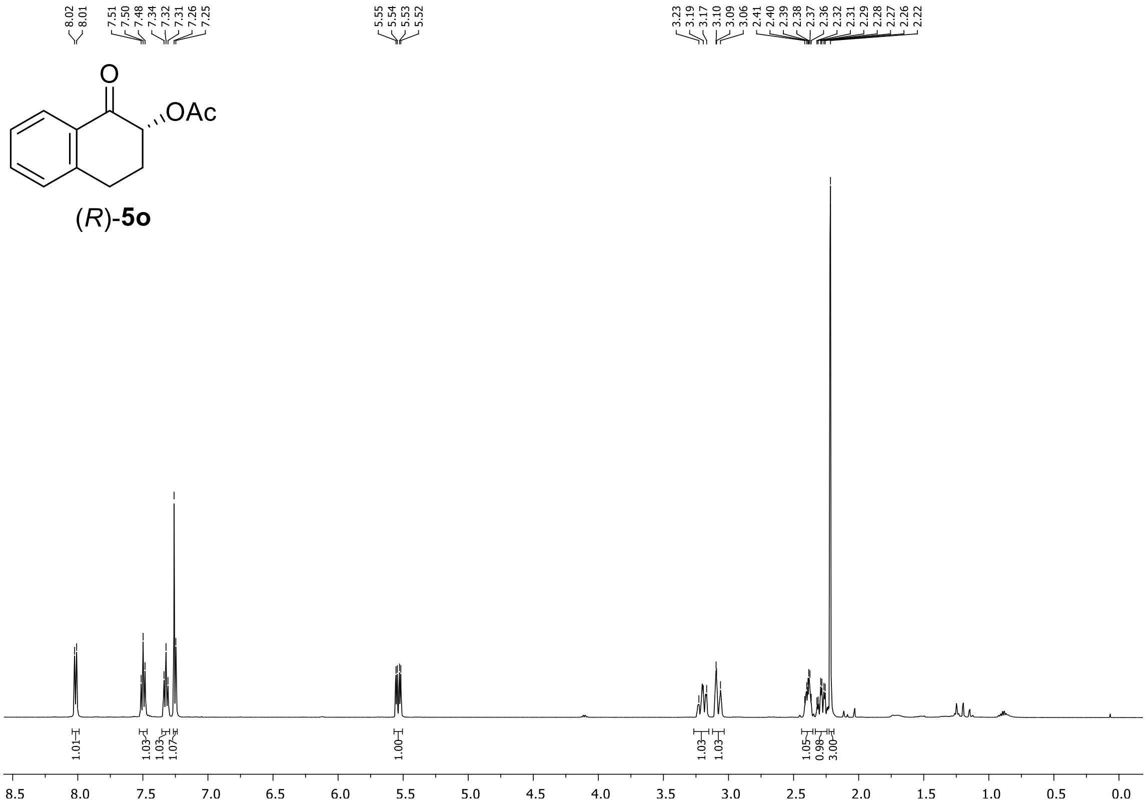

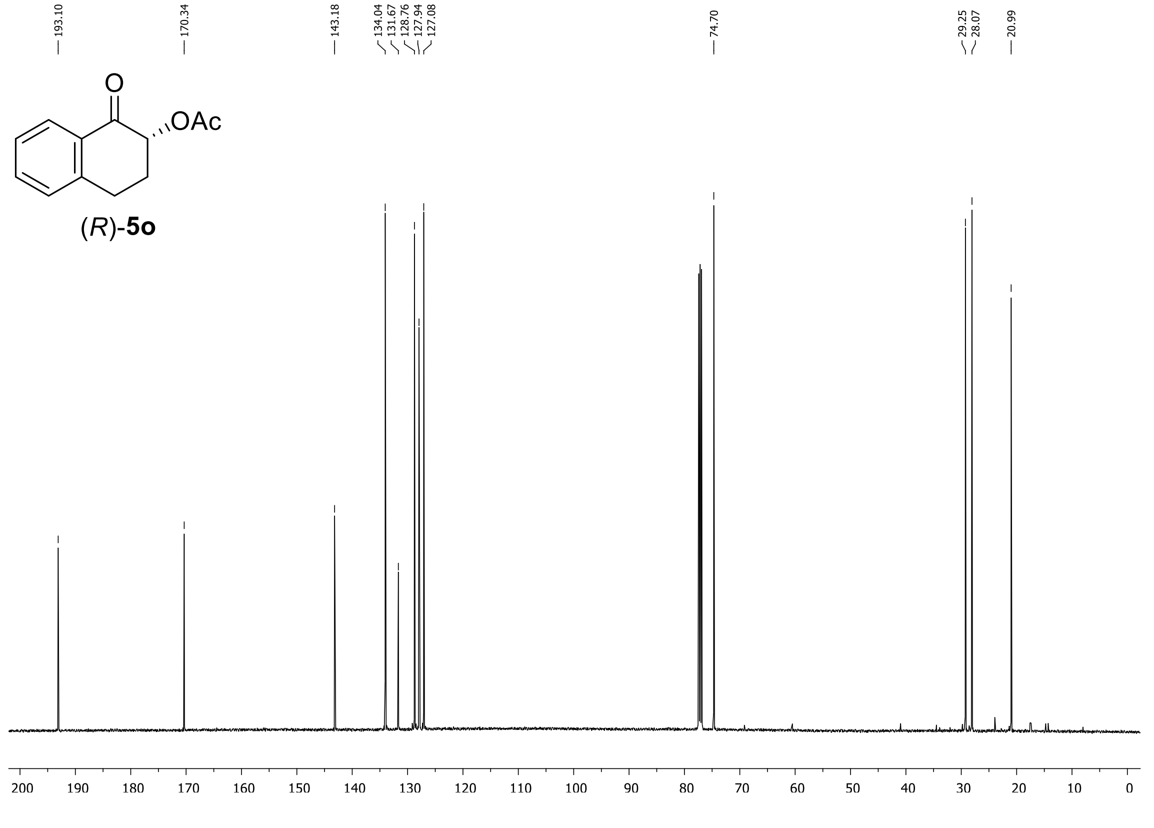

(*R*)-1-Oxo-1-(thiophen-2-yl)propan-2-yl acetate ((*R*)-5p)

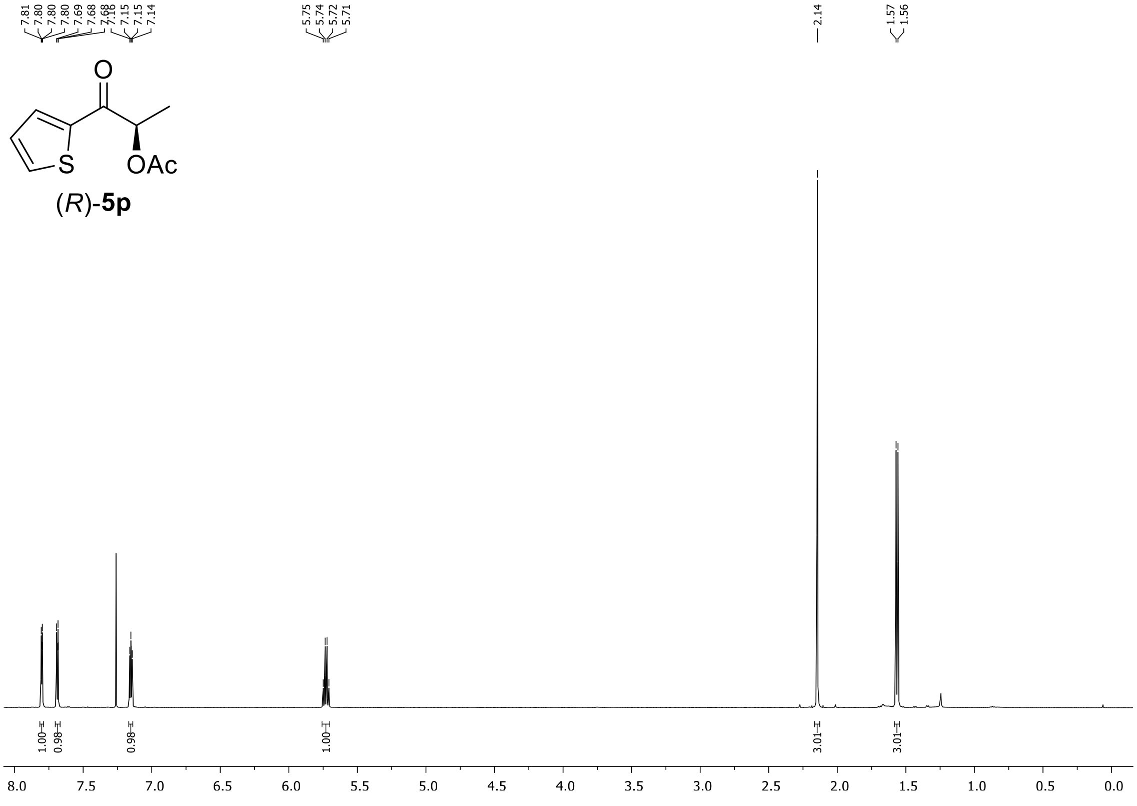

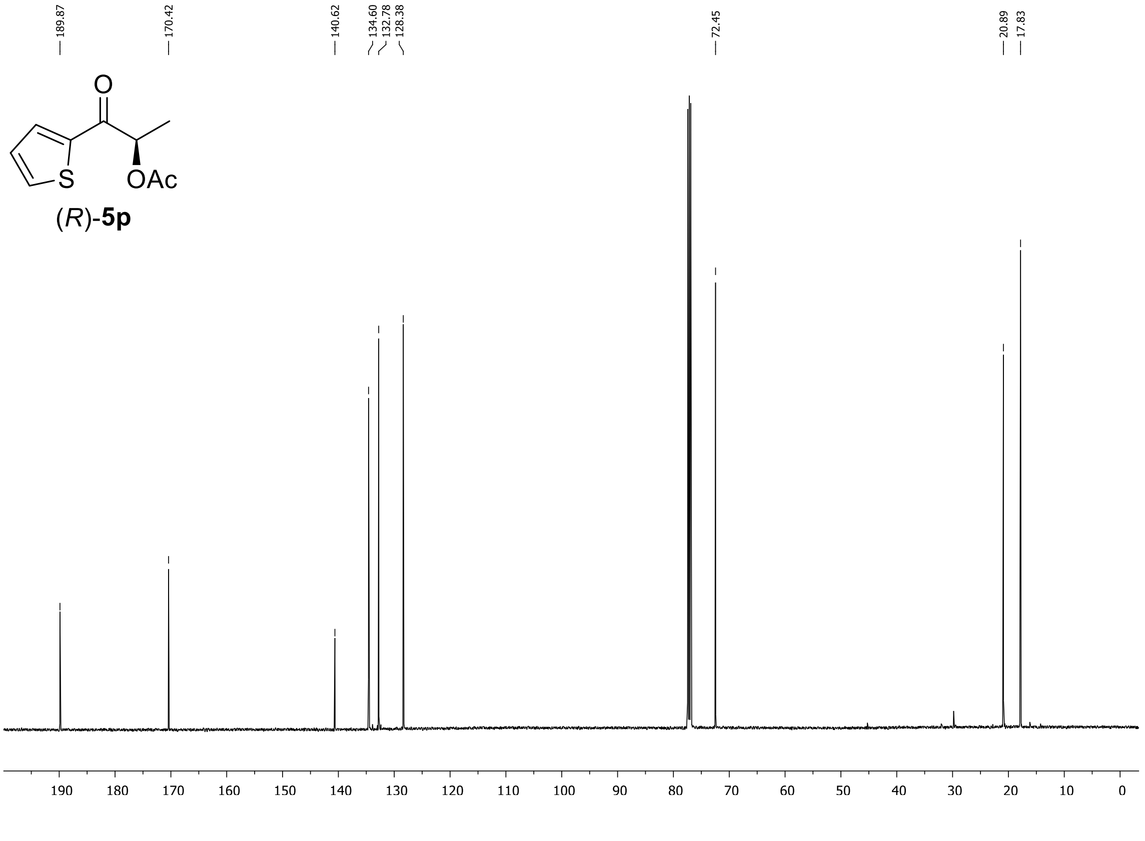

1-Cyano-2-oxo-2-phenylethyl acetate ((*rac*)-5q)

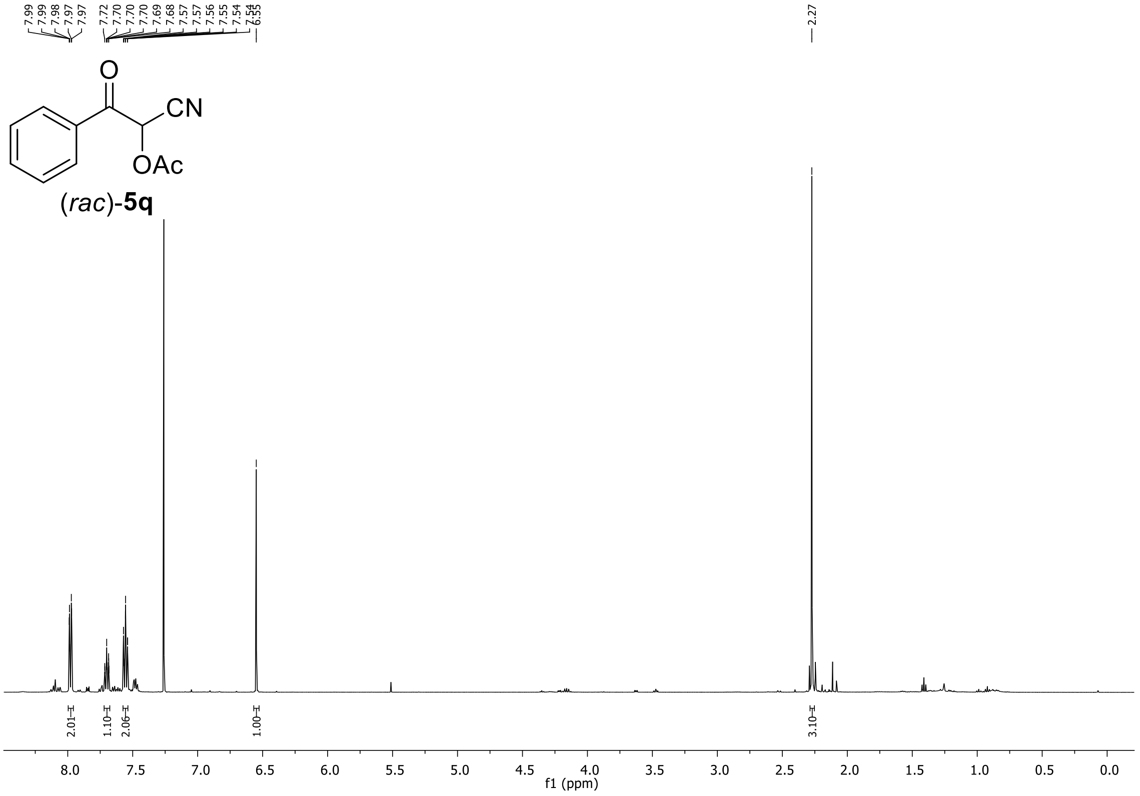

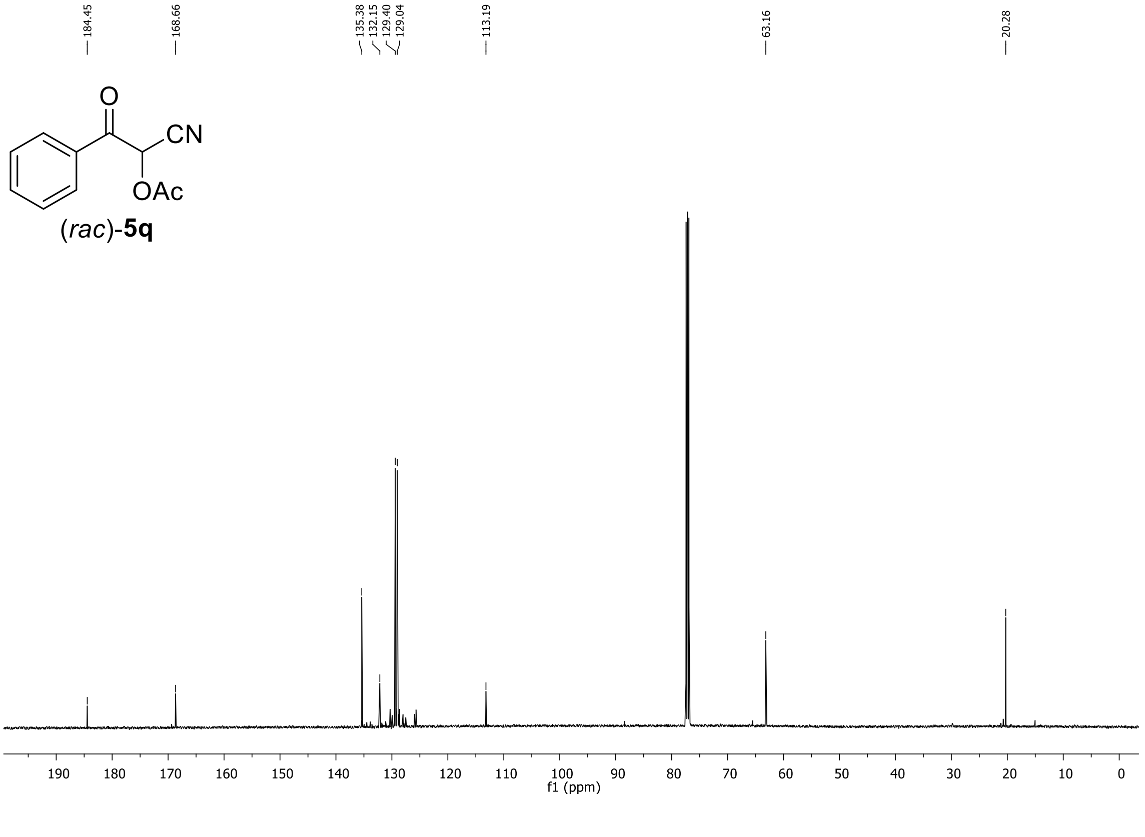

(2*S*)-2-[(4-Methylbenzenesulfonyl)oxy]-1-phenylpropan-1-one ((*S*)-S5)

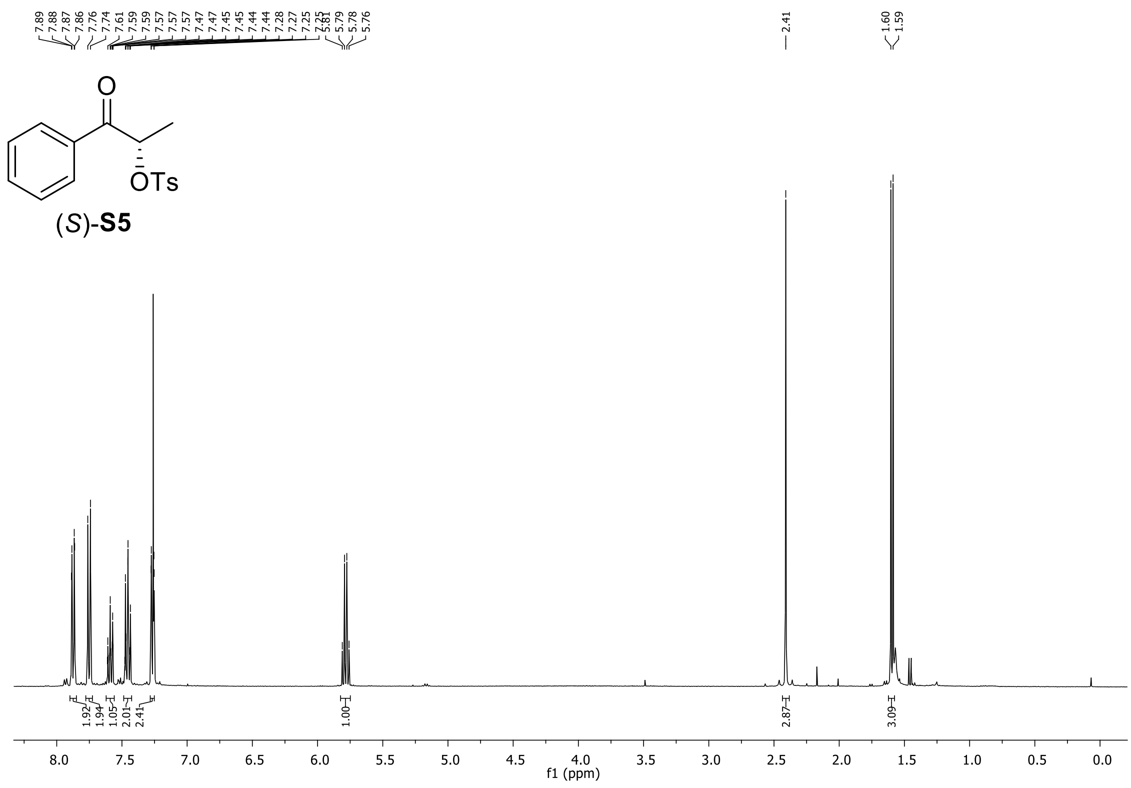

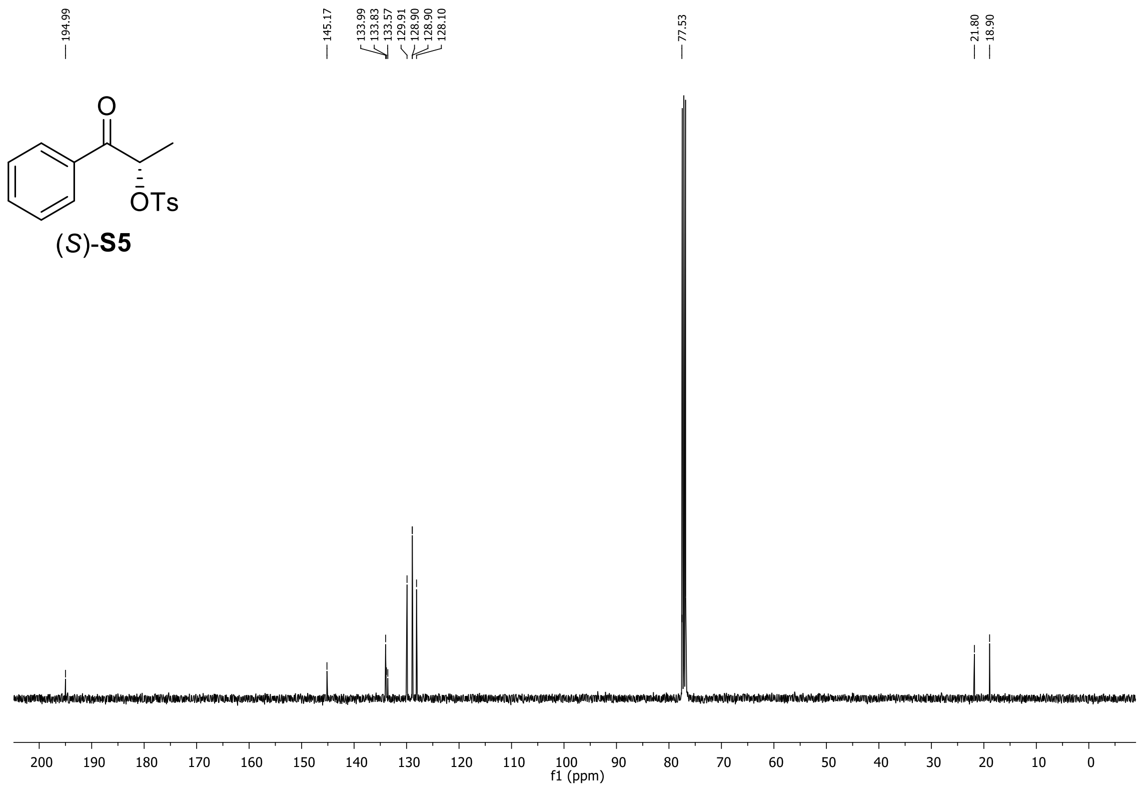

9 HPLC Chromatograms

(*R*)-1-Oxo-1-phenylpropan-2-yl acetate ((*R*)-5a)

HPLC-analysis: Lux® 5 µm Cellulose-1, LC Column (250 x 4.6 mm), *n*-hexane/*i*-PrOH = 96:4, 1.0 mL/min, 254 nm, t_R (minor) = 8.7 min, t_R (major) = 17.3 min, 84% *ee*. The absolute configuration was determined by comparison of the HPLC data with the literature.[15]

Racemic mixture:

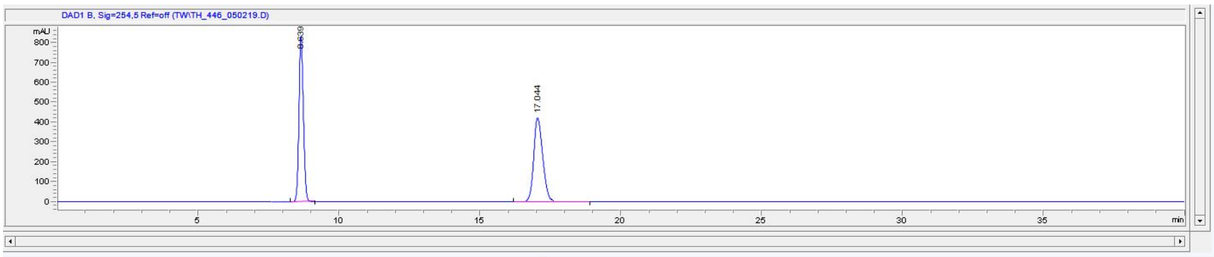

File Information	
LC-File	TH_446_050219.D
File Path	C:\CHEM32\1\DATA\TW
Date	05-Feb-19, 12:47:26
Sample	TH_446_050219
Sample Info	10 uL inj, 1mL/min 96% Hex:IPA 20oC
Barcode	
Operator	SYSTEM
Method	TW_TH_CHRIACELL_LUX_050219.M

#	Time	Area	Height	Width	Area%	Symmetry
1	8.639	9136.5	837.5	0.1673	49.303	0.792
2	17.044	9394.9	424.2	0.3384	50.697	0.732

Enantioenriched mixture:

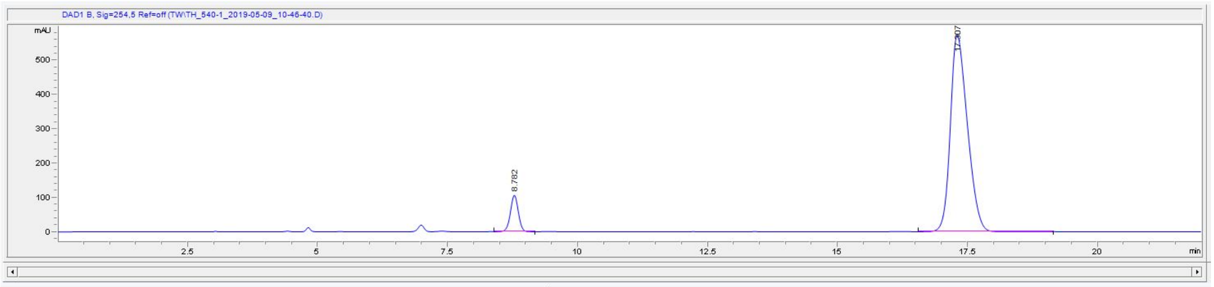

File Information	
LC-File	TH_540-1_2019-05-09_10-46-40.D
File Path	C:\CHEM32\1\DATA\TW
Date	09-May-19, 10:47:35
Sample	TH_540-1
Sample Info	10 uL, 1 mL/min, 96:4 Hexane : IPA, 20 C, Lux Cellulose
Barcode	
Operator	SYSTEM
Method	TW_TH_CHRIACELL_LUX_050219.M
Reference	

#	Time	Area	Height	Width	Area%	Symmetry
1	8.782	1117.2	105.5	0.1616	7.900	0.976
2	17.307	13025.2	570.9	0.3502	92.100	0.648

(*R*)-1-(4-Fluorophenyl)-1-oxopropan-2-yl acetate ((*R*)-5b)

HPLC-analysis: Lux® 5 µm Cellulose-1, LC Column (250 x 4.6 mm), *n*-hexane/*i*-PrOH = 96:4, 1.0 mL/min, 254 nm, t_R (minor) = 8.8 min, t_R (major) = 9.7 min, 78% *ee*. The absolute configuration was determined by comparison of the HPLC data and the optical rotation with the literature.[15]

Racemic mixture:

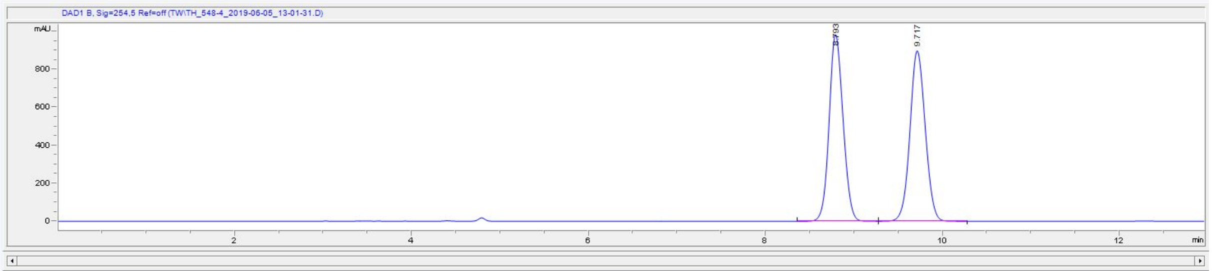

File Information	
LC-File	TH_548-4_2019-06-05_13-01-31.D
File Path	C:\CHEM32\1\DATA\TW
Date	05-Jun-19, 13:02:26
Sample	TH 548-4

#	Time	Area	Height	Width	Area%	Symmetry
1	8.793	10788.1	987.4	0.1695	49.915	0.838
2	9.717	10825	901.6	0.1861	50.085	0.891

Enantioenriched mixture:

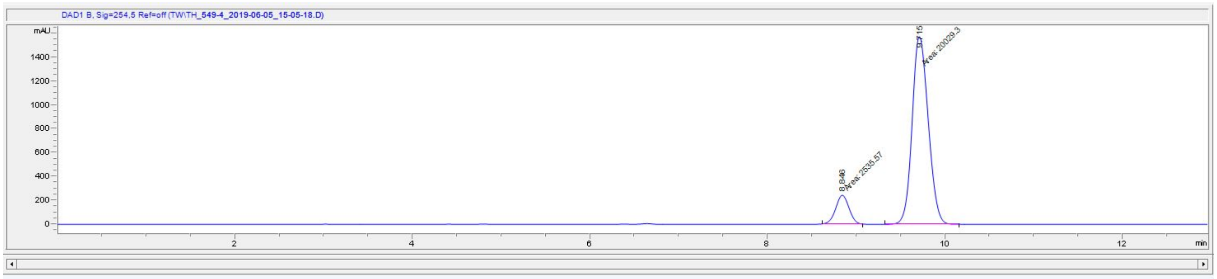

File Information	
LC-File	TH_549-4_2019-06-05_15-05-18.D
File Path	C:\CHEM32\1\DATA\TW
Date	05-Jun-19, 15:06:13
Sample	TH 549-4

#	Time	Area	Height	Width	Area%	Symmetry
1	8.846	2535.6	242.6	0.1742	11.237	0.987
2	9.715	20029.3	1580.7	0.2112	88.763	0.801

(*R*)-1-(3-Fluorophenyl)-1-oxopropan-2-yl acetate ((*R*)-5c)

HPLC-analysis: Lux® 5 µm Cellulose-1, LC Column (250 x 4.6 mm), *n*-hexane/*i*-PrOH = 96:4, 1.0 mL/min, 280 nm, t_R (minor) = 8.1 min, t_R (major) = 8.9 min, 88% *ee*. The determination of the absolute configuration of further products suggests an (*R*)-configuration for all products.

Racemic mixture:

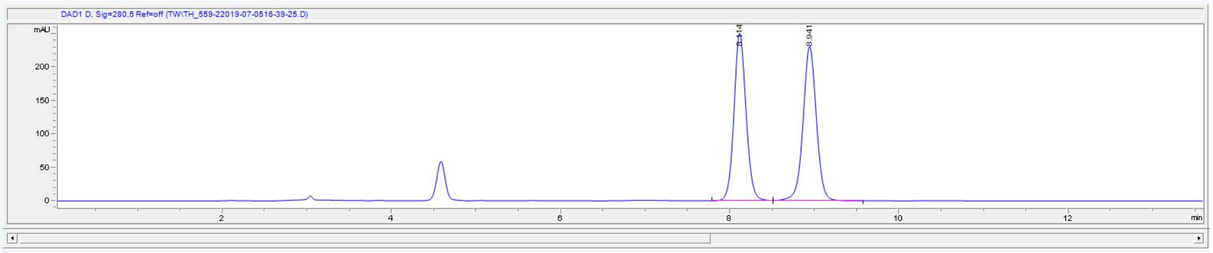

File Information	
LC-File	TH_559-22019-07-0516-39-25.D
File Path	C:\CHEM32\1\DATA\TW
Date	05-Jul-19, 16:40:18
Sample	TH_559-2
Sample Info	10 uL, 1 mL/min, 96:4 Hex:IPA, 20 degC, Lux Cellulose
Barcode	
Operator	SYSTEM

#	Time	Area	Height	Width	Area%	Symmetry
1	8.114	2481.7	249.9	0.1519	49.588	0.859
2	8.941	2522.9	229.9	0.168	50.412	0.96

Enantioenriched mixture:

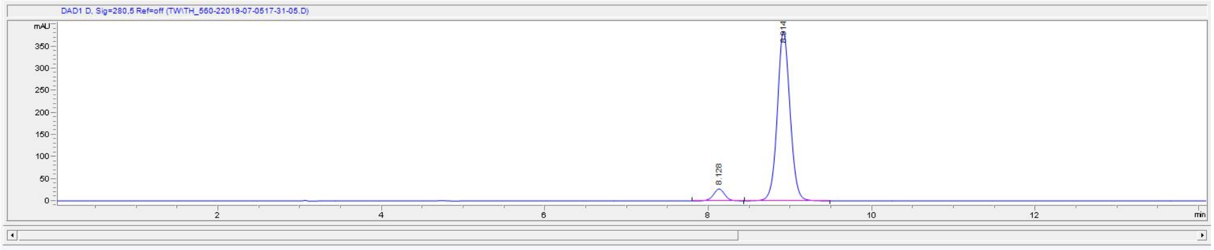

File Information	
LC-File	TH_560-22019-07-0517-31-05.D
File Path	C:\CHEM32\1\DATA\TW
Date	05-Jul-19, 17:31:58
Sample	TH_560-2
Sample Info	10 uL, 1 mL/min, 96:4 Hex:IPA, 20 degC, Lux Cellulose
Barcode	
Operator	SYSTEM

#	Time	Area	Height	Width	Area%	Symmetry
1	8.128	260.1	27.1	0.1479	5.869	0.998
2	8.914	4172.4	385.6	0.1662	94.131	0.89

(*R*)-1-(4-Bromophenyl)-1-oxopropan-2-yl acetate ((*R*)-5d)

HPLC-analysis: Lux® 5 µm Amylose-1, LC Column (250 x 4.6 mm), *n*-hexane/*i*-PrOH = 96:4, 1.0 mL/min, 254 nm, t_R (minor) = 9.2 min, t_R (major) = 12.5 min, 86% *ee*.

Racemic mixture:

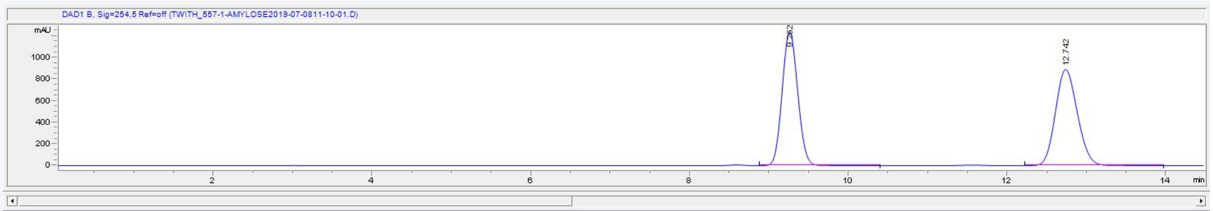

File Information	
LC-File	TH_557-1-AMYLOSE2019-07-0811-10-01.D
File Path	C:\CHEM32\1\DATA\TW
Date	08-Jul-19, 11:10:56
Sample	TH_557-1-Amylose
Sample Info	5 uL, 1 mL/min, 96:4 Hex:IPA, 15 degC, Lux Amylose
Barcode	
Operator	SYSTEM
Method	TW_TH_CHRIACELL_LUX_050219.M
Reference	

#	Time	Area	Height	Width	Area%	Symmetry
1	9.262	16755.9	1232.6	0.2126	49.770	0.896
2	12.742	16911.1	884.1	0.2972	50.230	0.84

Enantioenriched mixture:

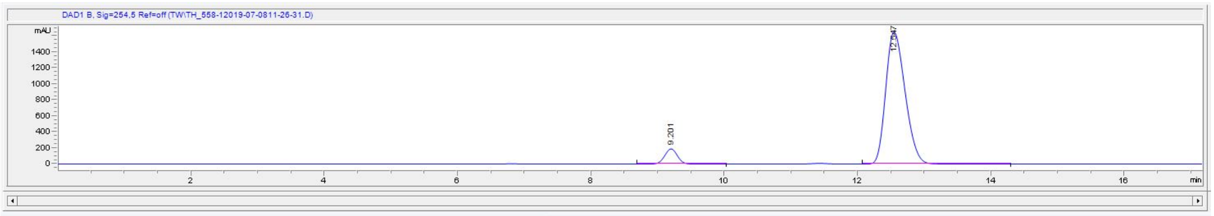

File Information	
LC-File	TH_558-12019-07-0811-26-31.D
File Path	C:\CHEM32\1\DATA\TW
Date	08-Jul-19, 11:27:25
Sample	TH_558-1
Sample Info	10 uL, 1 mL/min, 96:4 Hex:IPA, 20 degC, Lux Amylose
Barcode	
Operator	SYSTEM
Method	TW_TH_CHRIACELL_LUX_050219.M
Reference	

#	Time	Area	Height	Width	Area%	Symmetry
1	9.201	2481.1	188.3	0.2058	6.968	0.987
2	12.547	33125.7	1641.1	0.3155	93.032	0.722

(*R*)-1-Oxo-1-(3-(trifluoromethyl)phenyl)propan-2-yl acetate ((*R*)-5e)

HPLC-analysis: Lux® 5 µm Cellulose-1, LC Column (250 x 4.6 mm), *n*-hexane/*i*-PrOH = 96:4, 1.0 mL/min, 209 nm, t_R (major) = 15.1 min, t_R (minor) = 16.0 min, 81% *ee*.

Racemic mixture:

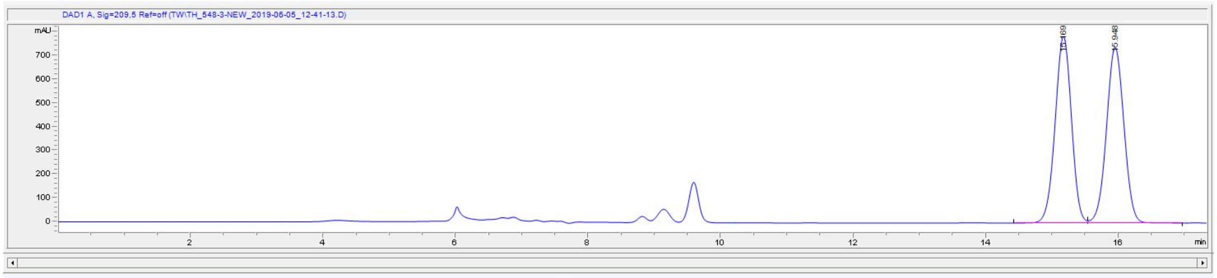

File Information

LC-File	TH_548-3-NEW_2019-06-05_12-41-13.D
File Path	C:\CHEM32\1\DATA\TW
Date	05-Jun-19, 12:42:06
Sample	TH_548-3-new

#	Time	Area	Height	Width	Area%	Symmetry
1	15.169	13490.5	787.4	0.2679	49.821	1.025
2	15.948	13587.4	741.7	0.2857	50.179	0.953

Enantioenriched mixture:

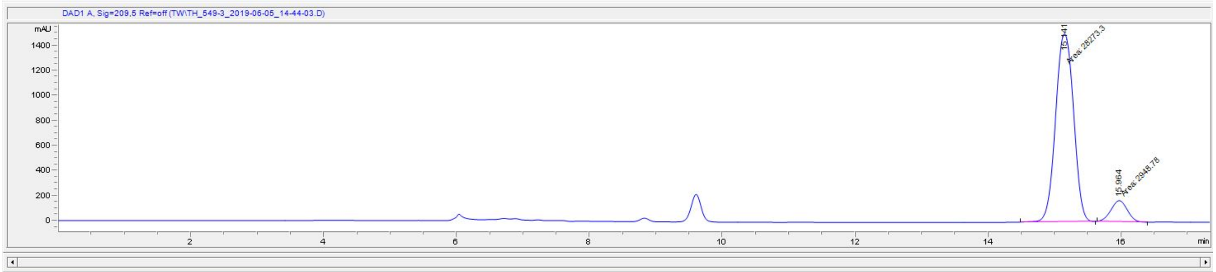

File Information

LC-File	TH_549-3_2019-06-05_14-44-03.D
File Path	C:\CHEM32\1\DATA\TW
Date	05-Jun-19, 14:44:58
Sample	TH_549-3

#	Time	Area	Height	Width	Area%	Symmetry
1	15.141	28273.3	1500.5	0.314	90.555	0.965
2	15.964	2948.8	170.1	0.2889	9.445	1.008

(*R*)-1-Oxo-1-(2-(trifluoromethyl)phenyl)propan-2-yl acetate ((*R*)-5f)

HPLC-analysis: Lux® 5 µm Cellulose-1, LC Column (250 x 4.6 mm), *n*-hexane/*i*-PrOH = 96:4, 1.0 mL/min, 209 nm, t_R (minor) = 7.6 min, t_R (major) = 10.5 min, 6% *ee*.

Racemic mixture:

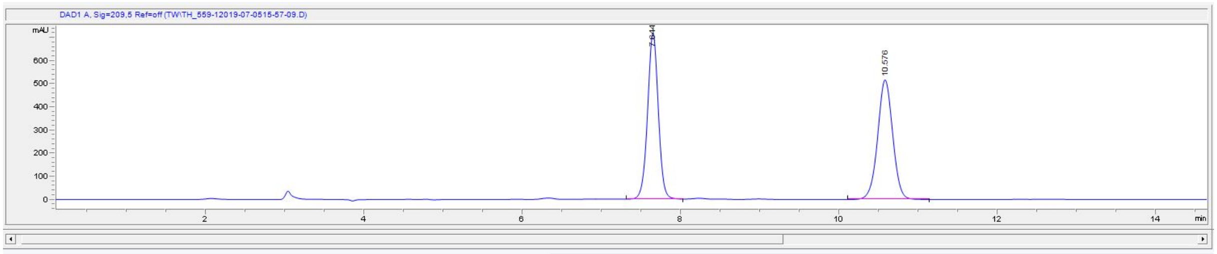

File Information

LC-File	TH_559-12019-07-0515-57-09.D
File Path	C:\CHEM32\1\DATA\TW
Date	05-Jul-19, 15:58:02
Sample	TH_559-1
Sample Info	10 uL, 1 mL/min, 96:4 Hex:IPA, 20 degC, Lux Cellulose, racemic
Barcode	
Operator	SYSTEM

#	Time	Area	Height	Width	Area%	Symmetry
1	7.644	6727.5	713.4	0.146	49.765	0.97
2	10.576	6791	514	0.2021	50.235	0.954

Enantioenriched mixture:

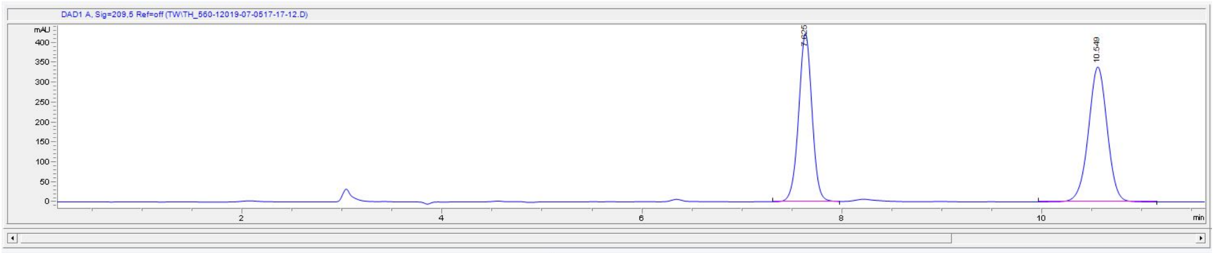

File Information	
LC-File	TH_560-12019-07-0517-17-12.D
File Path	C:\CHEM32\1\DATA\TW
Date	05-Jul-19, 17:18:04
Sample	TH_560-1
Sample Info	10 uL, 1 mL/min, 96:4 Hex:IPA, 20 degC, Lux Cellulose
Barcode	
Operator	SYSTEM

#	Time	Area	Height	Width	Area%	Symmetry
1	7.625	3912.3	421.2	0.1444	46.949	0.989
2	10.549	4420.7	337.9	0.2026	53.051	0.991

(*R*)-1-(3-Nitrophenyl)-1-oxopropan-2-yl acetate ((*R*)-5g)

HPLC-analysis: Lux® 5 µm Cellulose-1, LC Column (250 x 4.6 mm), *n*-hexane/*i*-PrOH = 96:4, 1.0 mL/min, 254 nm, t_R (major) = 27.0 min, t_R (minor) = 28.6 min, 59% *ee*.

Racemic mixture:

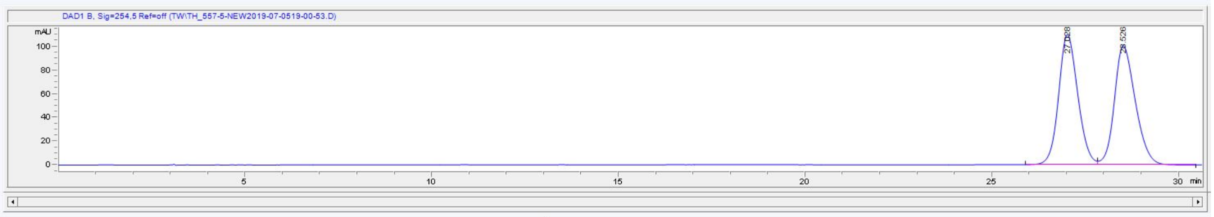

File Information	
LC-File	TH_557-5-NEW2019-07-0519-00-53.D
File Path	C:\CHEM32\1\DATA\TW
Date	05-Jul-19, 19:01:42
Sample	TH_557-5-new
Sample Info	5 uL, 1 mL/min, 96:4 Hex:IPA, 15 degC, Lux Cellulose
Barcode	
Operator	SYSTEM
Method	TW_TH_CHRIACELL_LUX_050219.M
Reference	

#	Time	Area	Height	Width	Area%	Symmetry
1	27.028	3937.8	110.2	0.5488	50.008	0.822
2	28.526	3936.4	100.9	0.5951	49.992	0.715

Enantioenriched mixture:

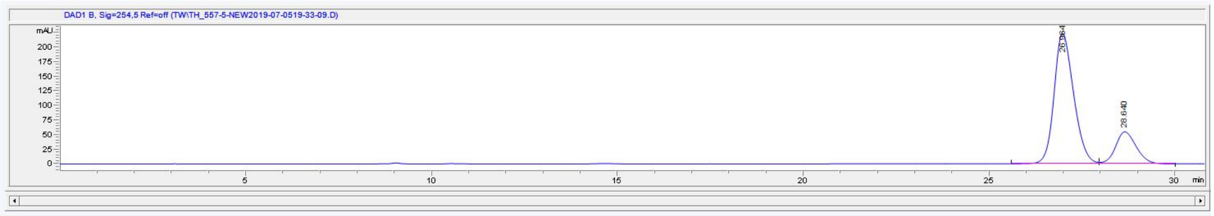

File Information	
LC-File	TH_557-5-NEW2019-07-0519-33-09.D
File Path	C:\CHEM32\1\DATA\TW
Date	05-Jul-19, 19:33:59
Sample	TH_557-5-new
Sample Info	5 uL, 1 mL/min, 96:4 Hex:IPA, 15 degC, Lux Cellulose
Barcode	
Operator	SYSTEM
Method	TW_TH_CHRIACELL_LUX_050219.M
Reference	

#	Time	Area	Height	Width	Area%	Symmetry
1	26.964	8169.5	224.1	0.5594	79.381	0.734
2	28.64	2122	54.9	0.5868	20.619	0.794

(*R*)-1-Oxo-1-(*p*-tolyl)propan-2-yl acetate ((*R*)-5h)

HPLC-analysis: Lux® 5 µm Cellulose-1, LC Column (250 x 4.6 mm), *n*-hexane/*i*-PrOH = 96:4, 1.0 mL/min, 254 nm, t_R (minor) = 8.6 min, t_R (major) = 12.9 min, 77% *ee*. The absolute configuration was determined by comparison of the HPLC data and the optical rotation with the literature.[15]

Racemic mixture:

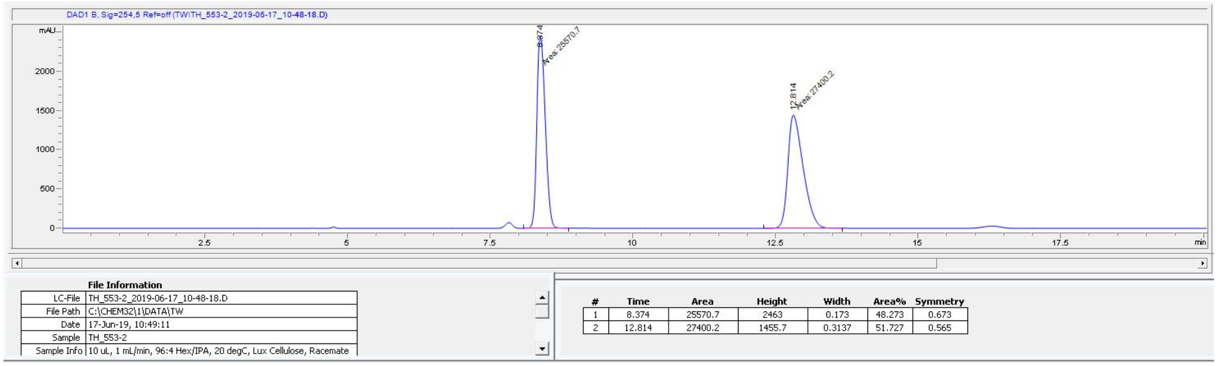

File Information	
LC-File	TH_553-2_2019-06-17_10-48-18.D
File Path	C:\CHEM32\1\DATA\TW
Date	17-Jun-19, 10:49:11
Sample	TH_553-2
Sample Info	10 uL, 1 mL/min, 96:4 Hex/IPA, 20 degC, Lux Cellulose, Racemate

#	Time	Area	Height	Width	Area%	Symmetry
1	8.374	25570.7	2463	0.173	48.273	0.673
2	12.814	27400.2	1455.7	0.3137	51.727	0.565

Enantioenriched mixture:

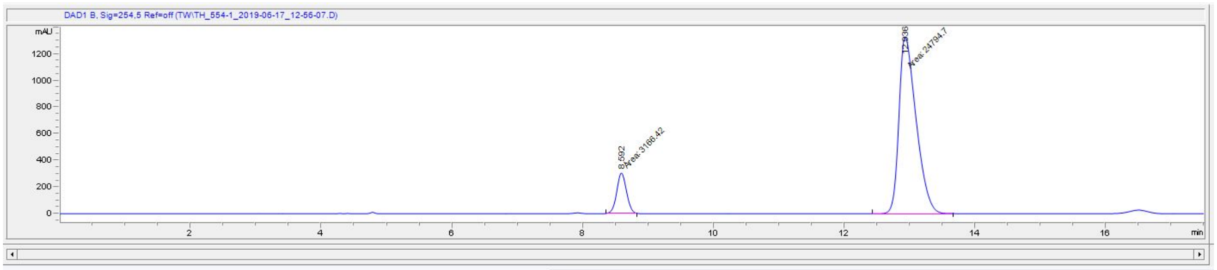

File Information	
LC-File	TH_554-1_2019-06-17_12-56-07.D
File Path	C:\CHEM32\1\DATA\TW
Date	17-Jun-19, 12:57:00
Sample	TH_554-1
Sample Info	10 uL, 1 mL/min, 96:4 Hex/IPA, 20 degC, Lux Cellulose, non racemic
Barcode	

#	Time	Area	Height	Width	Area%	Symmetry
1	8.592	3166.4	304.8	0.1732	11.324	0.908
2	12.936	24794.7	1341.2	0.3081	88.676	0.567

(*R*)-1-(4-(*tert*-Butyl)phenyl)-1-oxopropan-2-yl acetate ((*R*)-5i)

HPLC-analysis: Lux® 5 µm Cellulose-1, LC Column (250 x 4.6 mm), *n*-hexane/*i*-PrOH = 96:4, 1.0 mL/min, 254 nm, t_R (minor) = 7.3 min, t_R (major) = 9.5 min, 72% *ee*.

Racemic mixture:

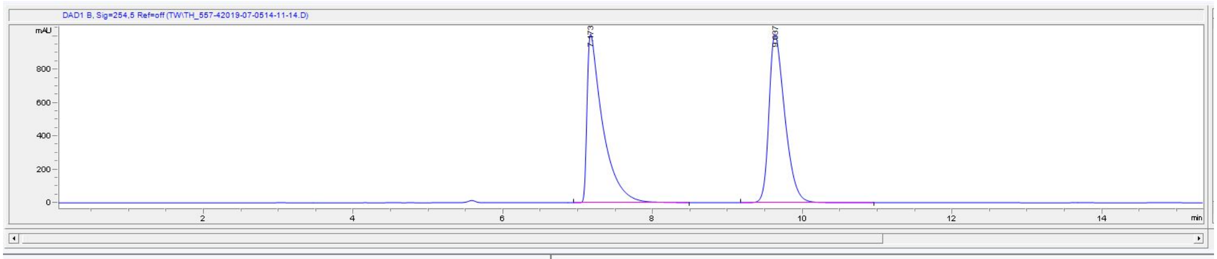

File Information	
LC-File	TH_557-42019-07-0514-11-14.D
File Path	C:\CHEM32\1\DATA\TW
Date	05-Jul-19, 14:12:08
Sample	TH_557-4
Sample Info	10 uL, 1 mL/min, 96:4 Hex:IPA, 20 degC, Lux Cellulose, racemic
Barcode	
Operator	SYSTEM

#	Time	Area	Height	Width	Area%	Symmetry
1	7.173	14700	1008.3	0.2041	49.835	0.264
2	9.637	14797	1008.1	0.2212	50.165	0.574

Enantioenriched mixture:

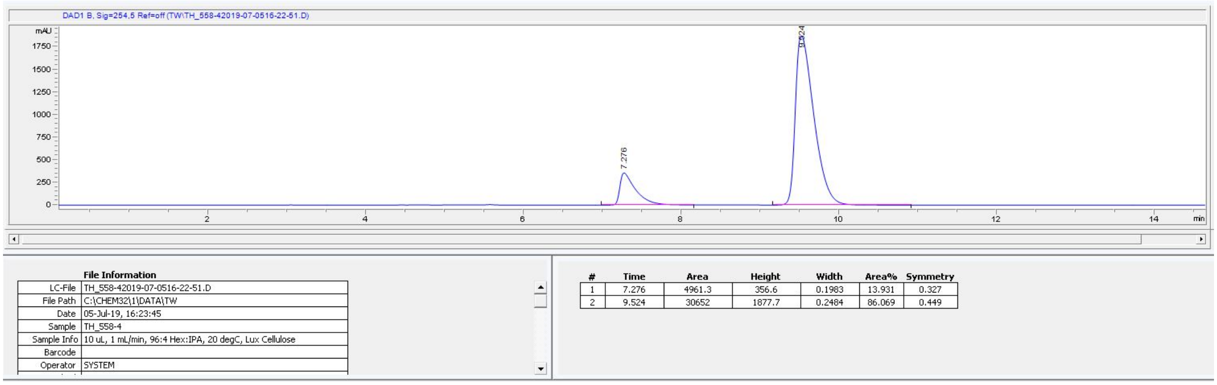

(*R*)-1-([1,1'-Biphenyl]-4-yl)-1-oxopropan-2-yl acetate ((*R*)-5j)

HPLC-analysis: Lux® 5 µm Cellulose-1, LC Column (250 x 4.6 mm), *n*-hexane/*i*-PrOH = 96:4, 1.0 mL/min, 254 nm, t_R (minor) = 13.5 min, t_R (major) = 16.9 min, 79% *ee*.

Racemic mixture:

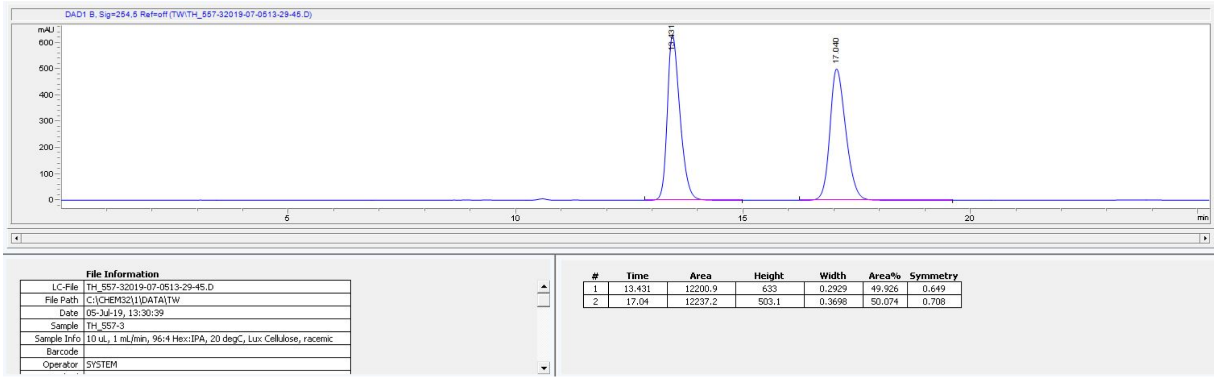

Enantioenriched mixture:

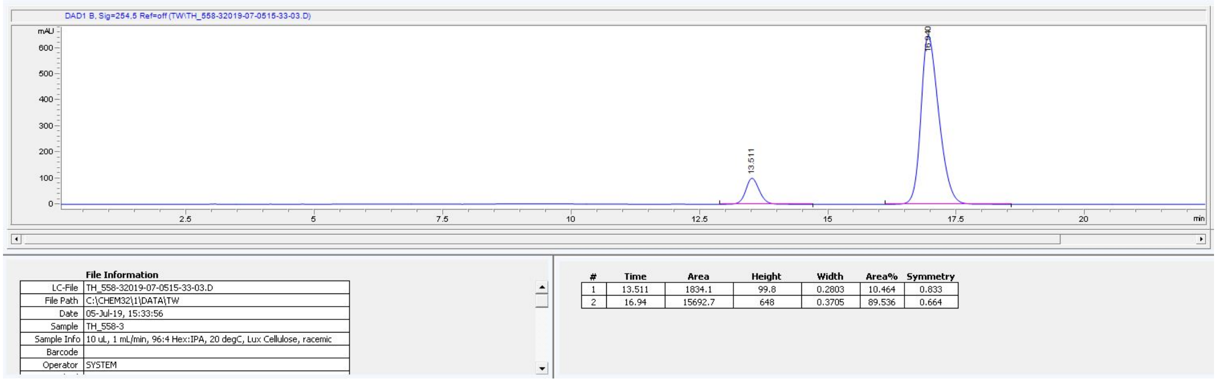

(*R*)-1-(4-Methoxyphenyl)-1-oxopropan-2-yl acetate ((*R*)-5k)

HPLC-analysis: Lux® 5 µm Cellulose-1, LC Column (250 x 4.6 mm), *n*-hexane/*i*-PrOH = 96:4, 1.0 mL/min, 254 nm, t_R (minor) = 16.0 min, t_R (major) = 31.8 min, 76% *ee*. The absolute configuration was determined by comparison of the HPLC data with the literature.[15]

Racemic mixture:

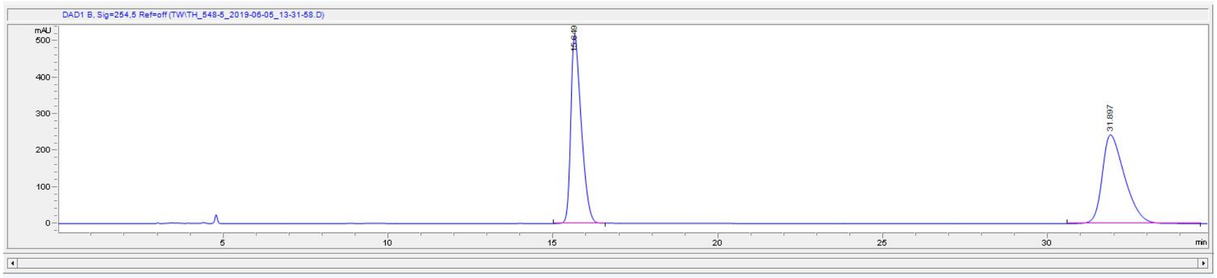

File Information

LC-File	TH_548-5_2019-06-05_13-31-58.D
File Path	C:\CHEM32\1\DATA\TW
Date	05-Jun-19, 13:32:54
Sample	TH_548-5

#	Time	Area	Height	Width	Area%	Symmetry
1	15.649	11358	514.6	0.3335	49.847	0.55
2	31.897	11427.6	242.3	0.7142	50.153	0.562

Enantioenriched mixture:

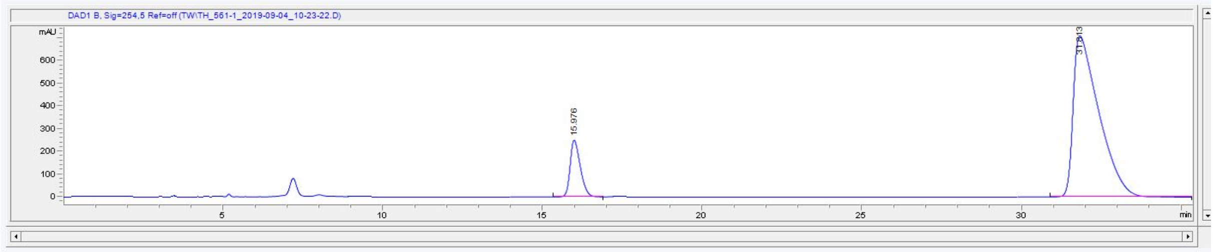

File Information

LC-File	TH_561-1_2019-09-04_10-23-22.D
File Path	C:\CHEM32\1\DATA\TW
Date	04-Sep-19, 10:24:14
Sample	TH_561-1
Sample Info	10 uL, 1 mL/min, 96:4 hex/ipa, 20 degC, LuxCellulose

#	Time	Area	Height	Width	Area%	Symmetry
1	15.976	5537.8	251	0.3354	12.244	0.625
2	31.813	39692.1	711.4	0.8249	87.756	0.329

(*R*)-2-Oxo-1,2-diphenylethyl acetate ((*R*)-5l)

HPLC-analysis: Lux® 5 µm Cellulose-1, LC Column (250 x 4.6 mm), *n*-hexane/*i*-PrOH = 96:4, 1.0 mL/min, 254 nm, t_R (minor) = 10.6 min, t_R (major) = 21.5 min, 2% *ee*.

Racemic mixture:

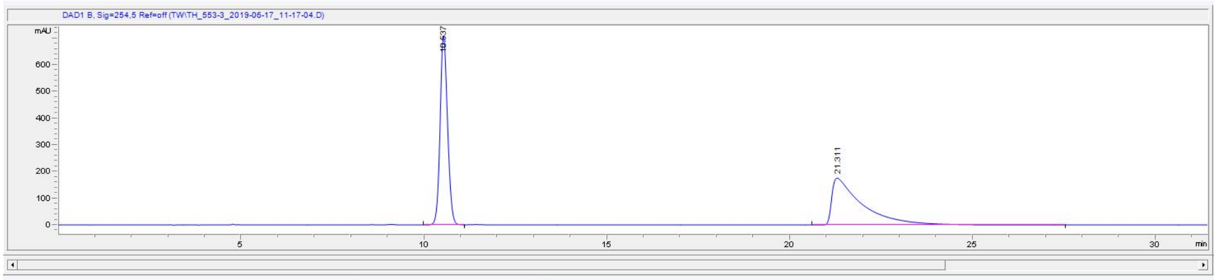

LC-File	TH_553-3_2019-06-17_11-17-04.D
File Path	C:\CHEM32\1\DATA\TW
Date	17-Jun-19, 11:17:58
Sample	TH_553-3
Sample Info	10 uL, 1 mL/min, 96:4 Hex/IPA, 20 degC, Lux Cellulose, Racemate

#	Time	Area	Height	Width	Area%	Symmetry
1	10.537	10027	707.9	0.2172	49.804	0.813
2	21.311	10105.9	174.9	0.786	50.196	0.19

Enantioenriched mixture:

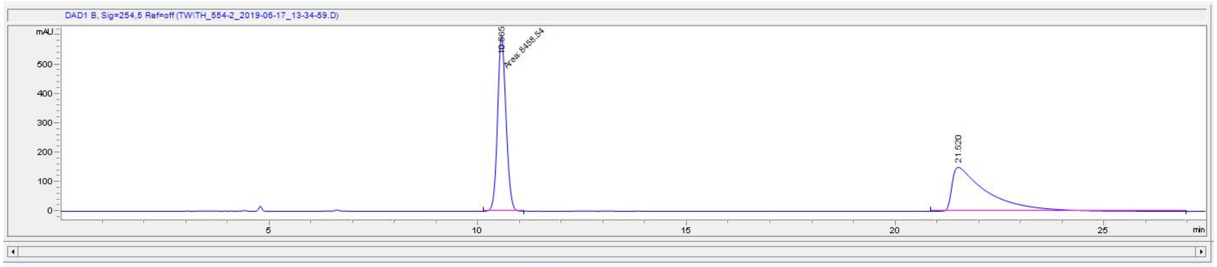

File Information

LC-File	TH_554-2_2019-06-17_13-34-59.D
File Path	C:\CHEM32\1\DATA\TW
Date	17-Jun-19, 13:35:53
Sample	TH_554-2
Sample Info	10 uL, 1 mL/min, 96:4 Hex/IPA, 20 degC, Lux Cellulose, non racemic
Barcode	

#	Time	Area	Height	Width	Area%	Symmetry
1	10.565	8458.5	596.5	0.2363	48.998	0.827
2	21.52	8804.4	150.1	0.7965	51.002	0.193

(*R*)-3-Methyl-1-oxo-1-phenylbutan-2-yl acetate ((*R*)-5m)

HPLC-analysis: Lux® 5 µm Cellulose-1, LC Column (250 x 4.6 mm), *n*-hexane/*i*-PrOH = 96:4, 0.5 mL/min, 254 nm, t_R (minor) = 13.6 min, t_R (major) = 37.3 min, 75% *ee*.

Racemic mixture:

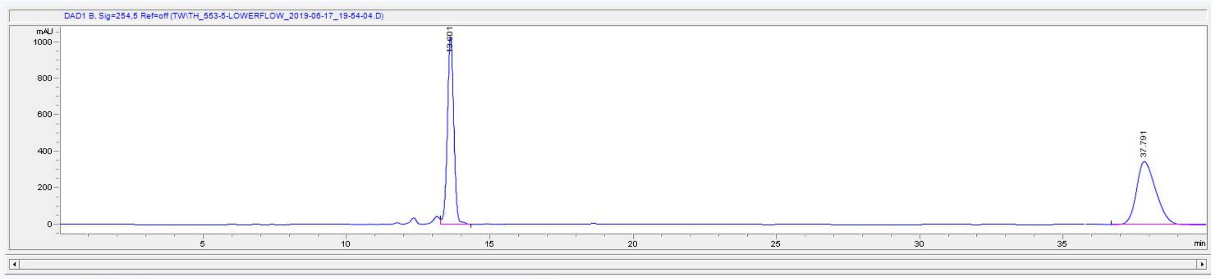

File Information

LC-File	TH_553-5-LOWERFLOW_2019-06-17_19-54-04.D
File Path	C:\CHEM32\1\DATA\TW
Date	17-Jun-19, 19:54:57
Sample	TH_553-5-lowerflow
Sample Info	10 uL, 0.5 mL/min, 96:4 Hex/IPA, 20 degC, Lux Cellulose, racemic

#	Time	Area	Height	Width	Area%	Symmetry
1	13.601	15911.5	1028.3	0.2405	49.592	0.929
2	37.791	16173.2	346.3	0.7168	50.408	0.672

Enantioenriched mixture:

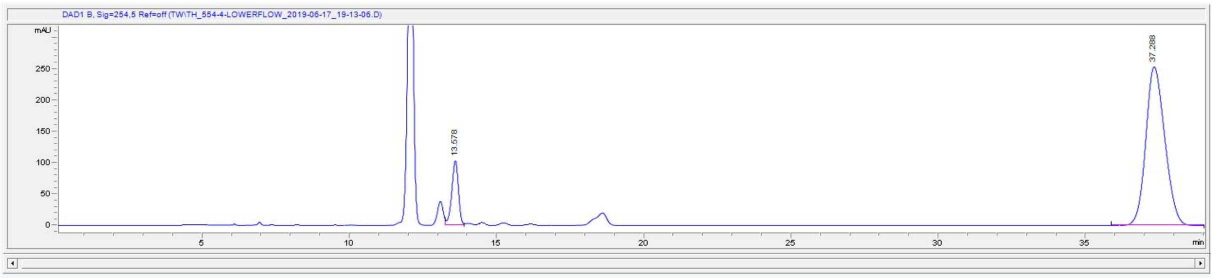

File Information

LC-File	TH_554-4-LOWERFLOW_2019-06-17_19-13-06.D
File Path	C:\CHEM32\1\DATA\TW
Date	17-Jun-19, 19:14:00
Sample	TH_554-4-lowerflow
Sample Info	10 uL, 0.5 mL/min, 96:4 Hex/IPA, 20 degC, Lux Cellulose, non racer

#	Time	Area	Height	Width	Area%	Symmetry
1	13.578	1604	103.4	0.239	12.331	1.131
2	37.288	11404.3	254.2	0.6914	87.669	0.766

(*R*)-1-Oxo-1-phenylbutan-2-yl acetate ((*R*)-5n)

HPLC-analysis: Lux® 5 µm Cellulose-1, LC Column (250 x 4.6 mm), *n*-hexane/*i*-PrOH = 96:4, 1.0 mL/min, 254 nm, t_R (minor) = 7.5 min, t_R (major) = 17.5 min, 84% *ee*.

Racemic mixture:

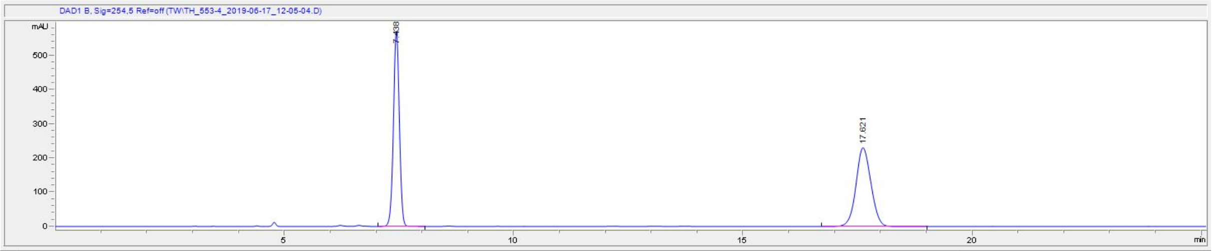

File Information	
LC-File	TH_553-4_2019-06-17_12-05-04.D
File Path	C:\CHEM32\1\DATA\TW
Date	17-Jun-19, 12:05:58
Sample	TH_553-4
Sample Info	10 uL, 1 mL/min, 96:4 Hex/IPA, 20 degC, Lux Cellulose, Racemate

#	Time	Area	Height	Width	Area%	Symmetry
1	7.438	5117	568.4	0.139	49.596	0.963
2	17.621	5200.4	229.3	0.3506	50.404	0.87

Enantioenriched mixture:

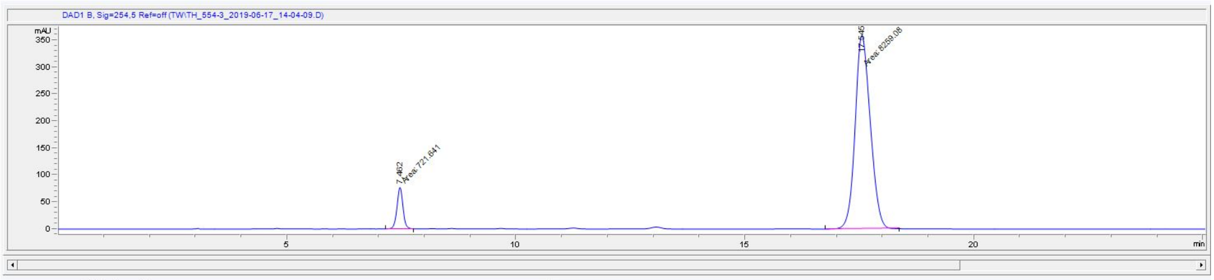

File Information	
LC-File	TH_554-3_2019-06-17_14-04-09.D
File Path	C:\CHEM32\1\DATA\TW
Date	17-Jun-19, 14:05:03
Sample	TH_554-3
Sample Info	10 uL, 1 mL/min, 96:4 Hex/IPA, 20 degC, Lux Cellulose, non racemic

#	Time	Area	Height	Width	Area%	Symmetry
1	7.462	721.6	77	0.1562	8.035	1.011
2	17.545	8259.1	357.3	0.3852	91.965	0.782

(*R*)-1-Oxo-1,2,3,4-tetrahydronaphthalen-2-yl acetate ((*R*)-5o)

HPLC-analysis: Lux® 5 µm Cellulose-1, LC Column (250 x 4.6 mm), *n*-hexane/*i*-PrOH = 96:4, 1.0 mL/min, 254 nm, t_R (major) = 15.8 min, t_R (minor) = 17.7 min, 9% *ee*. The absolute configuration was determined by comparison of the optical rotation with the literature.[21]

Racemic mixture:

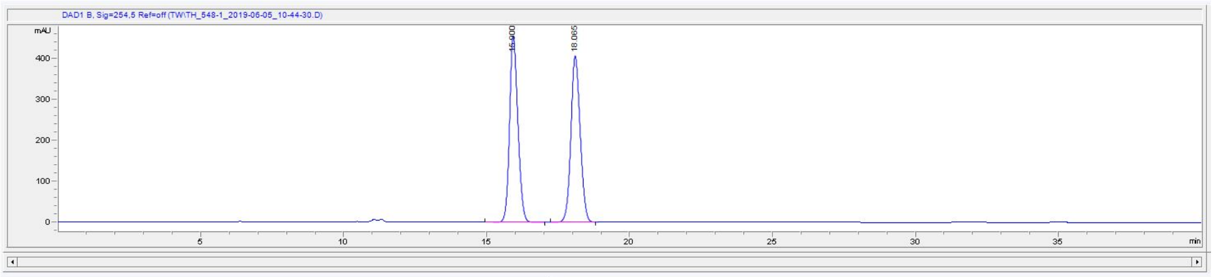

File Information	
LC-File	TH_548-1_2019-06-05_10-44-30.D
File Path	C:\CHEM32\1\DATA\TW
Date	05-Jun-19, 10:45:23
Sample	TH_548-1

#	Time	Area	Height	Width	Area%	Symmetry
1	15.9	9172.6	455.6	0.3069	50.072	0.761
2	18.065	9146.3	405.8	0.347	49.928	0.832

Enantioenriched mixture:

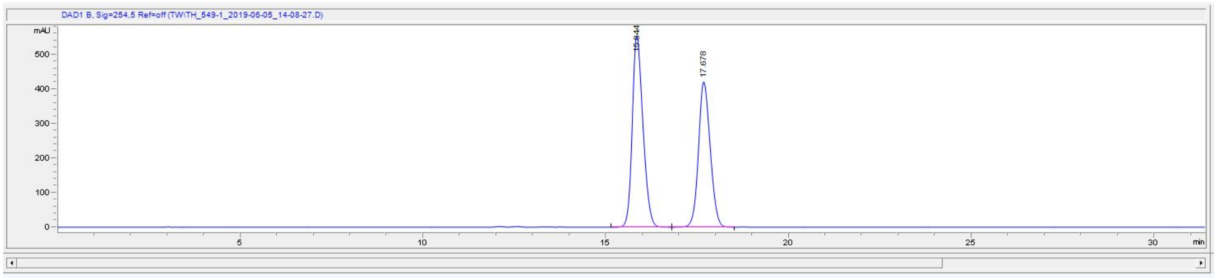

File Information

LC-File	TH_549-1_2019-06-05_14-08-27.D
File Path	C:\CHEM32\1\DATA\TW
Date	05-Jun-19, 14:09:23
Sample	TH 549-1

#	Time	Area	Height	Width	Area%	Symmetry
1	15.844	11268	553.5	0.3115	54.588	0.695
2	17.678	9373.9	420.7	0.342	45.412	0.804

(*R*)-1-Oxo-1-(thiophen-2-yl)propan-2-yl acetate ((*R*)-5p)

HPLC-analysis: Lux® 5 µm Cellulose-1, LC Column (250 x 4.6 mm), *n*-hexane/*i*-PrOH = 96:4, 1.0 mL/min, 254 nm, t_R (minor) = 12.1 min, t_R (major) = 23.2 min, 69% *ee*.

Racemic mixture:

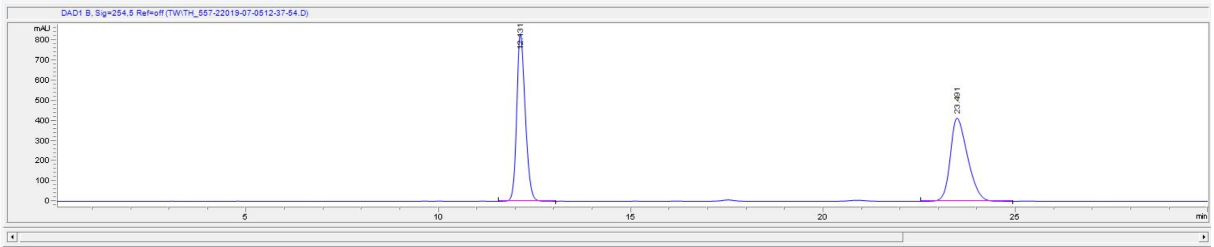

File Information

LC-File	TH_557-22019-07-0512-37-54.D
File Path	C:\CHEM32\1\DATA\TW
Date	05-Jul-19, 12:38:48
Sample	TH_557-2
Sample Info	10 uL, 1 mL/min, 96:4 Hex:IPA, 20 degC, Lux Cellulose, racemic
Barcode	
Operator	SYSTEM
Method	TW_TH_CHRIACELL_LUX_050219.M

#	Time	Area	Height	Width	Area%	Symmetry
1	12.131	12887.5	835	0.2341	49.823	0.719
2	23.491	12978.9	414	0.4777	50.177	0.626

Enantioenriched mixture:

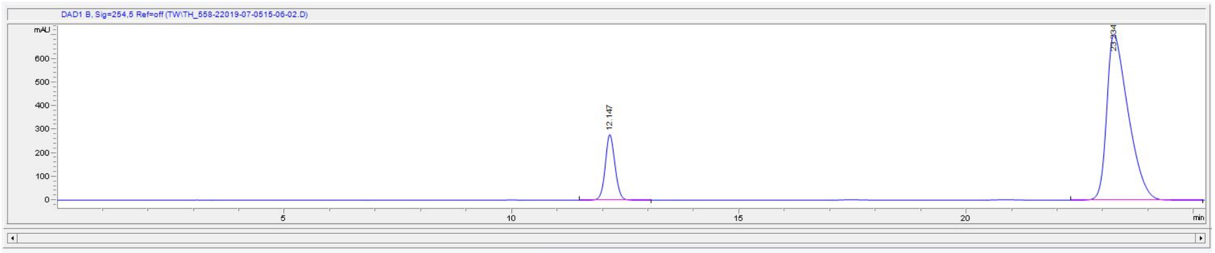

File Information

LC-File	TH_558-22019-07-0515-06-02.D
File Path	C:\CHEM32\1\DATA\TW
Date	05-Jul-19, 15:06:54
Sample	TH_558-2
Sample Info	10 uL, 1 mL/min, 96:4 Hex:IPA, 20 degC, Lux Cellulose, racemic
Barcode	
Operator	SYSTEM

#	Time	Area	Height	Width	Area%	Symmetry
1	12.147	4151	278.6	0.2279	15.270	0.859
2	23.234	23032.7	704.7	0.4893	84.730	0.486

(2*S*)-2-[(4-Methylbenzenesulfonyl)oxy]-1-phenylpropan-1-one ((*S*)-S5)

HPLC-analysis: YMC Chiral Amylose-C S-5µm (25 cm), *n*-hexane/*i*-PrOH = 85:15, 0.7 mL/min, 226 nm t_R (minor) = 16.0 min, t_R (major) = 17.9 min, 87% *ee*. The absolute configuration was determined by comparison of the HPLC data[20] and the optical rotation[22] with the literature.

Enantioenriched mixture:

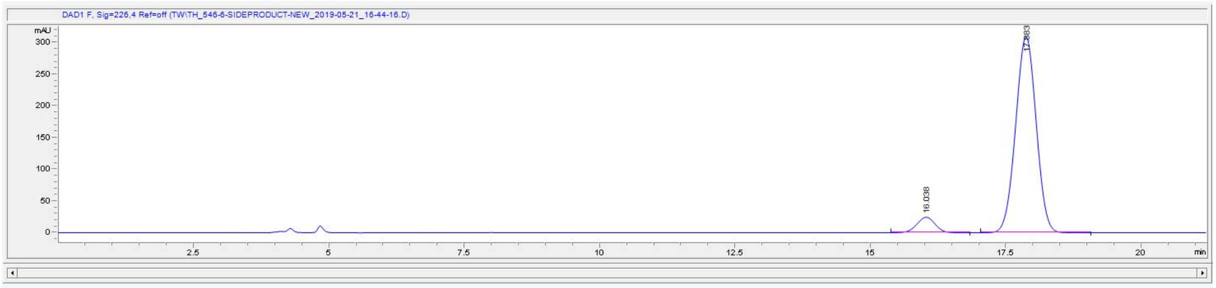

File Information

LC-File	TH_546-6-SIDEPRODUCT-NEW_2019-05-21_16-44-16.D
File Path	C:\CHEM32\1\DATA\TW
Date	21-May-19, 16:45:08
Sample	TH_546-6-sideproduct-new
Sample Info	10 uL, 0.7 mL/min, 85:15 Hexane:IPA, 20 degC, YMC
Barcode	
Operator	SYSTEM
Method	DEF_LC.M
Reference	

#	Time	Area	Height	Width	Area%	Symmetry
1	16.038	571.8	24.4	0.3651	6.533	1.03
2	17.883	8181.6	310.2	0.4137	93.467	0.996

10 Cyclic Voltammetry

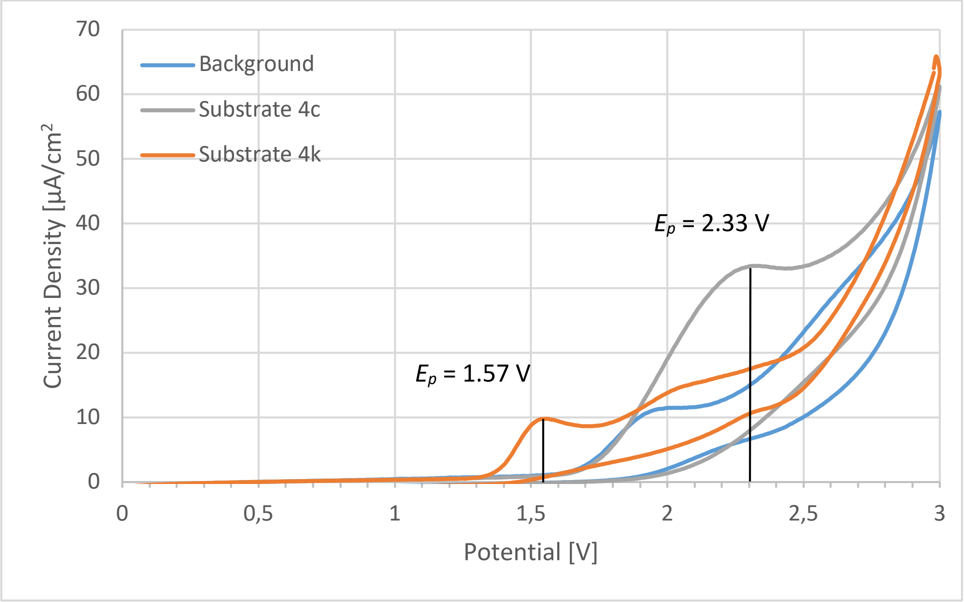

Figure S1: Cyclic voltammetry measurements of substrate **4c** (4 mM, grey line) and **4k** (4 mM, orange line) in CH_2Cl_2/HOAc (3:1); working electrode = glassy carbon; counter electrode = Pt wire; reference electrode = Ag/AgCl 0.01 M; supporting electrolyte = 0.1 M Bu_4NClO_4; scan rate = 20 mVs^{-1}. A blank CV (blue, 0.1 M in Bu_4NClO_4 CH_2Cl_2/HOAc (3:1) is inserted for comparison.

11 Literature

[1] M. Anada, M. Tanaka, T. Washio, M. Yamawaki, T. Abe, S. Hashimoto, *Org. Lett.* **2007**, *9*, 4559–4562.

[2] J. Eames, G. S. Coumbarides, M. J. Suggate, N. Weerasooriya, *Eur. J. Org. Chem.* **2003**, 634–641.

[3] B. Basdevant, C. Y. Legault, *J. Org. Chem.* **2015**, *80*, 6897–6902.

[4] S. Dupuy, D. Gasperini, S. P. Nolan, *ACS Catal.* **2015**, *5*, 6918–6921.

[5] H. Maekawa, K. Itoh, S. Goda, I. Nishiguchi, *Chirality* **2003**, *15*, 95–100.

[6] D. Gärtner, A. L. Stein, S. Grupe, J. Arp, A. Jacobi von Wangelin, *Angew. Chem. Int. Ed.* **2015**, *54*, 10545–10549; *Angew. Chem.* **2015**, *127*, 10691–10695.

[7] S. Tsujiyama, K. Suzuki, *Org. Synth.* **2007**, *84*, 272.

[8] S. Haubenreisser, T. H. Wöste, C. Martínez, K. Ishihara, K. Muñiz, *Angew. Chem. Int. Ed.* **2016**, *55*, 413–417; *Angew. Chem.* **2016**, *128*, 422–426.

[9] J. Shi, H. Xu, D. Qiu, J. He, Y. Li, *J. Am. Chem. Soc.* **2017**, *139*, 623–626.

[10] M. Jereb, M. Zupan, S. Stavber, *Chem. Commun.* **2004**, 2614–2615.

[11] M. Fujita, Y. Yoshida, K. Miyata, A. Wakisaka, T. Sugimura, *Angew. Chem. Int. Ed.* **2010**, *49*, 7068–7071; *Angew. Chem.* **2010**, *122*, 7222–7225.

[12] M. Uyanik, T. Yasui, K. Ishihara, *Angew. Chem. Int. Ed.* **2010**, *49*, 2175–2177; *Angew. Chem.* **2010**, *122*, 2221–2223.

[13] R. Pluta, P. E. Krach, L. Cavallo, L. Falivene, M. Rueping, *ACS Catal.* **2018**, *8*, 2582–2588.

[14] K. Muñiz, L. Barreiro, R. M. Romero, C. Martínez, *J. Am. Chem. Soc.* **2017**, *139*, 4354–4357.

[15] T. Sun, X. Zhang, *Adv. Synth. Catal.* **2012**, *354*, 3211–3215.

[16] T. Nagano, Z. Jia, X. Li, M. Yan, G. Lu, A. S. C. Chan, T. Hayashi, *Chem. Lett.* **2010**, *39*, 929–931.

[17] C. Zhao, L. Li, Q. Liu, C. Pan, G. Su, D. Mo, *Org. Biomol. Chem.* **2016**, *14*, 6795–6803.

[18] J. Sheng, X. Li, M. Tang, B. Gao, G. Huang, *Synthesis* **2007**, *8*, 1165–1168.

[19] E. Speckmeier, P. J. W. Fuchs, K. Zeitler, *Chem. Sci.* **2018**, *9*, 7096–7103.

[20] G. Levitre, A. Dumoulin, P. Retailleau, A. Panossian, F. R. Leroux, G. Masson, *J. Org. Chem.* **2017**, *82*, 11877–11883.

[21] W. Adam, M. T. Díaz, R. T. Fell, C. R. Saha-Möller, *Tetrahedron: Asymmetry* **1996**, *7*, 2207–2210.

[22] S. Brenet, F. Berthiol, J. Einhorn, *Eur. J. Org. Chem.* **2013**, 8094–8096.
